# Comparative efficacy and safety of vaccines to prevent seasonal influenza: A systematic review and network meta-analysis

**DOI:** 10.1016/j.eclinm.2022.101331

**Published:** 2022-03-25

**Authors:** Silvia Minozzi, Theodore Lytras, Silvia Gianola, Marien Gonzalez-Lorenzo, Greta Castellini, Cristina Galli, Danilo Cereda, Stefanos Bonovas, Elena Pariani, Lorenzo Moja

**Affiliations:** a Department of Epidemiology, Lazio regional health Service, Rome, Italy; b School of Medicine, European University Cyprus, Nicosia, Cyprus; c IRCCS Istituto Ortopedico Galeazzi, Unit of Clinical Epidemiology, Milan, Italy; d Laboratory of Clinical Research Methodology, Istituto di Ricerche Farmacologiche Mario Negri IRCCS, Milan, Italy; e Department of Biomedical Sciences for Health, University of Milan, Milan, Italy; f Directorate General for Health, Lombardy Region, Milan, Italy; g Department of Biomedical Sciences, IRCCS Humanitas Research Hospital, Humanitas University, Milan, Italy

**Keywords:** Influenza, Vaccines, Systematic review, Network meta-analysis, AE, adverse event, CI, confidence interval, CrI, credible interval, IIV, inactivated influenza vaccine, ILI, influenza-like illness, LAIV, live-attenuated influenza vaccine, NMA, network meta-analysis, RCT, randomized controlled trial, RIV, recombinant influenza vaccine, RR, risk ratio, SUCRA, surface under the cumulative ranking curve, 3-IIV, trivalent inactivated influenza vaccine, 3-IIV HD, trivalent inactivated high-dose influenza vaccine, 3-IIV ID, trivalent inactivated intradermal influenza vaccine, 3-IIV MF59/AS03-adj, trivalent inactivated influenza vaccine adjuvanted with MF59/AS03, 3-IIV vir/lip-adj, trivalent inactivated influenza vaccine adjuvanted with virosome/liposome, 3-RIV, trivalent recombinant influenza vaccine, 4-IIV, quadrivalent inactivated influenza vaccine, 4-IIV HD, quadrivalent inactivated high-dose influenza vaccine, 4-IIV ID, quadrivalent inactivated intradermal influenza vaccine, 4-IIV MF59/AS03-adj, quadrivalent inactivated influenza vaccine adjuvanted with MF59/AS03, 4-IIV vir/lip-adj, quadrivalent inactivated influenza vaccine adjuvanted with virosome/liposome, 4-RIV, quadrivalent recombinant influenza vaccine, 3-LAIV, trivalent live-attenuated influenza vaccine, 4-LAIV, quadrivalent live-attenuated influenza vaccine

## Abstract

**Background:**

Influenza is one of the most common respiratory viral infections worldwide. Numerous vaccines are used to prevent influenza. Their selection should be informed by the best available evidence. We aimed to estimate the comparative efficacy and safety of seasonal influenza vaccines in children, adults and the elderly.

**Methods:**

We conducted a systematic review and network meta-analysis (NMA). We searched the Cochrane Library Central Register of Controlled Trials, MEDLINE and EMBASE databases, and websites of regulatory agencies, through December 15th, 2020. We included placebo- or no vaccination-controlled, and head-to-head randomized clinical trials (RCTs). Pairs of reviewers independently screened the studies, abstracted the data, and appraised the risk of bias in accordance to the Cochrane Handbook for Systematic Reviews of Interventions. The primary outcome was laboratory-confirmed influenza. We also synthesized data for hospitalization, mortality, influenza-like illness (ILI), pneumonia or lower respiratory-tract disease, systemic and local adverse events (AEs). We estimated summary risk ratios (RR) using pairwise and NMA with random effects. This study is registered with PROSPERO, number CRD42018091895.

**Findings:**

We identified 13,439 citations. A total of 231 RCTs were included after screening: 11 studies did not provide useful data for the analysis; 220 RCTs [100,677 children (< 18 years) and 329,127 adults (18–60 years) and elderly (≥ 61 years)] were included in the NMA. In adults and the elderly, all vaccines, except the trivalent inactivated intradermal vaccine (3-IIV ID), were more effective than placebo in reducing the risk of laboratory-confirmed influenza, with a RR between 0.33 (95% credible interval [CrI] 0.21–0.55) for trivalent inactivated high-dose (3-IIV HD) and 0.56 (95% CrI 0.41–0.74) for trivalent live-attenuated vaccine (3-LAIV). In adults and the elderly, compared with trivalent inactivated vaccine (3-IIV), no significant differences were found for any, except 3-LAIV, which was less efficacious [RR 1.41 (95% CrI 1.04–1.88)]. In children, compared with placebo, RR ranged between 0.13 (95% CrI 0.03–0.51) for trivalent inactivated vaccine adjuvanted with MF59/AS03 and 0.55 (95% CrI 0.36–0.83) for trivalent inactivated vaccine. Compared with 3-IIV, 3-LAIV and trivalent inactivated adjuvanted with MF59/AS03 were more efficacious [RR 0.52 (95% CrI 0.32–0.82) and RR 0.23 (95% CrI 0.06–0.87)] in reducing laboratory-confirmed influenza. With regard to safety, higher systemic AEs rates after vaccination with 3-IIV, 3-IIV HD, 3-IIV ID, 3-IIV MF59/AS03-adj, quadrivalent inactivated (4-IIV), quadrivalent adjuvanted (4-IIV MF59/AS03-adj), quadrivalent recombinant (4-RIV), 3-LAIV or quadrivalent live attenuated (4-LAIV) vaccines were noted in adults and the elderly [RR 1.5 (95% CrI 1.18–1.89) to 1.15 (95% CrI 1.06–1.23)] compared with placebo. In children, the systemic AEs rate after vaccination was not significantly higher than placebo.

**Interpretation:**

All vaccines cumulatively achieved major reductions in the incidence of laboratory-confirmed influenza in children, adults, and the elderly. While the live-attenuated was more efficacious than the inactivated vaccine in children, many vaccine types can be used in adults and the elderly.

**Funding:**

The directorate general of welfare, Lombardy region.

**Research in context**

**Evidence before this study**

The World Health Organization recommends annual influenza vaccination for high-risk groups, but these recommendations do not favor any particular type of vaccine over others. A large number of studies confirm the superiority of any type of vaccine against placebo or no vaccine for several relevant outcomes. To date, an assessment of the merits of one vaccine over another using a network meta-analysis approach have been limited to specific populations, such as HIV patients, but have never extended to all patients irrespective of age and clinical characteristics. Literature search for this meta-analysis was performed using PubMed, Embase and Cochrane Central from 1991 to December 15th, 2020, with no language restrictions. We included RCTs that assessed the efficacy and safety of any type of trivalent/quadrivalent seasonal influenza vaccine, at the doses licensed by the European Medicines Agency and/or the US Food and Drug Administration, on children, adults and the elderly.

**Added value of this study**

Our evidence synthesis includes 220 studies and 429,804 participants and represents to the best of our knowledge the most comprehensive synthesis to date on the comparative efficacy and safety of influenza vaccines across age groups. All types of vaccines we included in our study (except trivalent inactivated intradermal) produce important gains when compared to placebo in terms of incidence of laboratory-confirmed influenza in adults and the elderly. Evidence for quadrivalent vaccines, introduced more recently, is rapidly cumulating achieving benefits similar to well-established trivalent vaccines. Most vaccines had similar efficacy profiles. Vaccines were less efficacious and less well tolerated in the elderly than in adults and children. In children, trivalent live attenuated vaccine was more efficacious than trivalent inactivated.

**Implications of all the available evidence**

Progress in efficacy of influenza vaccines has achieved consistent improvements in the prevention of influenza. Many vaccines can be used with comparable efficacy profiles. Given similar relative benefits, differences in safety and price might be considered as additional important dimensions to select vaccines in preventive strategies.

Alt-text: Unlabelled box

## Introduction

Influenza is a respiratory illness caused by influenza viruses that are transmitted efficiently from human to human.^1^ Globally, seasonal influenza affects 5–10% of adults and 20–30% of children every year^1^ and is responsible for 3–5 million cases of severe illness and up to 650,000 deaths every year.^2^ The continuing evolution of seasonal influenza viruses, which limits the ability of our immune system to fight effectively the infection, is associated with the recurrent burden of seasonal epidemics.^3^ The socioeconomic costs incurred by each influenza season are estimated at USD 87 billion per year.^4^

Influenza vaccination is a pillar of public health and is focused on people at high risk of complications - pregnant women, children, the elderly and immunocompromised, and persons with chronic illnesses - as well as those who live with or care for persons at high risk. The World Health Organization recommends annual vaccination for all high-risk groups.^1^ The American Advisory Committee on Immunization Practices recommends that all persons aged ≥ 6 months without contraindications receive routine annual vaccination with a licensed and age-appropriate vaccine.^5^ These recommendations do not favor any particular type of vaccine over others. They reflect the results of randomized clinical trials (RCTs), generally designed to test a vaccine against placebo but limiting the ability of single studies to inform immunization strategies on the most appropriate vaccine options.

Network meta-analyses (NMAs) can be applied to estimate comparative efficacy, summarize and interpret the available evidence, and identify the best vaccine types for different populations.^6^ To date, only one NMA on comparative efficacy of influenza vaccines among HIV-positive people has been published.^7^ To fill this gap, we performed a systematic review and NMA to inform influenza vaccination strategies by comparing the efficacy and safety of different types of seasonal influenza vaccines.

## Methods

We followed the PRISMA Extension Statement for Reporting of Systematic Reviews Incorporating Network Meta-analyses of Health Care Interventions guidelines.^8^ This review was registered with PROSPERO, number CRD42018091895.

### Search strategy and selection criteria

We conducted a systematic review and NMA. We searched the Cochrane Central Register of Controlled Trials (CENTRAL) (The Cochrane Library), MEDLINE (PubMed) and EMBASE from 1991 to December 15th, 2020, with no language restrictions. The full search strategy is reported in the appendix (pp 3–4). We also manually screened the citation lists from relevant literature sources (e.g., previously published systematic reviews).

We included RCTs that assessed the efficacy and safety of any type of trivalent/quadrivalent seasonal influenza vaccine, at the doses licensed by the European Medicines Agency and/or the US Food and Drug Administration, for the prevention of seasonal influenza in any individual irrespective of age and health status, i.e., healthy children (< 18 years), healthy adults (18–60 years), and the elderly (age ≥ 61 years), pregnant women, individuals of any age at risk of influenza-related complications due to pre-existing diseases (cancer, immunosuppression, chronic respiratory, cardiovascular or metabolic diseases). Studies with multiple arms comparing different dosages of vaccine were included only for the arms with licensed doses. We did not consider vaccination co-interventions, such as topical adjuvants (e.g. topical imiquimod).

The following trivalent (3-)/quadrivalent (4-) seasonal influenza vaccines were included in the review:

• Inactivated influenza vaccines (IIVs) (whole virus, split or sub-unit) administered intramuscularly (IM) or intradermically (ID). IIVs were further grouped in: MF59/AS03 (oil-in-water emulsion) adjuvanted (MF59/AS03-adj-IIVs), virosome/liposome (microparticule) adjuvanted (vir/lip-adj-IIVs), and high-dose (60 µg of hemagglutinin per strain compared with 15 µg per strain of standard-dose vaccines) (HD-IIVs).
• Live-attenuated influenza vaccines (LAIVs) administered by intranasal (IN) route.
• Recombinant influenza vaccines (RIVs) administered IM.

We included studies that used placebo, no vaccination or no-influenza vaccine as comparator. We excluded cluster, crossover RCTs and studies that compared the same type of vaccine produced by different companies; we also excluded studies that analyzed the 2009 pandemic influenza vaccine, as the influenza manufacturing process and timeline are different.^9^ [Details of inclusion criteria are reported in the appendix (p 5)]

## Outcomes

The primary outcome was laboratory-confirmed influenza (i.e., influenza symptoms with a positive laboratory diagnosis). Secondary outcomes were: hospitalization, overall and influenza-related mortality, influenza-like illness (ILI), influenza-related pneumonia or lower respiratory-tract disease, systemic and local adverse events (AEs). For safety outcomes we measured the number of patients with at least one AE (systemic or local) in each study arm. When the number of participants with at least one systemic AE was not reported, we used as proxy measures for adults the number of participants with malaise as first choice, headache as second choice, and fever ≥ 37.5 °C as third choice; for children we used as proxy measures irritability as first choice, decreased activity/weakness as second choice, and fever ≥ 37.5 °C as third choice. When the number of participants with at least one local AE was not reported, we used as a proxy measure nasal congestion or rhinorrhea for intranasal vaccines; for intramuscular or intradermal vaccines, we used pain as first choice, local swelling/induration as second choice, and erythema/redness as third choice. For pregnant women, we examined both pregnancy outcomes (spontaneous abortion, fetal death, stillbirth, preterm birth < 37 weeks gestation) and neonatal outcomes (minor and major congenital malformations and neonatal death). For children, we examined otitis media and exacerbation of primary disease in those with pre-existing respiratory disease.

## Procedures

Two authors (SM, MGL) independently screened all titles and abstracts and assessed the full text for eligibility. Any doubt was resolved by discussion; in case of persisting disagreement, a third author (EP) acted as arbitrator. Two authors independently extracted the data (SG, GC) and assessed risk of bias (SM, SG) according to the criteria of the Cochrane Handbook for Systematic Reviews of Interventions.^10^ When outcome data were available only in graphs, we extrapolated them using web plot digitizer application (https://automeris.io/WebPlotDigitizer/).^11^ The intercoder reliability and validity of this software for estimating event rates from figures is high.^12^ In addition, to increase accuracy, as acceptable standards of practice for data extraction in systematic reviews, two independent authors (GC, SG) extracted data and reached consensus also for the data extracted from figures. Any doubt was resolved by discussion; in case of persisting disagreement, a third author (SM) acted as arbitrator.

## Data analysis

We conducted conventional pairwise random-effects meta-analyses for all dichotomous outcomes and comparisons when at least two studies were available. Fixed effects models were not used as a certain degree of heterogeneity was expected among and between the randomized studies.^13^ We calculated the risk ratio (RR) for each RCT, with the uncertainty in each result expressed with a 95% confidence interval (CI). Heterogeneity was analyzed by means of the I^2^ statistic (with I^2^values ≥ 60% considered as “substantial heterogeneity”) and the chi-square test (statistically significant for *P* value < 0.10).^14^

NMA was then conducted to estimate the effect size for all possible pairwise comparisons between vaccines, as well as to rank the efficacy and safety of the vaccines. The amount of heterogeneity was assumed to be equal across all treatment comparisons in the network.^15^ To rank vaccine efficacy with respect to each outcome, the Surface Under the Cumulative Ranking curve (SUCRA) and the mean ranks were used.^16^ We evaluated the transitivity assumption by visually comparing the distribution of clinical and methodological variables (e.g., age, risk of bias) that could act as effect modifiers across treatment comparisons. Incoherence (i.e., agreement between direct and indirect evidence) was evaluated for laboratory-confirmed influenza and systemic AEs using the design-by-treatment test^17^^,^^18^ and dividing the direct and indirect evidence.^19^ The random-effects model was fitted in a Bayesian framework using the RR as effect estimate and uncertainty expressed with a 95% credible interval (CrI).^16^^,^^19^, ^20^, ^21^

All analyses were performed using the R software environment, version 3.5.^22^ NMA was conducted with JAGS^23^ and the gemtc package for R.

We compared any vaccine with placebo or no vaccination, and then any other vaccine with trivalent inactivated vaccine as a common comparator, after which we compared each type of vaccine against each other. Network nodes were categorized as per licensed vaccines grouped by production method and vaccine characteristics (appendix, p 7), and are summarized in the appendix (Box 1, p 6.).

Separate analyses were performed for children (< 18 years) and adults and elderly (≥ 18years). Subgroup analyses were performed for children (0–5 years), the elderly (≥ 61 years), and participants with a comorbidity (i.e., cancer, immunocompromised state, and pre-existing respiratory diseases).

For brevity and consistency, we focus the present report on comparisons that included placebo and no vaccination or trivalent inactivated vaccine as comparators. The Netleague tables and forest plots present the comparisons in the following order: placebo, trivalent inactivated and recombinant, quadrivalent inactivated and recombinant, trivalent and quadrivalent live-attenuated vaccines.

### Role of the funding source

The funder of the study had no role in study design, data collection, data analysis, data interpretation, or writing of the report. The corresponding author had full access to all the data in the study and had final responsibility for the decision to submit for publication.

## Results

**Figure 1:**
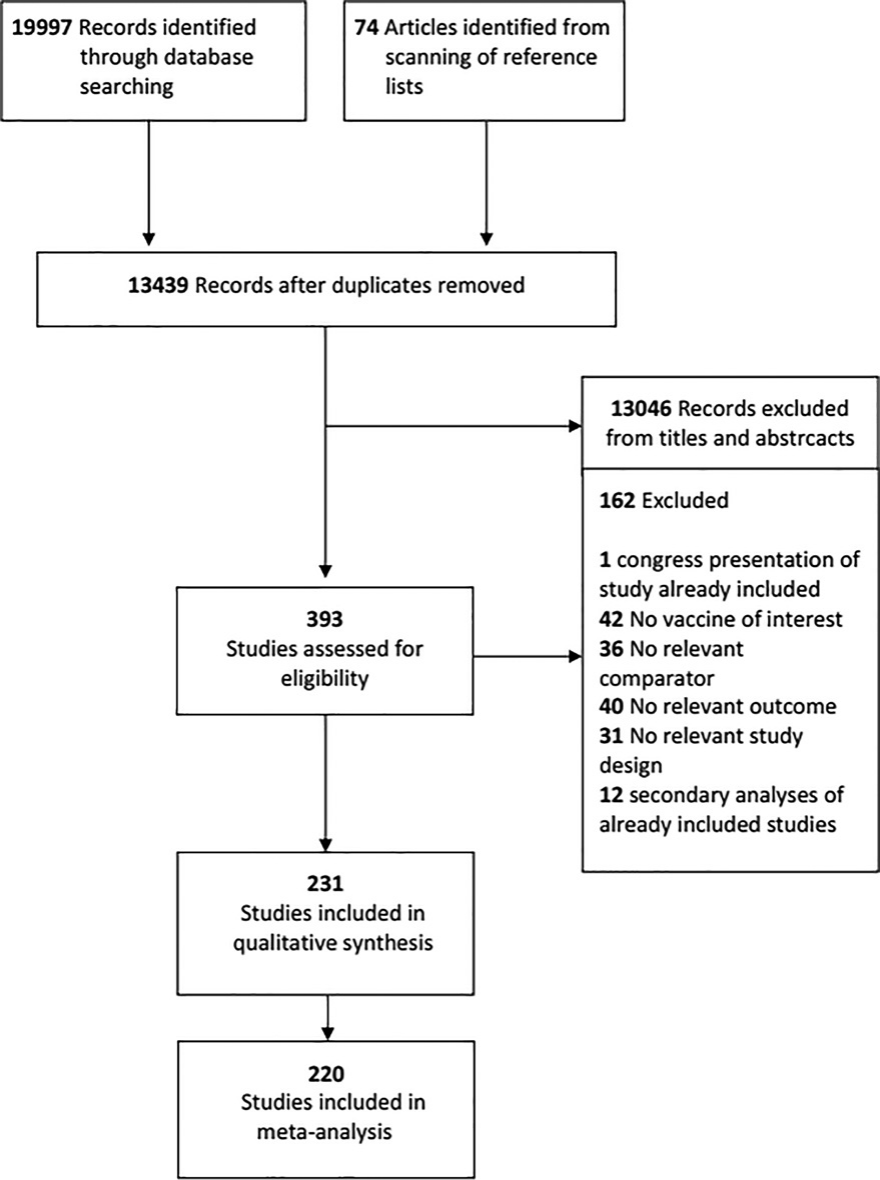
Study flow from literature search.

We identified 19,997 records through the database search and 74 articles from scanning the reference lists. After removing duplicates, 13,439 unique references remained, 13,046 of which were excluded because of title and abstract. We retrieved 393 full text studies for more detailed evaluation, 162 of which were excluded. References of excluded studies are reported in the appendix (pp 8–16). A total of 231 studies with 441,093 participants were included (appendix, pp 17–26): 220 studies (519 arms, including multi-arm and multi-cohort studies) with 429,804 participants (100,677 children and 329,127 adults and elderly) contributed to at least one NMA, whereas 11 studies did not provide useful quantitative data (Figure 1).

Out of the 220 studies, 161 arms involved children (age < 18 years), of which children aged ≤ 5 years accounted for 54.7% (*n* = 68,726); 358 arms involved adults and elderly, of which the elderly (≥ 61 years) accounted for 45.4% (*n* = 149,437). Twenty-six arms involved patients with chronic respiratory disease (*n* = 7250; 5471 children and 1779 adults and elderly) and 65 arms involved patients with cancer or immunocompromised status (*n* = 3865, 1141 children and 2724 adults and elderly). Sixteen arms (11,130 participants) included pregnant women. Furthermore, 210 studies (95.4%) were conducted in an outpatient setting; 88 included one or more placebo arms and five compared influenza vaccine with another vaccine. In addition, 93 (42.3%) studies were funded by the industry, 76 (34.5%) by government or private no profit agencies, while the remaining did not specify the funding source. Finally, 36.4% were conducted in North America, and 21.4% in Europe (Table 1). The appendix (pp 27–68) reports details of the characteristics of included studies.

**Table 1:** Characteristics of studies included in the review.

Study Characteristics	No. (%) of RCTs (*N* = 220)	
Year of publication		
1988–1995	9 (4.1)	
1996–2000	18 (8.2)	
2001–2005	24 (10.9)	
2006–2010	51 (23.2)	
2011–2015	56 (25.4)	
2016–2020	62 (28.2)	
Continent		
Europe	47 (21.4)	
Africa	10 (4.5)	
Asia	42 (19.1)	
North America	80 (36.4)	
South America	6 (2.7)	
Oceania	5 (2.3)	
Multi-continent	21 (9.5)	
Not reported	9 (4.1)	
Setting		
Outpatient	210 (95.4)	
Inpatient	5 (2.3)	
Long-term care facility	5 (2.3)	
Funding		
Industry	93 (42.3)	
Government/ private no profit	76 (34.5)	
Not reported	46 (20.9)	
Industry and private no profit	5 (2.3)	

	No. of participants	No. (%) of compared arms (*N* = 519) *
Type of vaccine		
placebo/no vaccine/other vaccine	75,511	112 (21.6)
3-IIV trivalent inactivated	153,885	192 (37.0)
3-IIV HD trivalent inactivated high-dose	30,101	21 (4.1)
3-IIV ID trivalent inactivated intradermal	15,197	33 (6.3)
3-IIV MF59/AS03-adj trivalent inactivated adjuvanted MF59/AS03	36,310	33 (6.3)
3-IIV vir/lip-adj trivalent inactivated adjuvanted virosome/liposome	1871	14 (2.7)
3-RIV trivalent inactivated recombinant	3708	7 (1.3)
4-IIV quadrivalent inactivated	52,599	47 (9.1)
4-IIV HD quadrivalent inactivated high-dose	1807	2 (0.4)
4-IIV ID quadrivalent inactivated intradermal	1672	1 (0.2)
4-IIV MF59/AS03-adj quadrivalent inactivated adjuvanted MF59/AS03	994	2 (0.4)
4-RIV quadrivalent inactivated recombinant	17,455	7 (1.3)
3-LAIV trivalent live-attenuated	36,491	45 (8.7)
4-LAIV quadrivalent live-attenuated	2203	3 (0.6)
Age (years)		
≤5	68,726	88 (17.0)
6–17	12,512	37 (7.1)
mixed aged children (<18)	19,439	36 (6.9)
18 - 60	156,642	212 (40.9)
≥61	149,437	109 (21.0)
adult and elderly (³18)	23,048	37 (7.1)
Comorbidity		
none (i.e. healthy participants)	341,853	362 (69.7)
chronic respiratory disease	7250	28 (5.4)
multimorbidity	65,071	45 (8.7)
immunodepression (any cause)/cancer	3865	65 (12.5)
pregnant women	10,936	14 (2.7)
pregnant women/ immunodepression	194	2 (0.4)
not reported	635	3 (0.6)

⁎ the overall number of arms includes multi-arms and multi-cohort studies.

The results of pairwise meta-analyses are presented in the appendix (pp 77–86). Five vaccines (3-IIV, 3-RIV, 4-IIV, 3-LAIV, 3-IIV ID) had at least one placebo- or no vaccination-controlled trial. We use the term “placebo-controlled” to mean both placebo, no vaccination or other vaccine than the influenza one. The vaccines 3-LAIV, 3-IIV ID, 3-RIV, 3-IIV HD, 3-IIV MF59/AS03-adj, 3-IIV vir/lip-adj and 4-IIV, were compared directly with 3-IIV. For laboratory-confirmed influenza and any systemic AE, we found no evidence of incoherence between direct and indirect evidence (appendix, pp 87–94).

**Figure 2:**
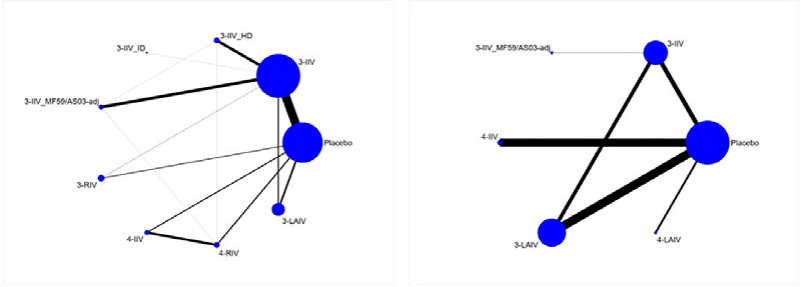
Network Geometry of laboratory-confirmed influenza Panel a: adults and elderly; Panel b: children. The thickness of the line is proportional to the precision of each direct estimate, and the width of each circle is proportional to the number of studies included in the treatment. Placebo: placebo/no vaccine; 3-IIV: trivalent inactivated intramuscular; 3-IIV HD: trivalent inactivated high dose intramuscular; 3-IIV ID: trivalent inactivated intradermal; 3-IIV MF59/AS03-adj: trivalent inactivated adjuvanted with MF59/ASO3 intramuscular: 3-RIV: trivalent recombinant intramuscular; 4-IIV: quadrivalent inactivated intramuscular; 4-RIV: quadrivalent recombinant intramuscular; 3-LAIV: trivalent live attenuated intranasal; 4-LAIV: quadrivalent live attenuated intranasal.

The NMA for the primary outcome of laboratory-confirmed influenza in adults and the elderly included 40 RCTs (209,095 participants) with eight different types of vaccines (Figure 2). All comparisons except one (3-IIV ID) showed a significant difference compared against placebo, with RR between 0.33 (95% CrI 0.21–0.55) for 3-IIV HD and 0.56 (95% CrI 0.41–0.74) for 3-LAIV; little and non-significant differences were observed on comparison between different vaccines. Compared with 3-IIV, the differences were again small and not significant, except for 3-LAIV, which was less efficacious [RR 1.41 (95% CrI 1.04–1.88)] (Table 2; appendix, p 95). Ranking of vaccines based on SUCRAs are presented in the appendix (p 144).

**Table 2:** Netleague laboratory-confirmed influenza: adults and elderly.

placebo	0.4 (0.34, 0.46)	0.33 (0.21, 0.55)	0.4 (0.04, 3.89)	0.37 (0.22, 0.64)	0.39 (0.23, 0.61)	0.56 (0.36, 0.83)	0.47 (0.29, 0.7)	0.56 (0.41, 0.74)
2.51 (2.18, 2.95)	3-IIV	0.83 (0.53, 1.37)	1 (0.11, 9.75)	0.94 (0.57, 1.59)	0.97 (0.59, 1.54)	1.41 (0.9, 2.15)	1.18 (0.73, 1.81)	1.41 (1.04, 1.88)
3.01 (1.82, 4.86)	1.2 (0.73, 1.88)	3-IIV HD	1.19 (0.12, 12.08)	1.13 (0.57, 2.18)	1.16 (0.57, 2.19)	1.68 (0.87, 3.07)	1.41 (0.71, 2.56)	1.68 (0.94, 2.85)
2.53 (0.26, 24.11)	1 (0.1, 9.52)	0.84 (0.08, 8.49)	3-IIV ID	0.95 (0.09, 9.45)	0.97 (0.1, 9.55)	1.41 (0.14, 13.77)	1.17 (0.12, 11.47)	1.41 (0.14, 13.68)
2.67 (1.57, 4.5)	1.06 (0.63, 1.74)	0.89 (0.46, 1.75)	1.06 (0.11, 10.86)	3-IIV MF59/AS03-adj	1.03 (0.49, 2.01)	1.49 (0.76, 2.8)	1.25 (0.62, 2.33)	1.5 (0.82, 2.63)
2.59 (1.65, 4.29)	1.03 (0.65, 1.7)	0.86 (0.46, 1.76)	1.03 (0.1, 10.51)	0.97 (0.5, 2.02)	3-RIV	1.44 (0.79, 2.74)	1.21 (0.64, 2.29)	1.45 (0.86, 2.54)
1.79 (1.21, 2.76)	0.71 (0.47, 1.11)	0.59 (0.33, 1.15)	0.71 (0.07, 7.15)	0.67 (0.36, 1.32)	0.69 (0.37, 1.27)	4-IIV	0.84 (0.56, 1.21)	1 (0.61, 1.66)
2.13 (1.42, 3.44)	0.85 (0.55, 1.38)	0.71 (0.39, 1.4)	0.85 (0.09, 8.65)	0.8 (0.43, 1.61)	0.83 (0.44, 1.55)	1.2 (0.83, 1.78)	4-RIV	1.2 (0.72, 2.05)
1.79 (1.35, 2.43)	0.71 (0.53, 0.96)	0.59 (0.35, 1.06)	0.71 (0.07, 7.02)	0.67 (0.38, 1.23)	0.69 (0.39, 1.17)	1 (0.6, 1.64)	0.84 (0.49, 1.38)	3-LAIV

The estimate is located at the intersection of the column-defining vaccine and the row-defining vaccine. Data are RRs (95% CrI). Significant results are in bold.

In the upper triangle, comparison of treatments should be read from right to left. An RR below 1 favors the medication on the bottom right vs. the medication on the top left in the diagonal. E.g., RR 0.40 (95% CrI 0.34–0.46) indicates a significant reduction in the incidence of laboratory-confirmed influenza for the trivalent inactivated vaccine (3-IIV) compared with placebo or no vaccination.

In the bottom triangle, comparison of treatments should be read from left to right. An RR below 1 favors the medication on the top left vs. the medication on the bottom right in the diagonal. E.g., RR 0.84 (95% CrI 0.08–8.49) indicates a non-significant reduction in the incidence of laboratory-confirmed influenza for the trivalent inactivated high-dose vaccine (3-IIV-HD) compared with the trivalent inactivated intradermal vaccine (3-IIV ID).

Abbreviations: Placebo: placebo or no vaccination; 3-IIV: trivalent inactivated intramuscular; 3-IIV HD: trivalent inactivated high-dose intramuscular; 3-IIV ID: trivalent inactivated intradermal; 3-IIV MF59/AS03-adj: trivalent inactivated adjuvanted with MF59/AS03 intramuscular; 3-RIV: trivalent recombinant intramuscular; 4-IIV: quadrivalent inactivated intramuscular; 4-RIV: quadrivalent recombinant intramuscular; 3-LAIV: trivalent live-attenuated intranasal.

In the elderly subgroup (age ≥ 61 years) (12 RCTs, 107,265 participants, seven vaccines in the network) only 4-RIV showed a significant effect on laboratory-confirmed influenza against placebo [RR 0.3 (95% CrI 0.06–0.97)], although point estimates favored all vaccines over placebo, with RR between 0.4 (95% CrI 0.11–1.14) for 4-IIV, 0.4 (95% CrI 0.07–2.00) for 3-RIV and 0.63 (95% CrI 0.27–1.44) for 3-LAIV (appendix, p 96). In the subgroup of immunocompromised/cancer patients (6 RCTs, 1276 participants, 4 vaccines) only 3-IIV appeared better than placebo [RR 0.19 (95% CrI 0.03–0.94)] (appendix, p 98). Because of paucity of data, sub group analysis of participants with pre-existing respiratory diseases was not performed.

The NMA for laboratory-confirmed influenza in children included 24 RCTs (60,502 participants) with 5 vaccines (Figure 2). 3-IIV, 3-LAIV and 3-IIV MF59/AS03-adj showed significant difference compared against placebo with RR of 0.55 (95% CrI 0.36–0.83), 0.28 (95% CrI 0.19–0.41) and 0.13 (95% CrI 0.03–0.51) respectively, while 4-IIV and 4-LAIV showed no difference. Comparison of the vaccines with each other showed that 3-LAIV and 3-IIV MF59/AS03-adj were more efficacious than 3-IIV with RR of 0.52 (95% CrI 0.32–0.82) and 0.23 (95% CrI 0.06–0.87), respectively (Table 3; appendix, p 101). Ranking of vaccines based on SUCRAs are summarized in the appendix (p 144).

**Table 3:** Netleague laboratory-confirmed influenza: children.

placebo	0.55 (0.36, 0.83)	0.13 (0.03, 0.51)	0.5 (0.21, 1.19)	0.28 (0.19, 0.41)	0.71 (0.21, 2.4)
1.81 (1.2, 2.77)	3-IIV	0.23 (0.06, 0.87)	0.9 (0.35, 2.4)	0.52 (0.32, 0.82)	1.28 (0.35, 4.7)
7.96 (1.98, 32.92)	4.4 (1.15, 17.03)	3-IIV MF59/AS03-adj	3.97 (0.77, 21.01)	2.27 (0.54, 9.38)	5.64 (0.88, 36.6)
2 (0.84, 4.76)	1.11 (0.42, 2.88)	0.25 (0.05, 1.3)	4-IIV	0.57 (0.22, 1.44)	1.42 (0.32, 6.38)
3.51 (2.46, 5.17)	1.94 (1.22, 3.15)	0.44 (0.11, 1.85)	1.76 (0.7, 4.6)	3-LAIV	2.49 (0.7, 9.08)
1.41 (0.42, 4.81)	0.78 (0.21, 2.82)	0.18 (0.03, 1.13)	0.7 (0.16, 3.15)	0.4 (0.11, 1.42)	4-LAIV

The estimate is located at the intersection of the column-defining vaccine and the row-defining vaccine. Data are RRs (95% CrI). Significant results are in bold.

In the upper triangle, comparison of treatments should be read from right to left. An RR below 1 favors the medication on the bottom right vs. the medication on the top left in the diagonal. E.g., RR 0.55 (95% CrI 0.36–0.83) indicates a significant reduction in the incidence of laboratory-confirmed influenza for the trivalent inactivated vaccine (3-IIV) compared with placebo/ no vaccination.

In the bottom triangle, comparison of treatments should be read from left to right. An RR below 1 favors the medication on the top left vs. the medication on the bottom right in the diagonal. E.g., RR 0.25 (95% CrI 0.05–1.3) indicates a non-significant reduction in the incidence of laboratory-confirmed for the trivalent inactivated adjuvanted with MF59/AS03 intramuscular vaccine (3-IIV MF59/AS03-adj) compared with the quadrivalent inactivated intramuscular (4-IIV).

Abbreviations: Placebo: placebo/ no vaccination; 3-IIV: trivalent inactivated intramuscular; 3-IIV MF59/AS03-adj: trivalent inactivated adjuvanted with MF59/AS03 intramuscular; 4-IIV: quadrivalent inactivated intramuscular; 3-LAIV: trivalent live-attenuated intranasal; 4-LAIV: quadrivalent live-attenuated intranasal.

In the subgroup analysis of children aged ≤ 5 years, the 3-LAIV and the 3-IIV MF59/AS03-adj vaccines were more efficacious than placebo (19 RCTs, 53,973 participants, 4 vaccines in the network), being RR 0.30 (95% CrI 0.18–0.46) and 0.14 (95% CrI 0.03–0.68), respectively (appendix, p 101). In children with pre-existing respiratory diseases, only the 3-LAIV vaccine was more efficacious than placebo (RR 0.09, 95% CrI 0.00–0.54; 5 RCTs, 5801 participants, 3 vaccines) (appendix, p 103). Due to paucity of data, subgroup analyses of immunocompromised children and children with cancer were not performed.

The NMA for hospitalization in all adults and elderly showed that all vaccines but 4-IIV and 3-LAIV reduced the hospitalization rate compared against placebo (21 studies, 59,193 participants, 7 vaccines in the network), with RR between 0 (95% CrI 0–0.1) and 0.29 (95% CrI 0.14–0.52) (appendix, p 105). Among children, both 3-IIV vir/lip-adj and 4-RIV reduced hospitalization rate (13 studies, 50,249 participants, 5 vaccines in the network), RR 0.16 (95% CrI 0.04–0.58) and RR 0.0 (95% CrI 0.0–0.1), respectively, versus placebo, and RR 0.17 (95% CrI 0.04–0.8) and RR 0.0 (95% CrI 0.0–1.11), respectively, versus 3-IIV (appendix, p 107).

In adults and the elderly (31 RCTs, 174,705 participants, 10 vaccines in the network), all but two vaccines 3-LAIV and 4-IIV-HD) were associated with a significant reduction in mortality compared against placebo with RR between 0.0 (95% CrI 0.0–0.3 and 0.37 (95% CrI 0.16–0.78) (appendix, p 110). In children (15 studies, 42,834 participants, 6 vaccines in the network), 3-IIV MF59/AS03-adj, 4-RIV and 4-LAIV were associated with a reduction in mortality with RR of 0.04 (95% CrI 0.0–0.96), 0.0 (95% CrI 0.0–0.4) and 0.0 (95% CrI 0.0–0.43). However, the data were sparse and the results largely imprecise (appendix, p 112).

In adults and the elderly, only 3-IIV was shown to be efficacious in reducing influenza-like illness (ILI) compared with placebo (30 RCTs, 83,537 participants, 7 vaccines in the network), RR 0.8 (95% CrI 0.72–0.89) (appendix, p 114). In children, both 3-IIV and 3-LAIV were efficacious in reducing ILI compared against placebo (16 RCTs, 37,165 participants, 4 vaccines in the network), with RR 0.59 (95% CrI 0.42–0.80) and 0.64 (95% CrI 0.44–0.84), respectively (appendix, p 116).

None of the vaccines was found to be more efficacious than placebo (12 RCTs, 22,690 children, 5 vaccines in the network) in reducing the incidence of acute otitis media in children (appendix, p 118).

Six RCTs (11,130 participants) included pregnant women. Four studies compared 3-IIV against placebo/no intervention or other non-influenza vaccines, one compared 3-LAIV versus 3-IIV, and one 4-IIV versus 3-IIV. No significant differences were found for any pregnancy or neonatal outcome (appendix, p 120).

Due to paucity of data, we were unable to perform analyses for influenza-related pneumonia/lower respiratory diseases and influenza-related mortality.

The NMA for systemic AEs in adults and elderly included 121 RCTs (220,595 participants, 13 vaccines). Compared against placebo, after vaccination with 3-IIV, 3-IIV HD, 3-IIV ID, 3-IIV MF59/AS03-adj, 4-IIV, 4-IIV MF59/AS03-adj, 4-RIV, 3-LAIV or 4-LAIV, more participants reported at least one systemic AE, with a RR between 1.5 (95% CrI 1.18–1.89) for 4-IIV MF59/AS03-adj and 1.15 (95% CrI 1.06–1.23) for 3-IIV. Compared with 3-IIV, only the 3-IIV HD, the 3-IIV MF59/AS03-adj and the 4-IIV MF59/AS03-adj vaccines were associated with at least one systemic AE in significantly more participants, with a RR of 1.16 (95% CrI 1.04–1.29), 1.2 (95% CrI1.09–1.32) and 1.31 (95% CrI 1.05–1.63), respectively (appendix, p 122). The appendix (p 144) presents the vaccine ranking based on SUCRAs.

In the subgroup analysis of the elderly (age ≥ 61 years) (44 RCTs, 107,701 participants, 11 vaccines), none of the vaccines showed significant differences compared against placebo. In comparison with 3-IIV, the subgroup analysis of elderly participants yielded results similar to the overall analysis, with the 3-IIV HD and 3-IIV MF59/AS03-adj vaccines associated with a higher percentage of participants reporting at least one systemic AE (appendix, p 125). In the subgroup with immunocompromised or cancer patients (19 RCTs, 2212 participants, 8 vaccines) 3-RIV was associated with less systemic AEs compared against placebo and 3-IIV, but data are sparse and non-informative (appendix, p 128).

In children, the NMA of any systemic AEs included 59 RCTs (77,208 participants, 9 vaccines). None of the vaccines were associated with a significantly higher percentage of children reporting at least one systemic AE compared against placebo. Compared with 3-IIV, only 3-IIV MF59/AS03-adj had a higher percentage of children reporting at least one systemic AE (RR 1.23, 95% CrI 1.02–1.49) (appendix, p 131).

In children aged ≤ 5 years, (33 RCTs, 54,146 participants, 7 vaccines) only 3-LAIV was associated with a higher percentage of children reporting at least one systemic AE compared against placebo with RR of 1.17 (95% CrI 1.0–1.37). Compared against 3-IIV, only 3-IIV MF59/AS03-adj was associated with more AEs with a RR of 1.24 (95% CrI 1.0–1.54) (appendix, p 134). Due to paucity of data, we were unable to perform subgroup analyses in immunocompromised children and children with cancer.

The NMA for any local AEs in adults and the elderly included 123 RCTs (223,093 participants, 13 vaccines). All vaccines showed higher frequency of participants reporting at least one local AE compared against placebo, with RR from 4.47 (95% CrI 1.96–10.15) for 4-IIV ID to 2.23 (95% CrI 1.18–4.17) for 4-LAIV. In comparison with 3-IIV, only 3-IIV HD, 3-IIV ID, and 3-IIV MF59/AS03-adj were associated with more participants reporting local AEs (appendix, p 137).

The NMA for any local AE in children included 55 RCTs (64,004 participants, 9 vaccines). Only 3-LAIV showed significantly more children reporting at least one local AE compared with placebo (RR 1.22, 95% CrI 1.08–1.38) and 3-IIV (RR 1.25, 95% CrI 1.01–1.55) (appendix, p 140).

The appendix (p 145) reports the outcomes and subgroup analyses that were planned but not conducted because of insufficient data.

More than a half (57.6%) of the studies had low risk of bias for random sequence generation, only one study had high risk and the remaining (41.9%) had an unclear risk. Allocation concealment was judged at low risk in 39.8% of studies, unclear in 57.7%, and at high risk in 2.6%. Furthermore, 45.1% were judged at low risk for performance bias, 11.2% at unclear risk, and 43.7% at high risk; 54.6% were judged at low risk of detection bias, 18.6% at unclear risk, and 26.8% at high risk. The majority of studies were at low risk of attrition bias (87%) and selective reporting bias (93.0%) (appendix, pp 69–76).

## Discussion

Our NMA quantifies the progress achieved in the prevention of influenza with different types of vaccines over the last 30 years. Based on the cumulative data from 220 clinical trials of influenza vaccines involving 429,804 children, adults, and elderly people, we found that, in adults and elderly, all influenza vaccines, except for the trivalent inactivated intradermal vaccine, conferred protection against laboratory-confirmed influenza compared against placebo or no vaccination. Cumulative results for benefits such as reduction in mortality or hospitalization are less certain: while some vaccines reduced the relative risk of hospitalization or death by one half or more, the CIs around effect estimates were imprecise, limiting our confidence. In adults and the elderly, evidence for newer quadrivalent vaccines is rapidly cumulating achieving benefits similar to well-established trivalent inactivated vaccine, which is probably the most common in the world. In children, trivalent live-attenuated vaccines were significantly superior to trivalent inactivated vaccines against laboratory-confirmed influenza, with the exception of MF59-adjuvanted vaccines which seem more effective but also associated with more AEs.

In the elderly subgroup when compared to placebo vaccines protection against laboratory-confirmed influenza is less pronounced, with only the quadrivalent recombinant intramuscular vaccine reaching a statistically significant result. This discrepancy between age groups might reflect a heterogeneous immunological response, a higher susceptibility to disease, or some heterogeneity among studies. A Cochrane review addressing the role of influenza vaccination in the elderly concluded that there is low-certainty evidence of efficacy of influenza vaccines.^24^ Observational studies found that MF59-adjuvanted vaccines are more efficacious than unadjuvanted vaccines in the elderly.^25^ Moreover, less data have been collected on newer vaccines involving the very elderly (85+). Key target populations of vaccine campaigns for which there is at present little or no data should be the focus of future investigations so that more direct evidence about the relative merits of these vaccines can be obtained. However, these comparisons should privilege head-to-head vaccine comparisons.

In terms of safety, RCTs provided a wealth of indicative data. In adults and the elderly, all vaccines but trivalent inactivated adjuvanted with virosome/liposome, trivalent recombinant, quadrivalent inactivated high dose and intradermal caused significantly more systemic AEs than placebo. The largest differences were limited to frequent but not clinically relevant reactions. In children, only the trivalent live-attenuated vaccine was associated with more systemic and local AEs than placebo. When compared with trivalent inactivated vaccine, only the 3-IIV MF59/AS03-adj was associated with significantly more children reporting at least one systemic AE.

An overview of systematic reviews concluded that most seasonal influenza vaccines show statistically significant efficacy, with an overlapping degree of magnitude for laboratory-confirmed influenza cases.^26^ This and our review are comprehensive for the number of studies and range of outcomes covered, the study population (not restricted to any particular population or clinical setting), and the sophisticated statistical analyses (including consistency tests of direct and indirect evidence). Still, there might be clinically important differences among influenza vaccines that have not emerged so far. Given the modest rates of infection in low-incidence seasons, and the occurrence of complete or partial antigenic mismatch between circulating viruses and vaccine strains, a definitive assessment of the merits of one vaccine over another would require very large pragmatic RCTs. Differences in vaccine performance with different vaccines might be assessed by means of serum antibodies analyses However, seroconversion and seroprotection may not represent a valid surrogate of clinical protection, particularly in frail patients (e.g., the elderly).^27^

Our review has some limitations. First, analyses were limited by the amount of data in the studies. Although patient-relevant outcomes were included, not all studies reported them, and most had few events, especially with regard to key events such as pneumonia and influenza-related mortality. Trials were of short duration. Some vaccines based on virosomes were included in our network but are no longer available on the market^28^; pediatric influenza vaccine adjuvanted with MF59 was included but it has not been authorized for use in children in most countries.^29^^,^^30^ Since we analyzed only average treatment effects and did not investigate potentially important clinical and demographical modifiers of vaccination response at the individual patient level (e.g., age, sex, immune status), our subgroup analyses might have an aggregation bias. We were unable to assess variation in vaccine efficacy by influenza type/subtype, circulating strains and degree of match as only few studies reported type/subtype-specific estimates together with an overall efficacy estimate and provided data on individual years, a precondition to explore the effect of match/mismatch. This modifier could also have impact on the meta-analysis transitivity assumption. Finally, we did not perform a formal cost-effectiveness analysis.

In conclusion, influenza vaccines were associated with a lower risk of laboratory-confirmed influenza compared with placebo or no vaccination. Across the head-to-head comparisons, there were no significant differences in the associations between any of the vaccines considered here and the risk of influenza among adults and the elderly. Short-term safety outcomes were more heterogenous. In children, the trivalent live-attenuated vaccine was more efficacious in preventing laboratory-confirmed influenza than the trivalent inactivated vaccine, though it showed a greater number of minor AEs. In adults and the elderly, the choice of vaccine may depend on patient and caregiver values and preferences or costs and feasibility. The variable mismatch between vaccine strains and circulating viruses is an important confounder that we could not control. This and other uncertainties that might result from different treatment settings, temper any strong conclusions that can be drawn from the present findings. Nonetheless, we believe that health systems can exploit the prevention benefits conferred by similarly effective vaccines in large vaccination programs, selecting products based on price, safety and local factors (e.g., national production).

## Declaration of interests

EP declares support for attending meeting and travel by Sanofi and Seqirus and, participation in Advisory Board of Sanofi. CG declares participation in Advisory Board of Sanofi. SM, TL, SG, MGL, GC, DC, SB and LM declare no competing interests.

## Appendix Supplementary materials

Image, application 1
