# Supplementary material for: Comparative efficacy and safety of vaccines to prevent seasonal influenza: A systematic review and network meta-analysis
Source: eClinicalMedicine. 2022 Mar 25;46:101331. doi: 10.1016/j.eclinm.2022.101331 (PMC8961170; doi:10.1016/j.eclinm.2022.101331)
Supplement: Supplementary file 1 [file mmc1.pdf]

Comparative efficacy and safety of vaccines to prevent seasonal influenza: a systematic review and network meta-analysis

Silvia Minozzi, Theodore Lytras, Silvia Gianola, Marien Gonzalez-Lorenzo, Greta Castellini, Cristina Galli, Danilo Cereda, Stefanos Bonovas, Elena Pariani, Lorenzo Moja

Supplementary Appendix

Contents

Search strategy 3
Inclusion criteria details 5
Box 1. Summary of the different types of seasonal influenza vaccines available 6
Nodes in the network 7
References of excluded studies 8
References of included studies 17
Characteristics of included studies 27
Cochrane risk of bias results across studies 69
Cochrane risk of bias in individual studies 70
Pairwise meta-analyses 77
Adults and the elderly: Inconsistency in networks of laboratory- confirmed influenza and any systemic adverse event outcomes 87
All children: Inconsistency in networks of laboratory- confirmed influenza and any systemic adverse event outcomes 94
Adults and the elderly: Forest plots of laboratory- confirmed influenza, comparison with placebo/no vaccine and with 3-IIV 95
The elderly (≥61 years): Network geometry of laboratory -confirmed influenza 96
The elderly (≥61 years): Forest plots lof aboratory- confirmed influenza, comparison with placebo-no vaccine and with 3-IIV 96
The elderly (≥61 years): Netleague analysis of laboratory- confirmed influenza 97
Immunocompromised/cancer patients: Network geometry of laboratory -confirmed influenza 98
Immunocompromised/cancer patients: Forest plots lof aboratory- confirmed influenza, comparison with placebo-no vaccine and with 3-IIV 98
Immunocompromised/cancer patients: Netleague analysis of laboratory- confirmed influenza 99
All children: Forest plots of laboratory confirmed influenza, comparison with placebo/no vaccine and with 3-IIV 100
Children, aged ≤ 5 years: Network geometry of laboratory confirmed influenza 101
Children, aged ≤ 5 years: Forest plots of laboratory confirmed influenza, comparison with placebo/no vaccine and with 3-IIV 101
Children, aged ≤ 5 years: Netleague analysis of laboratory confirmed influenza 102
Children with pre-existing respiratory diseases: Network geometry of laboratory- confirmed influenza 103
Children with pre-existing respiratory diseases: Forest plots of laboratory confirmed influenza, comparison with placebo-no vaccine and with 3-IIV 103
Children with pre-existing respiratory diseases: Netleague analysis of laboratory- confirmed influenza 104
Adults and the elderly: Network geometry of hospitalization rate 105
Adults and the elderly: Forest plots of hosptitalization rate, comparison with placebo-no vaccine and with 3-IIV 105
Adults and the eldelry: Netleague analysis of hospitalization rate 106
All children: Network geometry of hospitalization rate 107
All children: Forest plots of hospitalization rate, comparison with placebo/no vaccine and with 3-IIV 107
All children. Netleague analysis of hospitalization rate 108
Adults and the elderly: Network geometry of mortality of any cause 109
Adults and the elderly: Forest plots of mortality of any cause, comparison with placebo/no vaccine and with 3-IIV 110
Adults and the elderly: Netleague analysis of mortality of any cause 111
All children: Network geometry of mortality of any cause 112
All children: Forest plots of mortality of any cause, comparison with placebo/no vaccine and with 3-IIV 112
All children: Netleague analysis of mortality of any cause 113
Adults and the elderly: Network geometry of Influenza like Illness (ILI) 114

Adults and the elderly: Forest plot of ILI, comparison with placebo/no vaccine and with 3-IIV 114
Adults and the elderly: Netleague analysis of ILI 115
All children: Network geometry of ILI 116
All children: Forest plots of ILI. Comparison with placebo/no vaccine and with 3-IIV 116
All children: Netleague analysis of ILI 117
All children: Network geometry of acute otitis media 118
All children: Forest plot of acute otitis media, comparison with placebo/no vaccine and with 3-IIV 118
All children: Netleague analysis of acute otitis media 119
Pregnancy outcomes: 3-IIV vs placebo/no vaccine, pairwise meta-analyses 120
Adults and the elderly: Network geometry of any systemic adverse event 121
Adults and the elderly: Forest plots of any systemic adverse event, comparison with placebo/no vaccine and with 3-IIV 122
Adults and the elderly: Netleague analysis of any systemic adverse event 123
The elderly, ≥61 years: Network geometry of any systemic adverse event 124
The elderly, ≥61 years: Forest plot of any systemic adverse event, comparison with placebo/no vaccine and with 3-IIV 125
The elderly, ≥61 years: Netleague analysis of any systemic adverse event 126
Adult and the elderly, immunocompromised-cancer participants: Network geometry of any systemic adverse event 127
Adult and the elderly, immunocompromised-cancer participants: Forest plots of any systemic adverse event, comparison with placebo/no vaccine and with 3-IIV 128
Adult and rhe elderly, immunocompromised-cancer participants: Netleague analysis of any systemic adverse event 129
All children: Network geometry of any systemic adverse event 130
All children: Forest plots of any systemic adverse event, comparison with placebo/no vaccine and with 3-IIV 131
All children: Netleague analysis of any systemic adverse event 132
Children, aged ≤5 years: Network geometry of any systemic adverse event 133
Children, aged ≤5 years: Forest plots of any systemic adverse event, comparison with placebo/no vaccine and with 3-IIV 134
Children, aged ≤5 years: Netleague analysis of any systemic adverse event 135
Adults and the elderly: Network geometry of any local adverse event 136
Adults and the elderly: Forest plots of any local adverse event, comparison with placebo/no vaccine and with 3-IIV 137
Adults and the elderly: Netleague analysis of any local adverse event 138
All children: Network geometry of any local adverse event 139
All children: Forest plots of any local adverse event, comparison with placebo/no vaccine and with 3-IIV 140
All children: Netleague analysis of any local adverse event 141
Children with pre-existing respiratory diseases: Network geometry of exacerbation chronic respiratory disease 142
Children with pre-existing respiratory diseases: Forest plots of exacerbation chronic respiratory disease, comparison with placebo-no vaccine and with 3-IIV* 142
Children with pre-existing respiratory diseases: Netleague analysis of exacerbation chronic respiratory disease 143
SUCRA Adults and the elderly 144
SUCRA probability any Children 144
Analyses of outcomes and subgroups not performed because of paucity of data 145

Search strategy

MEDLINE (PUBMED)
#1 “Influenza, Human”[MeSH]
#2 “Influenzavirus A”[MeSH]
#3 “Influenzavirus B”[MeSH]
#4 influenza*[Text Word] OR flu[Text Word]
#5 #1 OR #2 OR #3 OR #4
#6 “Vaccines”[MeSH]
#7 “Immunization”[MeSH]
#8 (vaccin*[Text Word] OR immuni*[Text Word] OR inocula*[Text Word])
#9 #6 OR #7 OR #8
#10 #5 AND #9
#11 “Influenza Vaccines”[MeSH]
#12 #10 OR #11
#13 “Randomized Controlled Trial” [Publication Type]
#14 “Controlled Clinical Trial” [Publication Type]
#15 randomized[Title/Abstract]
#16 placebo[Title/Abstract]
#17 “drug therapy” [Subheading]
#18 randomly[Title/Abstract]
#19 trial[Title/Abstract]
#20 groups[Title/Abstract]
#21 #13 OR #14 OR #15 OR #16 OR #17 OR #18 OR #19 OR #20
#22 (“Animals”[MeSH]) NOT “Humans”[MeSH]
#23 #21 NOT #22
#24 #12 AND #23

EMBASE
#1 ’influenza vaccine’/de
#2 ’influenza’/exp
#3 ’influenza virus a’/exp OR ’influenza virus b’/exp
#4 flu:ab,ti OR influenza*:ab,ti
#5 #2 OR #3 OR #4
#6 ’vaccine’/de OR ’acellular vaccine’/de OR ’dna vaccine’/de OR ’inactivated vaccine’/de OR ’live vaccine’/de OR ’subunit vaccine’/
de OR ’virus vaccine’/de OR ’virosome vaccine’/de OR ’recombinant vaccine’/de
#7 ’immunization’/de OR ’vaccination’/de OR ’active immunization’/de OR ’immunoprophylaxis’/de OR ’mass immunization’/de
#8 vaccin*:ab,ti OR immuni*:ab,ti OR inocul*:ab,ti
#9 #6 OR #7 OR #8
#10 #5 AND #9
#11 #1 OR #10
#12 ’randomized controlled trial’/exp OR ’single blind procedure’/exp OR ’double blind procedure’/exp
#13 random*:ab,ti OR placebo*:ab,ti OR factorial*:ab,ti OR assign*:ab,ti OR allocat*:ab,ti OR volunteer*:ab,ti OR ((singl* OR doubl*) NEAR/3 (blind* OR mask*)):ab,ti
#14 #12 OR #13
#15 #11 AND #14
[1-1-2017]/sd NOT [14-2-2018]/sd AND [embase]/lim

Cochrane central (Trials)
#1 MeSH descriptor: [Influenza, Human] explode all trees
#2 MeSH descriptor: [Influenzavirus A] explode all trees
#3 MeSH descriptor: [Influenzavirus B] explode all trees
#4 influenza* or flu
#5 #1 or #2 or #3 or #4
#6 MeSH descriptor: [Vaccines] explode all trees
#7 MeSH descriptor: [Immunization] explode all trees
#8 vaccin* or immuni* or inocula*
#9 #6 or #7 or #8
#10 #5 and #9
#11 MeSH descriptor: [Influenza Vaccines] explode all trees

#12	#10 or #11
#13	MeSH descriptor: [Randomized Controlled Trial] explode all trees
#14	MeSH descriptor: [Controlled Clinical Trial] explode all trees
#15	randomized
#16	placebo
#17	MeSH descriptor: [Drug Therapy] explode all trees
#18	randomly
#19	trial
#20	groups
#21	#13 or #14 or #15 or #16 or #17 or #18 or #19 or #20
#22	#12 and #21

Inclusion criteria details

We included only vaccines and doses licensed by regulatory authorities, namely

-Standard dose Inactivated vaccines, intramuscular: adults and elderly: virion, sub unit or split trivalent or quadrivalent vaccine containing 15 µg di HA per strains (TIV: 45 µg total, QIV: 60 µg total); children 6-36 months: 7,5 µg/strain; if never vaccinated before, two doses one month apart

-High- dose inactivated vaccines: 60 µg per strain; TIV: 180 µg; QIV 240 µg total. Vaccines with doses between 15 and 60 µg per strains were considered as standard dose (including vaccines prepared with the DNA recombinant technique)

-Adjuvanted vaccines: we included virosomal or liposome adjuvanted (one node) and MF59 or AS03 with or without IL2 (another node), both TIV or QIV. Vaccines adjuvanted using other methods (e.g TLR ligand or polyoxidonium) were excluded

-Recombinant trivalent inactivated (rHA): we included any dose

-Intradermal inactivated vaccines: we included only doses of 9 or 15 µg per strains; also QIV intradermal (doses of 9 or 15 µg per strains) were included in a separate node

-Trivalent live attenuated nasal spray

-Quadrivalent live attenuated nasal spray

Box 1. Summary of the different types of seasonal influenza vaccines available

Inactivated influenza vaccines (IIVs)

Consist of viruses killed through the use of different chemical compounds in production processes based on different technologies. IIVs are injectable and include: i) whole virion IIVs, containing whole viral particles; ii) split IIVs, in which the virus is inactivated with disrupting agents, yielding surface and internal antigens; iii) subunit IIVs, containing only surface antigens – hemagglutinin (HA) and neuraminidase (NA) - purified from other viral components. Among these, split and subunit IIVs are more commonly used due to improved tolerability. For split and subunit IIVs, the standard antigen dose corresponds to 15 µg of HA per viral strain. To enhance the vaccine-induced immune response, two types of vaccines were specifically developed: formulations that include the use of adjuvants (such as AS03 or MF59, oil-in-water emulsions) or virosomes/liposomes (vesicles with phospholipid membranes incorporating viral antigens), and high-dose (HD) influenza vaccines containing an amount of antigen four times higher than the standard dose (60 µg of HA per strain).

Most IIVs are administered intramuscularly (IM); intradermal (ID) preparations that enhance the immune response thanks to the simultaneous stimulation of innate and adaptive immunity are also available.

IIVs can be produced in eggs or in cell-cultures. Egg-based IIVs have been licensed in the US and Europe for use in adults and children six months and older, with the MF59-adjuvanted or high dose vaccines licensed for use in the elderly. Cell-based IIVs for individuals who are allergic to eggs are licensed in the US, and are under evalution in Europe.

Live-attenuated influenza vaccines (LAIVs)

Containing active whole viral particles; those currently available are made of temperature-sensitive viruses that can survive and replicate only at the low temperature of the upper respiratory tract (cold-adapted); they are administered intranasally (IN). LAIVs are licensed for persons aged 2 to 59 years old and are recommended for healthy, non-pregnant individuals who do not have chronic medical conditions, due to the relative lack of data in the other populations.

Recombinant influenza vaccines (RIVs)

Recombinant technologies have the added benefit of substantially reducing production times compared with egg-based and cell-based technologies, as production can begin once a virus genome has been sequenced rather than starting with a live virus. A recombinant-HA expressed in insect cells using baculovirus vectors was licensed for use in adults aged 18–49 years old in the US.

Nodes in the network

Label	Vaccine
placebo	placebo/no vaccine
3-IIV	trivalent inactivated intramuscular
3-IIV HD	trivalent inactivated high dose intramuscular
3-IIV ID	trivalent inactivated intradermal
3-IIV MF59/AS03-adj	trivalent inactivated adjuvanted with MF59/ASO3 intramuscular
3-IIV vir/lip-adj	trivalent inactivated adjuvanted with Virosome/liposome intramuscular
3-RIV	trivalent recombinant intramuscular
4-IIV	quadrivalent inactivated intramuscular
4-IIV HD	quadrivalent inactivated high dose intramuscular
4-IIV ID	quadrivalent inactivated intradermal
4-IIV MF59/AS03-adj	quadrivalent inactivated adjuvanted with MF59/ASO3 intramuscular
4-IIV vir/lip-adj	quadrivalent inactivated adjuvanted with virosome/liposome intramuscular
4-RIV	quadrivalent recombinant intramuscular
3-LAIV	trivalent live attenuated intranasal
4-LAIV	quadrivalent live attenuated intranasal

References of excluded studies

	References	Reasons for exclusion
1.	Agarkhedkar S, Chhatwal J, Kompithra RZ, Lalwani SK, Narayan A, Muninarayanaswam V, et al. Immunogenicity and safety of an intramuscular split-virion quadrivalent inactivated influenza vaccine in individuals aged ≥ 6 months in India. *Human vaccines & immunotherapeutics*. 2019;15(4):973-7.	No relevant outcome
2.	Airey J, Albano FR, Sawlwin DC, et al. Immunogenicity and safety of a quadrivalent inactivated influenza virus vaccine compared with a comparator quadrivalent inactivated influenza vaccine in a pediatric population: a phase 3, randomized noninferiority study. *Vaccine.* 2017;35(20):2745-2752.	No relevant comparator
3.	Ambati A, Boas LS, Ljungman P, et al. Evaluation of pretransplant influenza vaccination in hematopoietic SCT: a randomized prospective study. *Bone marrow transplantation.* Jun 2015;50(6):858-864.	No relevant outcome
4.	Ansaldi F, Bacilieri S, Durando P, Sticchi L, Valle L, Montomoli E, et al. Cross-protection by MF59-adjuvanted influenza vaccine: neutralizing and haemagglutination-inhibiting antibody activity against A(H3N2) drifted influenza viruses. *Vaccine*. 2008;26(12):1525-9.	No relevant outcome
5.	Ansaldi F, Zancolli M, Durando P, Montomoli E, Sticchi L, Del Giudice G, et al. Antibody response against heterogeneous circulating influenza virus strains elicited by MF59- and non-adjuvanted vaccines during seasons with good or partial matching between vaccine strain and clinical isolates. *Vaccine*. 2010;28(25):4123-9.	No relevant outcome
6.	Arakane R, Annaka R, Takahama A, et al. Superior immunogenicity profile of the new intradermal influenza vaccine compared to the standard subcutaneous vaccine in subjects 65 years and older: a randomized controlled phase III study. *Vaccine.* 2015;33(48):6650-6658.	No relevant comparator
7.	Arakane R, Nakatani H, Fujisaki E, Takahama A, Ishida K, Yoshiike M, et al. Immunogenicity and safety of the new intradermal influenza vaccine in adults and elderly: A randomized phase 1/2 clinical trial. *Vaccine*. 2015;33(46):6340-50.	No vaccine of interest
8.	Baldo V, Menegon T, Bonello C, Floreani A, Trivello R. Comparison of three different influenza vaccines in institutionalised elderly. *Vaccine.* 2001;19(25-26):3472-3475.<br>Baldo V, Baldovin T, Pellegrini M, et al. Immunogenicity of three different influenza vaccines against homologous and heterologous strains in nursing home elderly residents. Clinical & developmental immunology. 2010;2010:517198.	No relevant outcome
9.	Banzhoff A, Kaniok W, Muszer A. Effectiveness of an influenza vaccine used in Poland in the 1998-1999 influenza season. *Immunological investigations.* May 2001;30(2):103-113.	Secondary analysis of already included studies
10.	Belshe RB, Gruber WC, Mendelman PM, et al. Correlates of immune protection induced by live, attenuated, cold-adapted, trivalent, intranasal influenza virus vaccine. *Journal of infectious diseases.* 2000;181(3):1133-1137.	No vaccine of interest
11.	Belshe RB, Gruber WC. Safety, efficacy and effectiveness of cold-adapted, live, attenuated, trivalent, intranasal influenza vaccine in adults and children. *Philosophical transactions of the royal society of london. Series B, biological sciences.* 2001;356(1416):1947-1951.	Secondary analysis of already included studies
12.	Belshe RB, Newman FK, Cannon J, et al. Serum antibody responses after intradermal vaccination against influenza. *The New England journal of medicine.* Nov 25 2004;351(22):2286-2294.	Secondary analysis of already included studies
13.	Bernstein DI, Guptill J, Naficy A, Nachbagauer R, Berlanda-Scorza F, Feser J, et al. Immunogenicity of chimeric haemagglutinin-based, universal influenza virus vaccine candidates: interim results of a randomised, placebo-controlled, phase 1 clinical trial. *The Lancet Infectious diseases*. 2020;20(1):80-91.	No vaccine of interest
14.	Boyce TG, Hsu HH, Sannella EC, et al. Safety and immunogenicity of adjuvanted and unadjuvanted subunit influenza vaccines administered intranasally to healthy adults. *Vaccine.* Sep 15 2000;19(2-3):217-226.	No vaccine of interest
15.	Branagan A, Duffy E, Foster C, et al. Two dose series of high-dose influenza vaccine is associated with longer duration of serologic immunity in patients with plasma cell disorders. *Blood.* 2017;130.	No relevant outcome
16.	Burgess T, Richard S, Collins L, Colombo R, Ganesan A, Geaney C, et al. Pragmatic assessment of influenza vaccine effectiveness in the DOD (PAIVED): Influenza-like-illness rates in year 1. *Open forum infectious diseases.* 2019;6:S990.<br>Burgess T, Richard S, Collins L, Colombo R, Ganesan A, Geaney C, et al. Pragmatic assessment of influenza vaccine effectiveness in the DOD (PAIVED): Methods. Open forum infectious diseases. 2019;6:S969.	No parallel RCT
17.	Caldera F, Saha S, Wald A, Grimes I, Hillman L, Zhang Y, et al. Randomized trial evaluating the immunogenicity of high dose vs. standard dose influenza vaccine in IBD patients on anti-tnf monotherapy. *Gastroenterology*. 2018;154(6):S-69-.	Secondary analysis of already included studies
18.	Camilloni B, Basileo M, Di Martino A, Donatelli I, Iorio AM. Antibody responses to intradermal or intramuscular MF59-adjuvanted influenza vaccines as evaluated in elderly institutionalized volunteers during a season of partial mismatching between vaccine and circulating A(H3N2) strains. *Immunity & ageing : I & A.* 2014;11:10.	No relevant outcome

19.	Castro M, Dozor A, Fish J, et al. The safety of inactivated influenza vaccine in adults and children with asthma. *New England Journal of Medicine.* 2001;345(21):1529-1536.	No parallel RCT
20.	Celik A, Orscelik O. Effects of influenza vaccination in patients with heart failure. *Herz.* 2017;42(3):325-326.	No parallel RCT
21.	Chuaychoo B, Kositanont U, Rittayamai N, et al. The immunogenicity of the intradermal injection of seasonal trivalent influenza vaccine containing influenza A(H1N1) pdm09 in COPD patients soon after a pandemic. *Human vaccines & immunotherapeutics.* Jul 2 2016;12(7):1728-1737.	No relevant comparator
22.	Chuaychoo B, Wongsurakiat P, Nana A, Kositanont U, Maranetra KN. The immunogenicity of intradermal influenza vaccination in COPD patients. *Vaccine*. 2010;28(24):4045-51.	No vaccine of interest
23.	Chumakov MP, Beregovskii NA, Linev MB, et al. [Use of highly purified subvirion trivalent flue vaccine ("Grippovak") in groups with a high risk of complications]. *Zhurnal mikrobiologii, epidemiologii, i immunobiologii.* Mar 1992(3):55-57.	No parallel RCT
24.	Ciszewski A, Billinska ZT, Brydak LB. Influenza vaccination in secondary prevention from coronary ischemic events in Coronary Artery Disease (FLUCAD). *ACC Cardiosource Review Journal.* 2008;17(4):34.	No relevant outcome
25.	Claeys C, Drame M, Garcia-Sicilia J, Zaman K, Carmona A, Tran PM, et al. Assessment of an optimized manufacturing process for inactivated quadrivalent influenza vaccine: a phase III, randomized, double-blind, safety and immunogenicity study in children and adults. *BMC infectious diseases*. 2018;18(1).	No relevant comparator
26.	Clover RD, Crawford S, Glezen WP, Taber LH, Matson CC, Couch RB. Comparison of heterotypic protection against influenza A/Taiwan/86 (H1N1) by attenuated and inactivated vaccines to A/Chile/83-like viruses. *The Journal of infectious diseases.* Feb 1991;163(2):300-304.	No parallel RCT
27.	Colombo R, Richard S, Schofield C, Collins L, Ganesan A, Geaney C, et al. Pragmatic assessment of influenza vaccine effectiveness in the DOD (PAIVED): immunogenicity sub-study. *Open forum infectious diseases*. 2019;6:S971-S2.	No parallel RCT
28.	Conne P, Gauthey L, Vernet P, et al. Immunogenicity of trivalent subunit versus virosome-formulated influenza vaccines in geriatric patients. *Vaccine.* 1997;15(15):1675-1679.	No relevant outcome
29.	Cook Q, Burks AW. Safety and immunogenicity of a recombinant influenza vaccine: a randomized trial. *Pediatrics*. 2018;142:S273-S4.	Secondary analysis of already included studies
30.	Cools HJM, Gussekloo J, Remmerswaal JEM, Remarque EJ, Kroes ACM. Benefits of increasing the dose of influenza vaccine in residents of long-term care facilities: A randomized placebo-controlled trial. *Journal of Medical Virology*. 2009;81(5):908-914.	No relevant outcome
31.	Cooper C, Thorne A, Klein M, Conway B, Boivin G, Haase D, et al. Immunogenicity is not improved by increased antigen dose or booster dosing of seasonal influenza vaccine in a randomized trial of HIV infected adults. *PloS one*. 2011;6(3):e17758.	No vaccine of interest
32.	Cordero E, Roca-Oporto C, Bulnes-Ramos A, Aydillo T, Gavaldà J, Moreno A, et al. Two Doses of Inactivated Influenza Vaccine Improve Immune Response in Solid Organ Transplant Recipients: Results of TRANSGRIPE 1-2, a Randomized Controlled Clinical Trial. *Clinical infectious diseases : an official publication of the Infectious Diseases Society of America*. 2017;64(7):829-38.	No vaccine of interest
33.	Danier J, Rivera L, Claeys C, Dbaibo G, Jain VK, Kosalaraksa P, et al. Clinical Presentation of Influenza in Children 6 to 35 Months of Age: Findings from a Randomized Clinical Trial of Inactivated Quadrivalent Influenza Vaccine. *Pediatric Infectious Disease Journal*. 2019;38(8):866-72.	Secondary analysis of already included studies
34.	Dbaibo G, Amanullah A, Claeys C, Izu A, Jain VK, Kosalaraksa P, et al. Quadrivalent Influenza Vaccine Prevents Illness and Reduces Healthcare Utilization Across Diverse Geographic Regions During Five Influenza Seasons: A Randomized Clinical Trial. *The Pediatric infectious disease journal.* 2020;39(1):e1-e10.	Secondary analysis of already included studies
35.	de B, Zanasi A, Ragusa S, Gluck R, Herzog C. An open-label comparison of the immunogenicity and tolerability of intranasal and intramuscular formulations of virosomal influenza vaccine in healthy adults. *Clinical therapeutics.* Jan 2002;24(1):100-111.	No relevant comparator
36.	Della Cioppa G, Nicolay U, Lindert K, et al. A dose-ranging study in older adults to compare the safety and immunogenicity profiles of MF59(R)-adjuvanted and non-adjuvanted seasonal influenza vaccines following intradermal and intramuscular administration. *Human vaccines & immunotherapeutics.* 2014;10(6):1701-1710.	No relevant comparator
37.	Diallo A, Diop OM, Diop D, Niang MN, Sugimoto JD, Ortiz JR, et al. Effectiveness of Seasonal Influenza Vaccination in Children in Senegal During a Year of Vaccine Mismatch: A Cluster-randomized Trial. *Clinical infectious diseases : an official publication of the Infectious Diseases Society of America*. 2019;69(10):1780-8.	No parallel RCT
38.	Donatelli I, Zannolli R, Fuiano L, Biasio LR. Influenza vaccine in immunogenically naive healthy infants. *European journal of pediatrics.* Nov 1998;157(11):949-950.	No relevant comparator

39.	Durrer P, Gluck U, Spyr C, et al. Mucosal antibody response induced with a nasal virosome-based influenza vaccine. *Vaccine.* Oct 1 2003;21(27-30):4328-4334.	No vaccine of interest
40.	Edwards KM, Dupont WD, Westrich MK, Plummer Jr WD, Palmer PS, Wright PF. A randomized controlled trial of cold-adapted and inactivated vaccines for the prevention of influenza A disease. *Journal of Infectious Diseases.* 1994;169(1):68-76.	No vaccine of interest
41.	El'shina GA, Masalin Iu M, Shervali VI, et al. [The trivalent polymer-subunit influenza vaccine Grippol studied in a controlled epidemiological trial (1)]. *Voenno-meditsinskii zhurnal.* Aug 1996;317(8):57-60.	No vaccine of interest
42.	Esposito S, Fling J, Chokephaibulkit K, de Bruijn M, Oberye J, Zhang B, et al. Immunogenicity and Safety of an MF59-adjuvanted Quadrivalent Seasonal Influenza Vaccine in Young Children at High Risk of Influenza-associated Complications: A Phase III, Randomized, Observer-blind, Multicenter Clinical Trial. *The Pediatric infectious disease journal*. 2020;39(8):e185-e91.	Not relevant comparator
43.	Esposito S, Marchisio P, Ansaldi F, et al. A randomized clinical trial assessing immunogenicity and safety of a double dose of virosomal-adjuvanted influenza vaccine administered to unprimed children aged 6-35 months. *Vaccine.* Aug 31 2010;28(38):6137-6144.	No relevant outcome
44.	Esposito S, Marchisio P, Bosis S, et al. Clinical and economic impact of influenza vaccination on healthy children aged 2-5 years. *Vaccine.* Jan 30 2006;24(5):629-635.	No relevant outcome
45.	Esposito S, Marchisio P, Cavagna R, et al. Effectiveness of influenza vaccination of children with recurrent respiratory tract infections in reducing respiratory-related morbidity within the households. *Vaccine.* Jul 4 2003;21(23):3162-3168.	No relevant comparator
46.	Eun BW, Lee TJ, Lee J, Kim KH, Kim DH, Jo DS, et al. A Randomized, Double-blind, Active-controlled Phase III Trial of a Cell Culture-derived Quadrivalent Inactivated Influenza Vaccine in Healthy South Korean Children and Adolescents 6 Months to 18 Years of Age. *The Pediatric infectious disease journal*. 2019;38(9):e209-e15.	No vaccine of interest
47.	Forrest BD, Pride MW, Dunning AJ, et al. Correlation of cellular immune responses with protection against culture-confirmed influenza virus in young children. *Clinical and vaccine immunology: CVI.* Jul 2008;15(7):1042-1053.	No relevant outcome
48.	Fritz Verhees RA, Thijs C, Ambergen T, Dinant GJ, Knottnerus JA. Influenza vaccination in the elderly: 25 years follow-up of a randomized controlled trial. No impact on long-term mortality. *PloS one*. 2019;14(5).	No parallel RCT
49.	Gaughran F, Walwyn R, Lambkin-Williams R, et al. Flu: effect of vaccine in elderly care home residents: a randomized trial. *Journal of the American Geriatrics Society.* Dec 2007;55(12):1912-1920.	No relevant comparator
50.	Gelinck LB, Bemt BJ, Marijt WA, et al. Intradermal influenza vaccination in immunocompromized patients is immunogenic and feasible. *Vaccine.* 2009;27(18):2469-2474.	No relevant comparator
51.	Glesby MJ, Hoover DR, Farzadegan H, Margolick JB, Saah AJ. The effect of influenza vaccination on human immunodeficiency virus type 1 load: a randomized, double-blind, placebo-controlled study. *The Journal of infectious diseases.* Dec 1996;174(6):1332-1336.	No relevant outcome
52.	Glück R, Mischler R, Finkel B, Que JU, Scarpa B, Cryz Jr SJ. Immunogenicity of new virosome influenza vaccine in elderly people. *Lancet.* 1994;344(8916):160-163.	No relevant outcome
53.	Gordon DL, Sajkov D, Honda-Okubo Y, et al. Human Phase 1 trial of low-dose inactivated seasonal influenza vaccine formulated with Advax delta inulin adjuvant. *Vaccine.* Jul 19 2016;34(33):3780-3786.	No vaccine of interest
54.	Gorse GJ, Campbell MJ, Otto EE, Powers DC, Chambers GW, Newman FK. Increased anti-influenza A virus cytotoxic T cell activity following vaccination of the chronically ill elderly with live attenuated or inactivated influenza virus vaccine. *The Journal of infectious diseases.* Jul 1995;172(1):1-10.	No vaccine of interest
55.	Gorse GJ, O'Connor TZ, Young SL, et al. Efficacy trial of live, cold-adapted and inactivated influenza virus vaccines in older adults with chronic obstructive pulmonary disease: a VA cooperative study. *Vaccine.* May 16 2003;21(17-18):2133-2144.	No vaccine of interest
56.	Gorse GJ, Otto EE, Daughaday CC, et al. Influenza virus vaccination of patients with chronic lung disease. *Chest.* Nov 5 1997;112(5):1221-1233.	No vaccine of interest
57.	Gravenstein S, Davidson HE, Han LF, Ogarek JA, Dahal R, Gozalo PL, et al. Feasibility of a cluster-randomized influenza vaccination trial in U.S. nursing homes: Lessons learned. *Human vaccines & immunotherapeutics*. 2018;14(3):736-43.	No parallel RCT
58.	Gravenstein S, Davidson HE, McConeghy K, Han L, Canaday D, Saade E, et al. A cluster-randomized trial of adjuvanted trivalent influenza vaccine vs. standard dose in US nursing homes. *Open forum infectious diseases*. 2018;5:S296-.	No parallel RCT
59.	Gravenstein S, Davidson HE, Taljaard M, Ogarek J, Gozalo P, Han L, et al. Comparative effectiveness of high-dose versus standard-dose influenza vaccination on numbers of US nursing home residents admitted to hospital: a cluster-randomised trial. *The Lancet Respiratory medicine*. 2017;5(9):738-46.	No parallel RCT

60.	Grigoreva EP RA, Rudenko LG, Ramires A, Barro M, Lisovskaya KV, Klimov AI, Aleksandrova GI. Study of the Immunogenic Properties and Prophylactic Efficacy of a Live Influenza Polyvalent Vaccine in Children aged 5-14 years. *Voprosy Virusologii* 1994;39(1):26-29.	No relevant outcome
61.	Grigor'eva EP, Desheva Iu A, Donina SA, et al. [The comparative characteristics of the safety, immunogenic activity and prophylactic potency of the adult and children types of live influenza vaccine in schoolchildren aged 7-14 years]. *Voprosy virusologii.* Jul-Aug 2002;47(4):24-27.	No relevant outcome
62.	Gruber WC, Campbell PW, Thompson JM, Reed GW, Roberts B, Wright PF. Comparison of live attenuated and inactivated influenza vaccines in cystic fibrosis patients and their families: results of a 3-year study. *The Journal of infectious diseases.* Feb 1994;169(2):241-247.	No relevant comparator
63.	Gurfinkel EP, Fuente RL, Mendiz O, Mautner B. Influenza vaccine pilot study in acute coronary syndromes and planned percutaneous coronary interventions: the FLU Vaccination Acute Coronary Syndromes (FLUVACS) Study. *Circulation.* 2002;105(18):2143-2147.<br>Gurfinkel EP, Leon de la Fuente R, Mendiz O, Mautner B. Flu vaccination in acute coronary syndromes and planned percutaneous coronary interventions (FLUVACS) Study. European heart journal. 2004;25(1):25-31.	No relevant outcome
64.	Halloran ME, Piedra PA, Longini IM, Jr., et al. Efficacy of trivalent, cold-adapted, influenza virus vaccine against influenza A (Fujian), a drift variant, during 2003-2004. *Vaccine.* May 16 2007;25(20):4038-4045.	No parallel RCT
65.	Halperin SA, Smith B, Nolan C, Shay J, Kralovec J. Safety and immunoenhancing effect of a Chlorella-derived dietary supplement in healthy adults undergoing influenza vaccination: randomized, double-blind, placebo-controlled trial. *CMAJ: Canadian Medical Association journal = journal de l'Association medicale canadienne.* Jul 22 2003;169(2):111-117.	No relevant comparator
66.	Houser KV, Yamshchikov GV, Bellamy AR, May J, Enama ME, Sarwar U, et al. DNA vaccine priming for seasonal influenza vaccine in children and adolescents 6 to 17 years of age: a phase 1 randomized clinical trial. *PloS one*. 2018;13(11):e0206837.	No vaccine of interest
67.	Hu Y, Chu K, Lavis N, Li X, Liang B, Liu S, et al. Immunogenicity and safety of a trivalent inactivated influenza vaccine produced in Shenzhen, China versus a comparator influenza vaccine: a phase IV randomized study. *Human vaccines & immunotherapeutics*. 2019;15(5):1066-9.	Not relevant comparator
68.	Hung IF, Zhang AJ, To KK, Chan JF, Li C, Zhu HS, et al. Immunogenicity of intradermal trivalent influenza vaccine with topical imiquimod: a double blind randomized controlled trial. *Clinical infectious diseases: an official publication of the Infectious Diseases Society of America*. 2014;59(9):1246-55.	No vaccine of interest
69.	Iorio AM, Camilloni B, Basileo M, Guercini F, Conti S, Ferrante F, et al. Influenza vaccination in patients on long-term anticoagulant therapy. *Vaccine*. 2006;24(44-46):6624-8.	No parallel RCT
70.	Iorio AM, Francisci D, Camilloni B, et al. Antibody responses and HIV-1 viral load in HIV-1-seropositive subjects immunised with either the MF59-adjuvanted influenza vaccine or a conventional non-adjuvanted subunit vaccine during highly active antiretroviral therapy. *Vaccine.* 2003;21(25-26):3629-3637.	No relevant outcome
71.	Jain VK, Domachowske JB, Wang L, et al. Time to change dosing of inactivated quadrivalent influenza vaccine in young children: Evidence from a phase III, randomized, controlled trial. *Journal of the Pediatric Infectious Diseases Society.* 2017;6(1):9-19.	No relevant comparator
72.	Jamshed S, Falsey AR, Thushara P, Walker J, Walsh EE. Randomized study comparing high-dose (HD) influenza vaccine to standard-dose (SD) influenza vaccine in patients with breast cancer age < 65 receiving chemotherapy. *Journal of Clinical Oncology.* 2017;35(15_suppl):e21695-e21695.	No relevant outcome
73.	Jansen AG, Sanders EA, Hoes AW, van Loon AM, Hak E. Effects of influenza plus pneumococcal conjugate vaccination versus influenza vaccination alone in preventing respiratory tract infections in children: a randomized, double-blind, placebo-controlled trial. *The Journal of pediatrics.* Dec 2008;153(6):764-770.	No vaccine of interest
74.	Jianping H, Xin F, Changshun L, et al. Assessment of effectiveness of Vaxigrip. *Vaccine.* Jul 30 1999;17 Suppl 1:S57-58.	No parallel RCT
75.	Jing-Xia G, Yu-Liang Z, Jin-Feng L, Shu-Zhen L, Guo-Yang L, Qi L. Safety and effectiveness assessment of 2011–2012 seasonal influenza vaccine produced in China: a randomized trial. *Postgraduate Medicine.* 2017;129(8):907-914.	No relevant comparator
76.	Jo YM, Song JY, Hwang IS, et al. Dose sparing strategy with intradermal influenza vaccination in patients with solid cancer. *Journal of medical virology.* Apr 2009;81(4):722-727.	No relevant comparator
77.	Jo YM, Song JY, Hwang IS, Lee J, Oh SC, Kim JS, et al. Dose sparing strategy with intradermal influenza vaccination in patients with solid cancer. *Journal of medical virology*. 2009;81(4):722-7.	No vaccine of interest
78.	Kang JH, Oh CE, Lee J, et al. Safety and immunogenicity of a new trivalent inactivated split-virus influenza vaccine in healthy Korean children: a randomized, double-blinded, active-controlled, phase III study. *Journal of Korean medical science.* Nov 2011;26(11):1421-1427.	No relevant comparator
79.	Katz J, Englund JA, Steinhoff MC, Khatry SK, Shrestha L, Kuypers J, et al. Impact of Timing of Influenza Vaccination in Pregnancy on Transplacental Antibody Transfer, Influenza Incidence, and Birth Outcomes: a Randomized Trial in Rural Nepal. *Clinical infectious diseases*. 2018;67(3):334-40.	Secondary analysis of already included studies

80. Keitel W, Couch R, Bond N, Adair S, Nest G, Dekker C. Pilot evaluation of influenza virus vaccine (IVV) combined with adjuvant. *Vaccine.* 1993;11(9):909-913.	No relevant outcome
81. Keitel WA, Couch RB, Quarles JM, Cate TR, Baxter B, Maassab HF. Trivalent attenuated cold-adapted influenza virus vaccine: reduced viral shedding and serum antibody responses in susceptible adults. *The Journal of infectious diseases.* Feb 1993;167(2):305-311.	No parallel RCT
82. Kenney RT, Frech SA, Muenz LR, Villar CP, Glenn GM. Dose sparing with intradermal injection of influenza vaccine. *The New England journal of medicine.* Nov 25 2004;351(22):2295-2301.	No relevant comparator
83. Khan AS, Polezhaev F, Vasiljeva R, et al. Comparison of US inactivated split-virus and Russian live attenuated, cold-adapted trivalent influenza vaccines in Russian schoolchildren. *The Journal of infectious diseases.* Feb 1996;173(2):453-456.	No relevant outcome
84. Khanlou H, Sanchez S, Babaie M, Chien C, Hamwi G, Ricaurte JC, et al. The safety and efficacy of dose-sparing intradermal administration of influenza vaccine in human immunodeficiency virus-positive patients. *Archives of internal medicine*. 2006;166(13):1417.	No relevant outcome
85. Kiderman A, Furst A, Stewart B, Greenbaum E, Morag A, Zakay-Rones Z. A double-blind trial of a new inactivated, trivalent, intra-nasal anti-influenza vaccine in general practice: relationship between immunogenicity and respiratory morbidity over the winter of 1997-98. *Journal of clinical virology: the official publication of the Pan American Society for Clinical Virology.* Feb 2001;20(3):155-161.	No relevant outcome
86. Kmiecik T, Arnoux S, Kobryn A, Gorski P. Influenza vaccination in adults with asthma: safety of an inactivated trivalent influenza vaccine. *The Journal of asthma: official journal of the Association for the Care of Asthma.* Dec 2007;44(10):817-822.	No parallel RCT
87. Kostinov MP, Cherdantsev AP, Akhmatova NK, Praulova DA, Kostinova AM, Akhmatova EA, et al. Immunogenicity and safety of subunit influenza vaccines in pregnant women. *ERJ open research*. 2018;4(2).	No vaccine of interest
88. Kostinov MP, Cherdantsev AP, Kuselman AI, Akhmatova NK, Kostinova AM, Deryabina EV, et al. Prospective randomized open-label comparative study of immunogenicity after subunit and polymeric subunit influenza vaccines administration among mothers and infants. *Human vaccines & immunotherapeutics*. 2018;14(12):2971-8.	Secondary analysis of already included studies
89. Kumar R, Amarchand R, Narayan VV, Saha S, Lafond KE, Kapoor SK, et al. Challenges in conducting a community-based influenza vaccine trial in a rural community in northern India. *Human vaccines & immunotherapeutics*. 2018;14(8):1909-13.	No parallel RCT
90. Kunzi V, Klap JM, Seiberling MK, et al. Immunogenicity and safety of low dose virosomal adjuvanted influenza vaccine administered intradermally compared to intramuscular full dose administration. *Vaccine.* Jun 2 2009;27(27):3561-3567.	No relevant comparator
91. Langley J, Pastural E, Halperin S, McNeil S, Elsherif M, Mackinnon-Cameron D, et al. A randomized controlled study to evaluate the safety and reactogenicity of a novel rVLP-based plant virus nanoparticle adjuvant combined with seasonal trivalent influenza vaccine following single immunization in healthy adults 18–50 years of age. *Vaccines*. 2020;8(3):1-11.<br>Langley JM, Pastural E, Halperin S, McNeil S, ElSherif M, MacKinnon-Cameron D, et al. A phase I randomized, observer-blind, controlled, dose escalation trial of the safety and tolerability of a single intramuscular dose of a pal adjuvant (laboratory code, FB-631) co-administered with seasonal TIV (2013-2014) to healthy adults ≥18-50 years of age. *Open forum infectious diseases.* 2019;6:S966.	No vaccine of interest
92. Langley JM, Aoki F, Ward BJ, et al. A nasally administered trivalent inactivated influenza vaccine is well tolerated, stimulates both mucosal and systemic immunity, and potentially protects against influenza illness. *Vaccine.* Feb 24 2011;29(10):1921-1928.	No vaccine of interest
93. Langley JM, Halperin SA, McNeil S, et al. Safety and immunogenicity of a Proteosome -trivalent inactivated influenza vaccine, given nasally to healthy adults. *Vaccine.* 2006;24(10):1601-1608.	No vaccine of interest
94. Lee J, Lee KY, Kim JH, Kim CS, Eun BW, Kim HM, et al. Safety and Immunogenicity of an Egg-Cultivated Quadrivalent Inactivated Split-virion Influenza Vaccine (GC3110A) in Healthy Korean Children: a Randomized, Double-blinded, Active-controlled Phase III Study. *Journal of Korean medical science.* 2018;33(13):e100.	No parallel RCT
95. Lee JH, Cho HK, Kim KH, Lee J, Kim YJ, Eun BW, et al. Evaluation of Waning Immunity at 6 Months after Both Trivalent and Quadrivalent Influenza Vaccination in Korean Children Aged 6-35 Months. *Journal of Korean medical science.* 2019;34(46):e279.	Not relevant comparator
96. Lewis KDC, Ortiz JR, Rahman MZ, Levine MZ, Rudenko L, Wright PF, et al. Immunogenicity and Viral Shedding of Russian-Backbone, Seasonal, Trivalent, Live, Attenuated Influenza Vaccine in a Phase II, Randomized, Placebo-Controlled Trial Among Preschool-Aged Children in Urban Bangladesh. *Clinical infectious diseases*. 2019;69(5):777-85.	No relevant outcome
97. L'Huillier A G, Ferreira VH, Hirzel C, Natori Y, Slomovic J, Ku T, et al. Cell-Mediated Immune Responses After Influenza Vaccination of Solid Organ Transplant Recipients: Secondary Outcomes Analyses of a Randomized Controlled Trial. *The Journal of infectious diseases*. 2020;221(1):53-62.	Secondary analysis of already included studies

98. Ljungman P, Nahi H, Linde A. Vaccination of patients with haematological malignancies with one or two doses of influenza vaccine: a randomised study. *British journal of haematology.* Jul 2005;130(1):96-98.	No relevant comparator
99. Loeb N, Andrew MK, Loeb M, Kuchel GA, Haynes L, McElhaney JE, et al. Frailty Is Associated With Increased Hemagglutination-Inhibition Titers in a 4-Year Randomized Trial Comparing Standard- and High-Dose Influenza Vaccination. *Open forum infectious diseases*. 2020;7(5):ofaa148.	No relevant outcome
100. Loubet P, Launay O. Maternal influenza immunisation to reduce influenza infections and low birthweight in infants in resource-limited countries. *The Lancet Infectious Diseases.* 2017;17(9):890-892.	No parallel RCT
101. Maeda T, Shintani Y, Miyamoto H, et al. Prophylactic effect of inactivated influenza vaccine on young children. *Pediatrics international: official journal of the Japan Pediatric Society.* Feb 2002;44(1):43-46.	No relevant outcome
102. Maeda T, Shintani Y, Nakano K, Terashima K, Yamada Y. Failure of inactivated influenza A vaccine to protect healthy children aged 6-24 months. *Pediatrics international: official journal of the Japan Pediatric Society.* Apr 2004;46(2):122-125.	No relevant outcome
103. Mallory R, Nyborg AC, Kalyani R, Tsai LF, Block SL, Dubovsky F. A randomized study to evaluate the shedding and immunogenicity of H1N1 strains in trivalent and quadrivalent formulations of flumist in children 24 to <48 months of age. *Open forum infectious diseases*. 2018;5:S564-S5.	Congress presentation of an article already included
104. Manuel O, Humar A, Berutto C, et al. Low-dose intradermal versus intramuscular trivalent inactivated seasonal influenza vaccine in lung transplant recipients. *The Journal of heart and lung transplantation: the official publication of the International Society for Heart Transplantation.* Jun 2011;30(6):679-684.	No relevant comparator
105. Marchisio P, Cavagna R, Maspes B, et al. Efficacy of intranasal virosomal influenza vaccine in the prevention of recurrent acute otitis media in children. *Clinical infectious diseases.* 2002;35(2):168-174.	No vaccine of interest
106. Maves R, Utz G, Richard S, Smith M, Collins L, Myers C, et al. Pragmatic assessment of influenza vaccine effectiveness in the DOD (PAIVED), influenza-like-illnesses (ILIS) sub-study at the marine corps recruit depot-San Diego, CA (MCRD-SD) during the 2018-2019 influenza season. *Open forum infectious diseases*. 2019;6:S970-S1.	No parallel RCT
107. Mc Lean H, Levine M, King J, Spencer S, Flannery B, Belongia E. Randomized trial of high dose, adjuvanted, and standard inactivated influenza vaccine immune response against egg-and cell-propagated vaccine strains in older adults, 2016-2017 season. *Open forum infectious diseases*. 2018;5:S293-.	No relevant outcome
108. McConeghy KW, Davidson HE, Canaday DH, Han L, Saade E, Mor V, et al. Cluster-randomized trial of adjuvanted vs. non-adjuvanted trivalent influenza vaccine in 823 U.S. nursing homes. *Clinical infectious diseases : an official publication of the Infectious Diseases Society of America*. 2020.	No parallel RCT
109. Meijer WJ, Wensing AMJ, Bos AA, Kuiphuis JCF, Hagelen EMM, Wilschut JC, et al. Influenza vaccination in healthcare workers; comparison of side effects and preferred route of administration of intradermal versus intramuscular administration. *Vaccine*. 2017;35(11):1517-23.	No parallel RCT
110. Mendelman PM, Cordova J, Cho I. Safety, efficacy and effectiveness of the influenza virus vaccine, trivalent, types A and B, live, cold-adapted (CAIV-T) in healthy children and healthy adults. *Vaccine.* 2001;19(17-19):2221-2226.	Secondary analysis of already included studies
111. Menegon T, Baldo V, Bonello C, Dalla Costa D, Tommaso A, Trivello R. Influenza vaccines: antibody responses to split virus and MF59-adjuvanted subunit virus in an adult population. *European journal of epidemiology*. 1999;15(6):573-576.	No relevant outcome
112. Metanat M., Salehi M., Sharifi-Mood B., M. S. Serum Antibody Responses after Intradermal Injection of Influenza Vaccine. *International Journal of Virology.* 2006;2:35-8.	No vaccine of interest
113. Miller RL, Cheng M, DiMango EA, Geromanos K, Rothman PB. T-cell responses and hypersensitivity to influenza and egg antigens among adults with asthma immunized with the influenza vaccine. *The Journal of allergy and clinical immunology.* Sep 2003;112(3):606-608.	No parallel RCT
114. Mo Z, Nong Y, Liu S, et al. Immunogenicity and safety of a trivalent inactivated influenza vaccine produced in Shenzhen, China. *Human vaccines and immunotherapeutics.* 2017:1-7.	No parallel RCT
115. Moehling KK, Zimmerman RK, Nowalk MP, Jeng Lin C, Martin JM, Alcorn JF, et al. A randomized controlled trial of antibody response to 2018–19 cell-based vs. egg-based quadrivalent inactivated influenza vaccine in children. *Vaccine*. 2020.	No vaccine of interest
116. Mombelli M, Rettby N, Perreau M, Pascual M, Pantaleo G, Manuel O. Immunogenicity and safety of double versus standard dose of the seasonal influenza vaccine in solid-organ transplant recipients: A randomized controlled trial. *Vaccine*. 2018;36(41):6163-9.	No vaccine of interest
117. Munoz FM, Jackson LA, Swamy GK, Edwards KM, Frey SE, Stephens I, et al. Safety and immunogenicity of seasonal trivalent inactivated influenza vaccines in pregnant women. *Vaccine*. 2018;36(52):8054-61.	Not relevant comparator
118. Munoz FM, Patel SM, Jackson LA, Swamy GK, Edwards KM, Frey SE, et al. Safety and immunogenicity of three seasonal inactivated influenza vaccines among pregnant women and antibody persistence in their infants. *Vaccine*. 2020;38(33):5355-63.	Not relevant comparator

119. Nolan T, Lee MS, Cordova JM, et al. Safety and immunogenicity of a live-attenuated influenza vaccine blended and filled at two manufacturing facilities. *Vaccine.* 2003;21(11-12):1224-1231.	No relevant comparator
120. Nougarede N, Bisceglia H, Rozières A, Goujon C, Boudet F, Laurent P, et al. Nine µg intradermal influenza vaccine and 15 µg intramuscular influenza vaccine induce similar cellular and humoral immune responses in adults. *Human vaccines & immunotherapeutics*. 2014;10(9):2713-20.	No relevant outcome
121. Nunes MC, Cutland CL, Moultrie A, Jones S, Ortiz JR, Neuzil KM, et al. Immunogenicity and safety of different dosing schedules of trivalent inactivated influenza vaccine in pregnant women with HIV: a randomised controlled trial. *The lancet HIV*. 2020;7(2):e91-e103.	No vaccine of interest
122. Oh CE, Choi UY, Eun BW, Lee TJ, Kim KH, Kim DH, et al. A Randomized, Double-blind, Active-controlled Clinical Trial of a Cell Culture-derived Inactivated Trivalent Influenza Vaccine (NBP607) in Healthy Children 6 Months Through 18 Years of Age. *Pediatric infectious disease journal*. 2018;37(6):605-11.	No relevant comparator
123. Ojeda J, Arredondo JL, Salcedo P, Paredes-Paredes M, Dupuy M, Petit C, et al. Immunogenicity and safety of a multi-dose quadrivalent inactivated influenza vaccine in individuals aged 6 months to 17 years: a randomized phase III trial. *Human vaccines & immunotherapeutics*. 2020;16(6):1380-4.	No relevant comparator
124. Omer SB, Clark DR, Aqil AR, Tapia MD, Nunes MC, Kozuki N, et al. Maternal Influenza Immunization and Prevention of Severe Clinical Pneumonia in Young Infants: Analysis of Randomized Controlled Trials Conducted in Nepal, Mali and South Africa. *The Pediatric infectious disease journal.* 2018;37(5):436-40.	No vaccine of interest
125. Otten G, Matassa V, Ciarlet M, Leav B. A phase 1, randomized, observer blind, antigen and adjuvant dosage finding study to evaluate the safety and immunogenicity of an adjuvanted, trivalent subunit influenza vaccine in elderly subjects ≥65 years of age. *Open forum infectious diseases*. 2018;5:S747-.<br>Otten G, Matassa V, Ciarlet M, Leav B. A phase 1, randomized, observer blind, antigen and adjuvant dosage finding clinical trial to evaluate the safety and immunogenicity of an adjuvanted, trivalent subunit influenza vaccine in adults≥65 years of age. *Vaccine*. 2020;38(3):578-87.	No vaccine of interest
126. Pan SC, Hsieh SM, Lin CF, Hsu YS, Chang M, Chang SC. A randomized, double-blind, controlled clinical trial to evaluate the safety and immunogenicity of an intranasally administered trivalent inactivated influenza vaccine with adjuvant LTh(αK): A phase I study. *Vaccine*. 2019;37(14):1994-2003.	No vaccine of interest
127. Patel SS, Bizjajeva S, Heijnen E, Oberye J. MF59-adjuvanted seasonal trivalent inactivated influenza vaccine: Safety and immunogenicity in young children at risk of influenza complications. *International journal of infectious diseases : IJID : official publication of the International Society for Infectious Diseases*. 2019;85s:S18-s25.	Secondary analysis of already included studies
128. Phrommintikul A, Kuanprasert S, Wongcharoen W, Kanjanavanit R, Chaiwarith R, Sukonthasarn A. Influenza vaccination reduces cardiovascular events in patients with acute coronary syndrome. *European heart journal.* Jul 2011;32(14):1730-1735.	No relevant outcome
129. Pillet S, Couillard J, Trépanier S, Poulin JF, Yassine-Diab B, Guy B, et al. Immunogenicity and safety of a quadrivalent plant-derived virus like particle influenza vaccine candidate-Two randomized Phase II clinical trials in 18 to 49 and ≥50 years old adults. *PloS one*. 2019;14(6):e0216533.	No vaccine of interest
130. Pleguezuelos O, Dille J, de Groen S, Oftung F, Niesters HGM, Islam MA, et al. Immunogenicity, Safety, and Efficacy of a Standalone Universal Influenza Vaccine, FLU-v, in Healthy Adults: a Randomized Clinical Trial. *Annals of internal medicine*. 2020;172(7):453-62.<br>Pleguezuelos O, James E, Fernandez A, Lopes V, Rosas LA, Cervantes-Medina A, et al. Efficacy of FLU-v, a broad-spectrum influenza vaccine, in a randomized phase IIb human influenza challenge study. *NPJ vaccines*. 2020;5(1).	No vaccine of interest
131. Pleguezuelos O, Robinson S, Stoloff GA, Caparros-Wanderley W. Synthetic Influenza vaccine (FLU-v) stimulates cell mediated immunity in a double-blind, randomised, placebo-controlled Phase I trial. *Vaccine.* Jun 29 2012;30(31):4655-4660.	No vaccine of interest
132. Powers DC, Fries LF, Murphy BR, Thumar B, Clements ML. In elderly persons live attenuated influenza A virus vaccines do not offer an advantage over inactivated virus vaccine in inducing serum or secretory antibodies or local immunologic memory. *Journal of clinical microbiology.* Mar 1991;29(3):498-505.	No relevant outcome
133. Ramanathan RK, Potter DM, Belani CP, et al. Randomized trial of influenza vaccine with granulocyte-macrophage colony-stimulating factor or placebo in cancer patients. *Journal of clinical oncology: official journal of the American Society of Clinical Oncology.* Nov 1 2002;20(21):4313-4318.	No relevant outcome
134. Robertson CA, Mercer M, Selmani A, Klein NP, Jeanfreau R, Greenberg DP. Safety and Immunogenicity of a Full-dose, Split-virion, Inactivated, Quadrivalent Influenza Vaccine in Healthy Children 6-35 Months of Age: A Randomized Controlled Clinical Trial. *The Pediatric infectious disease journal.* 2019;38(3):323-8.	No vaccine of interest
135. Rudenko LG, Lonskaya NI, Klimov AI, Vasilieva RI, Ramirez A. Clinical and epidemiological evaluation of a live, cold-adapted influenza vaccine for 3-14-year-olds. *Bulletin of the world health organization.* 1996;74(1):77-84.	No vaccine of interest
136. Rudenko LG, Ramirez A, Barro M, et al. The inoculation properties of live recombinant influenza vaccine types A and B used separately and jointly in children 3 to 14. *Voprosy virusologii.* 1991;36(6):472-474.	No parallel RCT

137. Rudenko LG, Slepushkin AN, Monto AS, et al. Efficacy of live attenuated and inactivated influenza vaccines in schoolchildren and their unvaccinated contacts in Novgorod, Russia. *Journal of Infectious Diseases*. 1993;168(4):881-887.	No parallel RCT
138. Sanchez L, Matsuoka O, Inoue S, Inoue T, Meng Y, Nakama T, et al. Immunogenicity and safety of high-dose quadrivalent influenza vaccine in Japanese adults ≥65 years of age: a randomized controlled clinical trial. *Human vaccines & immunotherapeutics*. 2020;16(4):858-66.	No vaccine of interest
139. Sarker M, Branche A, Peasley M, Topham D. Comparison of antibody responses to vaccination with a pure hemagglutinin influenza vaccine (RHA) and licensed subvirion influenza vaccine made in eggs or cell culture in adults 60 years and older. *Open forum infectious diseases*. 2019;6:S963-.	No relevant outcome
140. Sarsenbayeva G, Issagulov T, Kassenov M, Abitay R, Orynbayev M, Stukova M, et al. Safety and immunogenicity of trivalent inactivated influenza vaccine in adults 60 years of age and older: a phase II, a randomized, comparative trial in Kazakhstan. *Human vaccines & immunotherapeutics*. 2020;16(8):1791-7.	No relevant comparator
141. Sarsenbayeva G, Volgin Y, Kassenov M, et al. Immunogenicity and safety of a novel seasonal influenza preservative-free vaccine manufactured in Kazakhstan: results of a randomized, comparative, phase II clinical trial in adults. *Human vaccines and immunotherapeutics.* 2017:1-6.	No relevant comparator
142. Schaad UB, Bühlmann U, Burger R, et al. Comparison of immunogenicity and safety of a virosome influenza vaccine with those of a subunit influenza vaccine in pediatric patients with cystic fibrosis. *Antimicrobial agents and chemotherapy.* 2000;44(5):1163-1167.	No relevant comparator
143. Şener M, Gürsel G, Türktaş H. Effects of inactivated influenza virus vaccination on bronchial reactivity symptom scores and peak expiratory flow variability in patients with asthma. *Journal of Asthma.* 1999;36(2):165-169.	No parallel RCT
144. Seo YB, Baek JH, Lee J, et al. Long-Term Immunogenicity and Safety of a Conventional Influenza Vaccine in Patients with Type 2 Diabetes. *Clinical and vaccine immunology.* 2015;22(11):1160-1165.	No parallel RCT
145. Simões EAF, Nunes MC, Carosone-Link P, Madimabe R, Ortiz JR, Neuzil KM, et al. Trivalent influenza vaccination randomized control trial of pregnant women and adverse fetal outcomes. *Vaccine*. 2019;37(36):5397-403.	Secondary analysis of already included studies
146. Skowronski DM, Hottes TS, Chong M, et al. Randomized controlled trial of dose response to influenza vaccine in children aged 6 to 23 months. *Pediatrics.* 2011;128(2): e276-289.	No relevant comparator
147. Slepushkin AN, Obrosova-Serova NP, Burtseva EI, et al. [A comparative study of the inoculation properties of live recombinant and inactivated influenza vaccines made from strain A/Philippines/2/82 (H3N2) in 8- to 15-year-old children]. *Voprosy virusologii.* Sep-Oct 1991;36(5):372-374.	No vaccine of interest
148. Song JY, Cheong HJ, Hyun HJ, Seo YB, Lee J, Wie SH, et al. Immunogenicity and safety of a 13-valent pneumococcal conjugate vaccine and an MF59-adjuvanted influenza vaccine after concomitant vaccination in ⩾60-year-old adults. Vaccine. 2017;35(2):313-20.	No relevant outcome
149. Song JY, Cheong HJ, Noh JY, Seo YB, Choi WS, Cho GJ, et al. Long-term and cross-reactive immunogenicity of inactivated trivalent influenza vaccine in the elderly: MF59-adjuvanted vaccine versus unadjuvanted vaccine. *Journal of medical virology*. 2013;85(9):1591-7.	No relevant outcome
150. Song JY, Cheong HJ, Noh JY, Yang TU, Seo YB, Hong KW, et al. Long-term immunogenicity of the influenza vaccine at reduced intradermal and full intramuscular doses among healthy young adults. *Clin Exp Vaccine Res*. 2013;2(2):115-9.	No vaccine of interest
151. Song JY, Cheong HJ, Tsai TF, Chang HA, Choi MJ, Jeon JH, et al. Immunogenicity and safety of concomitant MF59-adjuvanted influenza vaccine and 23-valent pneumococcal polysaccharide vaccine administration in older adults. *Vaccine*. 2015;33(36):4647-52.	No relevant outcome
152. Song JY, Cheong HJ, Woo HJ, et al. Immunogenicity and safety of trivalent inactivated influenza vaccine: a randomized, double-blind, multi-center, phase 3 clinical trial in a vaccine-limited country. *Journal of korean medical science.* 2011;26(2):191-195.	No relevant comparator
153. Statler VA, Albano FR, Airey J, Sawlwin DC, Graves Jones A, Matassa V, et al. Immunogenicity and safety of a quadrivalent inactivated influenza vaccine in children 6-59 months of age: A phase 3, randomized, noninferiority study. *Vaccine*. 2019;37(2):343-51.	No relevant comparator
154. Sullender WM, Fowler KB, Gupta V, Krishnan A, Ram Purakayastha D, Srungaram Vln R, et al. Efficacy of inactivated trivalent influenza vaccine in rural India: a 3-year cluster-randomised controlled trial. *The Lancet Global health.* 2019;7(7):e940-e50.	No parallel RCT
155. Tackenberg B, Schneider M, Blaes F, et al. Acetylcholine Receptor Antibody Titers and Clinical Course after Influenza Vaccination in Patients with Myasthenia Gravis: A Double-Blind Randomized Controlled Trial (ProPATIent-Trial). *EBioMedicine.* Feb 2018; 28:143-150.	No relevant outcome
156. Tasker SA, O'Brien WA, Treanor JJ, et al. Effects of influenza vaccination in HIV-infected adults: a double-blind, placebo-controlled trial. *Vaccine.* May-Jun 1998;16(9-10):1039-1042.	No relevant outcome

157. Teh B, Leung V, Mordant F, Sullivan S, Joyce T, Harrison S, et al. A randomised trial of two 2-dose influenza vaccination strategies for patients following autologous haematopoietic stem cell transplantation. *Hemasphere*. 2020;4:116-7.	No vaccine of interest
158. Treanor JJ, Mattison HR, Dumyati G, et al. Protective efficacy of combined live intranasal and inactivated influenza A virus vaccines in the elderly. *Annals of internal medicine.* 1992;117(8):625-633.	No vaccine of interest
159. Useche M, Agnihotram R, Bernatsky S, Ward B, Colmegna I. The high dose influenza vaccine increases immune protection in both adults and elderly seropositive RA patients. *Arthritis and Rheumatology.* 2019;71:3680-1.	No relevant outcome
160. Van Damme P, Oosterhuis-Kafeja F, Van der Wielen M, Almagor Y, Sharon O, Levin Y. Safety and efficacy of a novel microneedle device for dose sparing intradermal influenza vaccination in healthy adults. *Vaccine.* Jan 14 2009;27(3):454-459.	No vaccine of interest
161. Vesikari T, Kirstein J, Devota Go G, Leav B, Ruzycky ME, Isakov L, et al. Efficacy, immunogenicity, and safety evaluation of an MF59-adjuvanted quadrivalent influenza virus vaccine compared with non-adjuvanted influenza vaccine in children: a multicentre, randomised controlled, observer-blinded, phase 3 trial. T*he Lancet Respiratory medicine.* 2018;6(5):345-56.	No relevant comparator
162. Vesikari T, Richardus JH, Berglund J, et al. Immunogenicity and Safety of a Trivalent Inactivated Influenza Vaccine in Children 6 Months to 17 Years of Age, Previously Vaccinated with an AS03-Adjuvanted A(H1N1) Pdm09 Vaccine: two Open-label, Randomized Trials. *Pediatric infectious disease journal.* 2015;34(7):774-782.	No relevant comparator

References of included studies

1. Abadoğlu Ö, Mungan D, Paşaoglu G, Çelík G, Misirligil Z. Influenza vaccination in patients with asthma: Effect on the frequency of upper respiratory tract infections and exacerbations. *Journal of Asthma.* 2004;41(3):279-283.

2. Allsup S, Haycox A, Regan M, Gosney M. Is influenza vaccination cost effective for healthy people between ages 65 and 74 years? A randomised controlled trial. *Vaccine.* Dec 16 2004;23(5):639-645.
   Allsup SJ, Gosney M, Regan M, Haycox A, Fear S, Johnstone FC. Side effects of influenza vaccination in healthy older people: a randomised single-blind placebo-controlled trial. *Gerontology*. 2001;47(6):311-314.

3. Anh DD, Thiem VD, Anh NTH, et al. Randomized safety and immunogenicity trial of a seasonal trivalent inactivated split virion influenza vaccine (IVACFLU-S) in healthy young Vietnamese adults. *Vaccine.* Oct 26 2016;34(45):5457-5462.

4. Ansaldi F, Orsi A, Florentiis D, et al. Head-to-head comparison of an intradermal and a virosome influenza vaccine in patients over the age of 60: evaluation of immunogenicity, cross-protection, safety and tolerability. *Human vaccines & immunotherapeutics.* 2013;9(3):591-598.

5. Ansaldi F, Valle L, De Florentiis D, et al. Phase 4 randomized trial of intradermal low-antigen-content inactivated influenza vaccine vs. standard-dose intramuscular vaccine in HIV-1-infected adults. *Human Vaccines and Immunotherapeutics.* 2012;8(8):1048-1052.

6. Arnou R, Eavis P, Pardo JR, Ambrozaitis A, Kazek MP, Weber F. Immunogenicity, large scale safety and lot consistency of an intradermal influenza vaccine in adults aged 18-60 years: randomized, controlled, phase III trial. *Human vaccines.* 2010;6(4):346-354.

7. Arnou R, Icardi G, De Decker M, et al. Intradermal influenza vaccine for older adults: a randomized controlled multicenter phase III study. *Vaccine.* Dec 9 2009;27(52):7304-7312.

8. Ashkenazi S, Vertruyen A, Arístegui J, et al. Superior relative efficacy of live attenuated influenza vaccine compared with inactivated influenza vaccine in young children with recurrent respiratory tract infections. *Pediatric infectious disease journal.* 2006;25(10):870-879.

9. Auewarakul P, Kositanont U, Sornsathapornkul P, Tothong P, Kanyok R, Thongcharoen P. Antibody responses after dose-sparing intradermal influenza vaccination. *Vaccine.* 2007;25(4):659-663.

10. Baldo V, Baldovin T, Floreani A, Carraro AM, Trivello R. MF59-adjuvanted influenza vaccine confers superior immunogenicity in adult subjects (18-60 years of age) with chronic diseases who are at risk of post-influenza complications. *Vaccine.* 2007;25(20):3955-3961.

11. Baluch A, Humar A, Eurich D, et al. Randomized controlled trial of high-dose intradermal versus standard-dose intramuscular influenza vaccine in organ transplant recipients. *American journal of transplantation.* 2013;13(4):1026-1033.

12. Barrett PN, Berezuk G, Fritsch S, et al. Efficacy, safety, and immunogenicity of a Vero-cell-culture-derived trivalent influenza vaccine: a multicentre, double-blind, randomised, placebo-controlled trial. *Lancet (london, england).* 2011;377(9767):751-759.

13. Bart S, Cannon K, Herrington D, et al. Immunogenicity and safety of a cell culture-based quadrivalent influenza vaccine in adults: a Phase III, double-blind, multicenter, randomized, non-inferiority study. *Human vaccines & immunotherapeutics.* 2016;12(9):2278-2288.

14. Baxter R, Patriarca PA, Ensor K, Izikson R, Goldenthal KL, Cox MM. Evaluation of the safety, reactogenicity and immunogenicity of FluBlok trivalent recombinant baculovirus-expressed hemagglutinin influenza vaccine administered intramuscularly to healthy adults 50-64 years of age. *Vaccine.* 2011;29(12):2272-2278.

15. Belongia EA, Levine MZ, Olaiya O, Gross FL, King JP, Flannery B, et al. Clinical trial to assess immunogenicity of high-dose, adjuvanted, and recombinant influenza vaccines against cell-grown A(H3N2) viruses in adults 65 to 74 years, 2017-2018. *Vaccine.* 2020;38(15):3121-8.

16. Belshe RB, Edwards KM, Vesikari T, et al. Live attenuated versus inactivated influenza vaccine in infants and young children. *The New England journal of medicine.* Feb 15 2007;356(7):685-696.

17. Belshe RB, Gruber WC, Mendelman PM, et al. Efficacy of vaccination with live attenuated, cold-adapted, trivalent, intranasal influenza virus vaccine against a variant (A/Sydney) not contained in the vaccine. *Journal of pediatrics.* 2000;136(2):168-175.

18. Belshe RB, Mendelman PM, Treanor J, et al. The efficacy of live attenuated, cold-adapted, trivalent, intranasal influenzavirus vaccine in children. *New England Journal of Medicine.* 1998;338(20):1405-1412.
    Longini IM, Halloran ME, Nizam A, et al. Estimation of the efficacy of live, attenuated influenza vaccine from a two-year, multi-center vaccine trial: implications for influenza epidemic control. *Vaccine*. Mar 17 2000;18(18):1902-1909.
    Piedra PA, Yan L, Kotloff K, et al. Safety of the trivalent, cold-adapted influenza vaccine in preschool-aged children. *Pediatrics*. Oct 2002;110(4):662-672.

19. Belshe RB, Newman FK, Wilkins K, et al. Comparative immunogenicity of trivalent influenza vaccine administered by intradermal or intramuscular route in healthy adults. *Vaccine.* 2007;25(37-38):6755-6763.

20. Belshe RB, Swierkosz EM, Anderson EL, Newman FK, Nugent SL, Maassab HF. Immunization of infants and young children with live attenuated trivalent cold-recombinant influenza A H1N1, H3N2, and B vaccine. *Journal of infectious diseases.* 1992;165(4):727-732.

21. Ben-Yehuda A, Joseph A, Barenholz Y, et al. Immunogenicity and safety of a novel IL-2-supplemented liposomal influenza vaccine (INFLUSOME-VAC) in nursing-home residents. *Vaccine.* 2003;21(23):3169-3178.

22. Ben-Yehuda A, Joseph A, Zeira E, et al. Immunogenicity and safety of a novel liposomal influenza subunit vaccine (INFLUSOME-VAC) in young adults. *Journal of medical virology.* 2003;69(4):560-567.

23. Beran J, Ambrozaitis A, Laiskonis A, et al. Intradermal influenza vaccination of healthy adults using a new microinjection system: a 3-year randomised controlled safety and immunogenicity trial. *BMC medicine.* Apr 2 2009;7:13.

24. Beran J, Peeters M, Dewé W, Raupachová J, Hobzová L, Devaster JM. Immunogenicity and safety of quadrivalent versus trivalent inactivated influenza vaccine: a randomized, controlled trial in adults. *BMC infectious diseases.* 2013; 13:224.

25. Beran J, Vesikari T, Wertzova V, et al. Efficacy of inactivated split-virus influenza vaccine against culture-confirmed influenza in healthy adults: a prospective, randomized, placebo-controlled trial. *The Journal of infectious diseases.* Dec 15 2009;200(12):1861-1869.

26. Beran J, Wertzova V, Honegr K, et al. Challenge of conducting a placebo-controlled randomized efficacy study for influenza vaccine in a season with low attack rate and a mismatched vaccine B strain: a concrete example. *BMC infectious diseases.* Jan 17 2009;9:2.

27. Bergen R, Black S, Shinefield H, et al. Safety of cold-adapted live attenuated influenza vaccine in a large cohort of children and adolescents. *The Pediatric infectious disease journal.* Feb 2004;23(2):138-144.
Baxter RP, Lewis N, Fireman B, Hansen J, Klein NP, Ortiz JR. Live Attenuated Influenza Vaccination Before 3 Years of Age and Subsequent Development of Asthma: A 14-year Follow-up Study. *The Pediatric infectious disease journal*. May 2018;37(5):383-386.

28. Block SL, Falloon J, Hirschfield JA, et al. Immunogenicity and safety of a quadrivalent live attenuated influenza vaccine in children. *The Pediatric infectious disease journal.* Jul 2012;31(7):745-751.

29. Block SL, Yi T, Sheldon E, Dubovsky F, Falloon J. A randomized, double-blind noninferiority study of quadrivalent live attenuated influenza vaccine in adults. *Vaccine.* Nov 21 2011;29(50):9391-9397.

30. Boonnak K, Dhitavat J, Thantamnu N, et al. Immune responses to intradermal and intramuscular inactivated influenza vaccine among older age group. *Vaccine.* 2017;35:7339-7346.(no pagination).

31. Bracco Neto H, Farhat CK, Tregnaghi MW, et al. Efficacy and safety of 1 and 2 doses of live attenuated influenza vaccine in vaccine-naive children. *The Pediatric infectious disease journal*. May 2009;28(5):365-371.

32. Brady RC, Jackson LA, Frey SE, Shane AL, Walter EB, Swamy GK, et al. Randomized trial comparing the safety and antibody responses to live attenuated versus inactivated influenza vaccine when administered to breastfeeding women. *Vaccine*. 2018;36(31):4663-71.

33. Bridges CB, Thompson WW, Meltzer MI, et al. Effectiveness and cost-benefit of influenza vaccination of healthy working adults: A randomized controlled trial. *Jama.* Oct 4 2000;284(13):1655-1663.

34. Brooks WA, Zaman K, Lewis KD, et al. Efficacy of a Russian-backbone live attenuated influenza vaccine among young children in Bangladesh: a randomised, double-blind, placebo-controlled trial. *The Lancet. Global health.* Dec 2016;4(12):e946-e954.
Rotrosen E, Zaman K, Feser J, et al. Influenza among Young Children in Bangladesh: Clinical Characteristics and Outcomes from a Randomized Clinical Trial. *Clinical Infectious Diseases*. 2017;65(11):1914-1920.

35. Bueving HJ, Bernsen RM, de Jongste JC, et al. Influenza vaccination in children with asthma: randomized doubleblind placebo-controlled trial. *American Journal of Respiratory & Critical Care Medicine*. 2004;169(4):488–93.
Bueving HJ, Bernsen RM, de Jongste JC, et al. Does influenza vaccination exacerbate asthma in children? *Vaccine*. Nov 15 2004;23(1):91-96.
Bueving HJ, van der Wouden JC, Raat H, et al. Influenza vaccination in asthmatic children: effects on quality of life and symptoms. *The European respiratory journal*. Dec 2004;24(6):925-931.

36. Cadorna-Carlos JB, Nolan T, Borja-Tabora CF, et al. Safety, immunogenicity, and lot-to-lot consistency of a quadrivalent inactivated influenza vaccine in children, adolescents, and adults: A randomized, controlled, phase III trial. *Vaccine.* May 15 2015;33(21):2485-2492.

37. Caldera F, Hillman L, Saha S, Wald A, Grimes I, Zhang Y, et al. Immunogenicity of High Dose Influenza Vaccine for Patients with Inflammatory Bowel Disease on Anti-TNF Monotherapy: A Randomized Clinical Trial. *Inflammatory bowel diseases*. 2020;26(4):593-602.

38. Carr S, Allison KJ, Van De Velde LA, et al. Safety and immunogenicity of live attenuated and inactivated influenza vaccines in children with cancer. *The Journal of infectious diseases.* Nov 15 2011;204(10):1475-1482.

39. Chan TC, Hung IFN, Chan KH, et al. Immunogenicity and safety of intradermal trivalent influenza vaccination in nursing home older adults: A randomized controlled trial. *Journal of the American Medical Directors Association.* 2014;15(8):607.e605-607.e612.

40. Chang LJ, Meng Y, Janosczyk H, Landolfi V, Talbot HK. Safety and immunogenicity of high-dose quadrivalent influenza vaccine in adults ≥65 years of age: A phase 3 randomized clinical trial. *Vaccine*. 2019;37(39):5825-34.

41. Chi RC, Rock MT, Neuzil KM. Immunogenicity and safety of intradermal influenza vaccination in healthy older adults. *Clinical infectious diseases: an official publication of the Infectious Diseases Society of America.* May 15 2010;50(10):1331-1338.

42. Chiu SS, Chan KH, Tu W, Lau YL, Peiris JS. Immunogenicity and safety of intradermal versus intramuscular route of influenza immunization in infants less than 6 months of age: a randomized controlled trial. *Vaccine.* Jul 30 2009;27(35):4834-4839.

43. Chiu SS, Peiris JSM, Chan KH, Wong WHS, Yu LL. Immunogenicity and safety of intradermal influenza immunization at a reduced dose in healthy children. Pediatrics. 2007;119(6):1076-1082

44. Choi WS, Noh JY, Lee J, Choi JY, Lee JS, Kim MS, Kim HS, Bang J, Lavis N, Kim WJ. Immunogenicity and safety of a split-virion quadrivalent influenza vaccine in adults 18 60 years of age in the Republic of Korea. *Human vaccines and immunotherapeutics*. 2018;14(3):587-592.

45. Choi WS, Noh JY, Song JY, Cheong HJ, Wie SH, Lee JS, et al. Immunogenicity and safety of a cell culture-derived inactivated quadrivalent influenza vaccine (NBP607-QIV): A randomized, double-blind, multi-center, phase III clinical trial in adults and elderly subjects. *Human Vaccines and Immunotherapeutics*. 2017;13(7):1653-1660.

46. Chu K, Xu K, Tang R, Tian X, Hu J, Yang T, et al. Immunogenicity and safety of an inactivated quadrivalent influenza vaccine: A randomized, double-blind, controlled phase III study in healthy population aged ≥3 years. *Vaccine*. 2020;38(37):5940-6.

47. Chuaychoo B, Kositanont U, Niyomthong P, Rittayamai N, Srisuma S, Rattanasaengloet K, et al. Comparison of immunogenicity between intradermal and intramuscular injections of repeated annual identical influenza virus strains post-pandemic (2011-2012) in COPD patients. *Human Vaccines and Immunotherapeutics.* 2020;16(6):1371-9.

48. Claeys C, Zaman K, Dbaibo G, Li P, Izu A, Kosalaraksa P, et al. Prevention of vaccine-matched and mismatched influenza in children aged 6-35 months: a multinational randomised trial across five influenza seasons. *The Lancet Child & adolescent health*. 2018;2(5):338-49.

49. Colmegna I, Useche ML, Rodriguez K, McCormack D, Alfonso G, Patel A, et al. Immunogenicity and safety of high-dose versus standard-dose inactivated influenza vaccine in rheumatoid arthritis patients: a randomised, double-blind, active-comparator trial. *The lancet rheumatology*. 2020;2(1):e14-e23.

50. Colombo C, Argiolas L, Vecchia C, Negri E, Meloni G, Meloni T. Influenza vaccine in healthy preschool children. *Revue d'epidemiologie ET de sante publique.* 2001;49(2):157-162.

51. Couch RB, Winokur P, Brady R, et al. Safety and immunogenicity of a high dosage trivalent influenza vaccine among elderly subjects. *Vaccine.* 2007;25(44):7656-7663.
Cate TR, Rayford Y, Nino D, et al. A high dosage influenza vaccine induced significantly more neuraminidase antibody than standard vaccine among elderly subjects. *Vaccine*. Feb 25 2010;28(9):2076-2079.

52. Cowling BJ, Ng S, Ma ESK, et al. Protective efficacy of seasonal influenza vaccination against seasonal and pandemic influenza virus infection during 2009 in Hong Kong. *Clinical Infectious Diseases.* 2010;51(12):1370-1379.
Cowling BJ, Fang VJ, Nishiura H, et al. Increased risk of noninfluenza respiratory virus infections associated with receipt of inactivated influenza vaccine. *Clinical infectious diseases*. 2012;54(12):1778-1783.

53. Cowling BJ, Perera RA, Fang VJ, et al. Incidence of influenza virus infections in children in Hong Kong in a 3-year randomized placebo-controlled vaccine study, 2009-2012. *Clinical infectious diseases: an official publication of the Infectious Diseases Society of America.* Aug 15 2014;59(4):517-524.

54. Cowling BJ, Perera RAPM, Valkenburg SA, Leung NHL, Iuliano AD, Tam YH, et al. Comparative immunogenicity of several enhanced influenza vaccine options for older adults: A randomized, controlled trial. *Clinical Infectious Diseases*. 2020;71(7):1704-14.
Cowling BJ, Thompson MG, Ng TWY, Fang VJ, Perera R, Leung NHL, et al. Comparative Reactogenicity of Enhanced Influenza Vaccines in Older Adults. *The Journal of infectious diseases*. 2020;222(8):1383-91.

55. Cruz-Valdez A, Valdez-Zapata G, Patel SS, et al. MF59-adjuvanted influenza vaccine (FLUAD) elicits higher immune responses than a non-adjuvanted influenza vaccine (Fluzone): a randomized, multicenter, Phase III pediatric trial in Mexico. *Human vaccines and immunotherapeutics.* 20172018;14(2):386-95.:1-10.

56. de Bruijn I, Meyer I, Gerez L, Nauta J, Giezeman K, Palache B. Antibody induction by virosomal, MF59-adjuvanted, or conventional influenza vaccines in the elderly. *Vaccine.* Dec 21 2007;26(1):119-127.

57. de Bruijn IA, Nauta J, Gerez L, Palache AM. The virosomal influenza vaccine Invivac: immunogenicity and tolerability compared to an adjuvanted influenza vaccine (Fluad in elderly subjects. *Vaccine.* Nov 10 2006;24(44-46):6629-6631.

58. De Donato S, Granoff D, Minutello M, et al. Safety and immunogenicity of MF59-adjuvanted influenza vaccine in the elderly. *Vaccine.* Aug 6 1999;17(23-24):3094-3101.

59. De Villiers PJ, Steele AD, Hiemstra LA, et al. Efficacy and safety of a live attenuated influenza vaccine in adults 60 years of age and older. *Vaccine.* Dec 10 2009;28(1):228-234.

60. Della Cioppa G, Vesikari T, Sokal E, Lindert K, Nicolay U. Trivalent and quadrivalent MF59((R))-adjuvanted influenza vaccine in young children: a dose- and schedule-finding study. *Vaccine.* Nov 3 2011;29(47):8696-8704.

61. Desheva IA, Danini GV, Grigor'eva EP, et al. The investigation of the safety, genetic stability and immunogenicity of live influenza vaccine for adults in vaccination of 3-6 years old children. *Voprosy virusologii.* 2002;47(4):21-24.

62. Diallo A, Victor JC, Feser J, Ortiz JR, Kanesa-Thasan N, Ndiaye M, et al. Immunogenicity and safety of MF59-adjuvanted and full-dose unadjuvanted trivalent inactivated influenza vaccines among vaccine-naïve children in a randomized clinical trial in rural Senegal. *Vaccine*. 2018;36(43):6424-32.

63. DiazGranados CA, Dunning AJ, Jordanov E, Landolfi V, Denis M, Talbot HK. High-dose trivalent influenza vaccine compared to standard dose vaccine in elderly adults: safety, immunogenicity and relative efficacy during the 2009-2010 season. *Vaccine.* Jan 30 2013;31(6):861-866.

64. DiazGranados CA, Dunning AJ, Kimmel M, et al. Efficacy of high-dose versus standard-dose influenza vaccine in older adults. *The New England journal of medicine.* Aug 14 2014;371(7):635-645.
DiazGranados CA, Dunninga AJ, Robertson CA, Talbot HK, Landolfi VA, Greenberg DP. Efficacy and immunogenicity of high-dose influenza vaccine in olderadults by age, comorbidities, and frailty. *Vaccine*. 2015;33(36):4565–4571.

65. DiazGranados CA, Saway W, Gouaux J, et al. Safety and immunogenicity of high-dose trivalent inactivated influenza vaccine in adults 50-64 years of age. *Vaccine.* Dec 16 2015;33(51):7188-7193.

66. Domachowske JB, Pankow-Culot H, Bautista M, et al. A randomized trial of candidate inactivated quadrivalent influenza vaccine versus trivalent influenza vaccines in children aged 3-17 years. *The Journal of infectious diseases.* Jun 15 2013;207(12):1878-1887.

67. Dunkle LM, Izikson R, Patriarca P, et al. Efficacy of recombinant influenza vaccine in adults 50 years of age or older. *New England Journal of Medicine.* 2017;376(25):2427-2436.

68. Dunkle LM, Izikson R, Patriarca PA, Goldenthal KL, Cox M, Treanor JJ. Safety and Immunogenicity of a Recombinant Influenza Vaccine: A Randomized Trial. *Pediatrics*. 2018;141(5).

69. Dunkle LM, Izikson R, Patriarca PA, Goldenthal KL, Muse D, Cox MMJ. Randomized comparison of immunogenicity and safety of quadrivalent recombinant versus inactivated influenza vaccine in healthy adults 18-49 years of age. *Journal of Infectious Diseases.* 2017;216(10):1219-1226.

70. Durando P, Fenoglio D, Boschini A, et al. Safety and immunogenicity of two influenza virus subunit vaccines, with or without MF59 adjuvant, administered to human immunodeficiency virus type 1-seropositive and -seronegative adults. *Clinical and vaccine immunology : CVI.* Feb 2008;15(2):253-259.

71. Englund JA, Walter E, Black S, et al. Safety and immunogenicity of trivalent inactivated influenza vaccine in infants: a randomized double-blind placebo-controlled study. *The Pediatric infectious disease journal.* Feb 2010;29(2):105-110.

72. Esposito S, Cecinati V, Scicchitano B, et al. Impact of influenza-like illness and effectiveness of influenza vaccination in oncohematological children who have completed cancer therapy. *Vaccine.* Feb 10 2010;28(6):1558-1565.

73. Esposito S, Daleno C, Picciolli I, et al. Immunogenicity and safety of intradermal influenza vaccine in children. *Vaccine.* Oct 13 2011;29(44):7606-7610.

74. Esposito S, Mastrolia MV, Ghio L, et al. Influenza immunization in hemodialyzed or kidney transplanted adolescents and young adults. *Expert review of vaccines.* Aug 2014;13(8):1059-1066.

75. Essink B, Fierro C, Rosen J, Figueroa AL, Zhang B, Verhoeven C, et al. Immunogenicity and safety of MF59-adjuvanted quadrivalent influenza vaccine versus standard and alternate B strain MF59-adjuvanted trivalent influenza vaccines in older adults. *Vaccine.* 2020;38(2):242-50.

76. Evison J, Farese S, Seitz M, Uehlinger DE, Furrer H, Muhlemann K. Randomized, double-blind comparative trial of subunit and virosomal influenza vaccines for immunocompromised patients. *Clinical infectious diseases: an official publication of the Infectious Diseases Society of America.* May 15 2009;48(10):1402-1412.

77. Falsey AR, Treanor JJ, Tornieporth N, Capellan J, Gorse GJ. Randomized, double-blind controlled phase 3 trial comparing the immunogenicity of high-dose and standard-dose influenza vaccine in adults 65 years of age and older. *The Journal of infectious diseases.* Jul 15 2009;200(2):172-180.

78. Fayyaz Jahani F, Karramyyar M, Ahmadnezhad E. Inactivated-trivalent influenza vaccination in asthmatic under-5 children: A randomized double-blind placebo trial. *Archives of Disease in Childhood.* 2012;97:A516.

79. Fleming DM, Crovari P, Wahn U, et al. Comparison of the efficacy and safety of live attenuated cold-adapted influenza vaccine, trivalent, with trivalent inactivated influenza virus vaccine in children and adolescents with asthma. *The Pediatric infectious disease journal.* Oct 2006;25(10):860-869.

80. Forrest BD, Steele AD, Hiemstra L, Rappaport R, Ambrose CS, Gruber WC. A prospective, randomized, open-label trial comparing the safety and efficacy of trivalent live attenuated and inactivated influenza vaccines in adults 60 years of age and older. *Vaccine.* May 9 2011;29(20):3633-3639.

81. Frenck RW, Jr., Belshe R, Brady RC, et al. Comparison of the immunogenicity and safety of a split-virion, inactivated, trivalent influenza vaccine (Fluzone(R)) administered by intradermal and intramuscular route in healthy adults. *Vaccine.* Aug 5 2011;29(34):5666-5674.

82. Frey S, Poland G, Percell S, Podda A. Comparison of the safety, tolerability, and immunogenicity of a MF59-adjuvanted influenza vaccine and a non-adjuvanted influenza vaccine in non-elderly adults. *Vaccine.* Oct 1 2003;21(27-30):4234-4237.

83. Frey S, Vesikari T, Szymczakiewicz-Multanowska A, et al. Clinical efficacy of cell culture-derived and egg-derived inactivated subunit influenza vaccines in healthy adults. *Clinical infectious diseases: an official publication of the Infectious Diseases Society of America*. Nov 1 2010;51(9):997-1004.

84. Frey SE, Reyes M, Reynales H, et al. Comparison of the safety and immunogenicity of an MF59-adjuvanted with a non-adjuvanted seasonal influenza vaccine in elderly subjects. *Vaccine.* 2014;32(39):5027-5034.

85. Gabutti G, Guido M, Durando P, et al. Safety and immunogenicity of conventional subunit and MF59-adjuvanted influenza vaccines in human immunodeficiency virus-1-seropositive patients. *Journal of International Medical Research.* 2005;33(4):406-416.

86. Gasparini R, Pozzi T, Montomoli E, et al. Increased immunogenicity of the MF59-adjuvanted influenza vaccine compared to a conventional subunit vaccine in elderly subjects. *European journal of epidemiology.* 2001;17(2):135-140.

87. GiaQuinta S, Michaels MG, McCullers JA, et al. Randomized, double-blind comparison of standard-dose vs. high-dose trivalent inactivated influenza vaccine in pediatric solid organ transplant patients. *Pediatric transplantation.* Mar 2015;19(2):219-228.

88. Gorse GJ, Falsey AR, Fling JA, Poling TL, Strout CB, Tsang PH. Intradermally-administered influenza virus vaccine is safe and immunogenic in healthy adults 18-64 years of age. *Vaccine.* May 1 2013;31(19):2358-2365.

89. Gorse GJ, Falsey AR, Johnson CM, et al. Safety and immunogenicity of revaccination with reduced dose intradermal and standard dose intramuscular influenza vaccines in adults 18-64 years of age. *Vaccine.* Dec 5 2013;31(50):6034-6040.

90. Gorse GJ, Falsey AR, Ozol-Godfrey A, Landolfi V, Tsang PH. Safety and immunogenicity of a quadrivalent intradermal influenza vaccine in adults. *Vaccine.* Feb 25 2015;33(9):1151-1159.

91. Govaert Th ME, Dinant GJ, Aretz K, Masurel N, Sprenger MJW, Knottnerus JA. Adverse reactions to influenza vaccine in elderly people: Randomised double blind placebo controlled trial. *British Medical Journal.* 1993;307(6910):988-990.
Govaert Th ME, Thijs CTMCN, Masurel N, Sprenger MJW, Dinant GJ, Knottnerus JA. The efficacy of influenza vaccination in elderly individuals: A randomized double-blind placebo-controlled trial. Journal of the American Medical Association. 1994;272(21):1661-1665.

92. Greenberg DP, Robertson CA, Landolfi VA, Bhaumik A, Senders SD, Decker MD. Safety and immunogenicity of an inactivated quadrivalent influenza vaccine in children 6 months through 8 years of age. *Pediatric infectious disease journal.* 2014;33(6):630-636.

93. Greenberg DP, Robertson CA, Noss MJ, Blatter MM, Biedenbender R, Decker MD. Safety and immunogenicity of a quadrivalent inactivated influenza vaccine compared to licensed trivalent inactivated influenza vaccines in adults. *Vaccine.* 2013;31(5):770-776.

94. Greenberg DP, Robertson CA, Talbot HK, Decker MD. Safety and immunogenicity of a quadrivalent influenza vaccine in adults 65 y of age and older. *Human vaccines and immunotherapeutics.* 2017;13(9):2058-2064.

95. Hakim H, Allison KJ, Van de Velde LA, et al. Immunogenicity and safety of high-dose trivalent inactivated influenza vaccine compared to standard-dose vaccine in children and young adults with cancer or HIV infection. *Vaccine.* Jun 8 2016;34(27):3141-3148.

96. Halasa N, Englund JA, Nachman S, et al. Safety of live attenuated influenza vaccine in mild to moderately immunocompromised children with cancer. *Vaccine.* May 31 2011;29(24):4110-4115.

97. Halasa NB, Savani BN, Asokan I, et al. Randomized Double-Blind Study of the Safety and Immunogenicity of Standard-Dose Trivalent Inactivated Influenza Vaccine versus High-Dose Trivalent Inactivated Influenza Vaccine in Adult Hematopoietic Stem Cell Transplantation Patients. *Biology of blood and marrow transplantation: journal of the American Society for Blood and Marrow Transplantation.* Mar 2016;22(3):528-535.

98. Hartvickson R, Cruz M, Ervin J, et al. Non-inferiority of mammalian cell-derived quadrivalent subunit influenza virus vaccines compared to trivalent subunit influenza virus vaccines in healthy children: a phase III randomized, multicenter, double-blind clinical trial.

International journal of infectious diseases: IJID: official publication of the International Society for Infectious Diseases. Dec 2015;41:65-72.

99. Hoberman A, Greenberg DP, Paradise JL, et al. Effectiveness of inactivated influenza vaccine in preventing acute otitis media in young children: a randomized controlled trial. *Jama.* Sep 24 2003;290(12):1608-1616.

100. Hoft DF, Babusis E, Worku S, et al. Live and inactivated influenza vaccines induce similar humoral responses, but only live vaccines induce diverse T-cell responses in young children. *Journal of Infectious Diseases.* 2011;204(6):845-853.

101. Holland D, Booy R, Looze F, et al. Intradermal influenza vaccine administered using a new microinjection system produces superior immunogenicity in elderly adults: a randomized controlled trial. *Journal of infectious diseases.* 2008;198(5):650-658.

102. Hoon Han S, Hee Woo J, Weber F, Joo Kim W, Ran Peck K, Il Kim S. Immunogenicity and safety of Intanza ()/IDflu () intradermal influenza vaccine in South Korean adults: a multicenter, randomized trial. *Human vaccines.* 2013;9(9):1971-1977.

103. Hu Y, Shao M, Hu Y, Liang Q, Jia N, Chu K, et al. Immunogenicity and safety of an inactivated quadrivalent influenza vaccine: a randomized, double-blind, controlled phase III clinical trial in children aged 6-35 months in China. *Human vaccines & immunotherapeutics.* 2020;16(7):1691-8.

104. Hui SL, Chu LW, Peiris JSM, Chan KH, Chu D, Tsui W. Immune response to influenza vaccination in community-dwelling Chinese elderly persons. *Vaccine.* 2006;24(25):5371-5380.

105. Hung IF, Levin Y, To KK, Chan KH, Zhang AJ, Li P, et al. Dose sparing intradermal trivalent influenza (2010/2011) vaccination overcomes reduced immunogenicity of the 2009 H1N1 strain. *Vaccine*. 2012;30(45):6427-35.

106. Hurwitz ES, Haber M, Chang A, et al. Studies of the 1996-1997 inactivated influenza vaccine among children attending day care: immunologic response, protection against infection, and clinical effectiveness. *The Journal of infectious diseases.* Oct 2000;182(4):1218-1221.

107. Izikson R, Ibbitson EN, Adams D. Safety and Immunogenicity of Flublok, a Highly Purified Recombinant Recombinant Influenza Vaccine Made without Eggs or Live Influenza Viruses. *The Journal of Allergy and Clinical Immunology*. 2013;131,2.

108. Jackson LA, Gaglani MJ, Keyserling HL, et al. Safety, efficacy, and immunogenicity of an inactivated influenza vaccine in healthy adults: a randomized, placebo-controlled trial over two influenza seasons. *BMC infectious diseases.* Mar 17 2010;10:71.

109. Jamshed S, Walsh EE, Dimitroff LJ, Santelli JS, Falsey AR. Improved immunogenicity of high-dose influenza vaccine compared to standard-dose influenza vaccine in adult oncology patients younger than 65 years receiving chemotherapy: A pilot randomized clinical trial. *Vaccine*. 2016;34(5):630-5.

110. Jeffs LS, Peh CA, Jose MD, Lange K, Hurtado PR. Randomized trial investigating the safety and efficacy of influenza vaccination in patients with antineutrophil cytoplasmic antibody-associated vasculitis. *Nephrology (Carlton, Vic.).* May 2015;20(5):343-351.

111. Kanra G, Marchisio P, Feiterna-Sperling C, et al. Comparison of immunogenicity and tolerability of a virosome-adjuvanted and a split influenza vaccine in children. *The Pediatric infectious disease journal.* Apr 2004;23(4):300-306.

112. Keitel WA, Atmar RL, Cate TR, et al. Safety of high doses of influenza vaccine and effect on antibody responses in elderly persons. *Archives of internal medicine.* May 22 2006;166(10):1121-1127.

113. Keitel WA, Cate TR, Couch RB, Huggins LL, Hess KR. Efficacy of repeated annual immunization with inactivated influenza virus vaccines over a five-year period. *Vaccine.* Jul 1997;15(10):1114-1122.

114. Keitel WA, Treanor JJ, El Sahly HM, et al. Comparative immunogenicity of recombinant influenza hemagglutinin (rHA) and trivalent inactivated vaccine (TIV) among persons > or =65 years old. *Vaccine.* Dec 11 2009;28(2):379-385.

115. Khajedaluee M, Deldar K, Saeedinejat S, Sasan MS. Benefit of inactivated influenza vaccine in infants in Iran: a community based randomized control trial. *Razavi international journal of medicine*. 2018;6(4).

116. Khan AS, Polezhaev F, Vasiljeva R, et al. Comparison of US inactivated split-virus and Russian live attenuated, cold-adapted trivalent influenza vaccines in Russian schoolchildren. *The Journal of infectious diseases.* Feb 1996;173(2):453-456.

117. Kieninger D, Sheldon E, Lin WY, et al. Immunogenicity, reactogenicity and safety of an inactivated quadrivalent influenza vaccine candidate versus inactivated trivalent influenza vaccine: A phase III, randomized trial in adults aged ≥18 years. BMC Infectious Diseases. 2013;13(1).

118. King JC, Jr., Treanor J, Fast PE, et al. Comparison of the safety, vaccine virus shedding, and immunogenicity of influenza virus vaccine, trivalent, types A and B, live cold-adapted, administered to human immunodeficiency virus (HIV)-infected and non-HIV-infected adults. *The Journal of infectious diseases.* Feb 2000;181(2):725-728.

119. King Jr JC, Lagos R, Bernstein DI, et al. Safety and immunogenicity of low and high doses of trivalent live cold- adapted influenza vaccine administered intranasally as drops or spray to healthy children. *Journal of Infectious Diseases.* 1998;177(5):1394-1397.

120. Kositanont U, Kanyok R, Wasi C, Wongsurakiat P, Suthamsmai T, Maranetra N. Occurrence and protective level of influenza infections using serology in patients with COPD in vaccination study. *Journal of the Medical Association of Thailand = Chotmaihet thangphaet.* Aug 2004;87(8):964-969.

121. Kumar D, Campbell P, Hoschler K, et al. Randomized Controlled Trial of Adjuvanted Versus Nonadjuvanted Influenza Vaccine in Kidney Transplant Recipients. *Transplantation.* Mar 2016;100(3):662-669.

122. Lan PT, Toan NT, Thang HA, Thang TC, Be LV, Thai DH, et al. A phase 2/3 double-blind, randomized, placebo-controlled study to evaluate the safety and immunogenicity of a seasonal trivalent inactivated split-virion influenza vaccine (IVACFLU-S) in healthy adults in Vietnam. *Human vaccines & immunotherapeutics*. 2019;15(12):2933-9.

123. Langley JM, Carmona Martinez A, Chatterjee A, et al. Immunogenicity and safety of an inactivated quadrivalent influenza vaccine candidate: a phase III randomized controlled trial in children. *The Journal of infectious diseases.* Aug 15 2013;208(4):544-553.

124. Langley JM, Wang L, Aggarwal N, et al. Immunogenicity and Reactogenicity of an Inactivated Quadrivalent Influenza Vaccine Administered Intramuscularly to Children 6 to 35 Months of Age in 2012-2013: A Randomized, Double-Blind, Controlled, Multicenter, Multicountry, Clinical Trial. *Journal of the Pediatric Infectious Diseases Society.* Sep 2015;4(3):242-251.

125. Leroux-Roels I, Vets E, Freese R, et al. Seasonal influenza vaccine delivered by intradermal microinjection: A randomised controlled safety and immunogenicity trial in adults. *Vaccine.* Dec 2 2008;26(51):6614-6619.

126. Leung DYM, Beck LA, Hanifin JM, et al. A randomized, multi-center, NIH/NIAID funded study to assess the immunogenicity of fluzone® intradermal and intramuscular vaccines in atopic dermatitis. *Journal of Allergy and Clinical Immunology.* 2014;133(2):AB72.

127. Levin MJ, Song LY, Fenton T, et al. Shedding of live vaccine virus, comparative safety, and influenza-specific antibody responses after administration of live attenuated and inactivated trivalent influenza vaccines to HIV-infected children. *Vaccine.* Aug 5 2008;26(33):4210-4217.

128. Levin Y, Kochba E, Shukarev G, Rusch S, Herrera-Taracena G, Damme P. A phase 1, open-label, randomized study to compare the immunogenicity and safety of different administration routes and doses of virosomal influenza vaccine in elderly. *Vaccine.* 2016;34(44):5262-5272.

129. Li R, Fang H, Li Y, Liu Y, Pellegrini M, Podda A. Safety and immunogenicity of an MF59-adjuvanted subunit influenza vaccine in elderly Chinese subjects. *Immunity & ageing :ageing: I & A.* Feb 20 2008;5:2.

130. Liebowitz D, Gottlieb K, Kolhatkar NS, Garg SJ, Asher JM, Nazareno J, et al. Efficacy, immunogenicity, and safety of an oral influenza vaccine: a placebo-controlled and active-controlled phase 2 human challenge study. *The Lancet Infectious diseases.* 2020;20(4):435-44.

131. Li-Kim-Moy JP, Yin JK, Heron L, et al. Influenza vaccine efficacy in young children attending childcare: a randomised controlled trial. *Journal of paediatrics and child health.* 2016;(no pagination).

132. Lum LC, Borja-Tabora CF, Breiman RF, et al. Influenza vaccine concurrently administered with a combination measles, mumps, and rubella vaccine to young children. *Vaccine.* Feb 10 2010;28(6):1566-1574.

133. Madhi SA, Cutland CL, Kuwanda L, et al. Influenza vaccination of pregnant women and protection of their infants. *New England journal of medicine.* 2014;371(10):918-931.

134. Madhi SA, Dittmer S, Kuwanda L, et al. Efficacy and immunogenicity of influenza vaccine in HIV-infected children: a randomized, double-blind, placebo-controlled trial. *AIDS (London, England).* Jan 28 2013;27(3):369-379.

135. Madhi SA, Maskew M, Koen A, et al. Trivalent inactivated influenza vaccine in African adults infected with human immunodeficient virus: double blind, randomized clinical trial of efficacy, immunogenicity, and safety. *Clinical infectious diseases: an official publication of the Infectious Diseases Society of America.* Jan 1 2011;52(1):128-137.
Madhi S, Cutland C, Hugo A, et al. Efficacy and immunogenicity of inactivated influenza vaccine in pregnant women: A randomized, double-blind, placebo-controlled trial. *International Journal of Infectious Diseases*. 2014; 21:430-431.
Nunes MC, Cutland CL, Jones S, et al. Efficacy of Maternal Influenza Vaccination Against All-Cause Lower Respiratory Tract Infection Hospitalizations in Young Infants: Results from a Randomized Controlled Trial. *Clinical Infectious Diseases*. 2017;65(7):1066-1071.

136. Magnani G, Falchetti E, Pollini G, et al. Safety and efficacy of two types of influenza vaccination in heart transplant recipients: a prospective randomised controlled study. *The Journal of heart and lung transplantation: the official publication of the International Society for Heart Transplantation.* May 2005;24(5):588-592.

137. Mallory RM, Nyborg A, Kalyani RN, Yuan Y, Block SL, Dubovsky F. A study to evaluate the immunogenicity and shedding of live attenuated influenza vaccine strains in children 24-<48 months of age. *Vaccine*. 2020;38(5):1001-8.

138. Mallory RM, Yu J, Kameo S, Tanaka M, Rito K, Itoh Y, et al. The safety and efficacy of quadrivalent live attenuated influenza vaccine in Japanese children aged 2-18 years: Results of two phase 3 studies. *Influenza and other respiratory viruses.* 2018;12(4):438-45.

139. Marchisio P, Esposito S, Bianchini S, et al. Efficacy of injectable trivalent virosomal-adjuvanted inactivated influenza vaccine in preventing acute otitis media in children with recurrent complicated or noncomplicated acute otitis media. *The Pediatric infectious disease journal.* Oct 2009;28(10):855-859.

140. McBride WJH, Abhayaratna WP, Barr I, et al. Efficacy of a trivalent influenza vaccine against seasonal strains and against 2009 pandemic H1N1: a randomized, placebo-controlled trial. *Vaccine.* 2016;34(41):4991-4997.

141. McElhaney JE, Beran J, Devaster JM, et al. AS03-adjuvanted versus non-adjuvanted inactivated trivalent influenza vaccine against seasonal influenza in elderly people: a phase 3 randomised trial. *The lancet. Infectious diseases.* 2013;13(6):485-496.
van Essen GA, Beran J, Devaster JM, et al. Influenza symptoms and their impact on elderly adults: randomised trial of AS03-adjuvanted or non-adjuvanted inactivated trivalent seasonal influenza vaccines. *Influenza and other respiratory viruses*. Jul 2014;8(4):452-462.

142. McKittrick N, Frank I, Jacobson JM, et al. Improved immunogenicity with high-dose seasonal influenza vaccine in hiv-infected persons. a single-center, parallel, randomized trial. *Annals of Internal Medicine.* 2013;158(1):19-26.

143. McManus M, Frangoul H, McCullers JA, Wang L, O'Shea A, Halasa N. Safety of high dose trivalent inactivated influenza vaccine in pediatric patients with acute lymphoblastic leukemia. *Pediatric blood & cancer.* 2014;61(5):815-820.

144. Mesa Duque SS, Pérez Moreno A, Hurtado G, Arbeláez Montoya MP. Effectiveness of an influenza vaccine in a working population in Colombia. *Revista panamericana de salud publica [Pan American journal of public health].* 2001;10(4):232-239.

145. Minutello M, Senatore F, Cecchinelli G, et al. Safety and immunogenicity of an inactivated subunit influenza virus vaccine combined with MF59 adjuvant emulsion in elderly subjects, immunized for three consecutive influenza seasons. *Vaccine.* Jan 1999;17(2):99-104.

146. Mixeu MA, Vespa GN, Forleo-Neto E, Toniolo-Neto J, Alves PM. Impact of influenza vaccination on civilian aircrew illness and absenteeism. *Aviation, space, and environmental medicine.* Sep 2002;73(9):876-880.

147. Miyazaki C, Nakayama M, Tanaka Y, et al. Immunization of institutionalized asthmatic children and patients with psychomotor retardation using live attenuated cold-adapted reassortment influenza A H1N1, H3N2 and B vaccines. *Vaccine.* 1993;11(8):853-858.

148. Monto AS, Ohmit SE, Petrie JG, et al. Comparative efficacy of inactivated and live attenuated influenza vaccines. *New England journal of medicine.* 2009;361(13):1260-1267.

149. Morelon E, Pouteil Noble C, Daoud S, et al. Immunogenicity and safety of intradermal influenza vaccination in renal transplant patients who were non-responders to conventional influenza vaccination. *Vaccine.* 2010;28(42):6885-6890.

150. Musto P, Carotenuto M. Vaccination against influenza in multiple myeloma. *British journal of haematology.* May 1997;97(2):505-506.

151. Nace DA, Lin CJ, Ross TM, Saracco S, Churilla RM, Zimmerman RK. Randomized, controlled trial of high-dose influenza vaccine among frail residents of long-term care facilities. *Journal of infectious diseases.* 2015;211(12):1915-1924.

152. Natori Y, Humar A, Lipton J, et al. A pilot randomized trial of adjuvanted influenza vaccine in adult allogeneic hematopoietic stem cell transplant recipients. *Bone marrow transplantation.* 2017;52(7):1016-1021.

153. Natori Y, Shiotsuka M, Slomovic J, et al. A Double-Blind, Randomized Trial of High-Dose vs Standard-Dose Influenza Vaccine in Adult Solid-Organ Transplant Recipients. *Clinical infectious diseases : an official publication of the Infectious Diseases Society of America.* May 17 2018;66(11):1698-1704.

154. Nichol KL, Lind A, Margolis KL, et al. The effectiveness of vaccination against influenza in healthy, working adults. *The New England journal of medicine.* Oct 5 1995;333(14):889-893.

155. Nichol KL, Mendelman PM, Mallon KP, et al. Effectiveness of live, attenuated intranasal influenza virus vaccine in healthy, working adults: a randomized controlled trial. *Jama.* Jul 14 1999;282(2):137-144.

156. Noh JY, Jang YS, Lee SN, Choi MJ, Yoon JG, Yu DH, et al. Randomized, single-blind, active-controlled phase I clinical trial to evaluate the immunogenicity and safety of GC3114 (high-dose, quadrivalent influenza vaccine) in healthy adults. *Vaccine*. 2019;37(36):5171-6.

157. Nolan T, Bravo L, Ceballos A, et al. Enhanced and persistent antibody response against homologous and heterologous strains elicited by a MF59-adjuvanted influenza vaccine in infants and young children. *Vaccine.* 2014;32(46):6146-6156.

158. Ohmit SE, Victor JC, Rotthoff JR, et al. Prevention of antigenically drifted influenza by inactivated and live attenuated vaccines. *New England journal of medicine.* 2006;355(24):2513-2522.

159. Ohmit SE, Victor JC, Teich ER, et al. Prevention of symptomatic seasonal influenza in 2005-2006 by inactivated and live attenuated vaccines. *Journal of infectious diseases.* 2008;198(3):312-317.

160. Ortiz JR, Goswami D, Lewis KDC, et al. Safety of Russian-backbone seasonal trivalent, live-attenuated influenza vaccine in a phase II randomized placebo-controlled clinical trial among children in urban Bangladesh. *Vaccine.* 2015;33(29):3415-3421.

161. Pedroza A, Huerta JG, de la Luz Garcia M, et al. The safety and immunogenicity of influenza vaccine in children with asthma in Mexico. *International Journal of Infectious Diseases.* 2009;13(4):469-475.

162. Pepin S, Donazzolo Y, Jambrecina A, Salamand C, Saville M. Safety and immunogenicity of a quadrivalent inactivated influenza vaccine in adults. *Vaccine.* Nov 12 2013;31(47):5572-5578.

163. Pepin S, Dupuy M, Borja-Tabora CFC, Montellano M, Bravo L, Santos J, et al. Efficacy, immunogenicity, and safety of a quadrivalent inactivated influenza vaccine in children aged 6-35 months: A multi-season randomised placebo-controlled trial in the Northern and Southern Hemispheres. *Vaccine*. 2019;37(13):1876-84.
Pepin S, Samson SI, Alvarez FP, Dupuy M, Gresset-Bourgeois V, De Bruijn I. Impact of a quadrivalent inactivated influenza vaccine on influenza-associated complications and health care use in children aged 6 to 35 months: Analysis of data from a phase III trial in the Northern and Southern Hemispheres. *Vaccine*. 2019;37(13):1885-8.

164. Pepin S, Szymanski H, Rochin Kobashi IA, et al. Safety and immunogenicity of an intramuscular quadrivalent influenza vaccine in children 3 to 8 y of age: A phase III randomized controlled study. *Human vaccines & immunotherapeutics.* Dec 2016;12(12):3072-3078.

165. Powers DC, Smith GE, Anderson EL, et al. Influenza A virus vaccines containing purified recombinant H3 hemagglutinin are well tolerated and induce protective immune responses in healthy adults. *The Journal of infectious diseases.* Jun 1995;171(6):1595-1599.

166. Pregliasco F, Mensi C, Serpilli W, Speccher L, Masella P, Belloni A. Immunogenicity and safety of three commercial influenza vaccines in institutionalized elderly. *Aging (Milan, Italy).* Feb 2001;13(1):38-43.

167. Principi N, Esposito S, Marchisio P, Gasparini R, Crovari P. Socioeconomic impact of influenza on healthy children and their families. *The Pediatric infectious disease journal.* Oct 2003;22(10 Suppl):S207-210.

168. Redding G, Walker RE, Hessel C, et al. Safety and tolerability of cold-adapted influenza virus vaccine in children and adolescents with asthma. *The Pediatric infectious disease journal.* Jan 2002;21(1):44-48.

169. Reid DW, Bromly CL, Stenton SC, Hendrick DJ, Bourke SJ. A double-blind placebo-controlled study of the effect of influenza vaccination on airway responsiveness in asthma. *Respiratory medicine.* Aug 1998;92(8):1010-1011.

170. Rodriguez Weber MA, Claeys C, Aranza Doniz C, et al. Immunogenicity and safety of inactivated quadrivalent and trivalent influenza vaccines in children 18-47 months of age. *The Pediatric infectious disease journal.* Dec 2014;33(12):1262-1269.

171. Rolfes MA, Goswami D, Sharmeen AT, et al. Efficacy of trivalent influenza vaccine against laboratory-confirmed influenza among young children in a randomized trial in Bangladesh. *Vaccine.* 2017;35(50):6967-6976.

172. Rouphael NG, Paine M, Mosley R, et al. The safety, immunogenicity, and acceptability of inactivated influenza vaccine delivered by microneedle patch (TIV-MNP 2015): a randomised, partly blinded, placebo-controlled, phase 1 trial. *The Lancet.* 2017;390(10095):649-658.

173. Rudenko LG, Arden NH, Grigorieva E, et al. Immunogenicity and efficacy of Russian live attenuated and US inactivated influenza vaccines used alone and in combination in nursing home residents. *Vaccine.* 2000;19(2-3):308-318.

174. Ruf BR, Colberg K, Frick M, Preusche A. Open, randomized study to compare the immunogenicity and reactogenicity of an influenza split vaccine with an MF59-adjuvanted subunit vaccine and a virosome-based subunit vaccine in elderly. *Infection.* 2004;32(4):191-198.

175. Safdar A, Rodriguez MA, Fayad LE, et al. Dose-related safety and immunogenicity of baculovirus-expressed trivalent influenza vaccine: A double-blind, controlled trial in adult patients with non-Hodgkin B cell lymphoma. *Journal of Infectious Diseases.* 2006;194(10):1394-1397.

176. Sarkar S, Bokade C, Garg K, Kumar R, Sanmukhani J, Mittal R. Immunogenicity and safety of the first indigenously developed Indian tetravalent influenza vaccine (split virion) in healthy children (6 months to 17 years of age): a randomized, multicenter, phase III clinical trial. *Human vaccines & immunotherapeutics*. 2020:1-9.

177. Sarsenbayeva G, Volgin Y, Kassenov M, et al. Safety and immunogenicity of the novel seasonal preservative- and adjuvant-free influenza vaccine: blind, randomized, and placebo-controlled trial. *Journal of medical virology.* 2017;(no pagination).

178. Saxén H, Virtanen M. Randomized, placebo-controlled double blind study on the efficacy of influenza immunization on absenteeism of health care workers. *Pediatric infectious disease journal.* 1999;18(9):779-783.

179. Schaad UB, Buehlmann U, Burger R, et al. Comparison of immunogenicity and safety of a virosome and a subunit influenza vaccine in pediatric cystic fibrosis (CF) patients: a Swiss multicenter study. *37th interscience conference on antimicrobial agents and chemotherapy; 1997 sept 28 - oct 1.* 1997:238.

180. Scheifele DW, McNeil SA, Ward BJ, et al. Safety, immunogenicity, and tolerability of three influenza vaccines in older adults: results of a randomized, controlled comparison. *Human vaccines & immunotherapeutics.* Nov 2013;9(11):2460-2473.

181. Seo YB, Lee J, Song JY, Choi HJ, Cheong HJ, Kim WJ. Safety and immunogenicity of influenza vaccine among HIV-infected adults: Conventional vaccine vs. intradermal vaccine. *Human vaccines & immunotherapeutics*. 2016;12(2):478-84.

182. Seo YB, Choi WS, Lee J, Song JY, Cheong HJ, Kim WJ. Comparison of the immunogenicity and safety of the conventional subunit, MF59-adjuvanted, and intradermal influenza vaccines in the elderly. *Clinical and vaccine immunology.* 2014;21(7):989-996.

183. Sesay S, Brzostek J, Meyer I, et al. Safety, immunogenicity, and lot-to-lot consistency of a split-virion quadrivalent influenza vaccine in younger and older adults: a phase III randomized, double-blind clinical trial. *Human vaccines and immunotherapeutics.* 20172018;14(3):596-608:1-13.

184. Sharma S, Singh VB, Kumar S, Prajapati V, Patel J, Vukkala R, et al. Immunogenicity and safety of the first indigenously developed Indian tetravalent influenza vaccine (split virion) in healthy adults ≥ 18 years of age: A randomized, multicenter, phase II / III clinical trial. *Human vaccines & immunotherapeutics*. 2018;14(6):1362-9.

185. Sheldon EA, Jeanfreau R, Sliman JA, et al. Immunogenicity of a quadrivalent Ann Arbor strain live attenuated influenza vaccine delivered using a blow-fill-seal device in adults: a randomized, active-controlled study*. *Influenza and other respiratory viruses.* 2013;7(6):1142-1150.

186. Sindoni D, La Fauci V, Squeri R, Cannavò G, Bacilieri S, Panatto D, et al. Comparison between a conventional subunit vaccine and the MF59-adjuvanted subunit influenza vaccine in the elderly: an evaluation of the safety, tolerability and immunogenicity. *Journal of preventive medicine and hygiene.* 2009;50(2):121-6.

187. Sleigh KM, Danforth DG, Hall RT, Fleming JA, Stiver HG. Double-blind, randomized study of the effects of influenza vaccination on the specific antibody response and clinical course of patients with chronic fatigue syndrome. *Journal canadien des maladies infectieuses [canadian journal of infectious diseases].* 2000;11(5):267-273.

188. Solares AR, Solares AR, Aragon CG, et al. Safety and immunogenicity profiles of an adjuvanted seasonal influenza vaccine in Guatemalan children. *Journal of Infection in Developing Countries.* 2014;8(9):1160-1168.

189. Song JY, Cheong HJ, Ha SH, Hwang IS, Kee SY, Jeong HW, et al. Clinical impact of influenza immunization in patients with liver cirrhosis. *Journal of clinical virology: the official publication of the Pan American Society for Clinical Virology*. 2007;39(3):159-63.

190. Song JY, Lee J, Woo HJ, Wie SH, Lee JS, Kim SW, et al. Immunogenicity and safety of an egg-based inactivated quadrivalent influenza vaccine (GC3110A) versus two inactivated trivalent influenza vaccines with alternate B strains: A phase Ⅲ randomized clinical trial in adults. *Human vaccines & immunotherapeutics.* 2019;15(3):710-6.

191. Souza AR, Braga JAP, de Paiva TM, Loggetto SR, Azevedo RS, Weckx LY. Immunogenicity and tolerability of a virosome influenza vaccine compared to split influenza vaccine in patients with sickle cell anemia. *Vaccine.* 2010;28(4):1117-1120.

192. Squarcione S, Sgricia S, Biasio LR, Perinetti E. Comparison of the reactogenicity and immunogenicity of a split and a subunit-adjuvanted influenza vaccine in elderly subjects. *Vaccine*. 2003;21(11-12):1268-74.

193. Stapleton JT, Wagner N, Tuetken R, Bellamy AR, Hill H, Kim S, et al. High dose trivalent influenza vaccine compared to standard dose vaccine in patients with rheumatoid arthritis receiving TNF-alpha inhibitor therapy and healthy controls: Results of the DMID 10-0076 randomized clinical trial. *Vaccine*. 2020;38(23):3934-41.

194. Steinhoff MC, Katz J, Englund JA, et al. Year-round influenza immunisation during pregnancy in Nepal: a phase 4, randomised, placebo-controlled trial. *The lancet. Infectious diseases.* 2017;17(9):981-989.
Kozuki N, Katz J, Englund JA, et al. Impact of maternal vaccination timing and influenza virus circulation on birth outcomes in rural Nepal. *International journal of gynaecology and obstetrics*. 2017;(no pagination).
Tielsch JM, Steinhoff M, Katz J, et al. Designs of two randomized, community-based trials to assess the impact of influenza immunization during pregnancy on respiratory illness among pregnant women and their infants and reproductive outcomes in rural Nepal. *BMC pregnancy and childbirth*. 2015; 15:40.

195. Stevanovic G, Lavadinovic L, Filipovic Vignjevic S, et al. Safety and immunogenicity of a seasonal trivalent inactivated split influenza vaccine: a phase I randomized clinical trial in healthy Serbian adults. *Human vaccines & immunotherapeutics.* Mar 4 2018;14(3):579-586.

196. Stevanovic G, Obradovic A, Ristic S, Petrovic D, Milenkovic B, Mitrovic D, et al. Safety and immunogenicity of a seasonal trivalent inactivated split influenza vaccine: a double blind, phase III randomized clinical trial in healthy Serbian adults. *Therapeutic advances in vaccines and immunotherapy.* 2020;8.

197. Strijbos E, Tannemaat MR, Alleman I, de Meel RHP, Bakker JA, van Beek R, et al. A prospective, double-blind, randomized, placebo-controlled study on the efficacy and safety of influenza vaccination in myasthenia gravis. *Vaccine.* 2019;37(7):919-25.

198. Swierkosz EM, Newman FK, Anderson EL, Nugent SL, Mills GB, Belshe RB. Multidose, live attenuated, cold-recombinant, trivalent influenza vaccine in infants and young children. *Journal of infectious diseases.* 1994;169(5):1121-1124.

199. Tam JS, Capeding MRZ, Lum LCS, et al. Efficacy and safety of a live attenuated, cold-adapted influenza vaccine, trivalent against culture-confirmed influenza in young children in Asia. *Pediatric Infectious Disease Journal.* 2007;26(7):619-628.

200. Tanaka Y, Ueda K, Miyazaki C, et al. Trivalent cold recombinant influenza live vaccine in institutionalized children with bronchial asthma and patients with psychomotor retardation. *Pediatric Infectious Disease Journal.* 1993;12(7):600-605.

201. Tapia MD, Sow SO, Tamboura B, Téguete I, Pasetti MF, Kodio M, et al. Maternal immunisation with trivalent inactivated influenza vaccine for prevention of influenza in infants in Mali: a prospective, active-controlled, observer-blind, randomised phase 4 trial. *The Lancet Infectious diseases*. 2016;16(9):1026-35.

202. Tasker SA, Treanor JJ, Paxton WB, Wallace MR. Efficacy of influenza vaccination in HIV-infected persons. A randomized, double-blind, placebo-controlled trial. *Annals of internal medicine.* 1999;131(6):430-433.

203. Tinoco JC, Pavia-Ruz N, Cruz-Valdez A, et al. Immunogenicity, reactogenicity, and safety of inactivated quadrivalent influenza vaccine candidate versus inactivated trivalent influenza vaccine in healthy adults aged ?18 years: a phase III, randomized trial. *Vaccine.* 2014;32(13):1480-1487.

204. Treanor J, Dumyati G, O'Brien D, et al. Evaluation of cold-adapted, reassortant influenza B virus vaccines in elderly and chronically ill adults. *Journal of infectious diseases.* 1994;169(2):402-407.

205. Treanor JJ, Campbell JD, Brady RC, et al. Rapid licensure of a new, inactivated influenza vaccine in the United States. *Human vaccines.* 2005;1(6):239-244.

206. Treanor JJ, El Sahly H, King J, et al. Protective efficacy of a trivalent recombinant hemagglutinin protein vaccine (FluBlok(R)) against influenza in healthy adults: a randomized, placebo-controlled trial. *Vaccine.* Oct 13 2011;29(44):7733-7739.

207. Treanor JJ, Kotloff K, Betts RF, et al. Evaluation of trivalent, live, cold-adapted (CAIV-T) and inactivated (TIV) influenza vaccines in prevention of virus infection and illness following challenge of adults with wild-type influenza A (H1N1), A (H3N2), and B viruses. *Vaccine.* 1999;18(9-10):899-906.

208. Treanor JJ, Schiff GM, Hayden FG, et al. Safety and immunogenicity of a baculovirus-expressed hemagglutinin influenza vaccine: a randomized controlled trial. *Jama.* 2007;297(14):1577-1582.

209. Treanor JT, Albano FR, Sawlwin DC, et al. Immunogenicity and safety of a quadrivalent inactivated influenza vaccine compared with two trivalent inactivated influenza vaccines containing alternate B strains in adults: a phase 3, randomized noninferiority study. *Vaccine.* 2017;35(15):1856-1864.

210. Tsang P, Gorse GJ, Strout CB, et al. Immunogenicity and safety of Fluzone intradermal and high-dose influenza vaccines in older adults >65 years of age: a randomized, controlled, phase II trial. *Vaccine.* 2014;32(21):2507-2517.

211. Van Damme P, Arnou R, Kafeja F, et al. Evaluation of non-inferiority of intradermal versus adjuvanted seasonal influenza vaccine using two serological techniques: A randomised comparative study. *BMC Infectious Diseases.* 20092010;10:134..

212. van de Witte S, Nauta J, Montomoli E, Weckx J. A Phase III randomised trial of the immunogenicity and safety of quadrivalent versus trivalent inactivated subunit influenza vaccine in adult and elderly subjects, assessing both anti-haemagglutinin and virus neutralisation antibody responses. *Vaccine*. 2018;36(40):6030-8.

213. Vardeny O, Kim K, Udell JA, Joseph J, Desai AS, Farkouh ME, et al. Effect of High-Dose Trivalent vs Standard-Dose Quadrivalent Influenza Vaccine on Mortality or Cardiopulmonary Hospitalization in Patients With High-risk Cardiovascular Disease: A Randomized Clinical Trial. *Jama*. 2020.

214. Vesikari T, Fleming DM, Aristegui JF, et al. Safety, efficacy, and effectiveness of cold-adapted influenza vaccine-trivalent against community-acquired, culture-confirmed influenza in young children attending day care. *Pediatrics.* Dec 2006;118(6):2298-2312.

215. Vesikari T, Karvonen A, Korhonen T, et al. A randomized, double-blind study of the safety, transmissibility and phenotypic and genotypic stability of cold-adapted influenza virus vaccine. *The Pediatric infectious disease journal.* Jul 2006;25(7):590-595.

216. Vesikari T, Karvonen A, Smith HM, et al. Safety and tolerability of cold-adapted influenza vaccine, trivalent, in infants younger than 6 months of Age. *Pediatrics.* 2008;121(3):e568-e573.

217. Vesikari T, Knuf M, Wutzler P, et al. Oil-in-water emulsion adjuvant with influenza vaccine in young children. *The New England journal of medicine.* Oct 13 2011;365(15):1406-1416.

218. Vesikari T, Nauta J, Lapini G, Montomoli E, van de Witte S. Immunogenicity and safety of quadrivalent versus trivalent inactivated subunit influenza vaccine in children and adolescents: A phase III randomized study. *International journal of infectious diseases: IJID : official publication of the International Society for Infectious Diseases.* 2020;92:29-37.

219. Vesikari T, Pellegrini M, Karvonen A, et al. Enhanced immunogenicity of seasonal influenza vaccines in young children using MF59 adjuvant. *Pediatric Infectious Disease Journal.* 2009;28(7):563-571.

220. Vesikari T, Virta M, Heinonen S, Eymin C, Lavis N, Chabanon AL, et al. Immunogenicity and safety of a quadrivalent inactivated influenza vaccine in pregnant women: a randomized, observer-blind trial. *Human Vaccines and Immunotherapeutics.* 2020;16(3):623-9.

221. Victor JC, Lewis KD, Diallo A, et al. Efficacy of a Russian-backbone live attenuated influenza vaccine among children in Senegal: a randomised, double-blind, placebo-controlled trial. *The Lancet. Global health.* Dec 2016;4(12):e955-e965.

222. Volling C, Coleman BL, Katz K, Simor AE, Muller M, Powis J, et al. Immunogenicity and reactogenicity of high- vs. standard-dose trivalent inactivated influenza vaccine in healthcare workers: a pilot randomized controlled trial. *Clinical microbiology and infection: the official publication of the European Society of Clinical Microbiology and Infectious Diseases*. 2019;25(2):217-24.

223. Wang L, Chandrasekaran V, Domachowske JB, Li P, Innis BL, Jain VK. Immunogenicity and Safety of an Inactivated Quadrivalent Influenza Vaccine in US Children 6-35 Months of Age During 2013-2014: Results From A Phase II Randomized Trial. *Journal of the Pediatric Infectious Diseases Society.* Jun 2016;5(2):170-179.

224. Wang S, Liu S, Chu K, et al. Immunogenicity and safety of an inactivated quadrivalent influenza vaccine candidate versus inactivated trivalent influenza vaccines in participants >/=3 years of age: a double-blind, randomized, parallel-controlled phase III clinical trial in China. *Expert review of vaccines.* 2017:1-15.

225. Wang S, Zheng Y, Jin X, Gan Z, Shao Y, Zhu C, et al. Efficacy and safety of a live attenuated influenza vaccine in Chinese healthy children aged 3–17 years in one study center of a randomized, double-blind, placebo-controlled phase 3 clinical trial, 2016/17 season. *Vaccine*. 2020.

226. Ward BJ, Makarkov A, Séguin A, Pillet S, Trépanier S, Dhaliwall J, et al. Efficacy, immunogenicity, and safety of a plant-derived, quadrivalent, virus-like particle influenza vaccine in adults (18-64 years) and older adults (≥65 years): two multicentre, randomised phase 3 trials. *Lancet* (London, England). 2020.

227. Weingarten S, Staniloff H, Ault M, Miles P, Bamberger M, Meyer RD. Do hospital employees benefit from the influenza vaccine? A placebo-controlled clinical trial. *Journal of general internal medicine.* 1988;3(1):32-7.

228. Wilde JA, McMillan JA, Serwint J, Butta J, O'Riordan MA, Steinhoff MC. Effectiveness of influenza vaccine in health care professionals: a randomized trial. *Jama.* 1999;281(10):908-13.

229. Wongsurakiat P, Maranetra KN, Wasi C, Kositanont U, Dejsomritrutai W, Charoenratanakul S. Acute respiratory illness in patients with COPD and the effectiveness of influenza vaccination: a randomized controlled study. *Chest.* Jun 2004;125(6):2011-2020.
Wongsurakiat P, Maranetra KN, Gulprasutdilog P, et al. Adverse effects associated with influenza vaccination in patients with COPD: a randomized controlled study. *Respirology (Carlton, Vic.).* Nov 2004;9(4):550-556.

230. Zaman K, Roy E, Arifeen SE, Rahman M, Raqib R, Wilson E, et al. Effectiveness of maternal influenza immunization in mothers and infants. *The New England journal of medicine*. 2008;359(15):1555-64.

231. Zedda L, Forleo-Neto E, Vertruyen A, et al. Dissecting the immune response to MF59-adjuvanted and nonadjuvanted seasonal influenza vaccines in children less than three years of age. *The Pediatric infectious disease journal.* Jan 2015;34(1):73-78.

Characteristics of included studies

REF	AUTHOR	YEAR	INTERVENTIONS	N. PATIENTS RANDOMISED	AGE	COMORBIDIY/ SPECIAL GROUPS	SETTING	FUNDING SOURCE	CONTINENT	COUNTRY	CENTER
**STUDIES INCLUDED IN THE NETWORK META-ANALYSES**											
1	Abadoglu	2004	trivalent inactivated IM	86	18 -60 years	chronic respiratory disease	outpatients	not reported	Asia	Turkey	not reported
			no vaccine	42							
3	Anh	2016	trivalent inactivated IM	30	18 -60 years	healthy subjects	outpatients	government/ private no profit	Asia	Vietnam	single center
			placebo	30							
2	Allsup	2004	trivalent inactivated IM	552	61 years and older	healthy subjects	outpatients	government/ private no profit	Europe	UK	no reported
			placebo	177							
5	Ansaldi	2012	trivalent inactivated IM	26	18 -60 years	imunodepression (any cause)	outpatients	not reported	Europe	Italy	not reported
			trivalent inactivated intradermal	28							
4	Ansaldi	2013	trivalent inactivated intradermal	250	61 years and older	healthy subjects	outpatients	not reported	Europe	Italy	multi-center
			trivalent inactivated adjuvanted virosome/liposome	250							
6	Arnou	2010	trivalent inactivated intradermal	1803	18 -60 years	healthy subjects	outpatients	industry	Europe	France, Italy, Belgium, Lithuania	multi-center
			trivalent inactivated IM	452							

7	Arnou	2009	trivalent inactivated IM	1089	61 years and older	healthy subjects	outpatients	industry	Europe	France, Italy, Belgium, Lithuania	multi-center
			trivalent inactivated intradermal	2618							
8	Ashkenazi	2006	trivalent live attenuated nasal spray	1101	<5 years	chronic respiratory disease	outpatients	not reported	Multi-Continent	Belgium, Czech Republic, Finland, Germany, Italy, Poland, Spain, Switzerland, UK, Israel	multi-center
			trivalent inactivated (subunit or split) IM	1086							
9	Auewarakul	2006	trivalent inactivated intradermal	400	18 -60 years	healthy subjects	outpatients	not reported	Asia	Thailand	not reported
			trivalent inactivated IM	100							
10	Baldo	2007	trivalent inactivated adjuvanted MF59/AS03 IM	128	18 -60 years	multicomortibity	outpatients	government/ private no profit	Europe	Italy	not reported
			trivalent inactivated IM	128							
11	Baluch	2013	trivalent inactivated) intradermal	114	18 -60 years	imunodepression (any cause)	outpatients	industry	North America	Canada	not reported
			trivalent inactivated IM	115							
12	Barrett	2011	trivalent inactivated IM	3626	18 -60 years	healthy subjects	outpatients	government/ private no profit	North America	Usa	multi-center
			placebo	3624							

13	Bart	2016	quadrivalent inactivated IM	1335	18 -60 years	healthy subjects	outpatients	industry	North America	Usa	multi-center
			trivalent inactivated IM	1345							
14	Baxter	2011	trivalent inactivated recombinant	300	18 -60 years	healthy subjects	outpatients	not reported	North America	Usa	multi-center
			trivalent inactivated IM	302							
15	Belongia	2020	trivalent inactivated high doses (subunit or split) IM	29	61 years and older	healthy subjects	outpatients	government/ private no profit	North America	Usa	single center
			trivalent inactivated adjuvanted (subunit or split) MF59/AS03 IM	30							
			quadrivalent inactivated recombinant	30							
20	Belshe	1992	trivalent live attenuated nasal spray	32	mixed aged children	healthy subjects	outpatients	not reported	North America	Usa	not reported
			placebo	17							
18	Belshe	1998	trivalent live attenuated nasal spray	1070	<5 years	healthy subjects	outpatients	government/ private no profit	North America	Usa	multi-center
			placebo	532							
17	Belshe	2000	trivalent live attenuated nasal spray	917	<5 years	healthy subjects	outpatients	government/ private no profit	North America	Usa	multi-center
			placebo	441							

16	Belshe	2007	trivalent live attenuated nasal spray	4179	<5 years	healthy subjects	outpatients	not reported	Multi-Continent	USA (49% Of Subjects), Europe And Middle East (45% Of Subjects), Asia (6% Of Subjects)	multi-center
			trivalent inactivated IM	4173							
19	Belshe b	2007	trivalent inactivated intradermal	30	18 -60 years	healthy subjects	outpatients	government/ private no profit	North America	Usa	single center
			trivalent inactivated IM	31							
21	Ben-Yehuda	2003	trivalent inactivated IM	33	≥61 years	multicomortibity	long- term care facilities	government/ private no profit	Asia	Israel	not reported
			trivalent inactivated adjuvanted virosome/liposome	48							
22	Ben-Yehuda b	2003	trivalent inactivated IM	37	18 -60 years	healthy subjects	outpatients	not reported	Asia	Israel	not reported
			trivalent inactivated adjuvanted virosome/liposome	36							
26	Beran	2009	trivalent inactivated IM	4137	18 -60 years	healthy subjects	outpatients	industry	Europe	Czech Republic	not reported
			placebo	2066							

24	Beran a	2013	trivalent inactivated IM	105	18 -60 years	healthy subjects	outpatients	industry	Europe	Czech Republic	single center
			trivalent inactivated adjuvanted MF59/AS03 IM	105							
			quadrivalent inactivated IM	105							
			quadrivalent inactivated adjuvanted MF59/AS03	105							
25	Beran b	2009	trivalent inactivated IM	5103	18 -60 years	healthy subjects	outpatients	industry	Europe	Czech Republic, Finland	multi-center
			placebo	2549							
23	Beran c cohort 1	2009	trivalent inactivated IM	547	18 -60 years	healthy subjects	outpatients	industry	Europe	Belgium, Czech Republic, Lithuania	multi-center
			trivalent inactivated intradermal	544							
	Beran c cohort 2		trivalent inactivated IM	410							
			trivalent inactivated (subunit or split) intradermal	418							
27	Bergen	2004	trivalent live attenuated nasal spray	6473	18 -60 years	healthy subjects	outpatients	not reported	Not Reported	Not Reported	not reported
			placebo	3216							
28	Block	2012	quadrivalent inactivated IM	1385	18 -60 years	healthy subjects	outpatients	industry	North America	USA	multi-center
			trivalent inactivated IM	927							

29	Block	2011	quadrivalent inactivated IM	1200	18 -60 years	healthy subjects	outpatients	industry	North America	USA	multi center
			T/LAIV-B/Yamagata	299							
			T/LAIV-B/Victoria	301							
30	Boonnak	2017	trivalent inactivated IM	111	≥61 years	multicomortibity	outpatients	government/ private no profit	not reported	not reported	not reported
			trivalent inactivated intradermal	111							
31	Bracco Neto cohort 1	2009	trivalent live attenuated nasal spray	944	<5 years	healthy subjects	outpatients	industry	Multi-continent	South Africa, Brazil, Argentina	multi-center
			placebo	941							
	Bracco Neto cohort 2	2009	placebo	342							
			trivalent live attenuated nasal spray	338							
32	Bradyy	2018	trivalent live attenuated nasal spray	124	18 -60 years	pregnant women	outpatients	government/ private no profit	North America	USA	multi center
			trivalent inactivated (subunit or split) IM	124							
33	Bridges cohort 1	2000	trivalent inactivated) IM	595	18 -60 years	healthy subjects	outpatients	government/ private no profit	North America	USA	not reported
			placebo	589							
	Bridges cohort 2	2000	trivalent inactivated IM	587	18 -60 years	healthy subjects	outpatients	government/ private no profit			
			placebo	604							

34	Brooks	2016	trivalent live attenuated nasal spray	1174	<5 years	healthy subjects	outpatients	government/ private no profit	Africa	Bangladesh	multi-center
			placebo	587							
35	Bueving	2004	trivalent inactivated IM	347	6-17 years	chronic respiratory disease	outpatients	government/ private no profit	Europe	Holland	not reported
			placebo	349							
36	Cadorna-Carlos	2015	quadrivalent inactivated IM	1648	18 -60 years	healthy subjects	outpatients	not reported	Oceania	Philippines, Australia	multi center
			trivalent inactivated IM	56							
			quadrivalent inactivated IM	329	6-17 years	healthy subjects	outpatients				
			trivalent inactivated IM	55							
37	Caldera	2019	trivalent inactivated high doses (subunit or split) IM	25	18 -60 years	immunodepression (any cause)	outpatients	government/ private no profit	North America	USA	multi center
			quadrivalent inactivated (subunit or split) IM	15							
38	Carr	2011	trivalent inactivated IM	27	mixed aged children	cancer	outpatients	industry	North America	USA	not reported
			trivalent live attenuated nasal spray	28							
39	Chan	2014	trivalent inactivated IM	50	≥61 years	multicomortibity	inpatients	government/ private no profit	Asia	Hong Kong	single center
			trivalent inactivated intradermal	50							

40	Chang	2019	quadrivalent inactivated high doses IM	1777	61 years and older	multicomortibity	outpatients	industry	North America	USA	multi center
			trivalent inactivated high doses (subunit or split) IM	893				industry		USA	
41	Chi	2010	trivalent inactivated IM	65	≥61 years	multicomortibity	outpatients	government/ private no profit	North America	Washington	not reported
			trivalent inactivated intradermal	63							
43	Chiu	2007	trivalent inactivated IM	56	mixed aged children	healthy subjects	outpatients	government/ private no profit	Asia	Hong Kong	not reported
			trivalent inactivated intradermal	56							
42	Chiu	2009	trivalent inactivated IM	63	<5 years	healthy subjects	outpatients	government/ private no profit	Asia	Hong Kong	not reported
			trivalent inactivated intradermal	63							
44	Choi	2018	trivalent inactivated (IM	100	adult and elderly	healthy subjects	outpatients	industry	Asia	Republic of Korea	multi center
			quadrivalent inactivated IM	200							
45	Choi	2017	trivalent inactivated) IM	751	adult and elderly	healthy subjects	outpatients	industry	Asia	Republic of Korea	multi center
			quadrivalent inactivated IM	752							

47	Chuaychoo	2019	trivalent inactivated (subunit or split) IM	39	61 years and older	chronic respiratory disease	outpatients	government/ private no profit	Asia	Bangkok, Thailand	single center
		2019	trivalent inactivated (subunit or split) intradermal ID	41	61 years and older	chronic respiratory disease	outpatients	government/ private no profit		Bangkok, Thailand	
48	Claeys	2018	quadrivalent inactivated (subunit or split) IM	6006	<5 years	healthy subjects	outpatients	industry	Multi continent	Europe,Central America, and Asia	multi center
		2018	pneumococcal conjugate vaccine (PCV), hepatitis A vaccine, or varicella vaccine.	6012							
49	Colmegna	2020	trivalent inactivated high doses (subunit or split) IM	139	adult and elderly	immunodepression (any cause)	outpatients	industry	North America	Canada	multi center
		2020	quadrivalent inactivated (subunit or split) IM	140							
49	Colombo	2001	trivalent inactivated IM	177	<5 years	healthy subjects	outpatients	government/ private no profit	Europe	Italy	not reported
			no vaccine	167							
51	Couch	2007	trivalent inactivated IM	208	≥61 years	healthy subjects	outpatients	government/ private no profit	North America	USA	multi center
			trivalent inactivated high doses IM	206							
52	Cowling	2010	trivalent inactivated IM	71	6-17 years	healthy subjects	outpatients	government/ private no profit	Asia	Hong Kong	not reported
			placebo	48							
53	Cowling	2014	trivalent inactivated IM	479	6-17 years	healthy subjects	outpatients	government/ private no profit	Asia	Hong Kong	multi center
			placebo	317							

54	Cowling	2020	quadrivalent inactivated (subunit or split) IM	508	61 years and older	multicomortibity	outpatients	government/ private no profit	Asia	Hong Kong	single center
			trivalent inactivated adjuvanted (subunit or split) MF59/AS03 IM	508	61 years and older	multicomortibity	outpatients	government/ private no profit		Hong Kong	
			trivalent inactivated high doses (subunit or split) IM	510	61 years and older	multicomortibity	outpatients	government/ private no profit		Hong Kong	
			quadrivalent inactivated recombinant	335	61 years and older	multicomortibity	outpatients	government/ private no profit		Hong Kong	
55	Cruz-Valdez	2018	trivalent inactivated IM	143	<5 years	healthy subjects	outpatients	industry	South America	Mexico	multi center
			trivalent inactivated adjuvanted MF59/AS03 IM	144							
57	De Bruijn	2006	trivalent inactivated IM	129	adult and elderly	healthy subjects	outpatients	not reported	not reported	not reported	not reported
			trivalent inactivated adjuvanted virosome/liposome	127							
			trivalent inactivated adjuvanted MF59/AS03 IM	130							
56	De Bruijn	2007	trivalent inactivated IM	129	≥61 years	healthy subjects	outpatients	not reported	Europe	Germany, Sweden, Lithuania, Bulgaria	multi center
			trivalent inactivated adjuvanted MF59/AS03 IM	130							
			trivalent inactivated adjuvanted virosome/liposome	127							

58	De Donato	1999	trivalent inactivated adjuvanted MF59/AS03 IM	106	≥61 years	healthy subjects	outpatients	not reported	Europe	Italy	not reported
			trivalent inactivated IM	105							
59	De Villiers	2009	trivalent live attenuated nasal spray	1620	≥61 years	multicomortibity	outpatients	industry	Africa	Republic of South Africa	multi center
			placebo	1622							
61	Desheva	2002	trivalent live attenuated nasal spray	184	<5 years	healthy subjects	outpatients	not reported	Asia	Russia	not reported
			placebo	72							
62	Diallo 2018	2018	trivalent inactivated (subunit or split) IM	119	<5 years	healthy subjects	outpatients	government/ private no profit	Africa	Senegal	single center
			trivalent inactivated adjuvanted (subunit or split) MF59/AS03 IM	118	<5 years	healthy subjects	outpatients	government/ private no profit		Senegal	
			placebo/no intervention	59	<5 years	healthy subjects	outpatients	government/ private no profit		Senegal	
63	DiazGranados	2013	trivalent inactivated IM	3055	≥61 years	multicomortibity	outpatients	industry	North America	USA	multi center
			trivalent inactivated high doses IM	6117							
64	DiazGranados	2014	trivalent inactivated IM	15998	≥61 years	multicomortibity	outpatients	industry	North America	USA, Canada	multi center
			trivalent inactivated high doses IM	15991							
65	DiazGranados	2015	trivalent inactivated high doses IM	148	18 -60 years	healthy subjects	outpatients	industry	North America	USA, Canada	multi center
			trivalent inactivated IM	152							

66	Domachowske	2013	trivalent inactivated IM	1823	mixed aged children	healthy subjects	outpatients	industry	Multi-continent	Czech Republic, France, Germany, Philippines, USA	multi center
			quadrivalent inactivated (subunit or split) IM	915							
67	Dunkle	2017	quadrivalent inactivated IM	4505	adult and elderly	healthy subjects	outpatients	government/ private no profit	North America	USA	multi center
			quadrivalent inactivated recombinant	4498							
69	Dunkle b	2017	quadrivalent inactivated IM	339	18 -60 years	healthy subjects	outpatients	government/ private no profit	North America	USA	multi center
			quadrivalent inactivated recombinant	1011							
68	Dunkle	2018	quadrivalent inactivated recombinant	108	6-17 years	healthy subjects	outpatients	industry	North America	USA	multi center
			quadrivalent inactivated (subunit or split) IM	111	6-17 years						
70	Durando cohort 1	2008	trivalent inactivated adjuvanted MF59/AS03 IM	81	18 -60 years	healthy subjects	outpatients	industry	Europe	Italy	multi center
			trivalent inactivated IM	80							
	Durando cohort 2		trivalent inactivated adjuvanted MF59/AS03 IM	46	18 -60 years	imunodepression (any cause)	outpatients	industry			
			trivalent inactivated IM	49							
71	Englund	2010	trivalent inactivated IM	915	<5 years	healthy subjects	outpatients	not reported	North America	USA	multi center
			placebo	460							

72	Esposito	2010	trivalent inactivated IM	91	<5 years	cancer	outpatients	government/ private no profit	Europe	Italy	multi center
			no vaccine	91							
73	Esposito	2011	trivalent inactivated IM	37	6-17 years	healthy subjects	outpatients	government/ private no profit	Europe	Italy	not reported
			trivalent inactivated intradermal	75							
74	Esposito	2014	trivalent inactivated IM	30	6-17 years	imunodepression (any cause)	outpatients	government/ private no profit	Europe	Italy	not reported
			trivalent inactivated adjuvanted virosome/liposome	30							
75	Essink	2019	quadrivalent inactivated adjuvanted MF5quadrivalent inactivated intradermal/ASplacebo/no intervention trivalent inactivated (subunit or split) intradermal ID	889	61 years and older	healthy subjects+multicomortibity	outpatients	industry	North America	20 centers usa	multi center
			trivalent inactivated adjuvanted (subunit or split) MF59/AS03 IM	889	61 years and older	healthy subjects+multicomortibity	outpatients	industry		20 centers usa	
76	Evison	2009	trivalent inactivated IM	151	18 -60 years	imunodepression (any cause)	outpatients	industry	Europe	Switzerland	not reported
			trivalent inactivated adjuvanted virosome/liposome	153							
77	Falsey	2009	trivalent inactivated IM	1288	≥61 years	multicomortibity	outpatients	industry	North America	USA	multi center
			trivalent inactivated high doses IM	2588							

79	Fleming	2006	trivalent live attenuated nasal spray	1114	6-17 years	chronic respiratory disease	outpatients	industry	Multi-continent	Belgium, Finland, Germany, Greece, Israel, Italy, the Netherlands, Norway, Poland, Portugal, Spain, Switzerland, UK	multi center
			trivalent inactivated IM	1115							
80	Forrest	2011	trivalent inactivated IM	1501	≥61 years	healthy subjects	outpatients	industry	Africa	Republic of South Africa	multi center
			trivalent live attenuated nasal spray	1508							
81	Frenck	2011	trivalent inactivated IM	398	18 -60 years	healthy subjects	outpatients	government/ private no profit	North America	USA	multi center
			trivalent inactivated intradermal	394							
83	Frey	2010	trivalent inactivated IM	7504	18 -60 years	healthy subjects	outpatients	industry	Multi-continent	USA, Finland, Poland	multi center
			placebo	3900		healthy subjects	outpatients	industry			
82	Frey	2003	trivalent inactivated IM	151	18 -60 years	healthy subjects	outpatients	Not reported	North America	USA	multi center
			trivalent inactivated adjuvanted MF59/AS03 IM	150							

84	Frey	2014	trivalent inactivated IM	3552	≥61 years	healthy subjects	outpatients	industry	Multi-continent	Colombia, Panama, Philippines, USA	multi center
			trivalent inactivated adjuvanted MF59/AS03 IM	3552							
85	Gabutti	2005	trivalent inactivated IM	19	18 -60 years	imunodepression (any cause)	outpatients	government/ private no profit	Europe	Italy	multi center
			trivalent inactivated adjuvanted MF59/AS03 IM	18							
86	Gasparini	2001	trivalent inactivated IM	104	≥61 years	healthy subjects	outpatients	not reported	Europe	Italy	multi center
			trivalent inactivated adjuvanted MF59/AS03 IM	204							
87	Giaquinta	2014	trivalent inactivated IM	15	3-17 years	solid organ transplantation	outpatients	industry	North America	USA	multi center
			trivalent inactivated high doses IM	23							
89	Gorse b	2013	trivalent inactivated IM	495	18 -60 years	healthy subjects	outpatients	industry	North America	USA	multi center
			trivalent inactivated intradermal	755							
90	Gorse	2015	trivalent inactivated intradermal	1683	18 -60 years	healthy subjects	outpatients	industry	North America	USA	multi center
			quadrivalent inactivated intradermal	1672							
88	Gorse	2013	trivalent inactivated IM	1428	18 -60 years	healthy subjects	outpatients	industry	North America	USA	multi center
			trivalent inactivated intradermal	2864							

91	Govaert	1993	quadrivalent inactivated IM	927	≥61 years	multicomortibity	outpatients	government/ private no profit	Europe	Netherlands	multi center
			placebo	911							
92	Greenberg	2014	trivalent inactivated IM	1461	6-17 years	healthy subjects	outpatients	industry	North America	USA	multi center
			quadrivalent inactivated IM	2902		healthy subjects	outpatients	industry			
94	Greenberg	2017	trivalent inactivated IM	450	≥61 years	healthy subjects	outpatients	industry	North America	USA	multi center
			quadrivalent inactivated IM	225							
93	Greenberg	2013	trivalent inactivated IM	380	adult and elderly	healthy subjects	outpatients	industry	North America	USA	multi center
			quadrivalent inactivated IM	190							
95	Hakim	2016	trivalent inactivated) IM	42	6-17 years	cancer +imunodepression (any cause)	outpatients	government/ private no profit	North America	USA	single center
			trivalent inactivated high doses IM	41							
96	Halasa	2011	trivalent live attenuated nasal spray	10	6-17 years	imunodepression (any cause)	outpatients	industry	North America	USA	multi center
			placebo	10							
97	Halasa	2016	trivalent inactivated IM	15	18 -60 years	transplantation	outpatients	industry	North America	USA	single center
			trivalent inactivated high doses IM	29							

98	Hartvickson	2015	trivalent inactivatedIM	1174	6-17 years	healthy subjects	outpatients	industry	North America	USA	multi center
			quadrivalent inactivated IM	1159							
99	Hobermann cohort 1	2003	trivalent inactivated IM	278	<5 years	healthy subjects	outpatients	industry	North America	USA	single center
			placebo	139							
	Hobermann cohort 2	2003	trivalent inactivated IM	253	<5 years	healthy subjects	outpatients				
			placebo	123							
100	Hoft	2011	trivalent inactivated IM	14	<5 years	healthy subjects	outpatients	government/ private no profit	North America	USA	multi center
			trivalent live attenuated nasal spray	13							
101	Holland	2008	trivalent inactivated IM	368	≥61 years	multicomortibity	outpatients	industry	Oceania	Australia, New Zealand	multi center
			trivalent inactivated intradermal	370							
102	Hoon Han cohort 1	2013	trivalent inactivated IM	60	18 -60 years	healthy subjects	outpatients	not reported	Asia	South Korea	multi center
			trivalent inactivatedintradermal	60							
	Hoon Han cohort 2	2013	trivalent inactivated IM	60	≥61 years	healthy subjects	outpatients				
			trivalent inactivated intradermal	60							

103	Hu	2019 b	quadrivalent inactivated (subunit or split) IM	1160	<5 years	healthy subjects	outpatients	industry	Asia	China	multi center
			trivalent inactivated (subunit or split) IM	1160	<5 years						
104	Hui	2006	trivalent inactivated IM	65	≥61 years	healthy subjects	outpatients	government/ private no profit	Asia	Hong Kong	multi center
			placebo	63							
105	Hung	2012	trivalent inactivated (subunit or split) intradermal ID	133	61 years and older	multicomortibity	outpatients	government/ private no profit	Asia	Hong Kong	single center
			trivalent inactivated (subunit or split) IM	66							
106	Hurwitz	2000	trivalent inactivated (subunit or split) IM	76	<5 years	healthy subjects	outpatients	industry	North America	10 US Navy–affiliated day care centers	multi center
			hepatitis A vaccine	74							
108	Jackson cohort 1	2010	trivalent inactivated IM	1734	18 -60 years	healthy subjects	outpatients	industry	North America	USA	multi center
			placebo	1756							
	Jackson cohort 1+2	2010	trivalent inactivated IM	3783	18 -60 years	healthy subjects	outpatients				
			placebo	3828							
	Jackson cohort 2	2010	trivalent inactivated IM	2048	18 -60 years	healthy subjects	outpatients				
			placebo	2073							

109	Jamshe	2019	trivalent inactivated (subunit or split) IM	51	18 -60 years	cancer	outpatients	industry+government/ private no profit	North America	USA	single center
			trivalent inactivated high doses (subunit or split) IM	54	18 -60 years						
110	Jeffs	2015	trivalent inactivated IM	24	18 -60 years	immunosuppression in autoimmune vasculitis	outpatients	government/ private no profit	Oceania	Australia	not reported
			no vaccine	7							
			trivalent inactivated IM	53	18 -60 years	healthy subjects	outpatients				
			no vaccine	14							
111	Kanra	2004	trivalent inactivated IM	228	<5 years	healthy subjects	outpatients	industry	Multi-continent	Germany, Italy, Turkey.	multi center
			trivalent inactivated recombinant	224							
112	Keitel	2006	placebo	50	≥61 years	healthy subjects	outpatients	government/ private no profit	North America	USA	single center
			trivalent inactivated IM	51							
			trivalent inactivated high doses IM	50							
114	Keitel	2009	trivalent inactivated IM	434	≥61 years	healthy subjects	outpatients	not reported	not reported	not reported	multi center
			trivalent inactivated recombinant	436							

113	Keitel cohort 1	1997	placebo	298	18 -60 years	healthy subjects	outpatients	government/ private no profit	North America	USA	multi center
			trivalent inactivated IM	300							
	Keitel cohort 2	1997	placebo	241	18 -60 years	healthy subjects	outpatients				
			trivalent inactivated IM	457							
	Keitel cohort 3	1997	placebo	253	18 -60 years	healthy subjects	outpatients				
			trivalent inactivated IM	577							
	Keitel cohort 4	1997	placebo	217	18 -60 years	healthy subjects	outpatients				
			trivalent inactivated IM	723							
	Keitel cohort 5	1997	placebo	145	18 -60 years	healthy subjects	outpatients				
			trivalent inactivated IM	789							
115	Khajedaluee	2018	trivalent inactivated (subunit or split) IM	500	<5 years	healthy subjects	outpatients	not reported	Asia	Iran	multi center
			placebo/no intervention	500	<5 years						

116	Khan	1996	trivalent inactivated IM	168	6-17 years	healthy subjects	outpatients	not reported	Asia	Russia	not reported
			placebo	187							
			trivalent live attenuated	200							
117	Kieninger	2013	trivalent inactivated IM	1620	adult and elderly	healthy subjects	outpatients	industry	Multi-continent	Germany, Romania, Spain, Korea, Taiwan , USA	multi center
			quadrivalent inactivated IM	3036							
118	King cohort 1	2000	trivalent live attenuated nasal spray	27	18 -60 years	healthy subjects	outpatients	not reported	North America	USA	multi center
			placebo	27	18 -60 years						
	King cohort 2	2000	trivalent live attenuated nasal spray	28	18 -60 years	imunodepression (any cause)	outpatients				
			placebo	29	18 -60 years						
119	King	1998	trivalent live attenuated nasal spray	234	mixed aged children	healthy subjects	outpatients	industry	North America	USA	multi center
			placebo	122							
120	Kositanont	2004	trivalent inactivated IM	61	≥61 years	chronic respiratory disease	outpatients	government/ private no profit	Asia	Thailand	single center
			placebo	62							
121	Kumar	2016	trivalent inactivated IM	34	18 -60 years	imunodepression (any cause)	outpatients	government/ private no profit	North America	Canada	single center
			trivalent inactivated adjuvanted MF59/AS03 IM	34							

122	Lan	2019	trivalent inactivated (subunit or split) IM	740	18 -60 years	healthy subjects	outpatients	government/ private no profit	Asia	Vietnam	not reported
			placebo/no intervention	148							
124	Langley	2014	trivalent inactivated IM	302	<5 years	healthy subjects	outpatients	industry	Multi-continent	Canada, Dominican Republic, Honduras	multi center
			quadrivalent inactivated IM	299							
123	Langley	2013	trivalent inactivated IM	1861	mixed aged children	healthy subjects	outpatients	industry	Multi-continent	Canada, USA, Mexico, Spain, Taiwan	multi center
			quadrivalent inactivated IM	932							
125	Leroux-Roels	2008	trivalent inactivatedIM	390	18 -60 years	healthy subjects	outpatients	industry	Europe	Belgium, Germany, Switzerland	multi center
			trivalent inactivated intradermal	588							
127	Levin	2008	trivalent inactivated IM	121	6-17 years	imunodepression (any cause)	outpatients	government/ private no profit	North America	USA	not reported
			trivalent live attenuated nasal spray	122							
128	Levin	2016	trivalent inactivated intradermal	60	≥61 years	healthy subjects	outpatients	industry	Europe	Germany, Belgium	multi center
			trivalent inactivated adjuvanted MF59/AS03 IM	63							
			trivalent inactivated adjuvanted virosome/liposome	63							

129	Li	2008	trivalent inactivated IM	200	≥61 years	healthy subjects	outpatients	industry	Asia	China	not reported
			trivalent inactivated adjuvanted MF59/AS03 IM	400							
130	Liebowitz	2020	quadrivalent inactivated (subunit or split) IM	72	18 -60 years	healthy subjects	outpatients	government/ private no profit	North America	USA	not reported
			placebo/no intervention	36							
131	Li-Kim-Moy	2016	trivalent inactivated (subunit or split) IM	57	<5 years	healthy subjects	outpatients	industry+government/ private no profit	Oceania	Sydney, Australia	multi center
			hepatitis A vaccine	67							
132	Lum	2010	trivalent live attenuated nasal spray	819	<5 years	healthy subjects	outpatients	not reported	Multi-continent	Bangladesh, Belgium, Finland, Germany, Hong Kong, Lithuania, Malaysia, Mexico, Philippines, Poland, Singapore, South Korea, Thailand	multi center
			placebo	414							
135	Madhi	2011	trivalent inactivated IM	255	18 -60 years	imunodepression (any cause)	outpatients	government/ private no profit	Africa	South Africa	single center
			placebo	251							
134	Madhi	2013	trivalent inactivated IM	206	mixed aged children	imunodepression (any cause)	outpatients	government/ private no profit	Africa	South Africa	single center
			placebo	204							

133	Madhi cohort 1	2014	trivalent inactivated IM	1062	18 -60 years	pregnant women	outpatients	government/ private no profit	Africa	South Africa	single center
			placebo	1054							
	Madhi cohort b	2014	trivalent inactivated) IM	100	18 -60 years	pregnant women+imunodepression (any cause)	outpatients	government/ private no profit			
			placebo	94							
136	Magnani	2005	trivalent inactivated IM	21	adult and elderly	imunodepression (any cause)	outpatients	not reported	Europe	Italy	single center
			trivalent inactivated adjuvanted MF59/AS03 IM	21							
			no vaccine	16							
138	Mallory (only study 2)	2018	quadrivalent live attenuated nasal spray	868	mixed aged children	healthy subjects+chronic respiratory disease	outpatients	industry	Asia	Japan	multi center
			placebo/no intervention	433							
137	Mallory	2020	trivalent live attenuated nasal spray	67	<5 years	healthy subjects	outpatients	industry	North America	USA	multi center
			quadrivalent live attenuated nasal spray	133							
139	Marchisio	2009	trivalent inactivated adjuvanted virosome/liposome	90	<5 years	healthy subjects	outpatients	government/ private no profit	Europe	Italy	not reported
			placebo	90							

140	McBride cohort 1	2016	trivalent inactivated IM	5032	18 -60 years	healthy subjects	outpatients	industry	Oceania	Australia , New Zealand	multi center
			placebo	2512							
	McBride cohort 2	2016	trivalent inactivated IM	5001	18 -60 years	healthy subjects	outpatients	industry			
			placebo	2499							
141	McElhaney	2013	trivalent inactivated IM	21802	≥61 years	healthy subjects	outpatients	industry	Multi-continent	Belgium, Canada, Czech Republic, Estonia, France, Germany, Mexico, Norway, Poland, Romania, Russia, Taiwan, the Netherlands, UK, USA	multi center
			trivalent inactivated adjuvanted MF59/AS03 IM	21893		healthy subjects	outpatients	industry			
142	McKittrick	2013	trivalent inactivated IM	98	18 -60 years	imunodepression (any cause)	outpatients	government/ private no profit	North America	USA	single center
			trivalent inactivated high doses IM	100							
143	McManus	2014	trivalent inactivated IM	16	6-17 years	cancer	outpatients	government/ private no profit	North America	USA	single center
			trivalent inactivated high doses (subunit or split) IM	34							

144	Mesa -Duque	2001	trivalent inactivated IM	247	18 -60 years	healthy subjects	outpatients	not reported	South America	Colombia	not reported
			placebo	246							
145	Minutello	1999	trivalent inactivated IM	46	≥61 years	healthy subjects	outpatients	not reported	Europe	Italy	single center
			trivalent inactivated adjuvanted MF59/AS03 IM	46							
146	Mixeu	2002	trivalent inactivated IM	405	18 -60 years	healthy subjects	outpatients	industry	South America	Brazil	single center
			placebo	408							
147	Miyazaki	1993	trivalent live attenuated nasal spray	19	6-17 years	chronic respiratory disease	inpatients	government/ private no profit	Asia	Japan	single center
			no vaccine	20							
148	Monto	2009	trivalent inactivated IM	814	18 -60 years	healthy subjects	outpatients	government/ private no profit	North America	USA	single center
			trivalent live attenuated nasal spray	813							
			placebo	325							
149	Morelon	2010	trivalent inactivated IM	31	18 -60 years	imunodepression (any cause)	outpatients	industry	Europe	France	multi center
			trivalent inactivated intradermal	31							
150	Musto	1997	trivalent inactivated IM	25	18 -60 years	cancer	outpatients	not reported	Europe	Italy	single center
			no vaccine	25							

152	Natori a	2017	trivalent inactivated adjuvanted MF59/AS03 IM	35	adult and elderly	imunodepression (any cause)	outpatients	industry	not reported	not reported	not reported
			trivalent inactivated IM	38							
153	Natori b	2017	trivalent inactivated IM	85	18 -60 years	imunodepression (any cause)	outpatients	government/ private no profit	not reported	not reported	not reported
			trivalent inactivated highIM	87							
155	Nichol	1999	trivalent live attenuated nasal spray	3041	18 -60 years	healthy subjects	outpatients	industry	North America	USA	multi center
			placebo	1520							
154	Nichol	1995	trivalent inactivated IM	424	18 -60 years	healthy subjects	outpatients	government/ private no profit	North America	USA	not reported
			placebo	425		healthy subjects	outpatients	government/ private no profit			
156	Noh	2019	quadrivalent inactivated high doses IM	30	18 -60 years	healthy subjects	outpatients	industry	Asia	KOREA	single center
			quadrivalent inactivated (subunit or split) IM	10							
157	Nolan	2014	trivalent inactivated IM	2964	<5 years	healthy subjects	outpatients	industry	Multi-continent	Argentina, Australia, Chile, Philippines, South Africa	multi center
			trivalent inactivated adjuvanted MF59/AS03 IM	3136							

159	Ohmit	2008	trivalent inactivated IM	818	18 -60 years	healthy subjects	outpatients	government/ private no profit	North America	USA	multi center
			placebo	338							
			trivalent live attenuated nasal spray	787							
158	Ohmit	2006	trivalent inactivated IM	522	18 -60 years	healthy subjects	outpatients	government/ private no profit	North America	USA	multi center
			placebo	206							
			trivalent live attenuated nasal spray	519							
160	Ortiz	2015	trivalent live attenuated nasal spray	150	<5 years	healthy subjects	outpatients	government/ private no profit	Africa	Bangladesh	single center
			placebo	150							
161	Pedroza	2009	trivalent inactivated) IM	132	6-17 years	chronic respiratory disease	outpatients	industry	South America	Mexico	single center
			placebo	31							
162	Pepin	2013	trivalent inactivated IM	449	adult and elderly	healthy subjects	outpatients	industry	Europe	France, Germany	multi center
			quadrivalent inactivated (subunit or split) IM	1116							
164	Pepin	2016	trivalent inactivated IM	355	<5 years	healthy subjects	outpatients	industry	Multi-continent	Poland, Finland, Mexico, Taiwan	multi center
			quadrivalent inactivated IM	887							

163	Pepin	2019	quadrivalent inactivated (subunit or split) IM	2721	<5 years	healthy subjects	outpatients	industry	Multi continent	Asia, Latin America, Europe, and Africa	multi center
			placebo/no intervention	2715							
			trivalent inactivated (subunit or split) IM	369							
165	Powers	1995	trivalent inactivated recombinant	77	18 -60 years	healthy subjects	outpatients	not reported	North America	USA	multi center
			trivalent inactivated IM	26							
			placebo	24							
166	Pregliasco	2001	trivalent inactivated IM	213	≥61 years	nr	long term care facilities	not reported	Europe	Italy	multi center
			trivalent inactivated adjuvanted MF59/AS03 IM	207							
			trivalent inactivated adjuvanted virosome/liposome	215							
168	Redding	2002	trivalent live attenuated nasal spray	24	6-17 years	chronic respiratory disease	outpatients	industry	North America	USA	multi center
			placebo	24							
169	Reid	1998	trivalent inactivated IM	17	18 -60 years	chronic respiratory disease	outpatients	not reported	not reported	not reported	not reported
			placebo	5							

170	Rodriguez Weber	2014	trivalent inactivated IM	301	<5 years	healthy subjects	outpatients	industry	South America	Mexico	multi center
			quadrivalent inactivated IM	298							
171	Rolfes	2017	trivalent inactivated (subunit or split) IM	2576	<5 years	healthy subjects	outpatients	government/ private no profit	Asia	Bangladesh	single center
			inactivated polio vaccine (IPV)	2593							
172	Rouphael	2017	trivalent inactivated IM	25	18 -60 years	healthy subjects	outpatients	government/ private no profit	North America	USA	not reported
			trivalent inactivated intradermal	25							
			placebo	25							
173	Rudenko	2001	trivalent inactivated IM	95	≥61 years	multicomortibity	long term care facilities	not reported	Asia	Russia	multi center
			trivalent live attenuated nasal spray	114							
			placebo	110							
174	Ruf	2004	trivalent inactivated IM	273	adult and elderly	healthy subjects	outpatients	not reported	Europe	Germany	multi center
			trivalent inactivated adjuvanted MF59/AS03 IM	275							
			trivalent inactivated adjuvanted virosome/liposome	272							
175	Safdar	2006	trivalent inactivated IM	6	18 -60 years	cancer	inpatients	not reported	North America	USA	not reported
			trivalent inactivated recombinant	21							

176	Sarkar	2020	trivalent inactivated (subunit or split) IM	155	mixed aged children	healthy subjects	outpatients	industry	Asia	India	multi center
			quadrivalent inactivated (subunit or split) IM	151							
177	Sarsenbayeva	2018	trivalent inactivated IM	22	18 -60 years	healthy subjects	outpatients	not reported	Asia	Kazakhstan	single center
			placebo	22							
178	Saxen	1999	trivalent inactivated IM	216	18 -60 years	healthy subjects	outpatients	not reported	Europe	Finland	multi center
			placebo	211							
179	Schaad	2000	trivalent inactivated IM	21	mixed aged children	chronic respiratory disease	outpatients	not reported	Europe	Switzerland	multi center
			trivalent inactivated adjuvanted virosome/liposome	43							
180	Scheifele	2013	trivalent inactivated IM	310	≥61 years	healthy subjects	outpatients	government/ private no profit	North America	Canada	multi center
			trivalent inactivated intradermal	306							
			trivalent inactivated adjuvanted virosome/liposome	306							
182	Seo	2014	trivalent inactivated) IM	113	≥61 years	healthy subjects	outpatients	government/ private no profit	Asia	South Korea	multi center
			trivalent inactivated intradermal	111							
			trivalent inactivated adjuvanted virosome/liposome	111							

181	Seo	2016	trivalent inactivated IM	28	18 -60 years	imunodepression (any cause)	outpatients	not reported	Asia	Republic of Korea	multi center
			trivalent inactivated intradermal	58							
184	Sharma	2018	trivalent inactivated (subunit or split) IM	174	adult and elderly	healthy subjects	long term care facilities	industry	Asia	India	multi center
			quadrivalent inactivated (subunit or split) IM	176	adult and elderly						
185	Sheldon	2013	trivalent live attenuated nasal spray	598	18 -60 years	healthy subjects	outpatients	industry	North America	USA	multi center
			quadrivalent live attenuated nasal spray	1202							
186	Sindoni	2009	trivalent inactivated adjuvanted (subunit or split) MF59/AS03 IM	96	61 years and older	multicomortibity	long term care facilities	not reported	Europe	Italy	single center
			trivalent inactivated (subunit or split) IM	99	61 years and older	multicomortibity	long term care facilities	not reported			
188	Solares	2014	trivalent inactivated IM	180	<5 years	healthy subjects	outpatients	industry	South America	Guatemala	multi center
			trivalent inactivated adjuvante) MF59/AS03 IM	180							
189	Song	2007	trivalent inactivated (subunit or split) IM	198	adult and elderly	multicomortibity	outpatients	not reported	Asia	Seoul, Korea	single center
			placebo/no intervention	113	adult and elderly						
190	Song	2019	quadrivalent inactivated (subunit or split) IM	648	adult and elderly	healthy subjects	outpatients	industry+government/ private no profit	Asia	South Korea	multi center
			trivalent inactivated (subunit or split) IM	651							

192	Squarcione	2003	trivalent inactivated (subunit or split) IM	1076	61 years and older	healthy subjects	outpatients	not reported	Europe	Italy	single center
			trivalent inactivated adjuvanted (subunit or split) MF59/AS03 IM	1074	61 years and older						
194	Steinhoff cohort 1	2017	trivalent inactivated IM	1049	18 -60 years	pregnant women	outpatients	government/ private no profit	Asia	Nepal	multi center
			placebo	1041							
	Steinhoff cohort b	2017	trivalent inactivated) IM	798	18 -60 years						
			placebo	805							
195	Stevanovic	2017	trivalent inactivated IM	30	18 -60 years	healthy subjects	outpatients	government/ private no profit	Europe	Serbia	single center
			placebo	30							
196	Stevanovic	2020	trivalent inactivated (subunit or split) IM	320	18 -60 years	healthy subjects	outpatients	government/ private no profit	Asia	Serbia	multi center
			placebo/no intervention	160							
197	Strijbos	2019	trivalent inactivated (subunit or split) IM	24	adult and elderly	immunodepression (any cause)	outpatients	government/ private no profit	Europe	Netherlands	single center
			placebo/no intervention	23							
198	Swierkosz	1994	trivalent live attenuated nasal spray	17	mixed aged children	healthy subjects	outpatients	not reported	not reported	not reported	not reported
			placebo	5							

199	Tam cohort 1	2007	trivalent live attenuated nasal spray	1900	mixed aged children	healthy subjects	outpatients	industry	Multi-continent	China, Hong Kong, India, Malaysia, Philippines, Singapore, Taiwan, Thailand	multi center
			placebo	1274							
	Tam cohort b	2007	trivalent live attenuated nasal spray	1477							
			placebo	1470							
200	Tanaka	1993	trivalent live attenuated nasal spray	20	mixed aged children	chronic respiratory disease	inpatients	government/ private no profit	not reported	not reported	not reported
			placebo	25							
201	Tapia	2016	trivalent inactivated (subunit or split) IM	2108	18 -60 years	pregnant women	outpatients	government/ private no profit	Africa	Mali	multi center
			meningococcal vaccine	2085	18 -60 years	pregnant women	outpatients	government/ private no profit			
202	Tasker	1999	trivalent inactivated IM	55	18 -60 years	imunodepression (any cause)	outpatients	not reported	North America	USA	single center
			placebo	47							
203	Tinoco	2014	trivalent inactivated IM	431	adult and elderly	healthy subjects	outpatients	industry	Multi-continent	Canada, Mexico, USA	multi center
			quadrivalent inactivated IM	1272							
204	Treanor	1994	trivalent inactivated IM	30	≥61 years	multicomortibity	outpatients	government/ private no profit	North America	USA	multi center
			placebo	11							

206	Treanor	2011	trivalent inactivated recombinant	2344	18 -60 years	healthy subjects	outpatients	industry	North America	USA	multi center
			placebo	2304							
207	Treanor	2000	trivalent inactivated IM	33	18 -60 years	healthy subjects	outpatients	government/ private no profit	North America	USA	multi center
			trivalent live attenuated nasal spray	36							
			placebo	34							
205	Treanor	2005	trivalent inactivated IM	763	18 -60 years	healthy subjects	outpatients	government/ private no profit	North America	USA	multi center
			placebo	193							
208	Treanor	2007	trivalent inactivated recombinant	306	18 -60 years	healthy subjects	outpatients	industry	North America	USA	multi center
			placebo	154							
209	Treanor cohort 1	2017	trivalent inactivated IM	871	18 -60 years	healthy subjects	outpatients	industry	North America	USA	multi center
			quadrivalent inactivated IM	870							
	Treanor cohort 2	2017	trivalent inactivated IM	871	≥61 years	healthy subjects	outpatients				
			quadrivalent inactivated IM	870							
210	Tsang	2014	trivalent inactivated IM	319	≥61 years	healthy subjects	outpatients	government/ private no profit	North America	USA	multi center
			trivalent inactivated high doses IM	320							
			trivalent inactivated intradermal	637							

211	Van Damme	2010	trivalent inactivated intradermal	398	≥61 years	multicomortibity	outpatients	not reported	Europe	Belgium, France	multi center
			trivalent inactivated adjuvanted MF59/AS03 IM	397							
212	Van de Witte	2018	quadrivalent inactivated (subunit or split) IM	769	18 -60 years	healthy subjects	outpatients	industry	Europe	Belgium, Germany, Hungary, Latvia and Lithuania	multi center
			trivalent inactivated (subunit or split) IM	222	18 -60 years						
			quadrivalent inactivated (subunit or split) IM	769	61 years and older						
			trivalent inactivated (subunit or split) IM	220	61 years and older						
213	Vardeny	2020	trivalent inactivated high doses (subunit or split) IM	2630	61 years and older	multicomortibity	inpatients	industry+government/ private no profit	North America	US and Canada	multi center
			quadrivalent inactivated (subunit or split) IM	2630	61 years and older						
216	Vesikari	2008	trivalent live attenuated nasal spray	61	<5 years	healthy subjects	outpatients	industry	Europe	Finland	multi center
			placebo	59							
219	Vesikari	2009	trivalent inactivated IM	139	<5 years	healthy subjects	outpatients	industry	Europe	Finland	not reported
			trivalent inactivated adjuvante MF59/AS03 IM	130							

217	Vesikari	2011	trivalent inactivated IM	1773	<5 years	healthy subjects	outpatients	industry	Europe	Germany ,Finland	multi center
			trivalent inactivated adjuvanted (subunit or split) MF59/AS03 IM	1941							
214	Vesikari cohort 1	2006	trivalent live attenuated nasal spray	1059	<5 years	healthy subjects	outpatients	industry	Multi-continent	Belgium, Finland, Israel, Spain, UK	multi center
			placebo	725							
	Vesikari cohort 2	2006	trivalent live attenuated nasal spray	658	<5 years	healthy subjects	outpatients				
			placebo	461							
215	Vesikari b	2006	trivalent live attenuated nasal spray	98	<5 years	healthy subjects	outpatients	industry	Europe	Finland	multi center
			placebo	99							
220	Vesikari b	2020	quadrivalent inactivated (subunit or split) IM	230	18 -60 years	pregnant women	outpatients	industry	Europe	Finland	multi center
			trivalent inactivated (subunit or split) IM	116	18 -60 years						
218	Vesikari a	2020	quadrivalent inactivated (subunit or split) IM	402	mixed aged children	healthy subjects	outpatients	industry	Europe	Estonia, Finland, Germany, Hungary, Lithuania, and Poland	multi center
			trivalent inactivated (subunit or split) IM	798	mixed aged children						

221	Victor	2016	trivalent live attenuated nasal spray	1174	<5 years	healthy subjects	outpatients	government/ private no profit	Africa	Senegal	single center
			placebo	587							
222	Volling	2019	trivalent inactivated high doses (subunit or split) IM	87	18 -60 years	healthy subjects	outpatients	industry	North America	Canada	single center
			trivalent inactivated (subunit or split) IM	83	18 -60 years						
223	Wang	2015	trivalent inactivated IM	156	<5 years	healthy subjects	outpatients	industry	North America	USA	multi center
			quadrivalent inactivated IM	158							
224	Wang cohort 1	2017	trivalent inactivated IM	916	mixed aged children	healthy subjects	outpatients	industry	Asia	China	single center
			quadrivalent inactivated IM	913							
	Wang cohort 2	2017	trivalent inactivated IM	460	18 -60 years	healthy subjects	outpatients	industry			
			quadrivalent inactivated IM	458							
	Wang cohort 3	2017	trivalent inactivated IM	456	≥61 years	healthy subjects	outpatients	industry			
			quadrivalent inactivated IM	458							
225	Wang	2020	trivalent live attenuated nasal spray	1000	mixed aged children	healthy subjects	outpatients	industry	Asia	China	multi center
			placebo/no intervention	1000	mixed aged children						

226	Ward b	2020	quadrivalent inactivated recombinant	5077	18 -60 years	healthy subjects	outpatients	industry	Multi continent	Asia, Europe, and North America	multi center
			placebo/no intervention	5083	18 -60 years						
			quadrivalent inactivated recombinant	6396	61 years and older						
			quadrivalent inactivated (subunit or split) IM	6398	61 years and older						
227	Weingarten 1988	1988	trivalent inactivated (subunit or split) IM	91	18 -60 years	healthy subjects	outpatients	not reported	North America	USA	single center
			placebo/no intervention	88	18 -60 years						
228	Wilde cohort 1	1999	trivalent inactivated (subunit or split) IM	52	18 -60 years	healthy subjects	outpatients	industry+government/ private no profit	North America	USA	multi center
	cohort 1		meningococcal vaccine	50	18 -60 years						
	cohort 2		trivalent inactivated (subunit or split) IM	51	18 -60 years						
	cohort 2		pneumococcal vaccine	52	18 -60 years						
	cohort 3		trivalent inactivated (subunit or split) IM	78	18 -60 years						
	cohort 3		placebo/no intervention	78	18 -60 years						
229	Wongsurakiat	2004	trivalent inactivated IM	62	adult and elderly	chronic respiratory disease	outpatients	government/ private no profit	Asia	Thailand	single center
			placebo	63							

230	Zaman	2018	trivalent inactivated (subunit or split) IM	172	18 -60 years	pregnant women	outpatients	government/ private no profit	Asia	Bangladesh	not reported
			pneumococcoal vaccine	168	18 -60 years						
231	Zedda	2015	trivalent inactivated IM	41	<5 years	healthy subjects	outpatients	not reported	Europe	Belgium	multi center
			trivalent inactivated adjuvanted MF59/AS03 IM	43							
**Studies included in the systematic review but not in the network meta-analyses due to not reporting outcome data**											
60	Della Cioppa	2011	17 study groups		<5 years	healthy subjects	outpatients	industry	Europe	Finland, Belgium	multi center
46	Chu	2020	quadrivalent inactivated (subunit or split) IM	1160	mixed aged children; adult and elderly	healthy subjects	outpatients	not reported	Asia	China	multi center
			trivalent inactivated (subunit or split) IM	1160							
78	Fayyaz Jahani	2012	trivalent inactivated IM	70	<5 years	chronic respiratory disease	outpatients	not reported	not reported	not reported	not reported
			placebo	70							
107	Izikson	2013	trivalent inactivated recombinant	3384	18 -60 years	healthy subjects	outpatients	not reported	not reported	not reported	not reported
			placebo	2458							
126	Leung	2014	trivalent inactivated IM	73 tot	18 -60 years	multicomortibity	outpatients	not reported	not reported	not reported	not reported
			trivalent inactivated intradermal								

151	Nace	2015	trivalent inactivated IM	110	≥61 years	healthy subjects	outpatients	industry	North America	Pennsylvania	multi center
			trivalent inactivated high doses IM	95							
167	Principi	2003	trivalent inactivated adjuvanted virosome/liposome	202	<5 years	chronic respiratory disease	inpatients	not reported	Europe	Italy	not reported
			no vaccine	101							
183	Sesay	2018	quadrivalent inactivated adjuvanted virosome/liposome	836	18 -60 years	healthy subjects	outpatients	industry	Europe	Belgium, France, Germany, Poland	multi center
			trivalent inactivated IM	278							
			quadrivalent inactivated adjuvanted virosome/liposome	834	≥61 years						
			trivalent inactivated) IM	277							
187	Sleigh	2000	trivalent inactivated IM	19	18 -60 years	chronic fatigue syndrome	outpatients	government/ private no profit	not reported	not reported	not reported
			placebo	21							
191	Souza	2010	trivalent inactivated IM	47	mixed aged children; adult and elderly	sickle cell disease	outpatients	government/ private no profit	South America	Brazil	Souza
			trivalent inactivated adjuvanted virosome/liposome	43							

193	Stapleton	2020	trivalent inactivated IM	25	18 -60 years	imunodepression (any cause)	outpatients	government/ private no profit	North America	USA	single center
			trivalent inactivated high doses IM	26							

Cochrane risk of bias results across studies

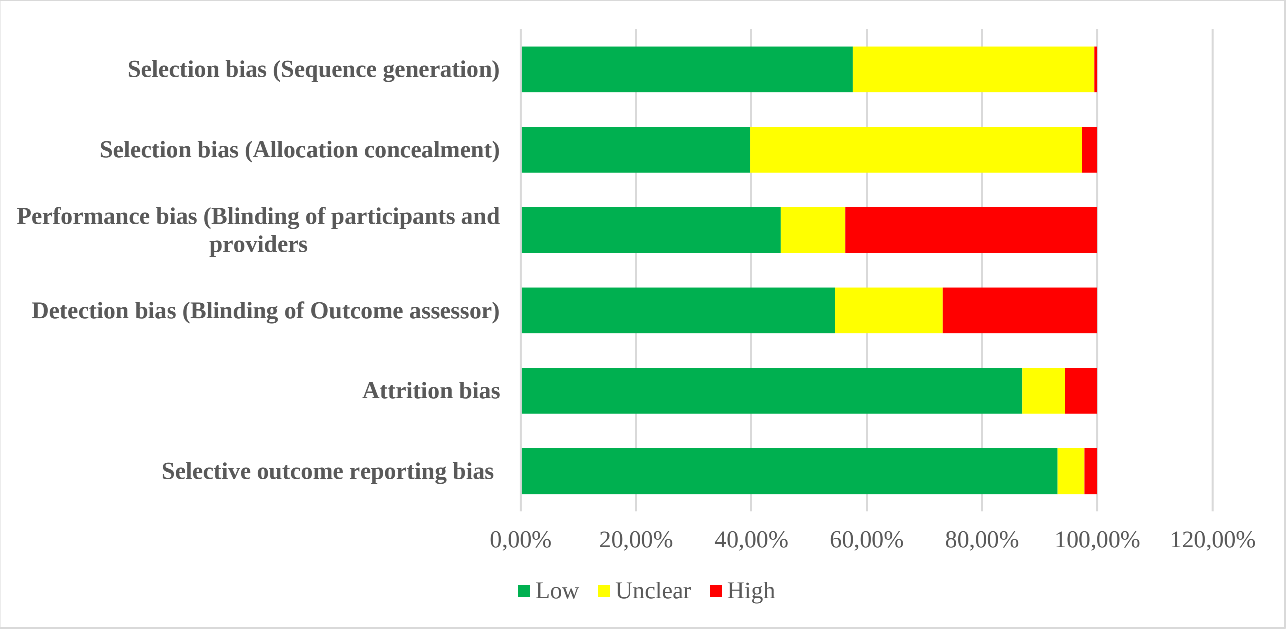

Cochrane risk of bias in individual studies

Author-Year	Selection bias (random sequence generation)	Selection bias (allocation concealment)	Performance bias (blinding of participants and personnel)	Detecton bias (blinding of outcome assessor)	Attrition bias	Selective outcome reporting bias
Abadoglu 2004	Unclear	Unclear	Unclear	Unclear	Low	Low
Allsup 2004	Low	Low	Low	Low	Low	Low
Anh 2016	Unclear	Low	Low	Unclear	Low	Low
Ansaldi 2012	Low	Unclear	High	High	Low	Low
Ansaldi 2013	Unclear	Unclear	High	High	Low	Low
Arnou 2009	Low	Low	High	High	Low	Low
Arnou 2010	Low	Low	High	High	Low	Low
Ashkenazi 2006	Low	Low	High	High	Low	Low
Auewarakul 2006	Unclear	Unclear	High	High	Low	Low
Baldo 2007	Low	Unclear	Low	Low	Low	Low
Baluch 2013	Low	Low	High	High	Low	Low
Barrett 2011	Low	Low	Low	Low	Low	Low
Bart 2016	Low	Low	Low	Low	Low	Low
Baxter 2011	Unclear	Unclear	High	High	Low	Low
Belongia 2020	Low	high	high	High	Low	High
Belshe 1992	Unclear	Unclear	Low	Low	Low	Low
Belshe 1998	Low	Low	Low	Low	Low	Low
Belshe 2000	Unclear	Unclear	Low	Low	Unclear	Low
Belshe 2007	Low	Low	Low	Low	Low	Low
Belshe 2007 b	Low	Unclear	High	High	Low	Low
Ben-Yehuda 2003	Unclear	Unclear	Low	Unclear	Low	Low
Ben-Yehuda b 2003	Unclear	Unclear	Unclear	Unclear	Low	Low
Beran 2009	Low	Low	Low	Low	Low	Low
Beran 2009 b	Unclear	Unclear	Low	Low	Low	High
Beran 2009 c	Low	Low	High	High	Low	Low
Beran 2013	Low	Low	Low	Low	Low	Low
Bergen 2004	Unclear	Unclear	Low	Low	Low	Low
Block 2011	Unclear	Unclear	Low	Low	Low	Low
Block 2012	Low	Low	Low	Low	Low	Low
Boonnak 2017	Unclear	Unclear	High	High	Low	Low
Bracco Neto 2009	Unclear	Unclear	Low	Low	Low	Low
Brady 2018	unclear	unclear	Low	low	Low	Low

Bridges 2000	Low	Low	Low	Low	Low	Low
Brooks 2016	Low	Low	Low	Low	Low	Low
Bueving 2004	Low	Low	Low	Low	Low	Low
Cadorna-Carlos 2015	Low	Low	High	High	Low	Low
Caldera 2020	Low	unclear	Low	Low	Low	Low
Carr 2011	Low	Unclear	High	High	Low	Low
Chan 2014	Low	Unclear	High	High	Low	Low
Chang 2019	Low	Unclear	high	Low	Low	Low
Chi 2010	Low	Unclear	High	High	Low	Low
Chiu 2007	Low	High	High	High	Low	Low
Chiu 2009	Low	Unclear	High	High	Low	Low
Choi 2017	Low	Low	Low	Low	Low	Low
Choi 2018	Unclear	Unclear	High	High	Low	Low
Chu 2020	Low	Low	Low	Low	Low	Low
Chuaychoo 2020	Low	unclear	high	High	unclear	Low
Claeys 2018	Low	Low	high	Low	High	Low
Colmegna 2020l	Low	Low	high	Low	Low	Low
Colombo 2001	Low	Unclear	High	High	Low	Low
Couch 2007	Unclear	High	High	Unclear	Low	Low
Cowling 2010	Low	Low	Low	Low	Low	Low
Cowling 2014	Low	Low	Low	Low	Low	Low
Cowling 2020	Low	Low	high	Low	High	Low
Cruz-Valdez 2017	Low	Unclear	High	High	Low	Low
De Bruijn 2006	Unclear	Unclear	High	High	Low	Low
De Bruijn 2007	Low	Unclear	High	High	Low	Low
De Donato 1999	Unclear	Unclear	High	Unclear	Unclear	Low
De Villiers 2009	Low	Unclear	Low	Low	Low	Low
Della Cioppa 2011	Unclear	Unclear	Unclear	Unclear	Low	Low
Desheva 2002	Unclear	Unclear	Unclear	Unclear	Low	Unclear
Diallo 2018	Low	unclear	Low	Low	High	Low
DiazGranados 2013	Low	Low	Low	Low	Low	Low
DiazGranados 2014	Low	Low	Low	Low	Low	Low
DiazGranados 2015	Low	Low	High	Low	Low	Low
Domachowske 2013	Low	Low	Low	Low	Low	Low
Dunkle 2017	Low	Low	Low	Low	Low	Low
Dunkle 2017 b	Low	Low	High	Low	Low	Low
Dunkle 2018	unclear	high	high	Low	Low	High

Durando 2008	Unclear	Unclear	High	High	Low	Low
Englund 2010	Low	Low	Low	Low	Low	Low
Esposito 2010	Unclear	Unclear	High	High	Low	Low
Esposito 2011	Low	Low	High	High	Low	Low
Esposito 2014	Low	Unclear	High	Unclear	Low	Low
Essink 2020	Low	Low	Low	Low	Low	Low
Evison 2009	Low	Unclear	Low	Low	Low	Low
Falsey 2009	Low	Unclear	Low	Low	Low	Low
Fayyaz Jahani 2012	Unclear	Unclear	Unclear	Unclear	Unclear	Unclear
Fleming 2006	Unclear	Low	High	High	Low	Low
Forrest 2011	Unclear	Unclear	High	High	Low	Low
Frenck 2011	Unclear	Unclear	High	High	Low	Low
Frey 2003	Unclear	Unclear	High	Unclear	Low	Low
Frey 2010	Unclear	Low	High	Low	Low	Low
Frey2014	Unclear	Low	High	Unclear	Low	Low
Gabutti 2005	Unclear	Unclear	Unclear	Unclear	High	Low
Gasparini 2001	Unclear	Unclear	Low	Low	Low	Low
Giaquinta 2014	Unclear	Unclear	Low	Low	Low	Low
Gorse 2013	Unclear	Unclear	High	High	Low	Low
Gorse 2013 b	Low	Low	High	High	Low	Low
Gorse 2015	Unclear	Low	Low	Low	Unclear	Low
Govaert 1993	Low	Low	Low	Low	Low	Low
Greenberg 2013	Unclear	Low	High	High	Low	Low
Greenberg 2014	Unclear	Low	High	Unclear	High	Low
Greenberg 2017	Unclear	Low	Low	Low	Low	Low
Hakim 2016	Low	Unclear	High	High	Low	Low
Halasa 2011	Unclear	Unclear	Low	Low	Low	Low
Halasa 2016	Low	Unclear	High	High	Low	Low
Hartvickson 2015	Unclear	Low	Low	Low	Low	Low
Hobermann 2003	Low	Low	High	Unclear	Low	Low
Hoft 2011	Unclear	Unclear	High	High	Low	Low
Holland 2008	Low	Unclear	High	High	Low	Low
Hoon Han 2013	Unclear	Unclear	High	High	Low	Low
Hu 2020	Low	Low	Low	Low	Low	Low
Hui 2006	Low	Unclear	High	Unclear	Low	Low
Hung 2009	unclear	unclear	high	High	High	Low
Hurwitz 2000	unclear	unclear	high	unclear	High	Low

Izikson 2013	Unclear	Unclear	Unclear	Unclear	Unclear	Unclear
Jackson 2010	Low	Low	High	Unclear	Low	Low
Jamshed 2019	unclear	Low	Low	Low	Low	Low
Jeffs 2015	Low	Unclear	High	High	Low	Low
Kanra 2004	Unclear	Unclear	High	High	Low	Low
Keitel 1997	Unclear	Unclear	Low	Low	Unclear	Low
Keitel 2006	Unclear	Unclear	Unclear	Unclear	Low	Low
Keitel 2009	Unclear	Unclear	Low	Low	Low	Low
Khajedaluee 2018	high	high	high	High	Low	Low
Khan 1996	Low	Unclear	Low	Low	Low	Low
Kieninger 2013	Low	Low	Low	Low	Low	Low
King 1998	Low	Unclear	Low	Low	Low	Low
King 2000	Unclear	Unclear	Unclear	Unclear	Low	Low
Kositanont 2004	Unclear	Unclear	Unclear	Unclear	Low	Low
Kumar 2016	Low	Low	High	Low	Low	Low
Lan 2019	unclear	unclear	unclear	unclear	Low	Low
Langley 2013	Low	Low	Low	Low	Low	Low
Langley 2014	Low	Low	Low	Low	Low	Low
Leroux-Roels	Unclear	Unclear	High	High	Low	Low
Leung 2014	Unclear	Unclear	High	High	Unclear	Low
Levin 2008	Unclear	Unclear	High	High	Low	Low
Levin 2016	Unclear	Unclear	High	High	Low	Low
Li 2008	Low	Unclear	High	Unclear	Low	Low
Liebowitz 2020	Low	Low	Low	Low	Low	Low
Li-Kim-Moy 2016	Low	unclear	high	Low	Low	Low
Lum 2010	Low	Low	Low	Low	Low	Low
Madhi 2011	Low	Low	Low	Low	Low	Low
Madhi 2013	Low	Low	Low	Low	Low	Low
Madhi 2014	Low	Unclear	Low	Low	Unclear	Low
Magnani 2005	Unclear	Unclear	High	High	Low	Low
Mallory 2018	unclear	unclear	unclear	unclear	Low	Low
Mallory 2020	unclear	unclear	unclear	unclear	Low	low
Marchisio 2009	Unclear	Unclear	High	High	Low	Low
McBride 2016	Low	Unclear	High	Low	Low	Low
McElhaney	Low	Unclear	High	Low	Low	Low
McKittrick 2013	Low	Low	Low	Low	Low	Low
McManus 2014	Unclear	Unclear	Low	Low	Unclear	Unclear

Mesa –Duque 2001	Low	Low	Low	Low	Unclear	low
Minutello 1999	Unclear	Unclear	High	Unclear	Low	Low
Mixeu 2002	Unclear	Unclear	Low	Low	Low	Low
Miyazaki 1993	Unclear	Unclear	Unclear	Unclear	Low	Unclear
Monto 2009	Unclear	Unclear	Low	Low	Low	Low
Morelon 2010	Low	Unclear	High	High	Low	Low
Musto 1995	Unclear	Unclear	High	High	Unclear	Unclear
Nace 2015	Low	Unclear	High	High	High	High
Natori 2017	Low	Unclear	High	Low	Low	Low
Natori 2018	Low	Low	High	Low	High	Unclear
Nichol 1995	Low	Unclear	Low	Low	Low	Low
Nichol 1999	Low	Low	Low	Low	Low	Low
Noh 2019	Low	Low	high	Low	Low	Low
Nolan 2014	Unclear	Low	High	Unclear	Low	Low
Ohmit 2006	Low	Unclear	Low	Low	Low	Low
Ohmit 2008	Unclear	Unclear	Low	Low	Low	Low
Ortiz 2015	Unclear	Unclear	Low	Low	Low	Low
Pedroza 2009	Unclear	Unclear	Unclear	Low	Low	Low
Pepin 2013	Low	Low	Low	Low	Low	Low
Pepin 2016	Low	Low	Low	Low	Low	Low
Pepin 2019	Low	Low	high	High	High	Low
Powers 1995	Unclear	Unclear	Low	Low	Unclear	Unclear
Pregliasco 2001	Unclear	Unclear	High	Unclear	Unclear	Low
Principi 2003	Unclear	Unclear	High	High	Unclear	Unclear
Redding 2002	Low	Unclear	Low	Low	Low	Low
Reid 1998	Unclear	Unclear	Low	Low	Low	Unclear
Rodriguez Weber 2014	Low	Low	Unclear	Unclear	Low	Low
Rolfes 2017	Low	Low	Low	Low	Low	Low
Rouphael 2017	Low	Unclear	High	High	Low	Low
Rudenko 2001	Unclear	Unclear	Low	Low	Low	Low
Ruf2004	Unclear	Unclear	High	High	Low	Low
Safdar 2006	Unclear	Unclear	Unclear	Unclear	Low	Low
Sarkar 2020	Low	unclear	high	unclear	Low	Low
Sarsenbayeva 2018	Low	Low	Low	Low	Low	Low
Saxen 1999	Unclear	Unclear	Unclear	Unclear	Low	Low
Schaad 2000	Unclear	Unclear	High	High	Low	Low
Scheifele 2013	Low	Unclear	High	Low	Low	Low

Seo 2014	Unclear	Unclear	High	High	Low	Low
Seo 2016	Unclear	Unclear	High	High	Low	Low
Sesay 2018	Low	Low	Low	Low	Low	Low
Sharma 2018	Low	unclear	high	unclear	Low	Low
Sheldon 2013	Low	Low	Unclear	Unclear	Low	Low
Sindoni 2009	Low	unclear	unclear	unclear	Low	Low
Sleigh 2000	Low	Low	Low	Low	Low	Low
Solares 2014	Unclear	Unclear	High	Unclear	Low	Low
Song 2007	Low	unclear	high	Low	High	Low
Song 2019	Low	unclear	Low	Low	Low	Low
Souza 2010	Unclear	Unclear	High	High	High	Low
Squarcion 2003	unclear	unclear	high	high	Low	Low
Stapleton 2020	Low	Low	Low	Low	Low	Low
Steinhoff 2017	Low	Low	High	Low	Low	Low
Stevanovic 2018	Unclear	Low	Low	Low	Low	Low
Stevanovic 2020	unclear	unclear	Low	Low	Low	Low
Strijbos 2019	Low	unclear	high	Low	Low	Low
Swierkosz 1994	Unclear	Unclear	Low	Low	Unclear	Low
Tam 2007	Low	Low	Low	Low	Low	Low
Tanaka 1993	Unclear	Unclear	Unclear	Unclear	Unclear	Low
Tapia 2016	Low	Low	Low	Low	Low	Low
Tasker 1999	Low	Low	Low	Low	Low	Low
Tinoco 2014	Low	Low	Low	Low	Low	Low
Treanor 1994	Unclear	Unclear	Unclear	Unclear	Low	Low
Treanor 2000	Unclear	Unclear	Low	Low	Low	Low
Treanor 2005	Low	Unclear	Low	Low	Low	Low
Treanor 2007	Low	Unclear	Low	Low	Low	Low
Treanor 2011	Low	Unclear	Low	Low	Low	Low
Treanor 2017	Low	Low	Low	Low	Low	Low
Tsang 2014	Unclear	Low	High	High	Low	Low
Van Damme 2010	Low	Low	High	High	Low	Low
van de Witte 2018	Low	Low	Low	Low	Low	Low
Vardeny 2020	Low	Low	unclear	Low	Low	Low
Vesikari 2006	Low	Low	Low	Low	Low	Low
Vesikari 2006 b	Unclear	Unclear	Low	Low	Low	Low
Vesikari 2008	Unclear	Unclear	Low	Low	Low	Low
Vesikari 2009	Low	Unclear	Unclear	Low	Low	Unclear

Vesikari 2011	Unclear	Unclear	Unclear	Unclear	Low	Low
Vesikari 2020	Low	Low	Low	Low	Low	Low
Vesikari 2020 b	Low	unclear	high	Low	Low	Low
Victor 2016	Low	Low	Low	Low	Low	Low
Volling 2019	Low	high	Low	Low	Low	High
Wang 2015	Low	Unclear	High	Unclear	Low	Low
Wang 2017	Low	Low	Low	Low	Low	Low
Wang 2020	Low	Low	Low	Low	Low	Low
Ward 2020	Low	Low	high	Low	Low	Low
Weingarten 1988	Low	unclear	Low	Low	Low	Low
Wilde 1999	Low	Low	Low	Low	Low	Low
Wongsurakiat 2004	Low	Low	Low	Low	Low	Low
Zaman 2008	Low	Low	Low	Low	High	Low
Zedda	Unclear	Unclear	Unclear	Unclear	Low	Low

Pairwise meta-analyses

Comparison	Outcome	N. studies	N. patients	RR (95% CI)	I^2 RR	ARD (95%CI)	I^2ARD
**All adults and the elderly**							
placebo vs 3-IIV	Infl Lab Conf	31	74282	0.42 (0.36, 0.49)	48.86%	-0.03 (-0.04, -0.02)	**89.52%**
placebo vs 3-RIV	Infl Lab Conf	3	5209	0.28 (0.09, 0.89)	57.40%	-0.03 (-0.05, 0.00)	46.52%
placebo vs 4-IIV	Infl Lab Conf	2	1946	0.52 (0.39, 0.69)	0.00%	-0.13 (-0.33, 0.08)	**78.77%**
placebo vs 3-LAIV	Infl Lab Conf	6	6590	0.58 (0.48, 0.71)	0.00%	-0.04 (-0.06, -0.01)	66.82%
3-IIV vs 3-IIV HD	Infl Lab Conf	3	41333	0.76 (0.64, 0.90)	0.00%	-0.00 (-0.01, 0.00)	**79.88%**
3-IIV vs 3-IIV MF59/AS03-adj	Infl Lab Conf	2	43768	0.91 (0.68, 1.22)	6.58%	-0.00 (-0.00, 0.00)	0.00%
3-IIV vs 3-RIV	Infl Lab Conf	3	1575	1.10 (0.51, 2.37)	6.64%	0.00 (-0.01, 0.02)	3.96%
3-IIV vs 3-LAIV	Infl Lab Conf	7	7923	1.63 (1.24, 2.15)	0.00%	0.01 (0.00, 0.02)	42.14%
4-RIVvs 4-IIV	Infl Lab Conf	2	21797	0.80 (0.62, 1.03)	52.84%	-0.01 (-0.01, 0.00)	69.41%
placebo vs 3-IIV	Systemic AE	30	43713	1.18 (1.06, 1.31)	68.17%	0.03 (0.01, 0.05)	**84.43%**
placebo vs 3-IIV ID	Systemic AE	2	97	4.93 (0.81, 30.09)	0.00%	0.26 (-0.08, 0.61)	**76.09%**
placebo vs 3-RIV	Systemic AE	3	5209	0.97 (0.86, 1.09)	0.00%	-0.00 (-0.02, 0.02)	0.00%
placebo vs 4-IIV	Systemic AE	2	1946	0.64 (0.19, 2.16)	**90.52%**	-0.12 (-0.41, 0.17)	**90.17%**
placebo vs 3-LAIV	Systemic AE	7	5384	1.23 (1.12, 1.35)	0.00%	0.06 (0.03, 0.08)	0.00%
3-IIV vs 3-IIV HD	Systemic AE	10	6016	1.21 (1.10, 1.34)	16.90%	0.06 (0.03, 0.09)	11.39%
3-IIV vs 3-IIV ID	Systemic AE	24	19330	1.01 (0.94, 1.09)	38.07%	0.01 (-0.01, 0.03)	44.23%
3-IIV vs 3-IIV MF59/AS03-adj	Systemic AE	23	57933	1.25 (1.13, 1.39)	35.76%	0.02 (0.01, 0.04)	74.54%
3-IIV vs 3-IIV vir/lip-adj	Systemic AE	7	1940	0.83 (0.65, 1.05)	0.00%	-0.02 (-0.06, 0.02)	67.30%
3-IIV vs 3-RIV	Systemic AE	4	1602	0.97 (0.77, 1.22)	0.00%	0.00 (-0.03, 0.04)	0.00%

Comparison	Outcome	N. studies	N. patients	RR (95% CI)	I^2 RR	ARD (95%CI)	I^2ARD
3-IIV vs 4-IIV	Systemic AE	18	23154	1.03 (0.98, 1.08)	0.00%	0.01 (-0.00, 0.02)	0.00%
3-IIV vs 3-LAIV	Systemic AE	6	6151	1.13 (1.05, 1.22)	0.00%	0.04 (0.01, 0.06)	0.00%
3-IIV HD vs 4-IIV	Systemic AE	3	6318	1.00 (0.86, 1.16)	0.00%	-0.00 (-0.01, 0.01)	0.00%
3-IIV ID vs 3-IIV MF59/AS03-adj	Systemic AE	4	1766	0.93 (0.78, 1.11)	0.00%	0.00 (-0.02, 0.02)	0.00%
3-IIV ID vs 3-IIV vir/lip-adj	Systemic AE	2	623	0.93 (0.50, 1.72)	0.00%	-0.00 (-0.04, 0.03)	0.00%
3-IIV MF59/AS03-adj vs 3-IIV vir/lip-adj	Systemic AE	5	1605	0.74 (0.54, 1.02)	29.26%	-0.04 (-0.14, 0.05)	**91.23%**
3-IIV MF59/AS03-adj vs 4-IIV	Systemic AE	2	1226	0.84 (0.64, 1.10)	0.00%	-0.04 (-0.24, 0.16)	**88.45%**
4-IIV MF59/AS03-adj vs 3-IIV MF59/AS03-adj	Systemic AE	2	1988	1.10 (0.92, 1.31)	0.00%	0.01 (-0.02, 0.04)	0.00%
4-RIVvs 4-IIV	Systemic AE	4	23990	1.07 (0.95, 1.20)	28.40%	0.01 (-0.04, 0.05)	**97.67%**
3-LAIV vs 4-LAIV	Systemic AE	2	3600	0.99 (0.92, 1.07)	0.00%	-0.00 (-0.04, 0.03)	0.00%
placebo vs 3-IIV	Local AE	30	39906	2.78 (2.26, 3.43)	**92.89%**	0.26 (0.20, 0.32)	**98.09%**
placebo vs 3-IIV ID	Local AE	2	97	2.48 (0.63, 9.70)	0.00%	0.10 (-0.03, 0.24)	0.00%
placebo vs 3-RIV	Local AE	3	5209	3.81 (2.66, 5.47)	57.62%	0.25 (0.14, 0.37)	**85.81%**
placebo vs 4-IIV	Local AE	2	1946	3.86 (0.89, 16.81)	61.03%	0.19 (-0.00, 0.37)	**89.36%**
placebo vs 3-LAIV	Local AE	7	9875	1.76 (1.65, 1.87)	0.00%	0.18 (0.16, 0.21)	37.44%
3-IIV vs 3-IIV HD	Local AE	9	5915	1.48 (1.34, 1.63)	13.63%	0.12 (0.08, 0.16)	49.15%
3-IIV vs 3-IIV ID	Local AE	25	19551	1.54 (1.31, 1.82)	**93.38%**	0.16 (-0.01, 0.32)	**99.72%**
3-IIV vs 3-IIV MF59/AS03-adj	Local AE	21	57818	1.84 (1.60, 2.12)	**76.25%**	0.13 (0.09, 0.17)	**94.88%**
3-IIV vs 3-IIV vir/lip-adj	Local AE	7	1940	1.36 (1.05, 1.74)	37.19%	0.08 (-0.01, 0.17)	**88.89%**
3-IIV vs 3-RIV	Local AE	4	1602	0.61 (0.33, 1.10)	**79.89%**	-0.08 (-0.18, 0.02)	**75.32%**
3-IIV vs 4-IIV	Local AE	19	24857	1.14 (1.08, 1.21)	53.35%	0.04 (0.02, 0.06)	**84.27%**

Comparison	Outcome	N. studies	N. patients	RR (95% CI)	I^2 RR	ARD (95%CI)	I^2ARD
3-IIV vs 3-LAIV	Local AE	5	6082	1.25 (0.69, 2.25)	**98.00%**	0.04 (-0.13, 0.21)	**97.57%**
3-IIV HD vs 4-IIV	Local AE	3	6318	0.99 (0.81, 1.20)	0.00%	-0.00 (-0.01, 0.01)	0.00%
3-IIV ID vs 3-IIV MF59/AS03-adj	Local AE	4	1766	1.03 (0.56, 1.90)	**86.66%**	-0.03 (-0.15, 0.08)	**92.71%**
3-IIV ID vs 3-IIV vir/lip-adj	Local AE	2	623	0.50 (0.39, 0.64)	0.00%	-0.26 (-0.61, 0.09)	**94.94%**
3-IIV MF59/AS03-adj vs 3-IIV vir/lip-adj	Local AE	5	1605	0.57 (0.45, 0.73)	39.04%	-0.15 (-0.34, 0.04)	**96.67%**
3-IIV MF59/AS03-adj vs 4-IIV	Local AE	2	1226	1.02 (0.86, 1.21)	0.00%	-0.00 (-0.02, 0.01)	0.00%
4-IIV MF59/AS03-adj vs 3-IIV MF59/AS03-adj	Local AE	2	1988	1.07 (0.92, 1.23)	0.00%	0.01 (-0.05, 0.06)	29.05%
4-RIVvs 4-IIV	Local AE	4	23990	1.04 (0.80, 1.35)	**96.54%**	0.01 (-0.05, 0.07)	**97.12%**
3-LAIV vs 4-LAIV	Local AE	2	3600	0.95 (0.73, 1.24)	**88.64%**	-0.01 (-0.11, 0.08)	**87.90%**
placebo vs 3-IIV	ILI	27	42312	0.81 (0.74, 0.90)	73.74%	-0.04 (-0.06, -0.02)	**81.33%**
placebo vs 3-RIV	ILI	2	4749	0.77 (0.62, 0.96)	0.00%	-0.02 (-0.03, -0.00)	0.00%
placebo vs 4-IIV	ILI	2	1946	0.79 (0.63, 0.98)	0.00%	-0.05 (-0.16, 0.05)	41.94%
placebo vs 3-LAIV	ILI	2	7803	0.95 (0.90, 1.00)	0.00%	-0.02 (-0.03, 0.00)	0.00%
3-IIV vs 3-RIV	ILI	3	1575	0.98 (0.66, 1.46)	0.00%	-0.00 (-0.03, 0.02)	0.00%
placebo vs 3-IIV	Mortality	11	26844	1.16 (0.45, 3.01)	0.00%	0.00 (-0.00, 0.00)	0.00%
placebo vs 3-LAIV	Mortality	2	7803	0.57 (0.08, 3.97)	0.00%	-0.00 (-0.00, 0.00)	43.43%
3-IIV vs 3-IIV HD	Mortality	4	36165	1.00 (0.75, 1.33)	0.00%	0.00 (-0.00, 0.00)	0.00%
3-IIV vs 3-IIV MF59/AS03-adj	Mortality	3	50867	0.89 (0.60, 1.32)	42.45%	-0.00 (-0.00, 0.00)	0.00%
3-IIV vs 4-IIV	Mortality	6	9362	1.67 (0.55, 5.04)	0.00%	0.00 (-0.00, 0.00)	0.00%
4-RIVvs 4-IIV	Mortality	2	21797	0.69 (0.39, 1.22)	0.00%	-0.00 (-0.00, 0.00)	0.00%
placebo vs 3-IIV	Hospitalization	11	4794	0.44 (0.25, 0.79)	0.00%	-0.01 (-0.02, 0.01)	**88.71%**

Comparison	Outcome	N. studies	N. patients	RR (95% CI)	I^2 RR	ARD (95%CI)	I^2ARD
placebo vs 3-LAIV	Hospitalization	2	7803	0.78 (0.39, 1.58)	0.00%	-0.00 (-0.00, 0.00)	0.00%
3-IIV vs 3-IIV HD	Hospitalization	3	472	0.49 (0.23, 1.04)	0.00%	-0.03 (-0.08, 0.03)	69.04%
3-IIV vs 3-IIV MF59/AS03-adj	Hospitalization	3	43836	0.92 (0.70, 1.21)	0.00%	-0.00 (-0.00, 0.00)	0.00%
**The elderly (≥61 years)**							
placebo vs 3-IIV	Infl Lab Conf	3	453	0.58 (0.20, 1.67)	**80.51%**	-0.07 (-0.21, 0.07)	**75.49%**
placebo vs 3-LAIV	Infl Lab Conf	2	3466	0.57 (0.44, 0.75)	0.00%	-0.03 (-0.05, -0.02)	0.00%
3-IIV vs 3-IIV HD	Infl Lab Conf	2	41161	0.76 (0.64, 0.90)	0.00%	-0.00 (-0.01, 0.00)	**89.58%**
3-IIV vs 3-LAIV	Infl Lab Conf	2	3218	1.43 (0.81, 2.54)	0.00%	0.01 (-0.00, 0.01)	0.00%
placebo vs 3-IIV	Systemic AE	5	1124	0.88 (0.77, 1.01)	0.00%	-0.03 (-0.08, 0.03)	20.41%
placebo vs 3-LAIV	Systemic AE	2	3287	1.19 (1.06, 1.34)	0.00%	0.05 (0.02, 0.08)	0.00%
3-IIV vs 3-IIV HD	Systemic AE	4	5030	1.23 (1.05, 1.43)	30.67%	0.05 (0.01, 0.10)	44.15%
3-IIV vs 3-IIV ID	Systemic AE	10	6880	1.08 (0.98, 1.20)	4.39%	0.02 (0.00, 0.04)	0.13%
3-IIV vs 3-IIV MF59/AS03-adj	Systemic AE	12	55886	1.24 (1.06, 1.43)	57.91%	0.02 (0.00, 0.04)	**82.98%**
3-IIV vs 3-IIV vir/lip-adj	Systemic AE	3	765	0.93 (0.58, 1.50)	0.00%	0.01 (-0.02, 0.04)	27.71%
3-IIV vs 4-IIV	Systemic AE	4	4319	1.06 (0.92, 1.23)	0.00%	0.01 (-0.01, 0.03)	0.00%
3-IIV vs 3-LAIV	Systemic AE	2	3073	1.11 (0.99, 1.25)	0.00%	0.03 (-0.00, 0.06)	0.00%
3-IIV HD vs 4-IIV	Systemic AE	2	6278	0.97 (0.83, 1.13)	0.00%	-0.00 (-0.01, 0.01)	0.00%
3-IIV ID vs 3-IIV MF59/AS03-adj	Systemic AE	4	1766	0.93 (0.78, 1.11)	0.00%	0.00 (-0.02, 0.02)	0.00%
3-IIV ID vs 3-IIV vir/lip-adj	Systemic AE	2	623	0.93 (0.50, 1.72)	0.00%	-0.00 (-0.04, 0.03)	0.00%
3-IIV MF59/AS03-adj vs 3-IIV vir/lip-adj	Systemic AE	3	805	0.81 (0.40, 1.66)	38.54%	-0.03 (-0.23, 0.16)	**94.30%**
4-RIVvs 4-IIV	Systemic AE	2	13637	1.13 (1.06, 1.20)	0.00%	0.02 (-0.01, 0.05)	**90.01%**

Comparison	Outcome	N. studies	N. patients	RR (95% CI)	I^2 RR	ARD (95%CI)	I^2ARD
placebo vs 3-IIV	Local AE	4	1023	3.13 (1.68, 5.85)	8.69%	0.13 (0.05, 0.21)	63.61%
placebo vs 3-LAIV	Local AE	2	3287	1.82 (1.64, 2.03)	0.00%	0.12 (-0.03, 0.27)	**81.00%**
3-IIV vs 3-IIV HD	Local AE	3	4929	1.55 (1.33, 1.80)	47.38%	0.14 (0.09, 0.19)	50.80%
3-IIV vs 3-IIV ID	Local AE	11	7101	1.74 (1.38, 2.19)	73.46%	0.16 (0.00, 0.31)	**98.58%**
3-IIV vs 3-IIV MF59/AS03-adj	Local AE	12	55886	1.89 (1.58, 2.26)	**76.08%**	0.10 (0.05, 0.14)	**95.82%**
3-IIV vs 3-IIV vir/lip-adj	Local AE	3	765	1.12 (0.73, 1.72)	0.00%	0.01 (-0.01, 0.02)	0.00%
3-IIV vs 4-IIV	Local AE	4	4319	1.22 (1.01, 1.46)	26.40%	0.03 (0.01, 0.04)	0.00%
3-IIV vs 3-LAIV	Local AE	2	3073	0.76 (0.04, 12.85)	**86.92%**	0.03 (-0.35, 0.42)	**95.88%**
3-IIV HD vs 4-IIV	Local AE	2	6278	0.98 (0.80, 1.21)	0.00%	-0.00 (-0.01, 0.01)	0.00%
3-IIV ID vs 3-IIV MF59/AS03-adj	Local AE	4	1766	1.03 (0.56, 1.90)	**86.66%**	-0.03 (-0.15, 0.08)	**92.71%**
3-IIV ID vs 3-IIV vir/lip-adj	Local AE	2	623	0.50 (0.39, 0.64)	0.00%	-0.26 (-0.61, 0.09)	**94.94%**
3-IIV MF59/AS03-adj vs 3-IIV vir/lip-adj	Local AE	3	805	0.60 (0.38, 0.97)	59.05%	-0.12 (-0.45, 0.21)	**97.13%**
4-RIVvs 4-IIV	Local AE	2	13637	0.84 (0.23, 3.02)	68.13%	0.03 (-0.09, 0.15)	**99.26%**
placebo vs 3-IIV	ILI	2	854	0.42 (0.19, 0.96)	0.00%	-0.07 (-0.32, 0.17)	**93.21%**
3-IIV vs 3-IIV HD	Mortality	2	35865	1.00 (0.75, 1.34)	0.00%	0.00 (-0.00, 0.00)	0.00%
3-IIV vs 3-IIV MF59/AS03-adj	Mortality	2	50799	0.88 (0.57, 1.35)	65.16%	-0.00 (-0.00, 0.00)	4.85%
3-IIV vs 4-IIV	Mortality	2	1664	2.43 (0.12, 47.37)	0.00%	0.00 (-0.00, 0.01)	0.00%
placebo vs 3-IIV	Hospitalization	3	982	0.35 (0.13, 0.90)	0.00%	-0.05 (-0.22, 0.12)	**95.10%**
**Adults and the Elderly -Immunocompromised/cancer participants**							
placebo vs 3-IIV	Infl Lab Conf	3	802	0.24 (0.09, 0.64)	44.50%	-0.13 (-0.28, 0.02)	**90.06%**
placebo vs 3-IIV	Systemic AE	4	768	1.17 (0.88, 1.56)	0.00%	0.03 (-0.06, 0.12)	59.03%

Comparison	Outcome	N. studies	N. patients	RR (95% CI)	I^2 RR	ARD (95%CI)	I^2ARD
3-IIV vs 3-IIV HD	Systemic AE	4	516	1.16 (0.86, 1.57)	31.63%	0.07 (-0.00, 0.13)	0.00%
3-IIV vs 3-IIV ID	Systemic AE	3	206	2.21 (0.58, 8.41)	59.08%	0.12 (0.04, 0.21)	0.00%
3-IIV vs 3-IIV MF59/AS03-adj	Systemic AE	5	315	1.45 (0.78, 2.70)	0.00%	0.01 (-0.03, 0.05)	0.00%
placebo vs 3-IIV	Local AE	4	778	1.54 (0.78, 3.05)	47.10%	0.12 (-0.08, 0.31)	**87.45%**
3-IIV vs 3-IIV HD	Local AE	4	516	1.39 (0.97, 1.99)	0.00%	0.06 (-0.00, 0.12)	14.05%
3-IIV vs 3-IIV ID	Local AE	3	206	3.16 (1.07, 9.27)	**77.16%**	0.39 (0.29, 0.50)	0.00%
3-IIV vs 3-IIV MF59/AS03-adj	Local AE	3	200	1.87 (1.02, 3.44)	0.00%	0.09 (-0.05, 0.24)	53.69%
placebo vs 3-IIV	ILI	4	852	0.68 (0.48, 0.95)	26.07%	-0.13 (-0.30, 0.04)	**85.35%**
placebo vs 3-IIV	Mortality	2	537	0.57 (0.07, 4.79)	0.00%	-0.00 (-0.02, 0.01)	0.00%
3-IIV vs 3-IIV HD	Mortality	2	300	1.00 (0.06, 15.84)	0.00%	0.00 (-0.02, 0.02)	0.00%
placebo vs 3-IIV	Hospitalization	3	658	0.42 (0.11, 1.61)	33.07%	-0.05 (-0.14, 0.04)	**91.29%**
3-IIV vs 3-IIV HD	Hospitalization	3	472	0.49 (0.23, 1.04)	0.00%	-0.03 (-0.08, 0.03)	69.04%
3-IIV vs 3-IIV MF59/AS03-adj	Hospitalization	2	141	0.83 (0.41, 1.67)	0.00%	-0.02 (-0.13, 0.09)	0.00%
**Adults and elderly with chronic respiratory diseases**							
placebo vs 3-IIV	Infl Lab Conf	2	248	0.59 (0.11, 3.31)	**88.74%**	-0.06 (-0.36, 0.23)	**87.81%**
placebo vs 3-IIV	ILI	2	253	0.50 (0.36, 0.68)	0.00%	-0.24 (-0.43, -0.05)	68.05%
placebo vs 3-IIV	Hospitalization	2	253	0.31 (0.08, 1.22)	0.00%	-0.05 (-0.10, 0.01)	0.00%
**All children**							
placebo vs 3-IIV	Infl Lab Conf	8	7561	0.60 (0.48, 0.76)	31.20%	-0.04 (-0.07, -0.01)	70.51%
placebo vs 4-IIV	Infl Lab Conf	2	17454	0.51 (0.46, 0.56)	0.00%	-0.05 (-0.06, -0.04)	26.82%
placebo vs 3-LAIV	Infl Lab Conf	11	17704	0.29 (0.18, 0.47)	**94.04%**	-0.09 (-0.13, -0.05)	**94.07%**

Comparison	Outcome	N. studies	N. patients	RR (95% CI)	I^2 RR	ARD (95%CI)	I^2ARD
3-IIV vs 3-LAIV	Infl Lab Conf	3	12768	0.51 (0.40, 0.66)	50.43%	-0.03 (-0.05, -0.02)	61.23%
placebo vs 3-IIV	Systemic AE	5	5495	1.14 (0.69, 1.87)	**97.38%**	0.04 (-0.06, 0.15)	**93.57%**
placebo vs 3-LAIV	Systemic AE	20	21549	1.09 (1.03, 1.16)	0.00%	0.01 (-0.00, 0.02)	**79.33%**
3-IIV vs 3-IIV HD	Systemic AE	3	171	1.03 (0.32, 3.28)	61.36%	0.00 (-0.24, 0.24)	74.68%
3-IIV vs 3-IIV ID	Systemic AE	3	350	1.22 (0.73, 2.03)	41.44%	0.05 (-0.07, 0.17)	45.71%
3-IIV vs 3-IIV MF59/AS03-adj	Systemic AE	7	11051	1.17 (1.06, 1.30)	23.57%	0.04 (0.01, 0.07)	47.29%
3-IIV vs 3-IIV vir/lip-adj	Systemic AE	3	577	1.21 (0.92, 1.61)	0.00%	0.02 (-0.01, 0.06)	0.00%
3-IIV vs 4-IIV	Systemic AE	13	21378	0.97 (0.87, 1.08)	**84.87%**	-0.01 (-0.04, 0.02)	**81.37%**
3-IIV vs 3-LAIV	Systemic AE	6	13093	1.39 (0.97, 1.98)	**92.25%**	0.04 (0.02, 0.05)	10.57%
3-LAIV vs 4-LAIV	Systemic AE	2	2512	2.97 (0.86, 10.32)	0.00%	0.01 (0.00, 0.01)	0.00%
placebo vs 3-IIV	Local AE	5	5495	1.47 (0.92, 2.35)	**95.29%**	0.09 (-0.02, 0.20)	**90.90%**
placebo vs 3-LAIV	Local AE	19	21293	1.13 (1.08, 1.19)	55.59%	0.06 (0.03, 0.09)	**79.75%**
3-IIV vs 3-IIV HD	Local AE	3	171	1.07 (0.72, 1.59)	0.00%	0.00 (-0.14, 0.15)	3.78%
3-IIV vs 3-IIV ID	Local AE	3	350	1.68 (0.66, 4.26)	22.64%	0.04 (-0.07, 0.16)	**79.64%**
3-IIV vs 3-IIV MF59/AS03-adj	Local AE	7	11051	1.24 (0.93, 1.66)	**94.33%**	0.06 (0.00, 0.12)	**86.48%**
3-IIV vs 3-IIV vir/lip-adj	Local AE	3	577	0.90 (0.61, 1.33)	0.00%	0.00 (-0.03, 0.04)	0.00%
3-IIV vs 4-IIV	Local AE	13	21323	1.02 (0.95, 1.09)	60.97%	0.01 (-0.01, 0.02)	55.23%
3-IIV vs 3-LAIV	Local AE	4	2512	2.12 (1.93, 2.32)	0.00%	0.34 (0.26, 0.42)	41.00%
placebo vs 3-IIV	Otitis Media	3	1137	1.02 (0.78, 1.34)	52.15%	0.00 (-0.08, 0.08)	68.34%
placebo vs 3-LAIV	Otitis Media	7	15737	0.59 (0.30, 1.16)	**83.25%**	-0.02 (-0.05, 0.01)	**92.19%**
placebo vs 3-IIV	ILI	6	2148	0.61 (0.44, 0.84)	63.49%	-0.09 (-0.15, -0.03)	73.80%

Comparison	Outcome	N. studies	N. patients	RR (95% CI)	I^2 RR	ARD (95%CI)	I^2ARD
placebo vs 3-LAIV	ILI	7	12465	0.72 (0.57, 0.90)	70.61%	-0.07 (-0.12, -0.03)	**83.55%**
3-IIV vs 3-LAIV	ILI	3	10907	1.11 (0.98, 1.26)	0.00%	0.01 (-0.00, 0.02)	0.00%
placebo vs 3-IIV	Mortality	3	8663	0.90 (0.11, 7.43)	0.00%	0.00 (-0.00, 0.00)	0.00%
placebo vs 4-IIV	Mortality	2	17454	1.17 (0.10, 13.37)	58.11%	0.00 (-0.00, 0.00)	69.21%
placebo vs 3-LAIV	Mortality	3	4994	1.51 (0.25, 9.21)	0.00%	0.00 (-0.00, 0.00)	0.00%
3-IIV vs 3-IIV MF59/AS03-adj	Mortality	3	10101	0.28 (0.05, 1.49)	0.00%	-0.00 (-0.00, 0.00)	66.59%
3-IIV vs 4-IIV	Mortality	4	7008	1.32 (0.13, 13.50)	0.00%	0.00 (-0.00, 0.00)	0.00%
placebo vs 3-IIV	Hospitalization	5	7372	1.03 (0.59, 1.78)	45.59%	-0.00 (-0.01, 0.01)	64.60%
placebo vs 4-IIV	Hospitalization	2	17454	1.00 (0.23, 4.39)	0.00%	-0.00 (-0.00, 0.00)	0.00%
placebo vs 3-LAIV	Hospitalization	3	12254	1.02 (0.58, 1.80)	17.61%	-0.00 (-0.00, 0.00)	19.06%
3-IIV vs 3-LAIV	Hospitalization	3	12768	1.08 (0.85, 1.36)	0.00%	0.00 (-0.01, 0.01)	57.09%
**Young children (up to 5 years)**							
placebo vs 3-IIV	Infl Lab Conf	6	6646	0.65 (0.51, 0.82)	31.95%	-0.04 (-0.08, -0.01)	62.63%
placebo vs 4-IIV	Infl Lab Conf	2	17454	0.51 (0.46, 0.56)	0.00%	-0.05 (-0.06, -0.04)	26.82%
placebo vs 3-LAIV	Infl Lab Conf	8	15620	0.30 (0.18, 0.51)	**95.74%**	-0.09 (-0.12, -0.05)	**90.87%**
3-IIV vs 3-LAIV	Infl Lab Conf	2	10539	0.45 (0.38, 0.54)	0.00%	-0.04 (-0.05, -0.03)	46.54%
placebo vs 3-IIV	Systemic AE	3	4636	1.37 (0.69, 2.73)	**98.74%**	0.09 (-0.09, 0.26)	**96.91%**
placebo vs 3-LAIV	Systemic AE	16	19436	1.09 (1.03, 1.16)	0.00%	0.01 (-0.00, 0.03)	**83.58%**
3-IIV vs 3-IIV MF59/AS03-adj	Systemic AE	7	11051	1.17 (1.06, 1.30)	23.57%	0.04 (0.01, 0.07)	47.29%
3-IIV vs 4-IIV	Systemic AE	6	8166	0.93 (0.74, 1.16)	**92.18%**	-0.03 (-0.11, 0.05)	**89.48%**
3-IIV vs 3-LAIV	Systemic AE	3	10566	1.87 (0.83, 4.20)	**94.46%**	0.03 (0.03, 0.04)	0.00%

Comparison	Outcome	N. studies	N. patients	RR (95% CI)	I^2 RR	ARD (95%CI)	I^2ARD
placebo vs 3-IIV	Local AE	3	4636	1.30 (0.76, 2.23)	**95.60%**	0.05 (-0.07, 0.17)	**90.43%**
placebo vs 3-LAIV	Local AE	15	19180	1.13 (1.07, 1.19)	62.51%	0.05 (0.02, 0.09)	**82.22%**
3-IIV vs 3-IIV MF59/AS03-adj	Local AE	7	11051	1.24 (0.93, 1.66)	**94.33%**	0.06 (0.00, 0.12)	**86.48%**
3-IIV vs 4-IIV	Local AE	6	8166	0.96 (0.81, 1.14)	74.47%	-0.01 (-0.06, 0.04)	**81.96%**
3-IIV vs 3-LAIV	Local AE	2	2214	3.30 (0.83, 13.05)	59.08%	0.45 (0.19, 0.71)	71.52%
placebo vs 3-IIV	Otitis Media	3	1137	1.02 (0.78, 1.34)	52.15%	0.00 (-0.08, 0.08)	68.34%
placebo vs 3-LAIV	Otitis Media	5	5999	0.33 (0.13, 0.81)	**87.06%**	-0.03 (-0.04, -0.02)	0.00%
placebo vs 3-IIV	ILI	3	878	0.58 (0.33, 1.01)	**79.09%**	-0.14 (-0.30, 0.02)	**87.12%**
3-IIV vs 3-LAIV	ILI	2	10539	1.11 (0.97, 1.26)	0.00%	0.01 (-0.00, 0.02)	0.00%
placebo vs 3-IIV	Mortality	3	8663	0.90 (0.11, 7.43)	0.00%	0.00 (-0.00, 0.00)	0.00%
placebo vs 4-IIV	Mortality	2	17454	1.17 (0.10, 13.37)	58.11%	0.00 (-0.00, 0.00)	69.21%
placebo vs 3-LAIV	Mortality	2	2994	1.09 (0.12, 9.79)	0.00%	0.00 (-0.00, 0.00)	0.00%
3-IIV vs 3-IIV MF59/AS03-adj	Mortality	3	10101	0.28 (0.05, 1.49)	0.00%	-0.00 (-0.00, 0.00)	66.59%
placebo vs 3-IIV	Hospitalization	5	7372	1.03 (0.59, 1.78)	45.59%	-0.00 (-0.01, 0.01)	64.60%
placebo vs 4-IIV	Hospitalization	2	17454	1.00 (0.23, 4.39)	0.00%	-0.00 (-0.00, 0.00)	0.00%
placebo vs 3-LAIV	Hospitalization	2	2565	1.51 (0.67, 3.41)	0.00%	0.00 (-0.01, 0.01)	50.70%
3-IIV vs 3-LAIV	Hospitalization	2	10539	1.09 (0.86, 1.38)	0.00%	0.00 (-0.00, 0.01)	0.00%
**All children with chronic respiratory diseases**							
placebo vs 3-LAIV	Infl Lab Conf	2	84	0.17 (0.05, 0.58)	0.00%	-0.32 (-0.49, -0.15)	17.33%
3-IIV vs 3-LAIV	Infl Lab Conf	2	4416	0.58 (0.40, 0.82)	40.53%	-0.03 (-0.04, -0.01)	0.00%
placebo vs 3-LAIV	ILI	2	84	0.23 (0.08, 0.64)	0.00%	-0.35 (-0.51, -0.19)	0.00%

Comparison	Outcome	N. studies	N. patients	RR (95% CI)	I^2 RR	ARD (95%CI)	I^2ARD
3-IIV vs 3-LAIV	Hospitalization	2	4416	0.89 (0.31, 2.54)	10.39%	-0.00 (-0.00, 0.00)	0.00%
placebo vs 3-LAIV	Exacerb Chrnon Disease	2	93	2.76 (0.26, 29.54)	0.00%	0.03 (-0.06, 0.12)	27.84%
**All children immunocompromised/cancer**							
3-IIV vs 3-IIV HD	Systemic AE	3	171	1.03 (0.32, 3.28)	61.36%	0.00 (-0.24, 0.24)	74.68%
3-IIV vs 3-LAIV	Systemic AE	2	298	1.07 (0.73, 1.56)	0.00%	0.02 (-0.08, 0.12)	0.00%
3-IIV vs 3-IIV HD	Local AE	3	171	1.07 (0.72, 1.59)	0.00%	0.00 (-0.14, 0.15)	3.78%
3-IIV vs 3-LAIV	Local AE	2	298	2.27 (1.62, 3.19)	0.00%	0.28 (0.18, 0.39)	0.00%

I^2 values exceeding 75 % are highlighted

Abbreviations

Placebo: placebo/no vaccine; **3-IIV:** trivalent inactivated intramuscular; **3-IIV HD:** trivalent inactivated high dose intramuscular; **3-IIV ID:** trivalent inactivated intradermal; **3-IIV MF59/AS03-adj:** trivalent inactivated adjuvanted with MF59/ASO3 intramuscular: **3-IIV vir/lip-adj:** trivalent inactivated adjuvanted with Virosome/liposome intramuscular: **3-RIV:** trivalent recombinant intramuscular; **4-IIV:** quadrivalent inactivated intramuscular; **4-IIV HD:** quadrivalent inactivated high dose intramuscular: **4-IIV ID:** quadrivalent inactivated intradermal; **4-IIV MF59/AS03-adj:** quadrivalent inactivated adjuvanted with MF59/ASO3 intramuscular; **4-IIV vir/lip-adj:** quadrivalent inactivated adjuvanted with virosome/liposome intramuscular; **4-RIV:** quadrivalent recombinant intramuscular; **3-LAIV:** trivalent live attenuated intranasal; **4-LAIV:** quadrivalent live attenuated intranasal.

Adults and the elderly: Inconsistency in networks of laboratory- confirmed influenza and any systemic adverse event outcomes

Laboratory- confirmed influenza[*]

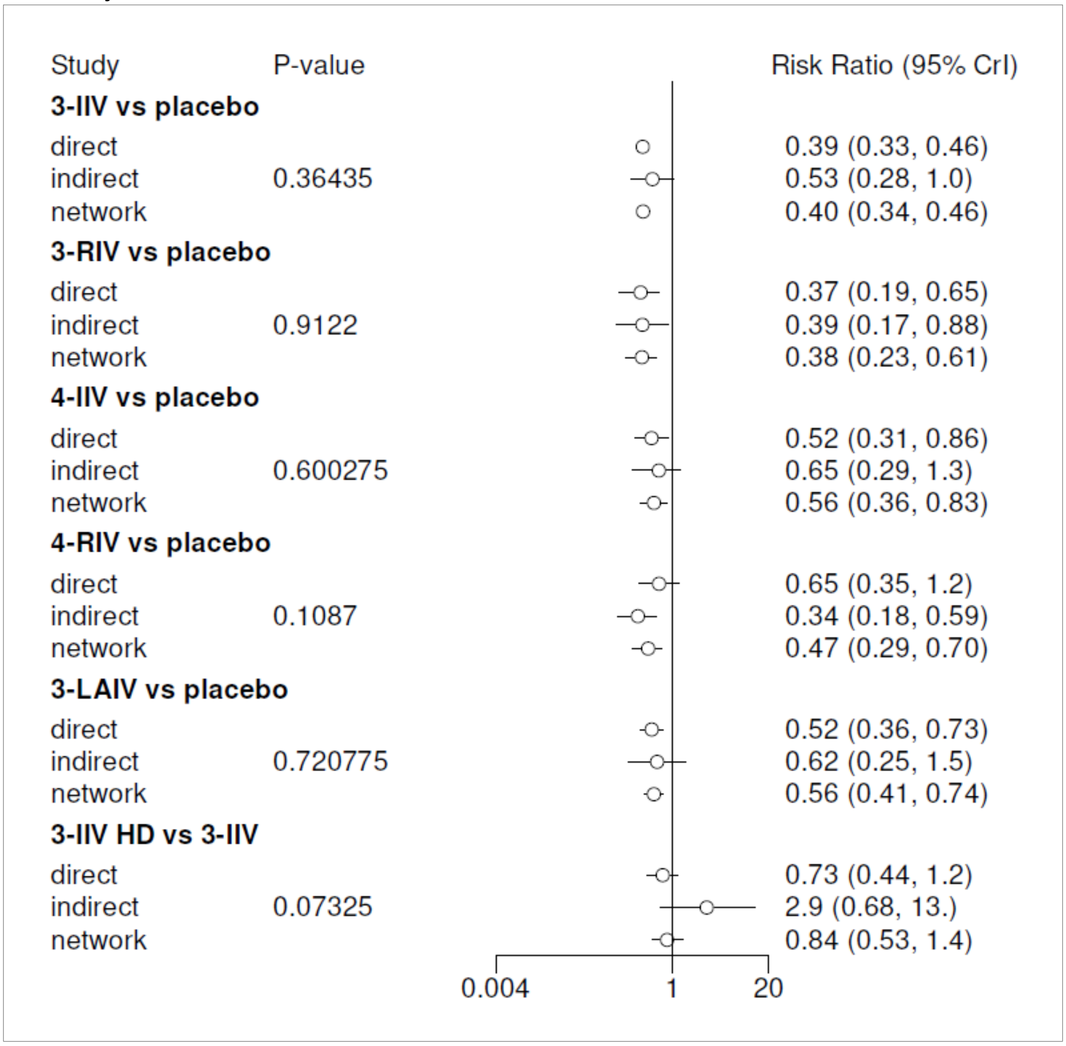

OR <1 favors experimental vaccine compared to placebo or 3-IIV

[*] **Placebo:** placebo/no vaccine; **3-IIV:** trivalent inactivated intramuscular; **3-IIV HD:** trivalent inactivated high dose intramuscular; **3-IIV ID:** trivalent inactivated intradermal; **3-IIV MF59/AS03-adj:** trivalent inactivated adjuvanted with MF59/ASO3 intramuscular: **3-IIV vir/lip-adj:** trivalent inactivated adjuvanted with Virosome/liposome intramuscular: **3-RIV:** trivalent recombinant intramuscular; **4-IIV:** quadrivalent inactivated intramuscular; **4-IIV HD:** quadrivalent inactivated high dose intramuscular: **4-IIV ID:** quadrivalent inactivated intradermal; **4-IIV MF59/AS03-adj:** quadrivalent inactivated adjuvanted with MF59/ASO3 intramuscular; **4-IIV vir/lip-adj:** quadrivalent inactivated adjuvanted with virosome/liposome intramuscular; **4-RIV:** quadrivalent recombinant intramuscular; **3-LAIV:** trivalent live attenuated intranasal; **4-LAIV:** quadrivalent live attenuated intranasal.

Laboratory- confirmed influenza*

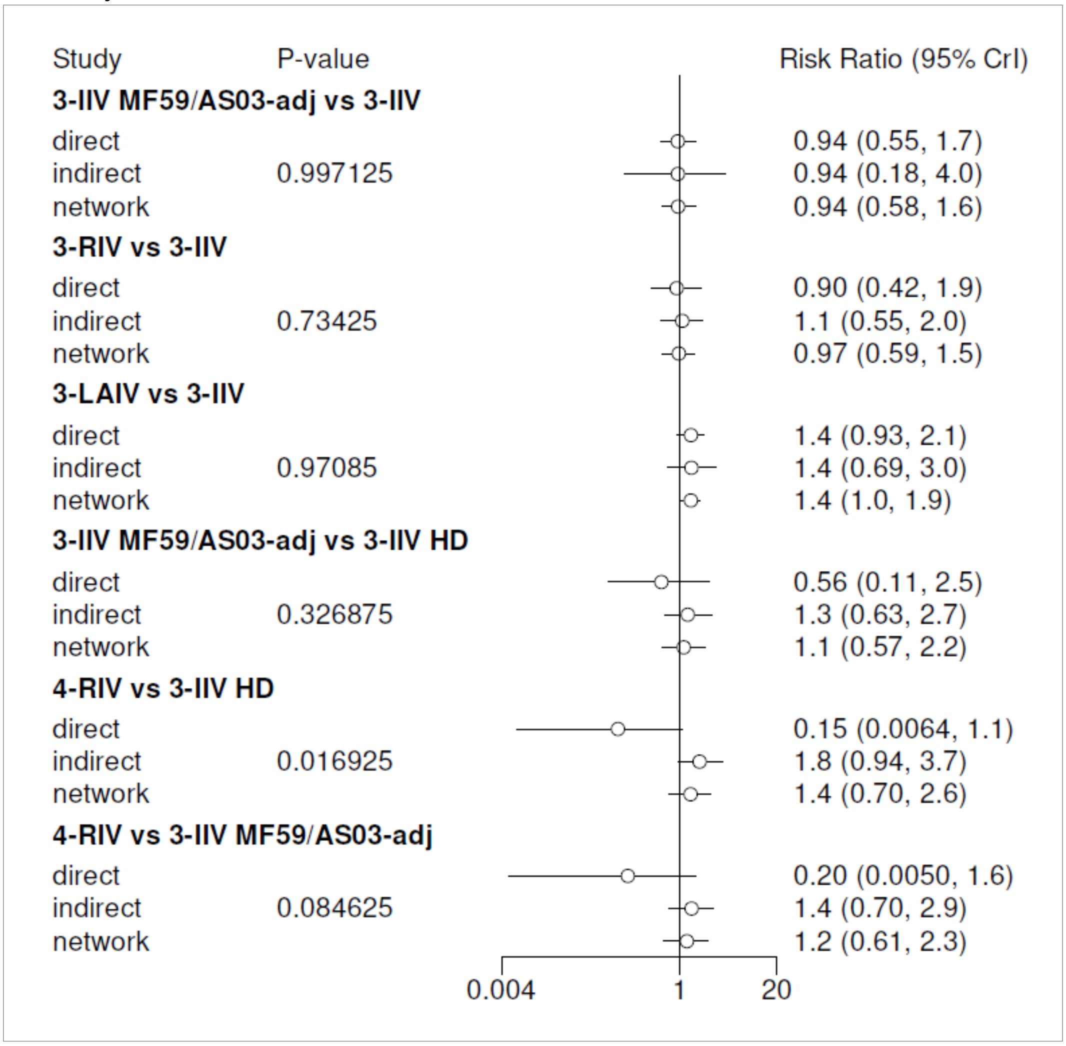

OR <1 favors experimental vaccine compared to placebo or 3-IIV

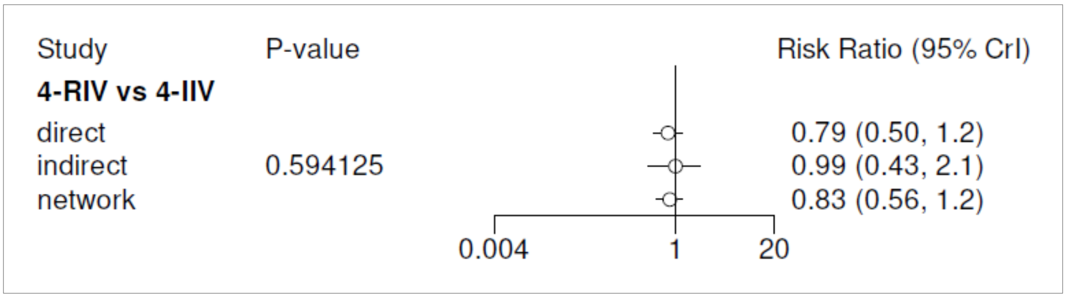

OR <1 favors experimental vaccine compared to placebo or 3-IIV

* **Placebo:** placebo/no vaccine; **3-IIV:** trivalent inactivated intramuscular; **3-IIV HD:** trivalent inactivated high dose intramuscular; **3-IIV ID:** trivalent inactivated intradermal; **3-IIV MF59/AS03-adj:** trivalent inactivated adjuvanted with MF59/ASO3 intramuscular: **3-IIV vir/lip-adj:** trivalent inactivated adjuvanted with Virosome/liposome intramuscular: **3-RIV:** trivalent recombinant intramuscular; **4-IIV:** quadrivalent inactivated intramuscular; **4-IIV HD:** quadrivalent inactivated high dose intramuscular: **4-IIV ID:** quadrivalent inactivated intradermal; **4-IIV MF59/AS03-adj:** quadrivalent inactivated adjuvanted with MF59/ASO3 intramuscular; **4-IIV vir/lip-adj:** quadrivalent inactivated adjuvanted with virosome/liposome intramuscular; **4-RIV:** quadrivalent recombinant intramuscular; **3-LAIV:** trivalent live attenuated intranasal; **4-LAIV:** quadrivalent live attenuated intranasal.

Any systemic adverse event[*]

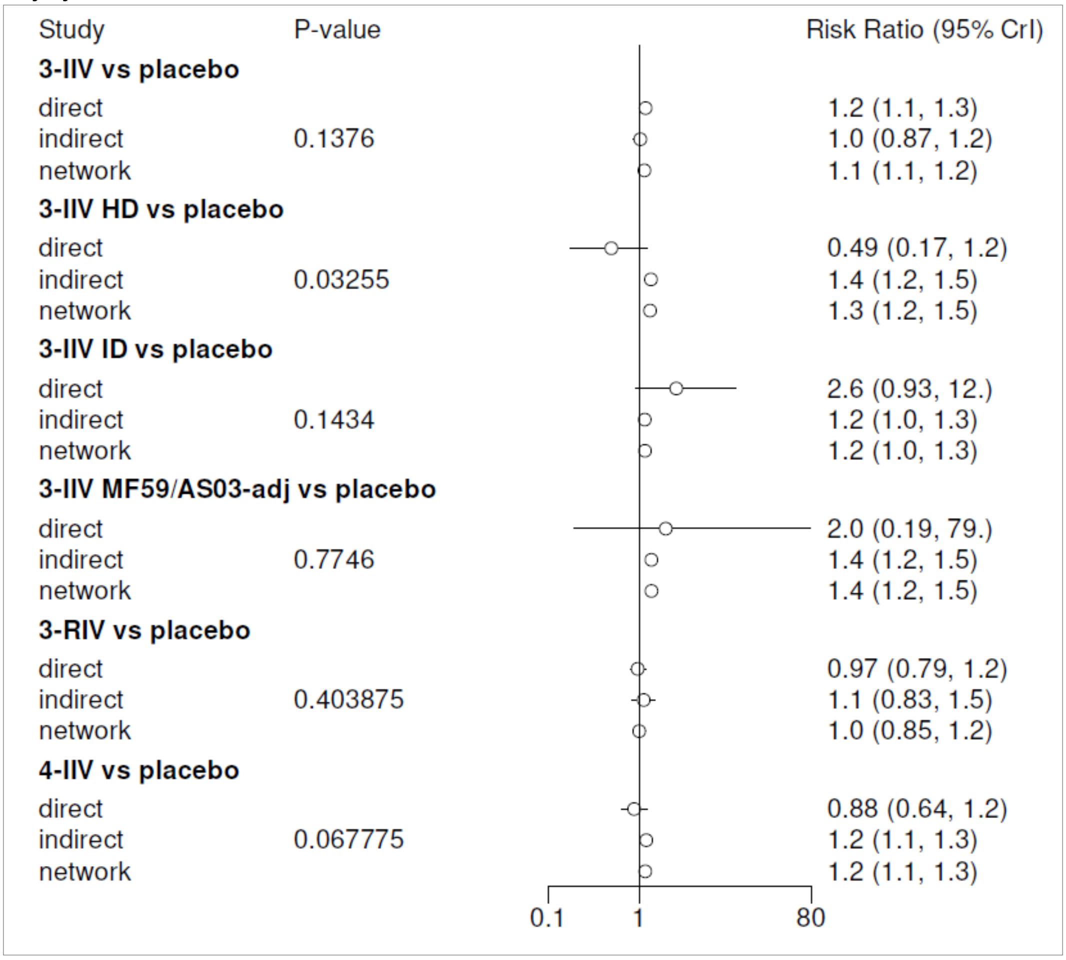

OR <1 favors experimental vaccine compared to placebo or 3-IIV

[*] **Placebo:** placebo/no vaccine; **3-IIV:** trivalent inactivated intramuscular; **3-IIV HD:** trivalent inactivated high dose intramuscular; **3-IIV ID:** trivalent inactivated intradermal; **3-IIV MF59/AS03-adj:** trivalent inactivated adjuvanted with MF59/ASO3 intramuscular: **3-IIV vir/lip-adj:** trivalent inactivated adjuvanted with Virosome/liposome intramuscular: **3-RIV:** trivalent recombinant intramuscular; **4-IIV:** quadrivalent inactivated intramuscular; **4-IIV HD:** quadrivalent inactivated high dose intramuscular: **4-IIV ID:** quadrivalent inactivated intradermal; **4-IIV MF59/AS03-adj:** quadrivalent inactivated adjuvanted with MF59/ASO3 intramuscular; **4-IIV vir/lip-adj:** quadrivalent inactivated adjuvanted with virosome/liposome intramuscular; **4-RIV:** quadrivalent recombinant intramuscular; **3-LAIV:** trivalent live attenuated intranasal; **4-LAIV:** quadrivalent live attenuated intranasal.

Any systemic adverse event[*]

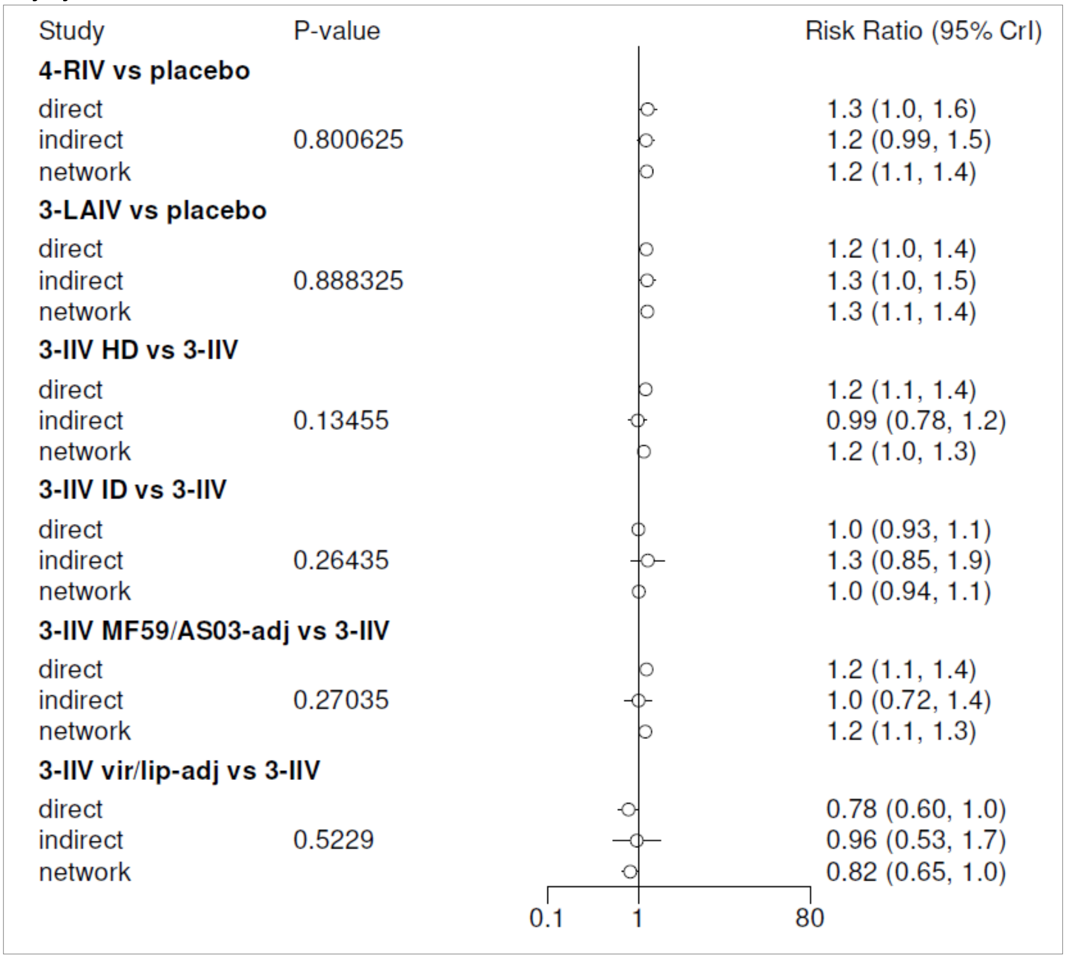

OR <1 favors experimental vaccine compared to placebo or 3-IIV

[*] **Placebo:** placebo/no vaccine; **3-IIV:** trivalent inactivated intramuscular; **3-IIV HD:** trivalent inactivated high dose intramuscular; **3-IIV ID:** trivalent inactivated intradermal; **3-IIV MF59/AS03-adj:** trivalent inactivated adjuvanted with MF59/ASO3 intramuscular: **3-IIV vir/lip-adj:** trivalent inactivated adjuvanted with Virosome/liposome intramuscular: **3-RIV:** trivalent recombinant intramuscular; **4-IIV:** quadrivalent inactivated intramuscular; **4-IIV HD:** quadrivalent inactivated high dose intramuscular: **4-IIV ID:** quadrivalent inactivated intradermal; **4-IIV MF59/AS03-adj:** quadrivalent inactivated adjuvanted with MF59/ASO3 intramuscular; **4-IIV vir/lip-adj:** quadrivalent inactivated adjuvanted with virosome/liposome intramuscular; **4-RIV:** quadrivalent recombinant intramuscular; **3-LAIV:** trivalent live attenuated intranasal; **4-LAIV:** quadrivalent live attenuated intranasal.

Any systemic adverse event[*]

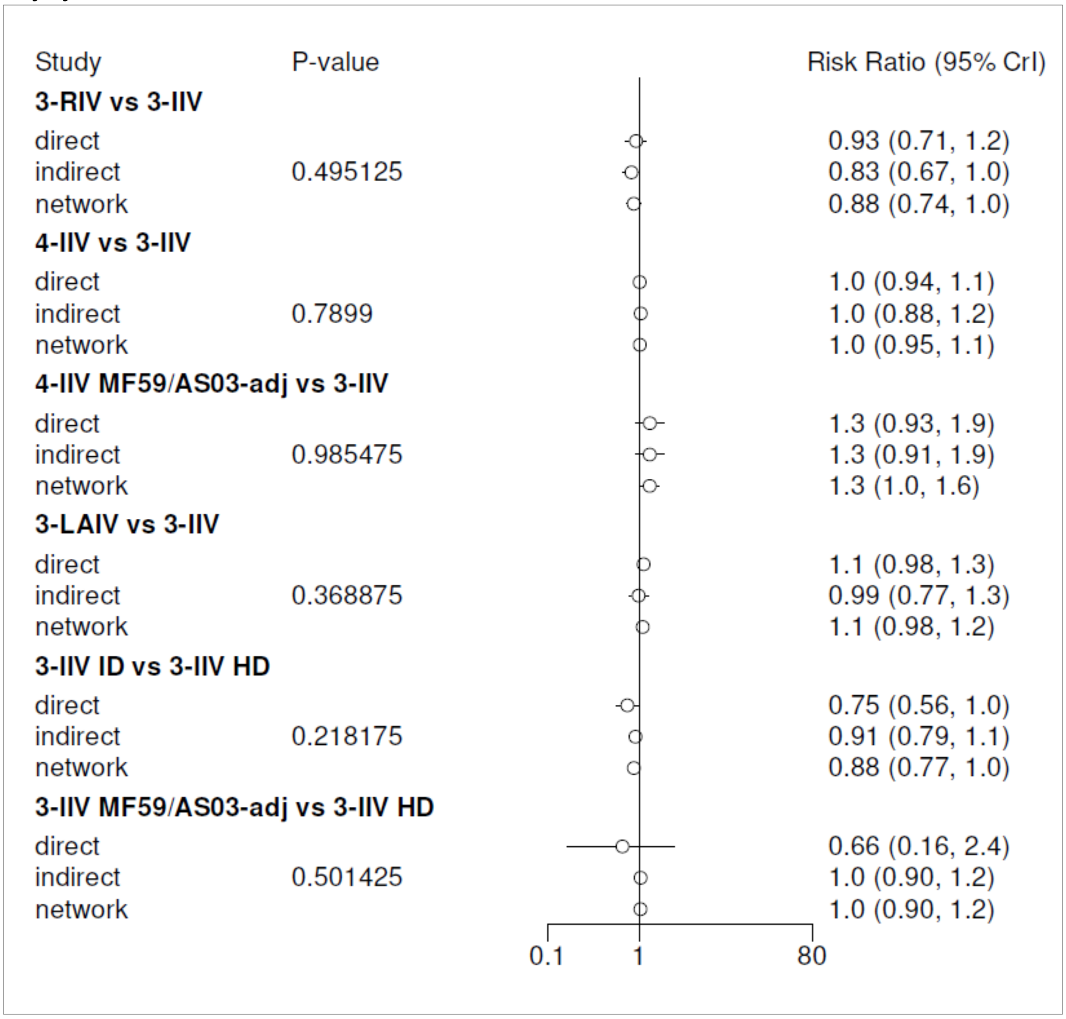

OR <1 favors experimental vaccine compared to placebo or 3-IIV

[*] **Placebo:** placebo/no vaccine; **3-IIV:** trivalent inactivated intramuscular; **3-IIV HD:** trivalent inactivated high dose intramuscular; **3-IIV ID:** trivalent inactivated intradermal; **3-IIV MF59/AS03-adj:** trivalent inactivated adjuvanted with MF59/ASO3 intramuscular: **3-IIV vir/lip-adj:** trivalent inactivated adjuvanted with Virosome/liposome intramuscular: **3-RIV:** trivalent recombinant intramuscular; **4-IIV:** quadrivalent inactivated intramuscular; **4-IIV HD:** quadrivalent inactivated high dose intramuscular: **4-IIV ID:** quadrivalent inactivated intradermal; **4-IIV MF59/AS03-adj:** quadrivalent inactivated adjuvanted with MF59/ASO3 intramuscular; **4-IIV vir/lip-adj:** quadrivalent inactivated adjuvanted with virosome/liposome intramuscular; **4-RIV:** quadrivalent recombinant intramuscular; **3-LAIV:** trivalent live attenuated intranasal; **4-LAIV:** quadrivalent live attenuated intranasal.

Any systemic adverse event*

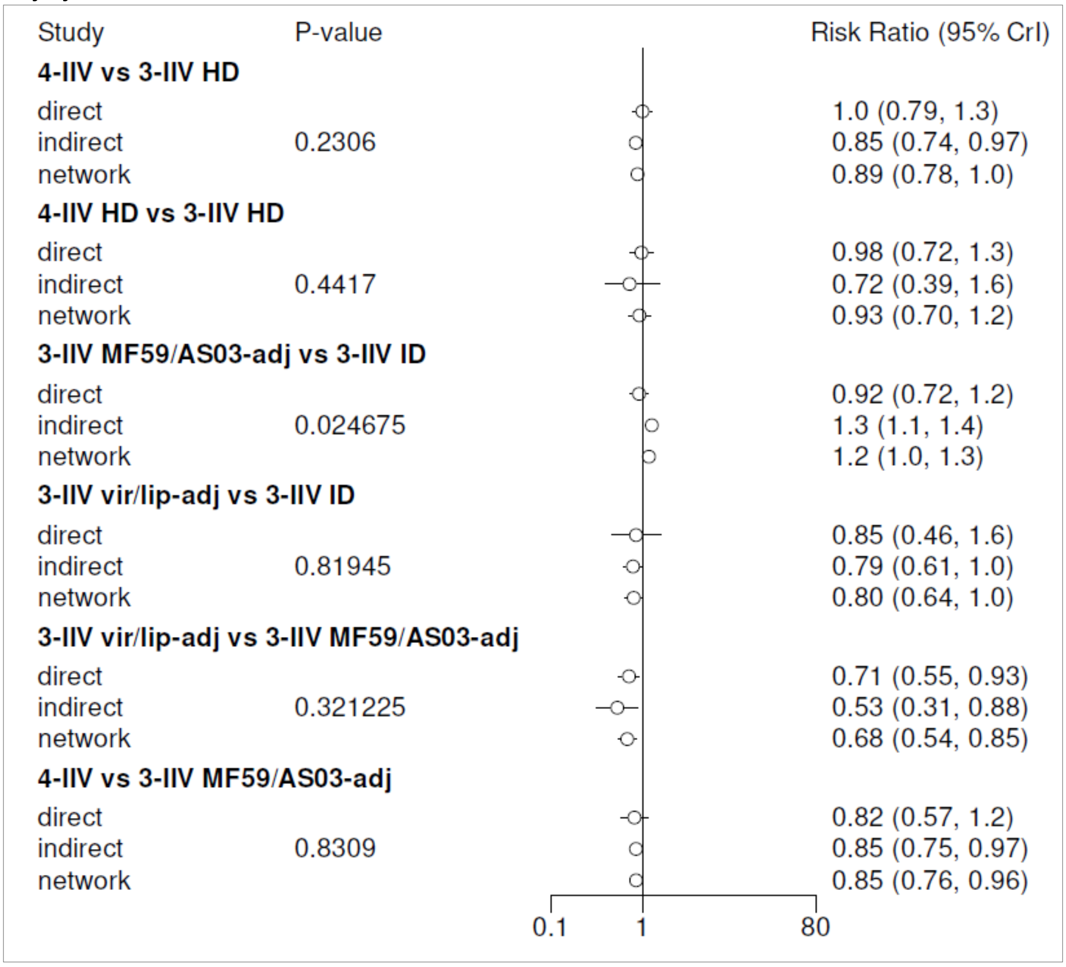

OR <1 favors experimental vaccine compared to placebo or 3-IIV

* **Placebo:** placebo/no vaccine; **3-IIV:** trivalent inactivated intramuscular; **3-IIV HD:** trivalent inactivated high dose intramuscular; **3-IIV ID:** trivalent inactivated intradermal; **3-IIV MF59/AS03-adj:** trivalent inactivated adjuvanted with MF59/ASO3 intramuscular: **3-IIV vir/lip-adj:** trivalent inactivated adjuvanted with Virosome/liposome intramuscular: **3-RIV:** trivalent recombinant intramuscular; **4-IIV:** quadrivalent inactivated intramuscular; **4-IIV HD:** quadrivalent inactivated high dose intramuscular: **4-IIV ID:** quadrivalent inactivated intradermal; **4-IIV MF59/AS03-adj:** quadrivalent inactivated adjuvanted with MF59/ASO3 intramuscular; **4-IIV vir/lip-adj:** quadrivalent inactivated adjuvanted with virosome/liposome intramuscular; **4-RIV:** quadrivalent recombinant intramuscular; **3-LAIV:** trivalent live attenuated intranasal; **4-LAIV:** quadrivalent live attenuated intranasal.

Any systemic adverse event[*]

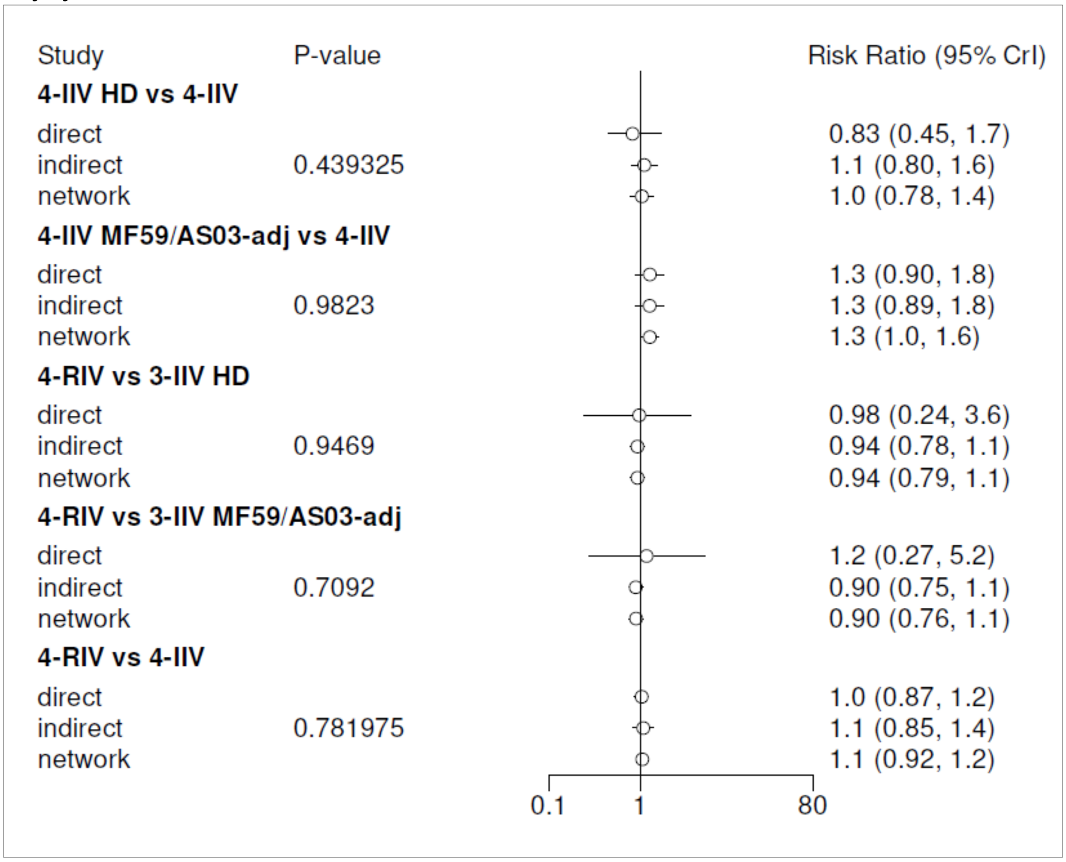

OR <1 favors experimental vaccine compared to placebo or 3-IIV

[*] **Placebo:** placebo/no vaccine; **3-IIV:** trivalent inactivated intramuscular; **3-IIV HD:** trivalent inactivated high dose intramuscular; **3-IIV ID:** trivalent inactivated intradermal; **3-IIV MF59/AS03-adj:** trivalent inactivated adjuvanted with MF59/ASO3 intramuscular: **3-IIV vir/lip-adj:** trivalent inactivated adjuvanted with Virosome/liposome intramuscular: **3-RIV:** trivalent recombinant intramuscular; **4-IIV:** quadrivalent inactivated intramuscular; **4-IIV HD:** quadrivalent inactivated high dose intramuscular: **4-IIV ID:** quadrivalent inactivated intradermal; **4-IIV MF59/AS03-adj:** quadrivalent inactivated adjuvanted with MF59/ASO3 intramuscular; **4-IIV vir/lip-adj:** quadrivalent inactivated adjuvanted with virosome/liposome intramuscular; **4-RIV:** quadrivalent recombinant intramuscular; **3-LAIV:** trivalent live attenuated intranasal; **4-LAIV:** quadrivalent live attenuated intranasal.

All children: Inconsistency in networks of laboratory- confirmed influenza and any systemic adverse event outcomes[*]

Laboratory- confirmed influenza

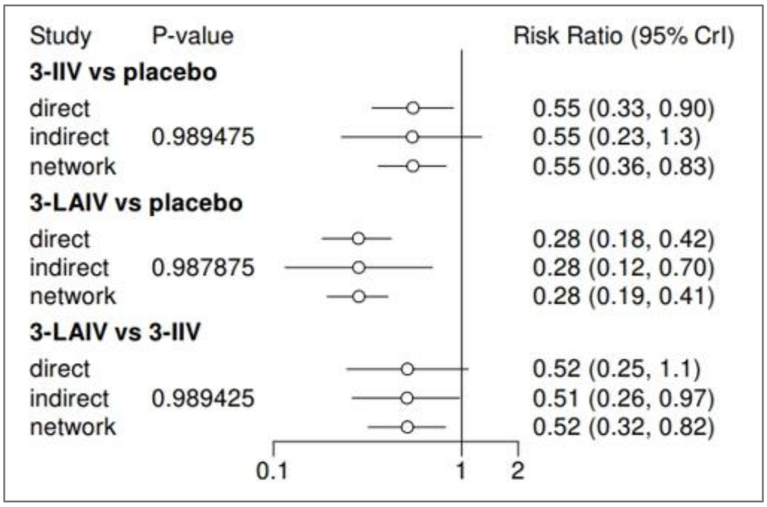

OR <1 favors experimental vaccine compared to placebo or 3-IIV

Any systemic adverse event

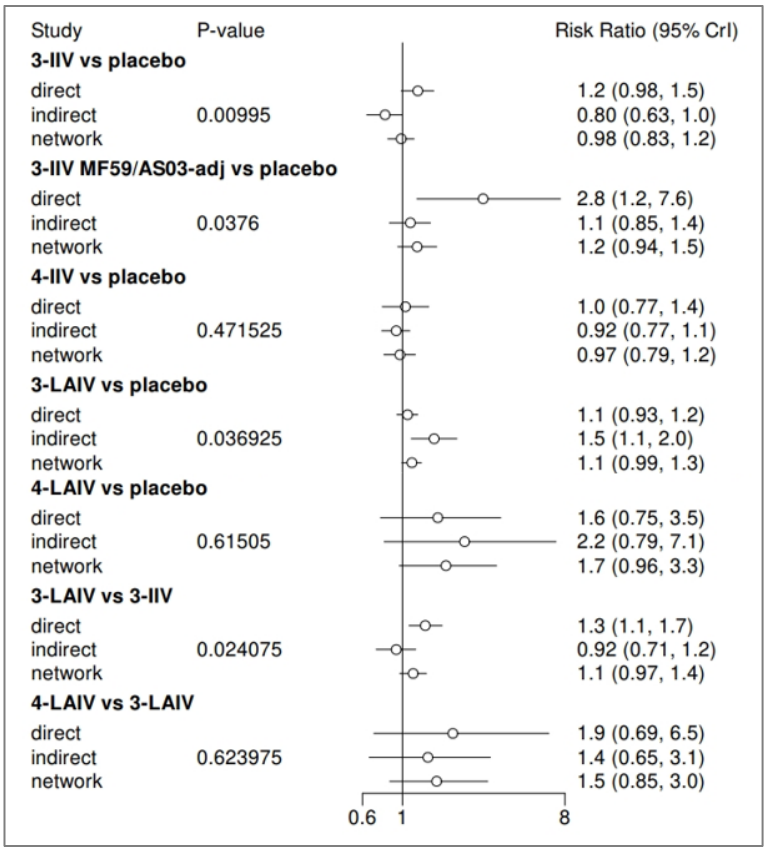

OR <1 favors experimental vaccine compared to placebo or 3-IIV

[*] **Placebo:** placebo/no vaccine; **3-IIV:** trivalent inactivated intramuscular; **3-IIV HD:** trivalent inactivated high dose intramuscular; **3-IIV ID:** trivalent inactivated intradermal; **3-IIV MF59/AS03-adj:** trivalent inactivated adjuvanted with MF59/ASO3 intramuscular: **3-IIV vir/lip-adj:** trivalent inactivated adjuvanted with Virosome/liposome intramuscular: **3-RIV:** trivalent recombinant intramuscular; **4-IIV:** quadrivalent inactivated intramuscular; **4-IIV HD:** quadrivalent inactivated high dose intramuscular: **4-IIV ID:** quadrivalent inactivated intradermal; **4-IIV MF59/AS03-adj:** quadrivalent inactivated adjuvanted with MF59/ASO3 intramuscular; **4-IIV vir/lip-adj:** quadrivalent inactivated adjuvanted with virosome/liposome intramuscular; **4-RIV:** quadrivalent recombinant intramuscular; **3-LAIV:** trivalent live attenuated intranasal; **4-LAIV:** quadrivalent live attenuated intranasal.

Adults and the elderly: Forest plots of laboratory- confirmed influenza, comparison with placebo/no vaccine and with 3-IIV*

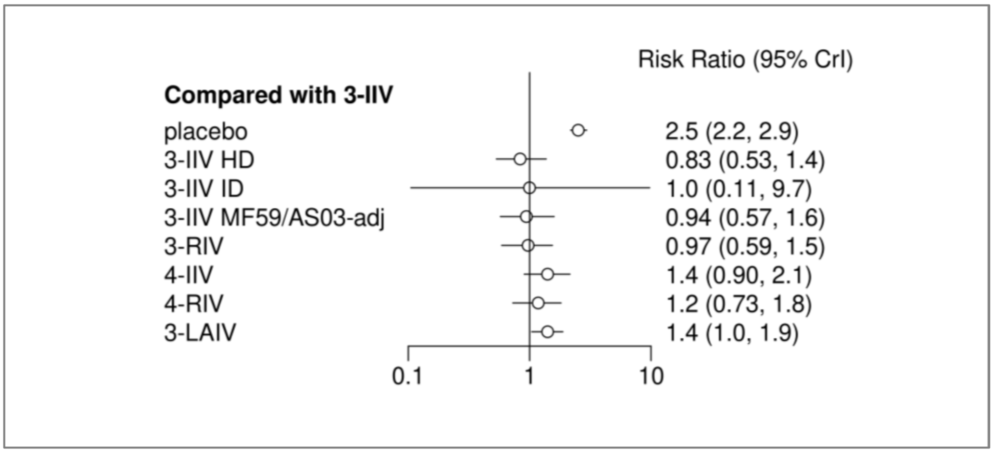

OR <1 favors experimental vaccine compared to placebo or 3-IIV

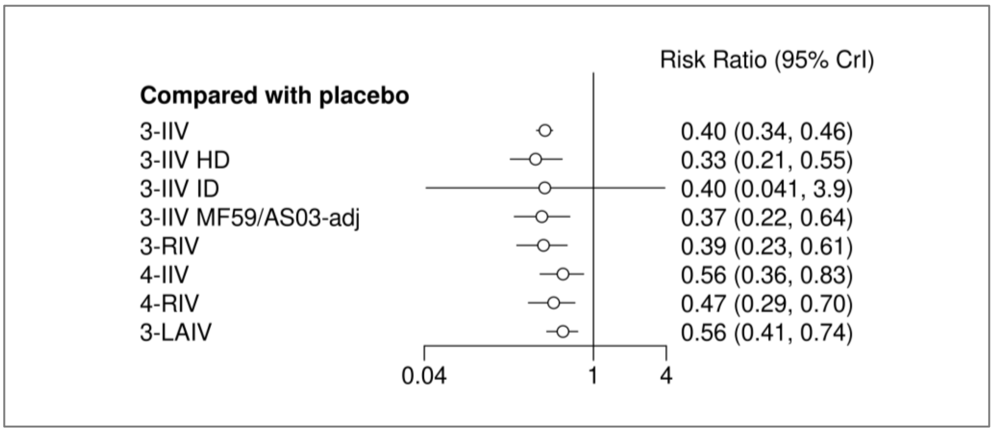

OR <1 favors experimental vaccine compared to placebo or 3-IIV

* **Placebo:** placebo/no vaccine; **3-IIV:** trivalent inactivated intramuscular; **3-IIV HD:** trivalent inactivated high dose intramuscular; **3-IIV ID:** trivalent inactivated intradermal; **3-IIV MF59/AS03-adj:** trivalent inactivated adjuvanted with MF59/ASO3 intramuscular: **3-IIV vir/lip-adj:** trivalent inactivated adjuvanted with Virosome/liposome intramuscular: **3-RIV:** trivalent recombinant intramuscular; **4-IIV:** quadrivalent inactivated intramuscular; **4-IIV HD:** quadrivalent inactivated high dose intramuscular: **4-IIV ID:** quadrivalent inactivated intradermal; **4-IIV MF59/AS03-adj:** quadrivalent inactivated adjuvanted with MF59/ASO3 intramuscular; **4-IIV vir/lip-adj:** quadrivalent inactivated adjuvanted with virosome/liposome intramuscular; **4-RIV:** quadrivalent recombinant intramuscular; **3-LAIV:** trivalent live attenuated intranasal; **4-LAIV:** quadrivalent live attenuated intranasal.

The elderly (≥61 years): Network geometry of laboratory -confirmed influenza*

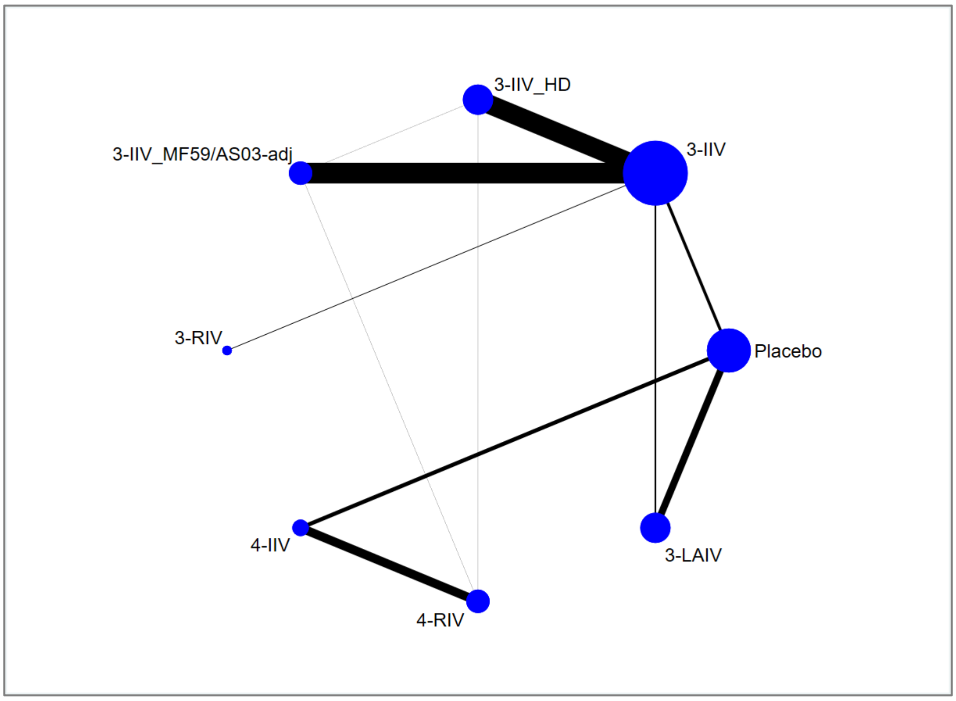

The thickness of the line is proportional to the precision of each direct estimate, and the width of each circle is proportional to the number of studies included in the treatment.

The elderly (≥61 years): Forest plots lof aboratory- confirmed influenza, comparison with placebo-no vaccine and with 3-IIV*

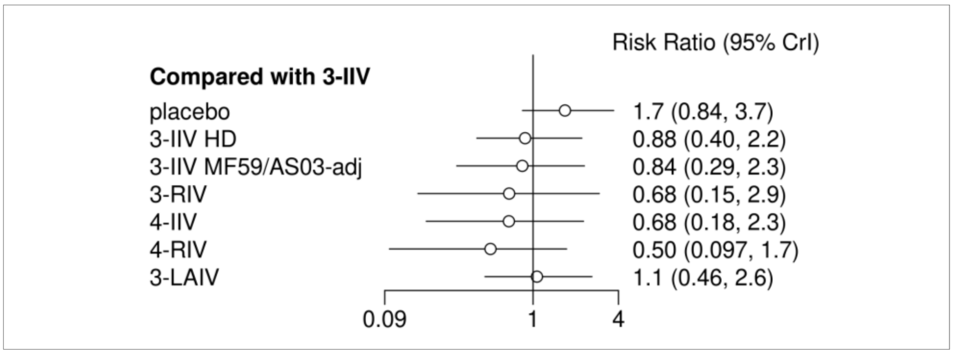

OR <1 favors experimental vaccine compared to placebo or 3-IIV

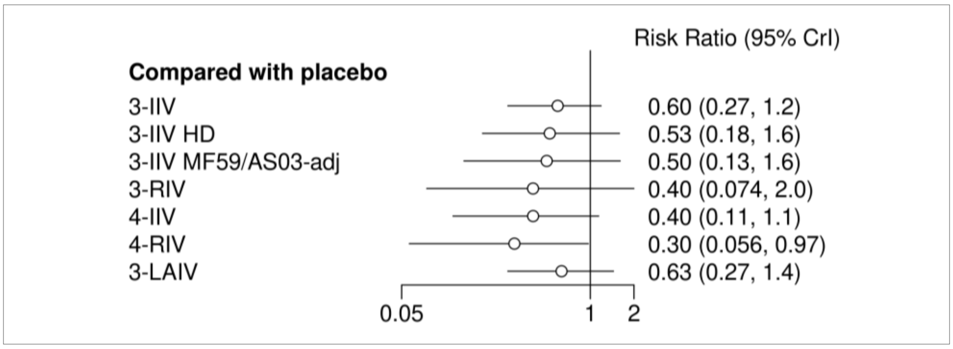

OR <1 favors experimental vaccine compared to placebo or 3-IIV

* **Placebo:** placebo/no vaccine; **3-IIV:** trivalent inactivated intramuscular; **3-IIV HD:** trivalent inactivated high dose intramuscular; **3-IIV ID:** trivalent inactivated intradermal; **3-IIV MF59/AS03-adj:** trivalent inactivated adjuvanted with MF59/ASO3 intramuscular: **3-IIV vir/lip-adj:** trivalent inactivated adjuvanted with Virosome/liposome intramuscular: **3-RIV:** trivalent recombinant intramuscular; **4-IIV:** quadrivalent inactivated intramuscular; **4-IIV HD:** quadrivalent inactivated high dose intramuscular: **4-IIV ID:** quadrivalent inactivated intradermal; **4-IIV MF59/AS03-adj:** quadrivalent inactivated adjuvanted with MF59/ASO3 intramuscular; **4-IIV vir/lip-adj:** quadrivalent inactivated adjuvanted with virosome/liposome intramuscular; **4-RIV:** quadrivalent recombinant intramuscular; **3-LAIV:** trivalent live attenuated intranasal; **4-LAIV:** quadrivalent live attenuated intranasal.

The elderly (≥61 years): Netleague analysis of laboratory- confirmed influenza*

**placebo**	0.6 (0.27, 1.19)	0.53 (0.18, 1.59)	0.5 (0.13, 1.61)	0.4 (0.07, 2)	0.4 (0.11, 1.14)	**0.3 (0.06, 0.97)**	0.63 (0.27, 1.44)
1.68 (0.84, 3.7)	**3-IIV**	0.88 (0.4, 2.21)	0.84 (0.29, 2.3)	0.68 (0.15, 2.93)	0.68 (0.18, 2.27)	0.5 (0.1, 1.72)	1.06 (0.46, 2.6)
1.9 (0.63, 5.54)	1.14 (0.45, 2.49)	**3-IIV HD**	0.95 (0.27, 2.79)	0.76 (0.13, 3.95)	0.76 (0.16, 2.83)	0.56 (0.1, 2.03)	1.21 (0.35, 3.84)
2 (0.62, 7.44)	1.19 (0.43, 3.45)	1.05 (0.36, 3.72)	**3-IIV MF59/AS03-adj**	0.81 (0.14, 4.99)	0.81 (0.17, 3.5)	0.6 (0.1, 2.45)	1.27 (0.36, 5.06)
2.48 (0.5, 13.43)	1.47 (0.34, 6.48)	1.31 (0.25, 7.67)	1.23 (0.2, 7.36)	**3-RIV**	0.99 (0.13, 6.62)	0.73 (0.08, 4.88)	1.57 (0.29, 8.96)
2.48 (0.88, 8.85)	1.48 (0.44, 5.66)	1.31 (0.35, 6.31)	1.24 (0.29, 5.96)	1.01 (0.15, 7.45)	**4-IIV**	0.75 (0.21, 2.02)	1.57 (0.43, 6.95)
**3.34 (1.03, 17.71)**	2 (0.58, 10.29)	1.77 (0.49, 10.42)	1.67 (0.41, 9.63)	1.37 (0.21, 12.53)	1.33 (0.49, 4.79)	**4-RIV**	2.12 (0.55, 12.69)
1.58 (0.69, 3.71)	0.94 (0.38, 2.18)	0.83 (0.26, 2.86)	0.79 (0.2, 2.8)	0.64 (0.11, 3.4)	0.64 (0.14, 2.3)	0.47 (0.08, 1.81)	**3-LAIV**

The estimate is located at the intersection of the column-defining vaccine and the row-defining vaccine. Data are RRs (95% CrI). Significant results are in bold. In the upper triangle, comparisons of treatments should be read from right to left. An RR below 1 favors the medication on the bottom right vs. the medication on the top left in the diagonal.

* **Placebo:** placebo/no vaccine; **3-IIV:** trivalent inactivated intramuscular; **3-IIV HD:** trivalent inactivated high dose intramuscular; **3-IIV ID:** trivalent inactivated intradermal; **3-IIV MF59/AS03-adj:** trivalent inactivated adjuvanted with MF59/ASO3 intramuscular: **3-IIV vir/lip-adj:** trivalent inactivated adjuvanted with Virosome/liposome intramuscular: **3-RIV:** trivalent recombinant intramuscular; **4-IIV:** quadrivalent inactivated intramuscular; **4-IIV HD:** quadrivalent inactivated high dose intramuscular: **4-IIV ID:** quadrivalent inactivated intradermal; **4-IIV MF59/AS03-adj:** quadrivalent inactivated adjuvanted with MF59/ASO3 intramuscular; **4-IIV vir/lip-adj:** quadrivalent inactivated adjuvanted with virosome/liposome intramuscular; **4-RIV:** quadrivalent recombinant intramuscular; **3-LAIV:** trivalent live attenuated intranasal; **4-LAIV:** quadrivalent live attenuated intranasal.

Immunocompromised/cancer patients: Network geometry of laboratory -confirmed influenza[*]

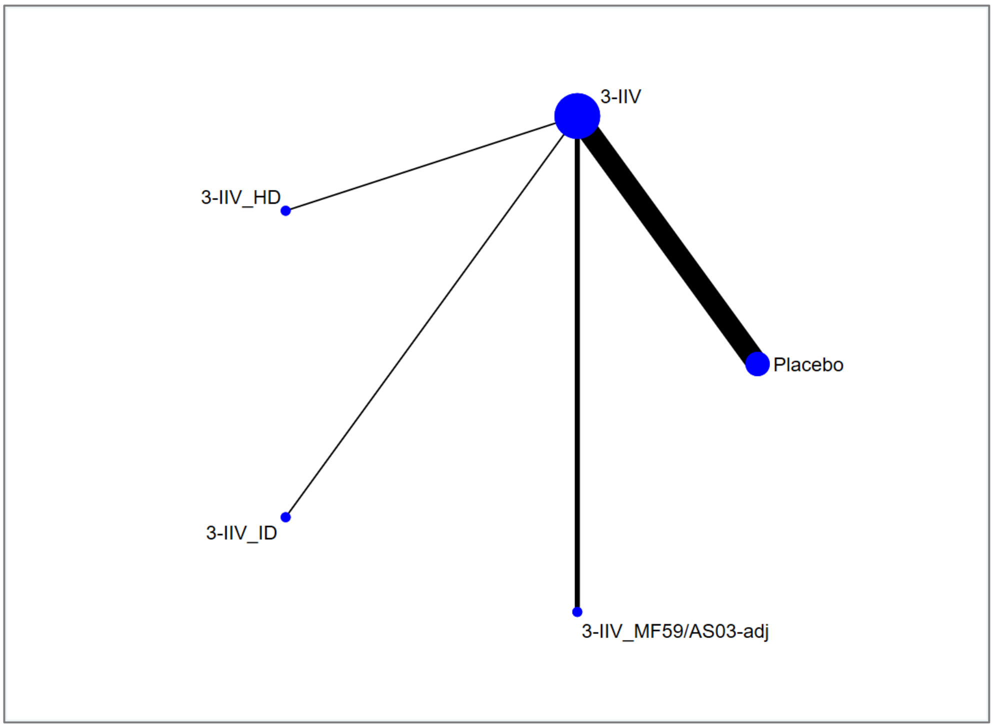

The thickness of the line is proportional to the precision of each direct estimate, and the width of each circle is proportional to the number of studies included in the treatment.

Immunocompromised/cancer patients: Forest plots lof aboratory- confirmed influenza, comparison with placebo-no vaccine and with 3-IIV[*]

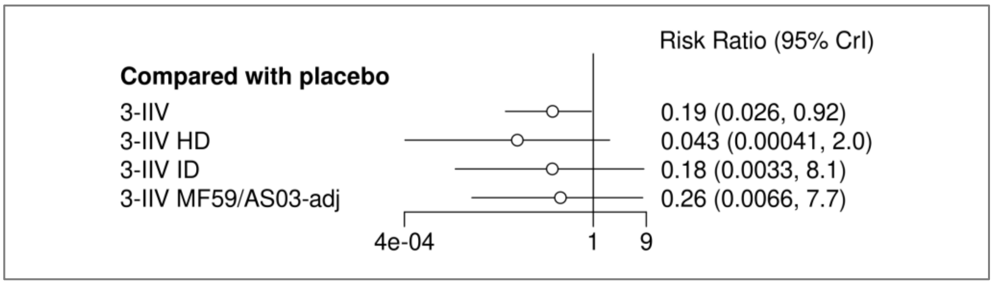

OR <1 favors experimental vaccine compared to placebo or 3-IIV

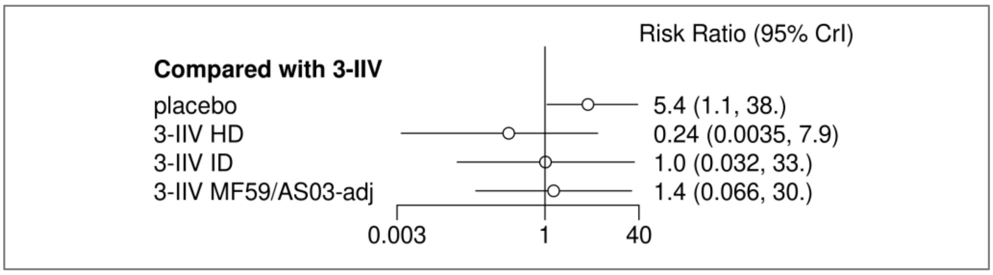

OR <1 favors experimental vaccine compared to placebo or 3-IIV

[*] **Placebo:** placebo/no vaccine; **3-IIV:** trivalent inactivated intramuscular; **3-IIV HD:** trivalent inactivated high dose intramuscular; **3-IIV ID:** trivalent inactivated intradermal; **3-IIV MF59/AS03-adj:** trivalent inactivated adjuvanted with MF59/ASO3 intramuscular: **3-IIV vir/lip-adj:** trivalent inactivated adjuvanted with Virosome/liposome intramuscular: **3-RIV:** trivalent recombinant intramuscular; **4-IIV:** quadrivalent inactivated intramuscular; **4-IIV HD:** quadrivalent inactivated high dose intramuscular: **4-IIV ID:** quadrivalent inactivated intradermal; **4-IIV MF59/AS03-adj:** quadrivalent inactivated adjuvanted with MF59/ASO3 intramuscular; **4-IIV vir/lip-adj:** quadrivalent inactivated adjuvanted with virosome/liposome intramuscular; **4-RIV:** quadrivalent recombinant intramuscular; **3-LAIV:** trivalent live attenuated intranasal; **4-LAIV:** quadrivalent live attenuated intranasal.

Immunocompromised/cancer patients: Netleague analysis of laboratory- confirmed influenza*

**placebo**	**0.19 (0.03, 0.92)**	0.04 (0, 1.96)	0.18 (0, 8.1)	0.26 (0.01, 7.74)
**5.4 (1.09, 37.91)**	**3-IIV**	0.24 (0, 7.89)	1.02 (0.03, 33.37)	1.4 (0.07, 29.93)
23.25 (0.51, 2439.31)	4.15 (0.13, 281.79)	**3-IIV HD**	4.45 (0.03, 938.25)	6 (0.06, 1022.82)
5.46 (0.12, 302.8)	0.98 (0.03, 31.37)	0.22 (0, 32.15)	**3-IIV ID**	1.38 (0.01, 137.12)
3.9 (0.13, 152.34)	0.71 (0.03, 15.1)	0.17 (0, 17.36)	0.73 (0.01, 70.38)	**3-IIV MF59/AS03-adj**

The estimate is located at the intersection of the column-defining vaccine and the row-defining vaccine. Data are RRs (95% CrI). Significant results are in bold. In the upper triangle, comparisons of treatments should be read from right to left. An RR below 1 favors the medication on the bottom right vs. the medication on the top left in the diagonal.

* **Placebo:** placebo/no vaccine; **3-IIV:** trivalent inactivated intramuscular; **3-IIV HD:** trivalent inactivated high dose intramuscular; **3-IIV ID:** trivalent inactivated intradermal; **3-IIV MF59/AS03-adj:** trivalent inactivated adjuvanted with MF59/ASO3 intramuscular: **3-IIV vir/lip-adj:** trivalent inactivated adjuvanted with Virosome/liposome intramuscular: **3-RIV:** trivalent recombinant intramuscular; **4-IIV:** quadrivalent inactivated intramuscular; **4-IIV HD:** quadrivalent inactivated high dose intramuscular: **4-IIV ID:** quadrivalent inactivated intradermal; **4-IIV MF59/AS03-adj:** quadrivalent inactivated adjuvanted with MF59/ASO3 intramuscular; **4-IIV vir/lip-adj:** quadrivalent inactivated adjuvanted with virosome/liposome intramuscular; **4-RIV:** quadrivalent recombinant intramuscular; **3-LAIV:** trivalent live attenuated intranasal; **4-LAIV:** quadrivalent live attenuated intranasal.

All children: Forest plots of laboratory confirmed influenza, comparison with placebo/no vaccine and with 3-IIV*

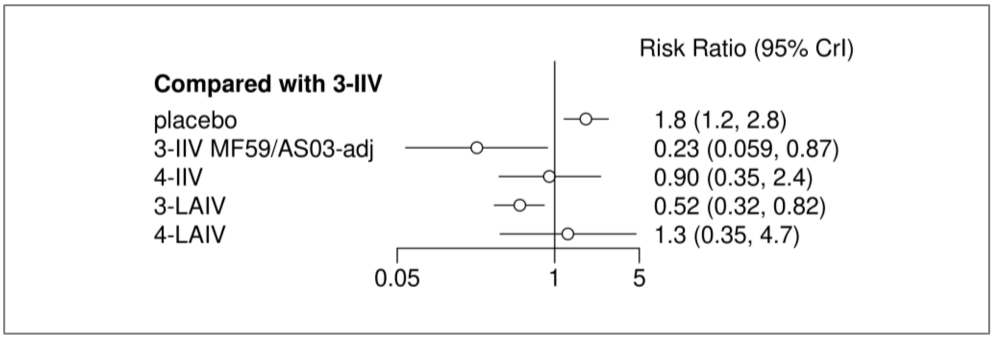

OR <1 favors experimental vaccine compared to placebo or 3-IIV

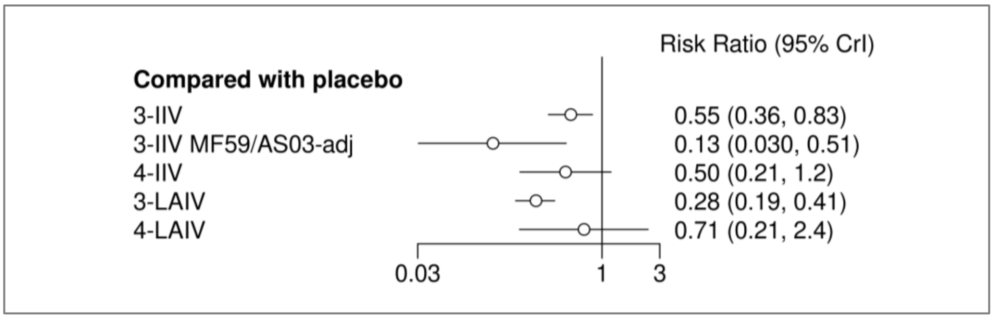

OR <1 favors experimental vaccine compared to placebo or 3-IIV

* **Placebo:** placebo/no vaccine; **3-IIV:** trivalent inactivated intramuscular; **3-IIV HD:** trivalent inactivated high dose intramuscular; **3-IIV ID:** trivalent inactivated intradermal; **3-IIV MF59/AS03-adj:** trivalent inactivated adjuvanted with MF59/ASO3 intramuscular: **3-IIV vir/lip-adj:** trivalent inactivated adjuvanted with Virosome/liposome intramuscular: **3-RIV:** trivalent recombinant intramuscular; **4-IIV:** quadrivalent inactivated intramuscular; **4-IIV HD:** quadrivalent inactivated high dose intramuscular: **4-IIV ID:** quadrivalent inactivated intradermal; **4-IIV MF59/AS03-adj:** quadrivalent inactivated adjuvanted with MF59/ASO3 intramuscular; **4-IIV vir/lip-adj:** quadrivalent inactivated adjuvanted with virosome/liposome intramuscular; **4-RIV:** quadrivalent recombinant intramuscular; **3-LAIV:** trivalent live attenuated intranasal; **4-LAIV:** quadrivalent live attenuated intranasal.

Children, aged ≤ 5 years: Network geometry of laboratory confirmed influenza*

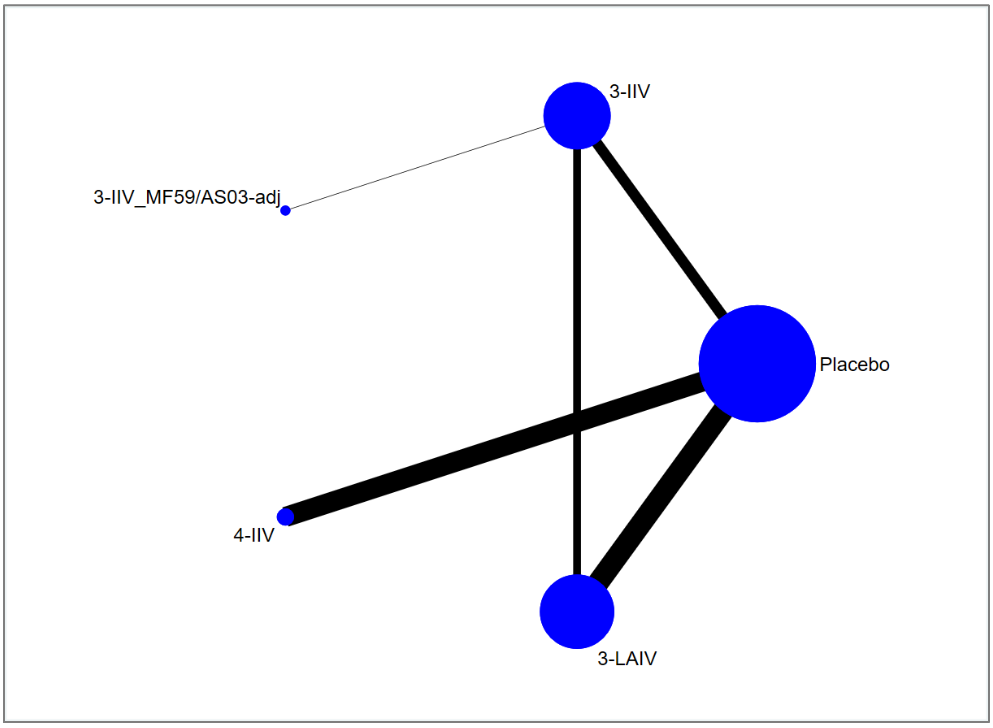

The thickness of the line is proportional to the precision of each direct estimate, and the width of each circle is proportional to the number of studies included in the treatment.

Children, aged ≤ 5 years: Forest plots of laboratory confirmed influenza, comparison with placebo/no vaccine and with 3-IIV*

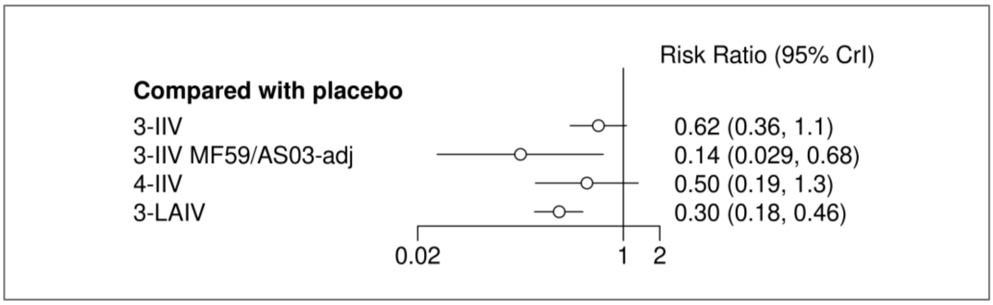

OR <1 favors experimental vaccine compared to placebo or 3-IIV

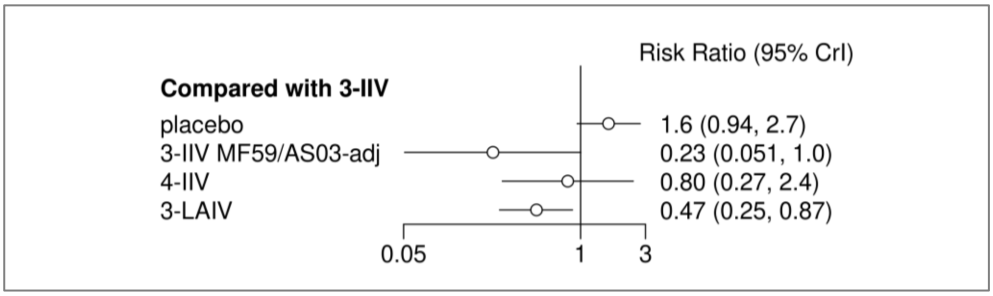

OR <1 favors experimental vaccine compared to placebo or 3-IIV

* **Placebo:** placebo/no vaccine; **3-IIV:** trivalent inactivated intramuscular; **3-IIV HD:** trivalent inactivated high dose intramuscular; **3-IIV ID:** trivalent inactivated intradermal; **3-IIV MF59/AS03-adj:** trivalent inactivated adjuvanted with MF59/ASO3 intramuscular: **3-IIV vir/lip-adj:** trivalent inactivated adjuvanted with Virosome/liposome intramuscular: **3-RIV:** trivalent recombinant intramuscular; **4-IIV:** quadrivalent inactivated intramuscular; **4-IIV HD:** quadrivalent inactivated high dose intramuscular: **4-IIV ID:** quadrivalent inactivated intradermal; **4-IIV MF59/AS03-adj:** quadrivalent inactivated adjuvanted with MF59/ASO3 intramuscular; **4-IIV vir/lip-adj:** quadrivalent inactivated adjuvanted with virosome/liposome intramuscular; **4-RIV:** quadrivalent recombinant intramuscular; **3-LAIV:** trivalent live attenuated intranasal; **4-LAIV:** quadrivalent live attenuated intranasal.

Children, aged ≤ 5 years: Netleague analysis of laboratory confirmed influenza*

**placebo**	0.62 (0.36, 1.06)	**0.14 (0.03, 0.68)**	0.5 (0.19, 1.32)	**0.3 (0.18, 0.46)**
1.61 (0.94, 2.74)	**3-IIV**	0.23 (0.05, 1)	0.8 (0.27, 2.44)	**0.47 (0.25, 0.87)**
**7.07 (1.47, 34.5)**	4.41 (1, 19.67)	**3-IIV MF59/AS03-adj**	3.55 (0.56, 22.93)	2.09 (0.42, 10.45)
2 (0.76, 5.31)	1.24 (0.41, 3.76)	0.28 (0.04, 1.79)	**4-IIV**	0.59 (0.2, 1.71)
**3.39 (2.16, 5.41)**	**2.11 (1.15, 3.94)**	0.48 (0.1, 2.41)	1.7 (0.58, 5.01)	**3-LAIV**

The estimate is located at the intersection of the column-defining vaccine and the row-defining vaccine. Data are RRs (95% CrI). Significant results are in bold. In the upper triangle, comparisons of treatments should be read from right to left. An RR below 1 favors the medication on the bottom right vs. the medication on the top left in the diagonal.

* **Placebo:** placebo/no vaccine; **3-IIV:** trivalent inactivated intramuscular; **3-IIV HD:** trivalent inactivated high dose intramuscular; **3-IIV ID:** trivalent inactivated intradermal; **3-IIV MF59/AS03-adj:** trivalent inactivated adjuvanted with MF59/ASO3 intramuscular: **3-IIV vir/lip-adj:** trivalent inactivated adjuvanted with Virosome/liposome intramuscular: **3-RIV:** trivalent recombinant intramuscular; **4-IIV:** quadrivalent inactivated intramuscular; **4-IIV HD:** quadrivalent inactivated high dose intramuscular: **4-IIV ID:** quadrivalent inactivated intradermal; **4-IIV MF59/AS03-adj:** quadrivalent inactivated adjuvanted with MF59/ASO3 intramuscular; **4-IIV vir/lip-adj:** quadrivalent inactivated adjuvanted with virosome/liposome intramuscular; **4-RIV:** quadrivalent recombinant intramuscular; **3-LAIV:** trivalent live attenuated intranasal; **4-LAIV:** quadrivalent live attenuated intranasal.

Children with pre-existing respiratory diseases: Network geometry of laboratory- confirmed influenza[*]

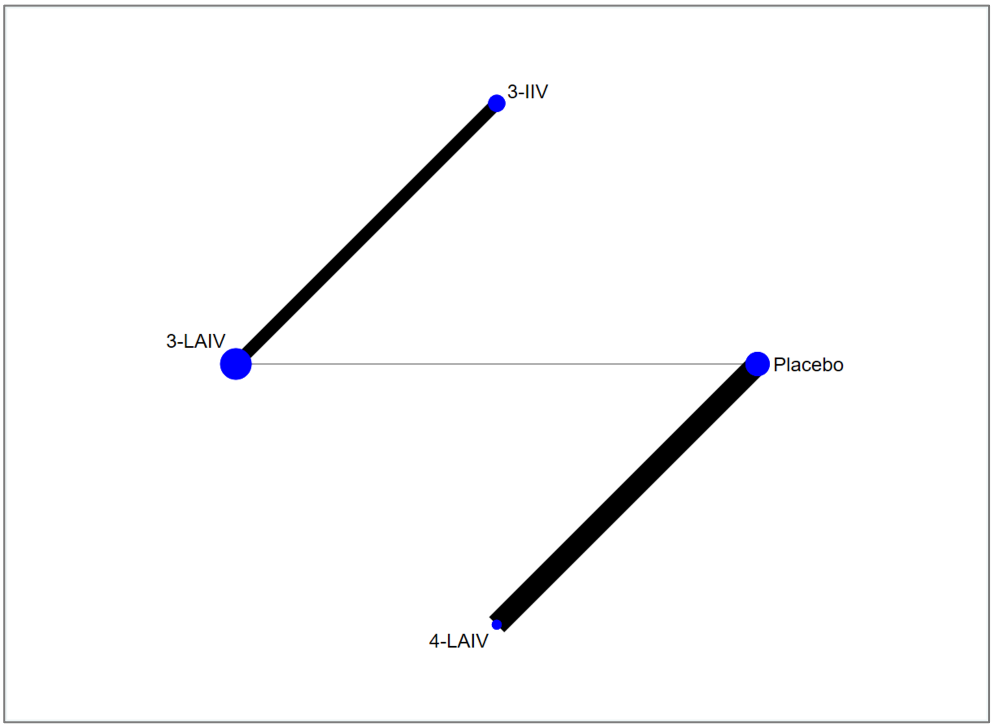

The thickness of the line is proportional to the precision of each direct estimate, and the width of each circle is proportional to the number of studies included in the treatment.

Children with pre-existing respiratory diseases: Forest plots of laboratory confirmed influenza, comparison with placebo-no vaccine and with 3-IIV[*]

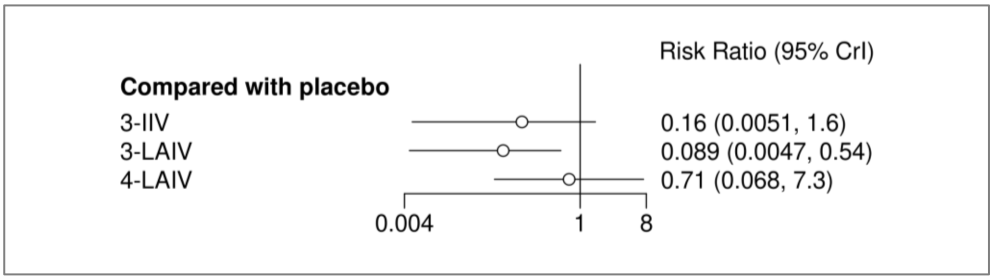

OR <1 favors experimental vaccine compared to placebo or 3-IIV

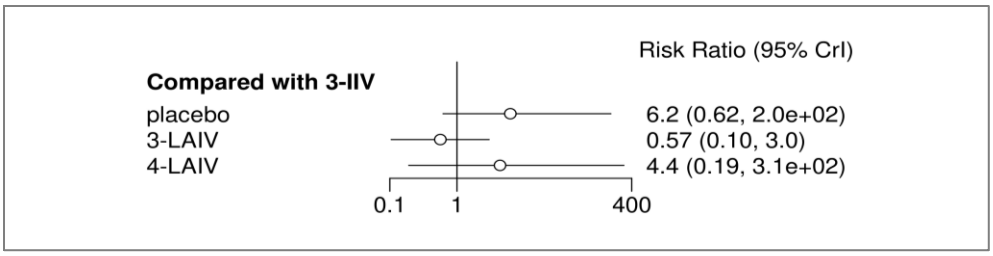

OR <1 favors experimental vaccine compared to placebo or 3-IIV

[*] **Placebo:** placebo/no vaccine; **3-IIV:** trivalent inactivated intramuscular; **3-IIV HD:** trivalent inactivated high dose intramuscular; **3-IIV ID:** trivalent inactivated intradermal; **3-IIV MF59/AS03-adj:** trivalent inactivated adjuvanted with MF59/ASO3 intramuscular: **3-IIV vir/lip-adj:** trivalent inactivated adjuvanted with Virosome/liposome intramuscular: **3-RIV:** trivalent recombinant intramuscular; **4-IIV:** quadrivalent inactivated intramuscular; **4-IIV HD:** quadrivalent inactivated high dose intramuscular: **4-IIV ID:** quadrivalent inactivated intradermal; **4-IIV MF59/AS03-adj:** quadrivalent inactivated adjuvanted with MF59/ASO3 intramuscular; **4-IIV vir/lip-adj:** quadrivalent inactivated adjuvanted with virosome/liposome intramuscular; **4-RIV:** quadrivalent recombinant intramuscular; **3-LAIV:** trivalent live attenuated intranasal; **4-LAIV:** quadrivalent live attenuated intranasal.

Children with pre-existing respiratory diseases: Netleague analysis of laboratory- confirmed influenza*

**placebo**	0.16 (0.01, 1.61)	**0.09 (0, 0.54)**	0.71 (0.07, 7.32)
6.23 (0.62, 195.63)	**3-IIV**	0.57 (0.1, 3.01)	4.37 (0.19, 305.6)
**11.23 (1.84, 213.35)**	1.77 (0.33, 9.54)	**3-LAIV**	7.82 (0.51, 372.38)
1.41 (0.14, 14.74)	0.23 (0, 5.22)	0.13 (0, 1.98)	**4-LAIV**

The estimate Is located at the Intersection of the column-defining vaccine and the row-defining vaccine. Data are RRs (95% CrI). Significant results are in bold. In the upper triangle, comparisons of treatments should be read from right to left. An RR below 1 favors the medication on the bottom right vs. the medication on the top left in the diagonal.

* **Placebo:** placebo/no vaccine; **3-IIV:** trivalent inactivated intramuscular; **3-IIV HD:** trivalent inactivated high dose intramuscular; **3-IIV ID:** trivalent inactivated intradermal; **3-IIV MF59/AS03-adj:** trivalent inactivated adjuvanted with MF59/ASO3 intramuscular: **3-IIV vir/lip-adj:** trivalent inactivated adjuvanted with Virosome/liposome intramuscular: **3-RIV:** trivalent recombinant intramuscular; **4-IIV:** quadrivalent inactivated intramuscular; **4-IIV HD:** quadrivalent inactivated high dose intramuscular: **4-IIV ID:** quadrivalent inactivated intradermal; **4-IIV MF59/AS03-adj:** quadrivalent inactivated adjuvanted with MF59/ASO3 intramuscular; **4-IIV vir/lip-adj:** quadrivalent inactivated adjuvanted with virosome/liposome intramuscular; **4-RIV:** quadrivalent recombinant intramuscular; **3-LAIV:** trivalent live attenuated intranasal; **4-LAIV:** quadrivalent live attenuated intranasal.

Adults and the elderly: Network geometry of hospitalization rate[*]

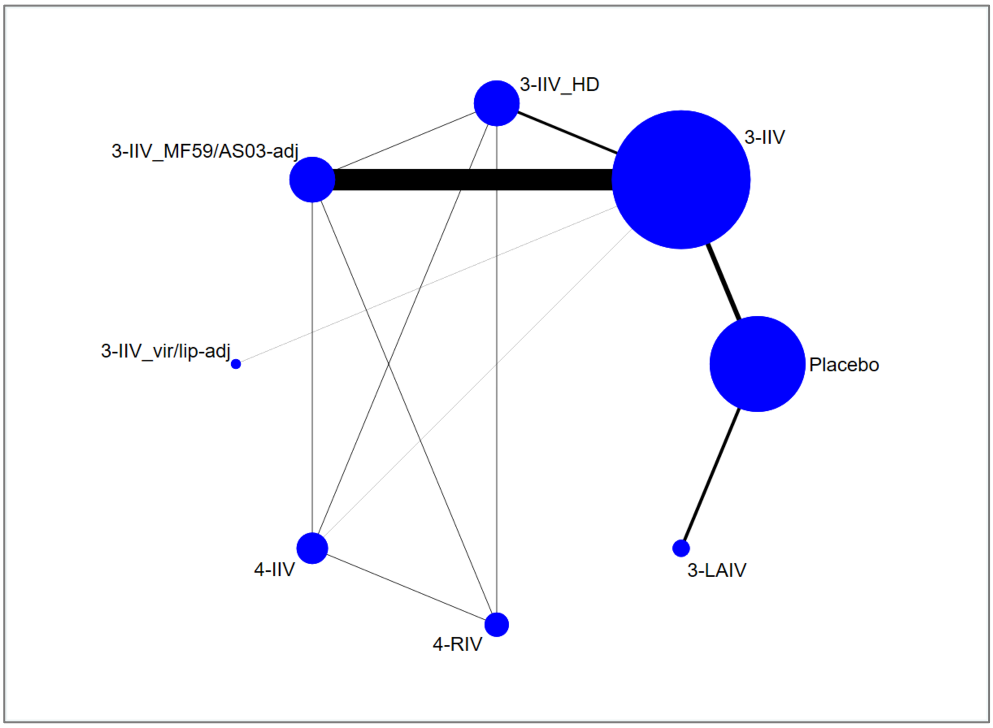

The thickness of the line is proportional to the precision of each direct estimate, and the width of each circle is proportional to the number of studies included in the treatment.

Adults and the elderly: Forest plots of hosptitalization rate, comparison with placebo-no vaccine and with 3-IIV[*]

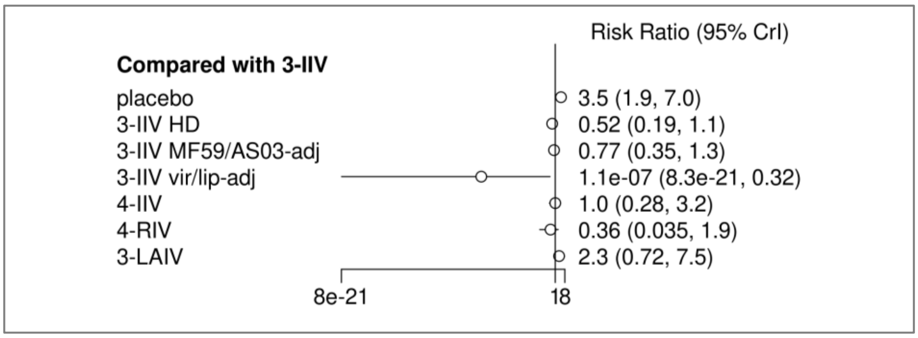

OR <1 favors experimental vaccine compared to placebo or 3-IIV

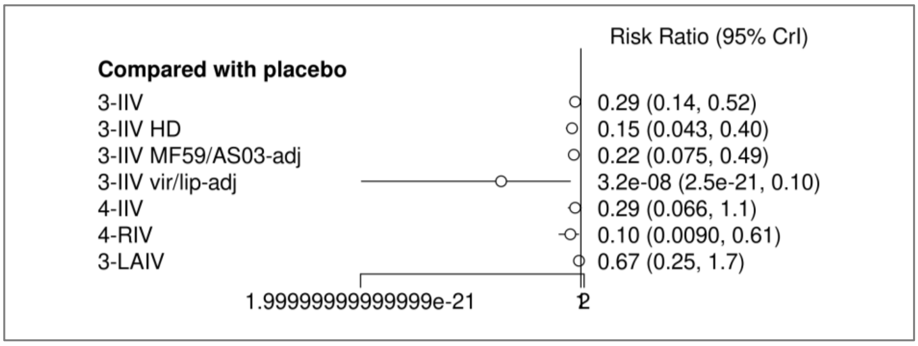

OR <1 favors experimental vaccine compared to placebo or 3-IIV

[*] **Placebo:** placebo/no vaccine; **3-IIV:** trivalent inactivated intramuscular; **3-IIV HD:** trivalent inactivated high dose intramuscular; **3-IIV ID:** trivalent inactivated intradermal; **3-IIV MF59/AS03-adj:** trivalent inactivated adjuvanted with MF59/ASO3 intramuscular: **3-IIV vir/lip-adj:** trivalent inactivated adjuvanted with Virosome/liposome intramuscular: **3-RIV:** trivalent recombinant intramuscular; **4-IIV:** quadrivalent inactivated intramuscular; **4-IIV HD:** quadrivalent inactivated high dose intramuscular: **4-IIV ID:** quadrivalent inactivated intradermal; **4-IIV MF59/AS03-adj:** quadrivalent inactivated adjuvanted with MF59/ASO3 intramuscular; **4-IIV vir/lip-adj:** quadrivalent inactivated adjuvanted with virosome/liposome intramuscular; **4-RIV:** quadrivalent recombinant intramuscular; **3-LAIV:** trivalent live attenuated intranasal; **4-LAIV:** quadrivalent live attenuated intranasal.

Adults and the eldelry: Netleague analysis of hospitalization rate*

**placebo**	**0.29 (0.14, 0.52)**	**0.15 (0.04, 0.4)**	**0.22 (0.08, 0.49)**	**0 (0, 0.1)**	0.29 (0.07, 1.06)	**0.1 (0.01, 0.61)**	0.67 (0.25, 1.66)
**3.47 (1.91, 7.02)**	**3-IIV**	0.52 (0.19, 1.14)	0.77 (0.35, 1.27)	**0 (0, 0.32)**	1 (0.28, 3.23)	0.36 (0.04, 1.88)	2.31 (0.72, 7.54)
**6.76 (2.52, 23.05)**	1.93 (0.88, 5.18)	**3-IIV HD**	1.48 (0.56, 3.83)	**0 (0, 0.63)**	1.96 (0.57, 6.84)	0.71 (0.07, 3.87)	**4.51 (1.13, 20.77)**
**4.58 (2.04, 13.33)**	1.3 (0.79, 2.83)	0.68 (0.26, 1.79)	**3-IIV MF59/AS03-adj**	**0 (0, 0.43)**	1.34 (0.41, 4.55)	0.48 (0.05, 2.61)	3.06 (0.83, 12.62)
**30972861.93 (9.94, 4.04665306984859e+20)**	**8944012.79 (3.11, 1.19969832201764e+20)**	**4505391.98 (1.59, 666638542409784688 64)**	**6471391.46 (2.35, 912740488052805468 16)**	**3-IIV vir/lip-adj**	**9063508.09 (2.86, 1.25859629335449e+20)**	3210895.96 (0.91, 463010584647364935 68)	**21054113.46 (5.47, 2.87605336845292e+20)**
3.46 (0.94, 15.13)	1 (0.31, 3.53)	0.51 (0.15, 1.74)	0.75 (0.22, 2.46)	**0 (0, 0.35)**	**4-IIV**	0.36 (0.04, 2.06)	2.29 (0.45, 12.93)
**9.72 (1.64, 110.92)**	2.76 (0.53, 28.46)	1.41 (0.26, 13.75)	2.07 (0.38, 20.33)	**0 (0, 1.1)**	2.75 (0.48, 27.71)	**4-RIV**	6.45 (0.86, 91.74)
1.5 (0.6, 4.07)	0.43 (0.13, 1.39)	**0.22 (0.05, 0.89)**	0.33 (0.08, 1.2)	**0 (0, 0.18)**	0.44 (0.08, 2.23)	0.16 (0.01, 1.16)	**3-LAIV**

The estimate is located at the intersection of the column-defining vaccine and the row-defining vaccine. Data are RRs (95% CrI). Significant results are in bold. In the upper triangle, comparisons of treatments should be read from right to left. An RR below 1 favors the medication on the bottom right vs. the medication on the top left in the diagonal.

* **Placebo:** placebo/no vaccine; **3-IIV:** trivalent inactivated intramuscular; **3-IIV HD:** trivalent inactivated high dose intramuscular; **3-IIV ID:** trivalent inactivated intradermal; **3-IIV MF59/AS03-adj:** trivalent inactivated adjuvanted with MF59/ASO3 intramuscular: **3-IIV vir/lip-adj:** trivalent inactivated adjuvanted with Virosome/liposome intramuscular: **3-RIV:** trivalent recombinant intramuscular; **4-IIV:** quadrivalent inactivated intramuscular; **4-IIV HD:** quadrivalent inactivated high dose intramuscular: **4-IIV ID:** quadrivalent inactivated intradermal; **4-IIV MF59/AS03-adj:** quadrivalent inactivated adjuvanted with MF59/ASO3 intramuscular; **4-IIV vir/lip-adj:** quadrivalent inactivated adjuvanted with virosome/liposome intramuscular; **4-RIV:** quadrivalent recombinant intramuscular; **3-LAIV:** trivalent live attenuated intranasal; **4-LAIV:** quadrivalent live attenuated intranasal.

All children: Network geometry of hospitalization rate*

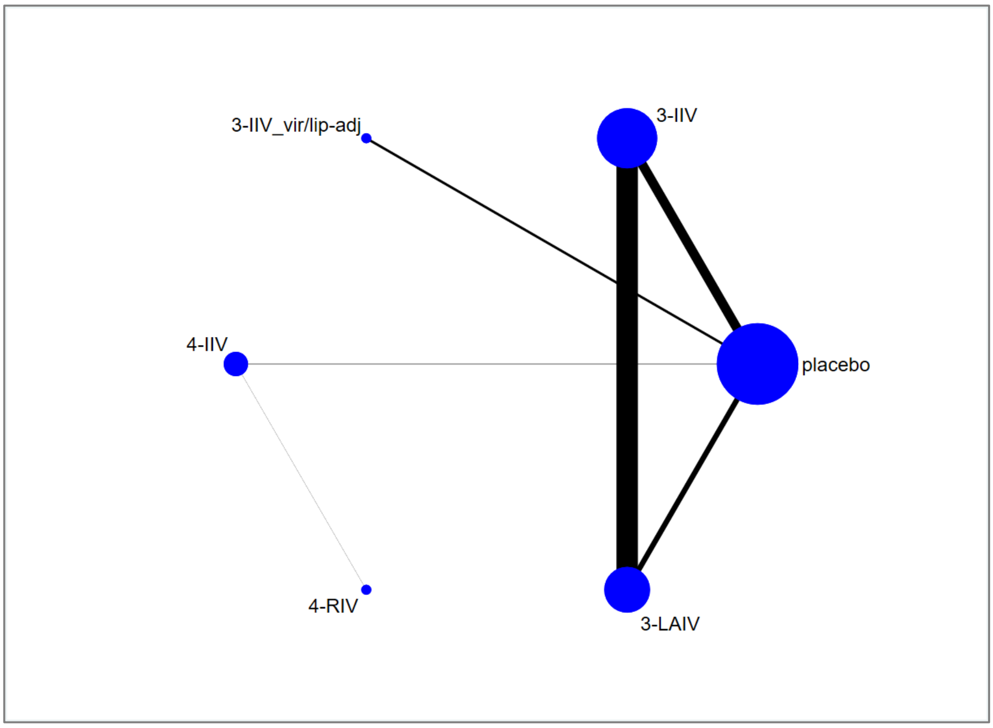

The thickness of the line is proportional to the precision of each direct estimate, and the width of each circle is proportional to the number of studies included in the treatment.

All children: Forest plots of hospitalization rate, comparison with placebo/no vaccine and with 3-IIV*

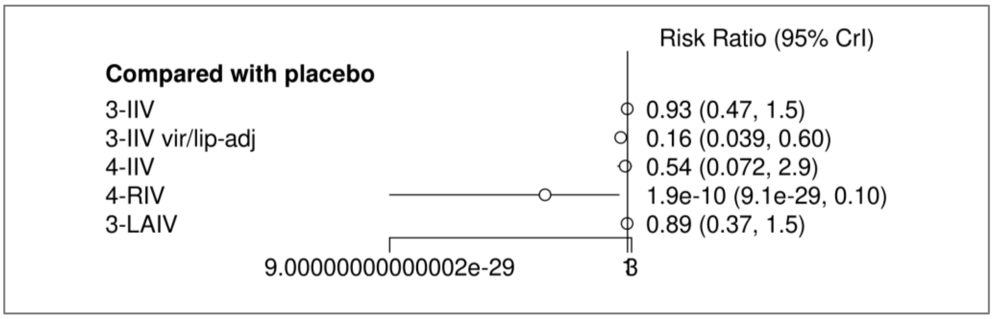

OR <1 favors experimental vaccine compared to placebo or 3-IIV

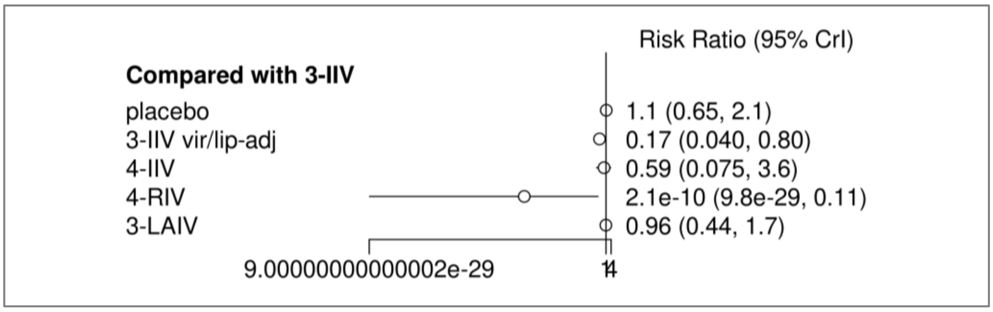

OR <1 favors experimental vaccine compared to placebo or 3-IIV

* **Placebo:** placebo/no vaccine; **3-IIV:** trivalent inactivated intramuscular; **3-IIV HD:** trivalent inactivated high dose intramuscular; **3-IIV ID:** trivalent inactivated intradermal; **3-IIV MF59/AS03-adj:** trivalent inactivated adjuvanted with MF59/ASO3 intramuscular: **3-IIV vir/lip-adj:** trivalent inactivated adjuvanted with Virosome/liposome intramuscular: **3-RIV:** trivalent recombinant intramuscular; **4-IIV:** quadrivalent inactivated intramuscular; **4-IIV HD:** quadrivalent inactivated high dose intramuscular: **4-IIV ID:** quadrivalent inactivated intradermal; **4-IIV MF59/AS03-adj:** quadrivalent inactivated adjuvanted with MF59/ASO3 intramuscular; **4-IIV vir/lip-adj:** quadrivalent inactivated adjuvanted with virosome/liposome intramuscular; **4-RIV:** quadrivalent recombinant intramuscular; **3-LAIV:** trivalent live attenuated intranasal; **4-LAIV:** quadrivalent live attenuated intranasal.

All children. Netleague analysis of hospitalization rate*

**placebo**	0.93 (0.47, 1.55)	**0.16 (0.04, 0.6)**	0.54 (0.07, 2.94)	**0 (0, 0.1)**	0.89 (0.37, 1.53)
1.08 (0.65, 2.11)	**3-IIV**	**0.17 (0.04, 0.8)**	0.59 (0.08, 3.57)	**0 (0, 0.11)**	0.96 (0.44, 1.72)
**6.33 (1.66, 25.63)**	**5.9 (1.25, 24.81)**	**3-IIV vir/lip-adj**	3.46 (0.29, 29.68)	**0 (0, 0.69)**	**5.65 (1.04, 22.7)**
1.84 (0.34, 13.92)	1.7 (0.28, 13.29)	0.29 (0.03, 3.46)	**4-IIV**	**0 (0, 0.17)**	1.61 (0.24, 12.28)
**5340664028.94 (9.96, 1.096985824046e+28)**	**4814636394.71 (8.84, 1.01619959257508e+28)**	**807035612.15 (1.45, 1.63374882187751e+27)**	**2736904700.67 (5.74, 5.91394639740442e+27)**	**4-RIV**	**4404777933.57 (8.32, 8.91148853023516e+27)**
1.12 (0.65, 2.73)	1.04 (0.58, 2.3)	**0.18 (0.04, 0.97)**	0.62 (0.08, 4.11)	**0 (0, 0.12)**	**3-LAIV**

The estimate is located at the intersection of the column-defining vaccine and the row-defining vaccine. Data are RRs (95% CrI). Significant results are in bold. In the upper triangle, comparisons of treatments should be read from right to left. An RR below 1 favors the medication on the bottom right vs. the medication on the top left in the diagonal.

* **Placebo:** placebo/no vaccine; **3-IIV:** trivalent inactivated intramuscular; **3-IIV HD:** trivalent inactivated high dose intramuscular; **3-IIV ID:** trivalent inactivated intradermal; **3-IIV MF59/AS03-adj:** trivalent inactivated adjuvanted with MF59/ASO3 intramuscular: **3-IIV vir/lip-adj:** trivalent inactivated adjuvanted with Virosome/liposome intramuscular: **3-RIV:** trivalent recombinant intramuscular; **4-IIV:** quadrivalent inactivated intramuscular; **4-IIV HD:** quadrivalent inactivated high dose intramuscular: **4-IIV ID:** quadrivalent inactivated intradermal; **4-IIV MF59/AS03-adj:** quadrivalent inactivated adjuvanted with MF59/ASO3 intramuscular; **4-IIV vir/lip-adj:** quadrivalent inactivated adjuvanted with virosome/liposome intramuscular; **4-RIV:** quadrivalent recombinant intramuscular; **3-LAIV:** trivalent live attenuated intranasal; **4-LAIV:** quadrivalent live attenuated intranasal.

Adults and the elderly: Network geometry of mortality of any cause*

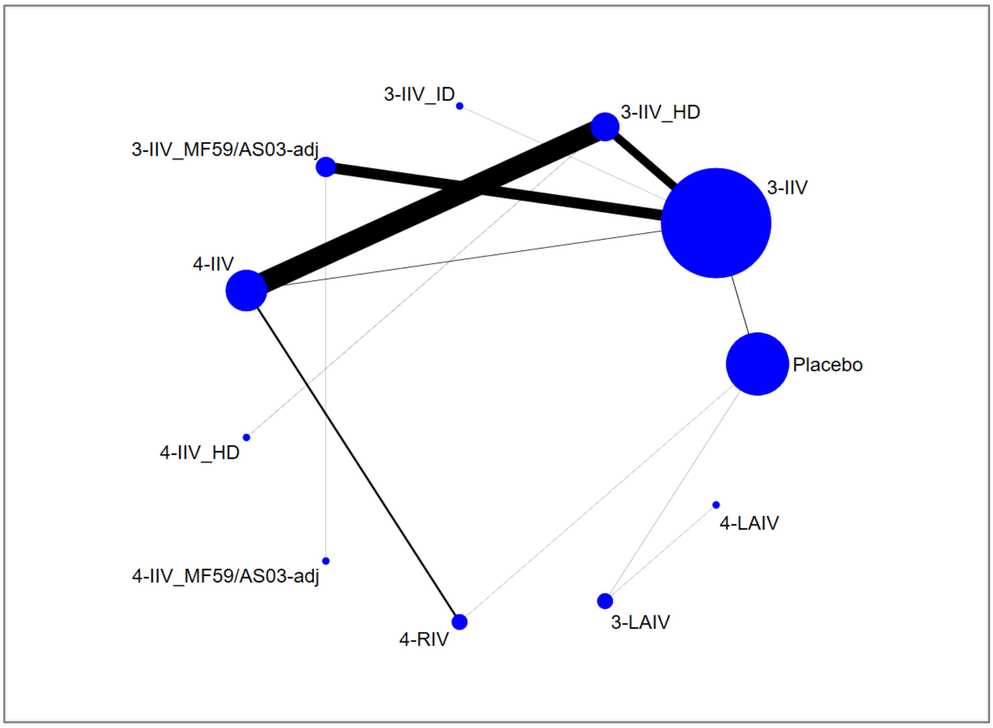

The thickness of the line is proportional to the precision of each direct estimate, and the width of each circle is proportional to the number of studies included in the treatment.

* **Placebo:** placebo/no vaccine; **3-IIV:** trivalent inactivated intramuscular; **3-IIV HD:** trivalent inactivated high dose intramuscular; **3-IIV ID:** trivalent inactivated intradermal; **3-IIV MF59/AS03-adj:** trivalent inactivated adjuvanted with MF59/ASO3 intramuscular: **3-IIV vir/lip-adj:** trivalent inactivated adjuvanted with Virosome/liposome intramuscular: **3-RIV:** trivalent recombinant intramuscular; **4-IIV:** quadrivalent inactivated intramuscular; **4-IIV HD:** quadrivalent inactivated high dose intramuscular: **4-IIV ID:** quadrivalent inactivated intradermal; **4-IIV MF59/AS03-adj:** quadrivalent inactivated adjuvanted with MF59/ASO3 intramuscular; **4-IIV vir/lip-adj:** quadrivalent inactivated adjuvanted with virosome/liposome intramuscular; **4-RIV:** quadrivalent recombinant intramuscular; **3-LAIV:** trivalent live attenuated intranasal; **4-LAIV:** quadrivalent live attenuated intranasal.

Adults and the elderly: Forest plots of mortality of any cause, comparison with placebo/no vaccine and with 3-IIV[*]

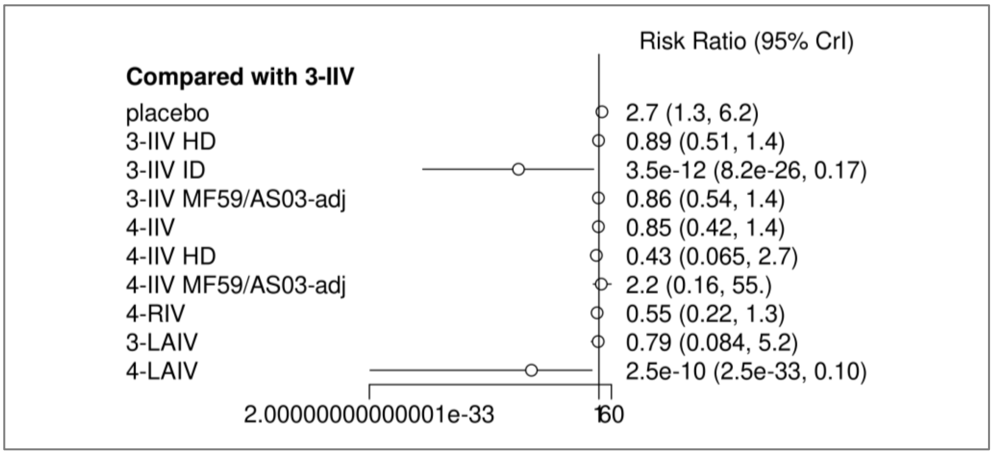

OR <1 favors experimental vaccine compared to placebo or 3-IIV

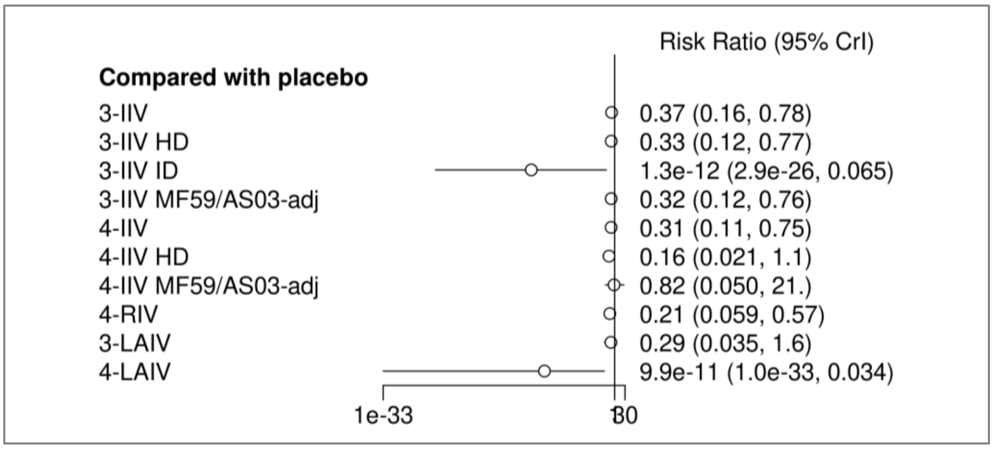

OR <1 favors experimental vaccine compared to placebo or 3-IIV

[*] **Placebo:** placebo/no vaccine; **3-IIV:** trivalent inactivated intramuscular; **3-IIV HD:** trivalent inactivated high dose intramuscular; **3-IIV ID:** trivalent inactivated intradermal; **3-IIV MF59/AS03-adj:** trivalent inactivated adjuvanted with MF59/ASO3 intramuscular: **3-IIV vir/lip-adj:** trivalent inactivated adjuvanted with Virosome/liposome intramuscular: **3-RIV:** trivalent recombinant intramuscular; **4-IIV:** quadrivalent inactivated intramuscular; **4-IIV HD:** quadrivalent inactivated high dose intramuscular: **4-IIV ID:** quadrivalent inactivated intradermal; **4-IIV MF59/AS03-adj:** quadrivalent inactivated adjuvanted with MF59/ASO3 intramuscular; **4-IIV vir/lip-adj:** quadrivalent inactivated adjuvanted with virosome/liposome intramuscular; **4-RIV:** quadrivalent recombinant intramuscular; **3-LAIV:** trivalent live attenuated intranasal; **4-LAIV:** quadrivalent live attenuated intranasal.

Adults and the elderly: Netleague analysis of mortality of any cause*

**placebo**	**0.37 (0.16, 0.78)**	**0.33 (0.12, 0.77)**	**0 (0, 0.06)**	**0.32 (0.12, 0.76)**	**0.31 (0.11, 0.75)**	0.16 (0.02, 1.08)	0.82 (0.05, 20.72)	**0.21 (0.06, 0.57)**	0.29 (0.04, 1.56)	**0 (0, 0.03)**
**2.71 (1.28, 6.22)**	**3-IIV**	0.89 (0.51, 1.37)	**0 (0, 0.17)**	0.86 (0.54, 1.43)	0.85 (0.42, 1.43)	0.43 (0.07, 2.69)	2.19 (0.16, 54.78)	0.55 (0.22, 1.26)	0.79 (0.08, 5.2)	**0 (0, 0.1)**
**3.07 (1.3, 8.25)**	1.12 (0.73, 1.95)	**3-IIV HD**	**0 (0, 0.2)**	0.95 (0.53, 2.1)	0.96 (0.53, 1.52)	0.49 (0.08, 2.91)	2.51 (0.17, 64.74)	0.62 (0.26, 1.4)	0.9 (0.09, 6.36)	**0 (0, 0.12)**
**757095718859.98 (15.39, 3.49771330636616e+25)**	**286187249246.17 (5.75, 1.22597994455618e+25)**	**245802862233.05 (4.94, 1.19251401004246e+25)**	**3-IIV-IDl**	**251621058355.28 (5.08, 1.0878170368198e+25)**	**227098867929.96 (4.81, 1.10843485845615e+25)**	**107043896605.87 (2.76, 9.96753916952377e+24)**	**1019261265498.14 (9.64, 4.75011699149773e+25)**	**148531142519.75 (3.17, 7.85689848306889e+24)**	**286361211425.39 (4.15, 1.51655217666623e+25)**	3.29 (0, 5639755709702273024)
**3.16 (1.32, 8.12)**	1.17 (0.7, 1.85)	1.05 (0.48, 1.9)	**0 (0, 0.2)**	**3-IIV MF59/AS03-adj**	0.99 (0.4, 1.91)	0.5 (0.07, 3.29)	2.53 (0.19, 60.55)	0.64 (0.22, 1.62)	0.92 (0.09, 6.38)	**0 (0, 0.12)**
**3.2 (1.34, 9.33)**	1.18 (0.7, 2.38)	1.05 (0.66, 1.89)	**0 (0, 0.21)**	1.01 (0.52, 2.52)	**4-IIV**	0.52 (0.08, 3.38)	2.65 (0.18, 71.89)	0.66 (0.33, 1.3)	0.95 (0.1, 7.05)	**0 (0, 0.13)**
6.36 (0.93, 48.16)	2.31 (0.37, 15.35)	2.04 (0.34, 12.32)	**0 (0, 0.36)**	1.99 (0.3, 14.2)	1.93 (0.3, 12.39)	**4-IIV-HD**	5.19 (0.21, 217.7)	1.28 (0.18, 9.28)	1.78 (0.1, 26.11)	**0 (0, 0.27)**
1.22 (0.05, 19.84)	0.46 (0.02, 6.32)	0.4 (0.02, 5.93)	**0 (0, 0.1)**	0.4 (0.02, 5.18)	0.38 (0.01, 5.54)	0.19 (0, 4.78)	**4-IIV MF59/AS03-adj**	0.25 (0.01, 4.04)	0.33 (0.01, 9.06)	**0 (0, 0.07)**
**4.86 (1.74, 16.81)**	1.81 (0.8, 4.62)	1.61 (0.72, 3.78)	**0 (0, 0.32)**	1.55 (0.62, 4.65)	1.52 (0.77, 3)	0.78 (0.11, 5.66)	4.07 (0.25, 116.16)	**4-RIV**	1.44 (0.14, 11.53)	**0 (0, 0.2)**
3.42 (0.64, 28.48)	1.27 (0.19, 11.86)	1.12 (0.16, 10.95)	**0 (0, 0.24)**	1.09 (0.16, 10.92)	1.06 (0.14, 10.5)	0.56 (0.04, 9.63)	3 (0.11, 132.65)	0.69 (0.09, 7.17)	**3-LAIV**	**0 (0, 0.12)**
**10107830726.22 (29.6, 9.55809160525854e+32)**	**4076335867.09 (9.74, 3.92443796769834e+32)**	**3601897655.47 (8.1, 3.8375142539474e+32)**	0.3 (0, 5.28807403165517e+21)	**3562104292.46 (8.34, 3.25877607064719e+32)**	**3365344196.61 (7.45, 3.64407653840852e+32)**	**1795204173.83 (3.73, 1.86674927401118e+32)**	**15586288987.02 (14.99, 1.36743352397567e+33)**	**2241877333.4 (5.11, 2.41429775310055e+32)**	**2707506275.43 (8.65, 1.93529251849772e+32)**	**4-LAIV**

The estimate is located at the intersection of the column-defining vaccine and the row-defining vaccine. Data are RRs (95% CrI). Significant results are in bold. In the upper triangle, comparisons of treatments should be read from right to left. An RR below 1 favors the medication on the bottom right vs. the medication on the top left in the diagonal.

* **Placebo:** placebo/no vaccine; **3-IIV:** trivalent inactivated intramuscular; **3-IIV HD:** trivalent inactivated high dose intramuscular; **3-IIV ID:** trivalent inactivated intradermal; **3-IIV MF59/AS03-adj:** trivalent inactivated adjuvanted with MF59/ASO3 intramuscular: **3-IIV vir/lip-adj:** trivalent inactivated adjuvanted with Virosome/liposome intramuscular: **3-RIV:** trivalent recombinant intramuscular; **4-IIV:** quadrivalent inactivated intramuscular; **4-IIV HD:** quadrivalent inactivated high dose intramuscular: **4-IIV ID:** quadrivalent inactivated intradermal; **4-IIV MF59/AS03-adj:** quadrivalent inactivated adjuvanted with MF59/ASO3 intramuscular; **4-IIV vir/lip-adj:** quadrivalent inactivated adjuvanted with virosome/liposome intramuscular; **4-RIV:** quadrivalent recombinant intramuscular; **3-LAIV:** trivalent live attenuated intranasal; **4-LAIV:** quadrivalent live attenuated intranasal.

All children: Network geometry of mortality of any cause*

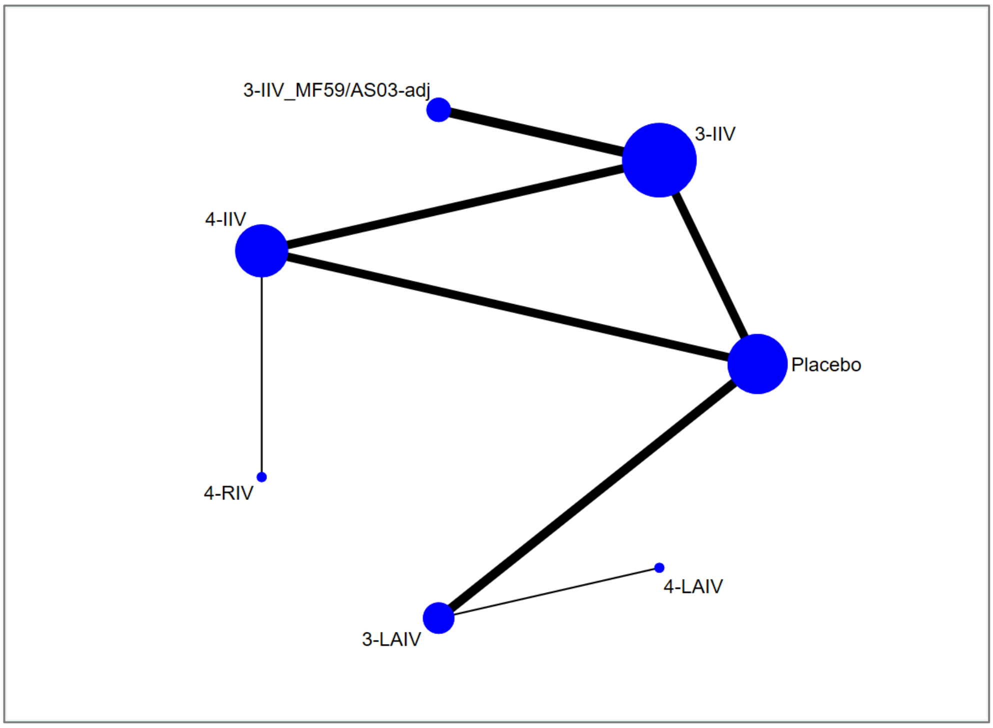

The thickness of the line is proportional to the precision of each direct estimate, and the width of each circle is proportional to the number of studies included in the treatment.

All children: Forest plots of mortality of any cause, comparison with placebo/no vaccine and with 3-IIV*

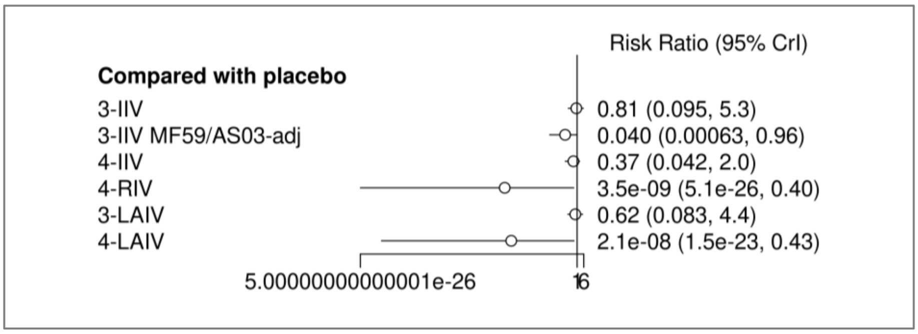

OR <1 favors experimental vaccine compared to placebo or 3-IIV

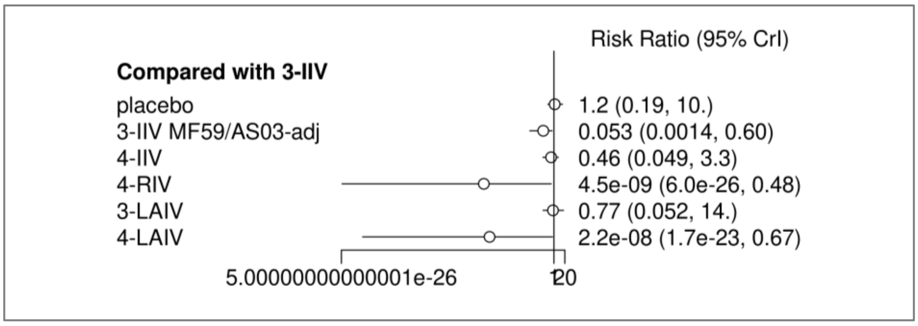

OR <1 favors experimental vaccine compared to placebo or 3-IIV

* **Placebo:** placebo/no vaccine; **3-IIV:** trivalent inactivated intramuscular; **3-IIV HD:** trivalent inactivated high dose intramuscular; **3-IIV ID:** trivalent inactivated intradermal; **3-IIV MF59/AS03-adj:** trivalent inactivated adjuvanted with MF59/ASO3 intramuscular: **3-IIV vir/lip-adj:** trivalent inactivated adjuvanted with Virosome/liposome intramuscular: **3-RIV:** trivalent recombinant intramuscular; **4-IIV:** quadrivalent inactivated intramuscular; **4-IIV HD:** quadrivalent inactivated high dose intramuscular: **4-IIV ID:** quadrivalent inactivated intradermal; **4-IIV MF59/AS03-adj:** quadrivalent inactivated adjuvanted with MF59/ASO3 intramuscular; **4-IIV vir/lip-adj:** quadrivalent inactivated adjuvanted with virosome/liposome intramuscular; **4-RIV:** quadrivalent recombinant intramuscular; **3-LAIV:** trivalent live attenuated intranasal; **4-LAIV:** quadrivalent live attenuated intranasal.

All children: Netleague analysis of mortality of any cause*

**placebo**	0.81 (0.1, 5.29)	**0.04 (0, 0.96)**	0.37 (0.04, 1.99)	**0 (0, 0.4)**	0.62 (0.08, 4.4)	**0 (0, 0.43)**
1.23 (0.19, 10.48)	**3-IIV**	**0.05 (0, 0.6)**	0.46 (0.05, 3.31)	**0 (0, 0.48)**	0.77 (0.05, 13.81)	**0 (0, 0.67)**
**24.98 (1.04, 1590.74)**	**18.73 (1.67, 733.69)**	**3-IIV MF59/AS03-adj**	9.16 (0.32, 495.19)	0 (0, 15.98)	15.68 (0.38, 1503.99)	0 (0, 23.08)
2.67 (0.5, 24.07)	2.17 (0.3, 20.38)	0.11 (0, 3.17)	**4-IIV**	**0 (0, 0.85)**	1.66 (0.13, 33.67)	0 (0, 1.52)
**282779832.13 (2.48, 1.94616517443502e+25)**	**221298718.92 (2.09, 1.67813234994266e+25)**	10423201.26 (0.06, 8.60967118893389e+23)	**95951450.43 (1.18, 5.93571191264789e+24)**	**4-RIV**	**178659206.29 (1.36, 1.14939258421012e+25)**	7.51 (0, 4489974184834196992)
1.61 (0.23, 11.99)	1.3 (0.07, 19.33)	0.06 (0, 2.66)	0.6 (0.03, 7.46)	**0 (0, 0.74)**	**3 LAIV**	**0 (0, 0.55)**
**48385226.43 (2.31, 6.76804260880723e+22)**	**46155629.06 (1.49, 5.95182453746959e+22)**	1897383.53 (0.04, 4.9873964888541e+21)	21705948.16 (0.66, 2.73166229700137e+22)	0.13 (0, 19521316104692516)	**32696951.53 (1.83, 3.64266067903709e+22)**	**4 LAIV**

The estimate is located at the intersection of the column-defining vaccine and the row-defining vaccine. Data are RRs (95% CrI). Significant results are in bold. In the upper triangle, comparisons of treatments should be read from right to left. An RR below 1 favors the medication on the bottom right vs. the medication on the top left in the diagonal.

.

* **Placebo:** placebo/no vaccine; **3-IIV:** trivalent inactivated intramuscular; **3-IIV HD:** trivalent inactivated high dose intramuscular; **3-IIV ID:** trivalent inactivated intradermal; **3-IIV MF59/AS03-adj:** trivalent inactivated adjuvanted with MF59/ASO3 intramuscular: **3-IIV vir/lip-adj:** trivalent inactivated adjuvanted with Virosome/liposome intramuscular: **3-RIV:** trivalent recombinant intramuscular; **4-IIV:** quadrivalent inactivated intramuscular; **4-IIV HD:** quadrivalent inactivated high dose intramuscular: **4-IIV ID:** quadrivalent inactivated intradermal; **4-IIV MF59/AS03-adj:** quadrivalent inactivated adjuvanted with MF59/ASO3 intramuscular; **4-IIV vir/lip-adj:** quadrivalent inactivated adjuvanted with virosome/liposome intramuscular; **4-RIV:** quadrivalent recombinant intramuscular; **3-LAIV:** trivalent live attenuated intranasal; **4-LAIV:** quadrivalent live attenuated intranasal.

Adults and the elderly: Network geometry of Influenza like Illness (ILI) [*]

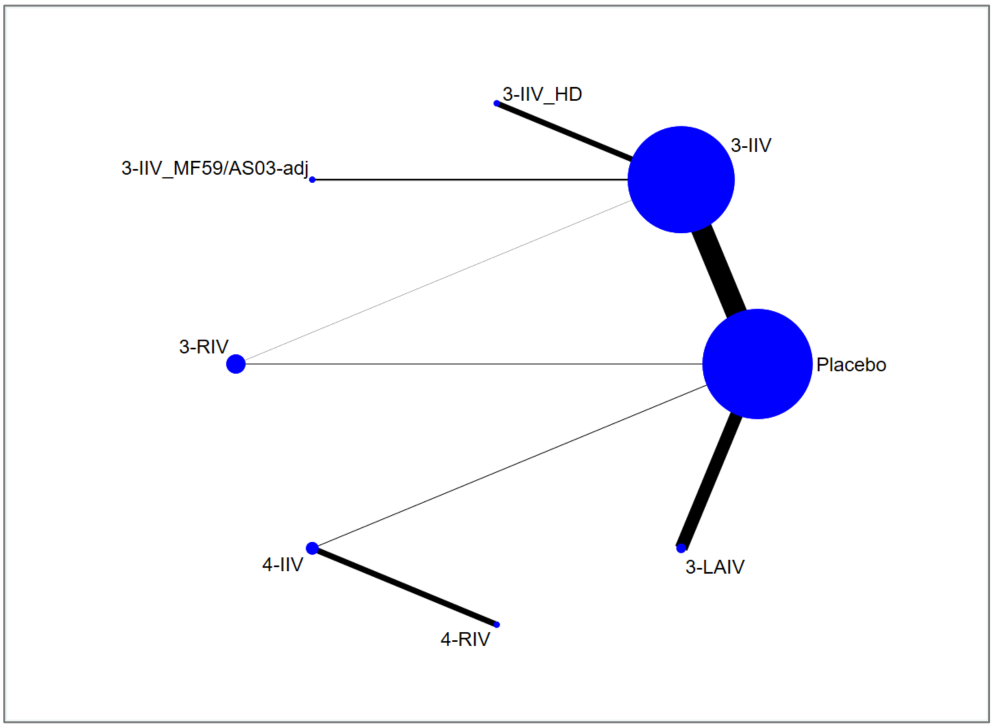

The thickness of the line is proportional to the precision of each direct estimate, and the width of each circle is proportional to the number of studies included in the treatment.

Adults and the elderly: Forest plot of ILI, comparison with placebo/no vaccine and with 3-IIV[*]

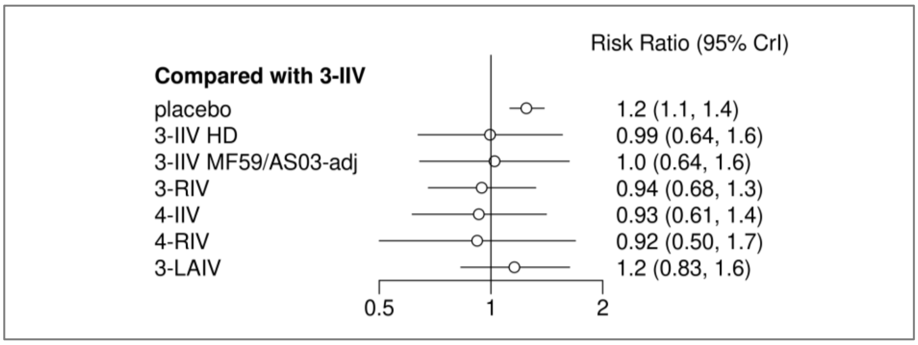

OR <1 favors experimental vaccine compared to placebo or 3-IIV

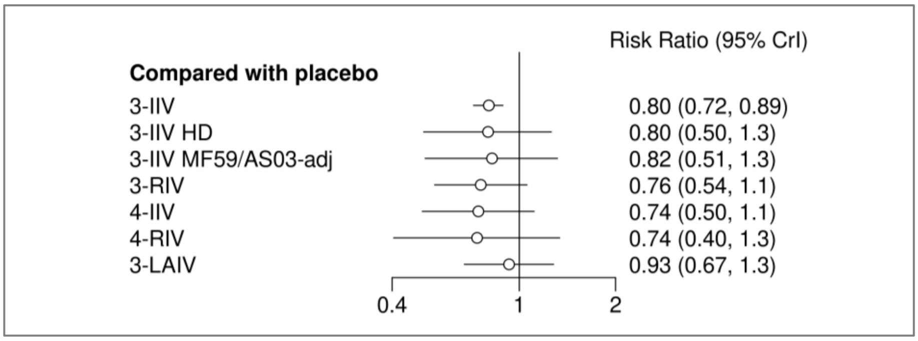

OR <1 favors experimental vaccine compared to placebo or 3-IIV

[*] **Placebo:** placebo/no vaccine; **3-IIV:** trivalent inactivated intramuscular; **3-IIV HD:** trivalent inactivated high dose intramuscular; **3-IIV ID:** trivalent inactivated intradermal; **3-IIV MF59/AS03-adj:** trivalent inactivated adjuvanted with MF59/ASO3 intramuscular: **3-IIV vir/lip-adj:** trivalent inactivated adjuvanted with Virosome/liposome intramuscular: **3-RIV:** trivalent recombinant intramuscular; **4-IIV:** quadrivalent inactivated intramuscular; **4-IIV HD:** quadrivalent inactivated high dose intramuscular: **4-IIV ID:** quadrivalent inactivated intradermal; **4-IIV MF59/AS03-adj:** quadrivalent inactivated adjuvanted with MF59/ASO3 intramuscular; **4-IIV vir/lip-adj:** quadrivalent inactivated adjuvanted with virosome/liposome intramuscular; **4-RIV:** quadrivalent recombinant intramuscular; **3-LAIV:** trivalent live attenuated intranasal; **4-LAIV:** quadrivalent live attenuated intranasal.

Adults and the elderly: Netleague analysis of ILI*

**placebo**	**0.8 (0.72, 0.89)**	0.8 (0.5, 1.26)	0.82 (0.51, 1.32)	0.76 (0.54, 1.06)	0.74 (0.5, 1.11)	0.74 (0.4, 1.34)	0.93 (0.67, 1.28)
**1.25 (1.13, 1.39)**	**3-IIV**	0.99 (0.64, 1.56)	1.02 (0.64, 1.62)	0.94 (0.68, 1.32)	0.93 (0.61, 1.41)	0.92 (0.5, 1.69)	1.16 (0.83, 1.63)
1.25 (0.79, 1.99)	1.01 (0.64, 1.57)	**3-IIV HD**	1.03 (0.54, 1.95)	0.95 (0.54, 1.66)	0.93 (0.51, 1.72)	0.92 (0.43, 1.97)	1.16 (0.67, 2.04)
1.22 (0.76, 1.97)	0.98 (0.62, 1.56)	0.97 (0.51, 1.85)	**3-IIV MF59/AS03-adj**	0.92 (0.52, 1.64)	0.91 (0.48, 1.7)	0.9 (0.42, 1.94)	1.13 (0.64, 2.01)
1.32 (0.95, 1.84)	1.06 (0.76, 1.47)	1.05 (0.6, 1.84)	1.08 (0.61, 1.92)	**3-RIV**	0.98 (0.58, 1.65)	0.97 (0.49, 1.93)	1.23 (0.77, 1.94)
1.34 (0.9, 2.01)	1.08 (0.71, 1.63)	1.07 (0.58, 1.97)	1.1 (0.59, 2.06)	1.02 (0.6, 1.72)	**4-IIV**	0.99 (0.64, 1.55)	1.25 (0.75, 2.09)
1.36 (0.75, 2.47)	1.09 (0.59, 2)	1.08 (0.51, 2.31)	1.11 (0.51, 2.4)	1.03 (0.52, 2.05)	1.01 (0.65, 1.57)	**4-RIV**	1.26 (0.64, 2.5)
1.08 (0.78, 1.49)	0.86 (0.61, 1.21)	0.86 (0.49, 1.5)	0.88 (0.5, 1.56)	0.82 (0.51, 1.29)	0.8 (0.48, 1.34)	0.79 (0.4, 1.56)	**3 LAIV**

The estimate is located at the intersection of the column-defining vaccine and the row-defining vaccine. Data are RRs (95% CrI). Significant results are in bold. In the upper triangle, comparisons of treatments should be read from right to left. An RR below 1 favors the medication on the bottom right vs. the medication on the top left in the diagonal.

.

* **Placebo:** placebo/no vaccine; **3-IIV:** trivalent inactivated intramuscular; **3-IIV HD:** trivalent inactivated high dose intramuscular; **3-IIV ID:** trivalent inactivated intradermal; **3-IIV MF59/AS03-adj:** trivalent inactivated adjuvanted with MF59/ASO3 intramuscular: **3-IIV vir/lip-adj:** trivalent inactivated adjuvanted with Virosome/liposome intramuscular: **3-RIV:** trivalent recombinant intramuscular; **4-IIV:** quadrivalent inactivated intramuscular; **4-IIV HD:** quadrivalent inactivated high dose intramuscular: **4-IIV ID:** quadrivalent inactivated intradermal; **4-IIV MF59/AS03-adj:** quadrivalent inactivated adjuvanted with MF59/ASO3 intramuscular; **4-IIV vir/lip-adj:** quadrivalent inactivated adjuvanted with virosome/liposome intramuscular; **4-RIV:** quadrivalent recombinant intramuscular; **3-LAIV:** trivalent live attenuated intranasal; **4-LAIV:** quadrivalent live attenuated intranasal.

All children: Network geometry of ILI*

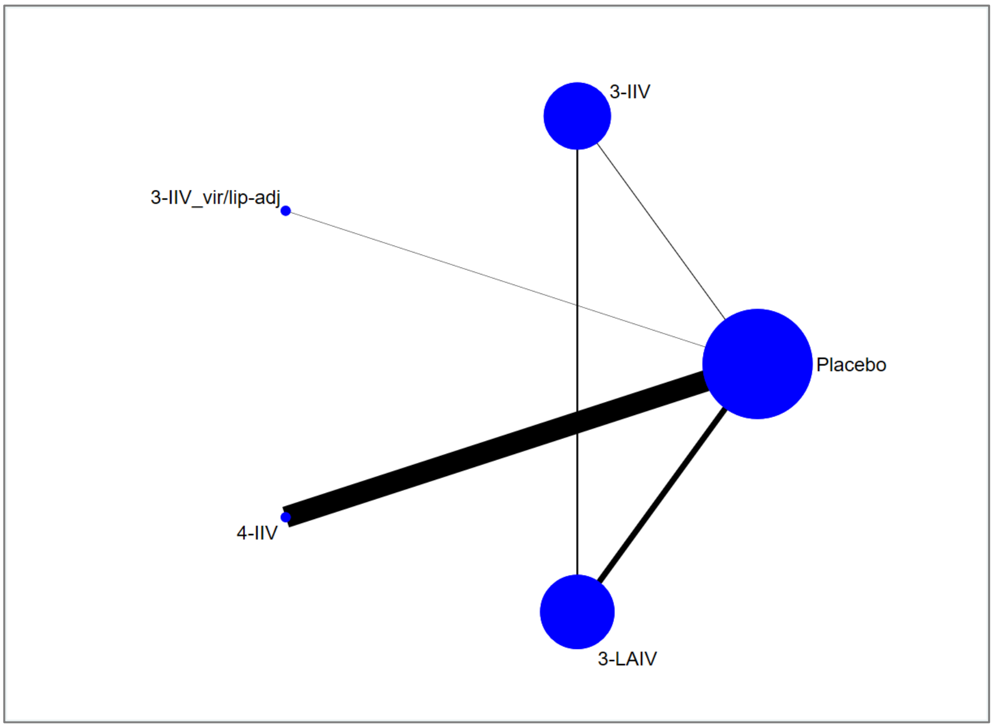

The thickness of the line is proportional to the precision of each direct estimate, and the width of each circle is proportional to the number of studies included in the treatment.

All children: Forest plots of ILI. Comparison with placebo/no vaccine and with 3-IIV*

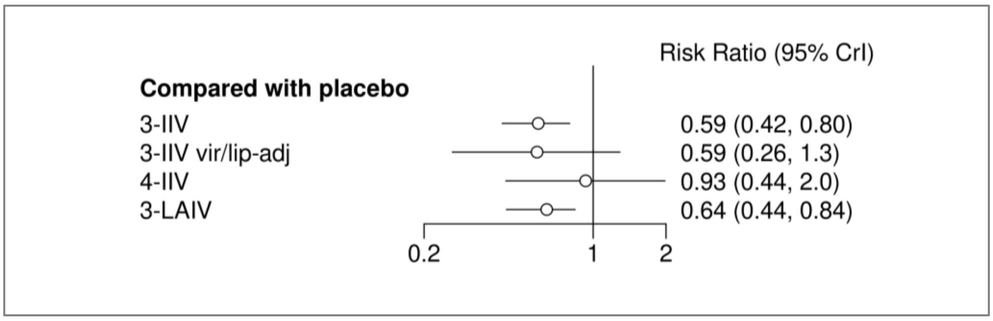

OR <1 favors experimental vaccine compared to placebo or 3-IIV

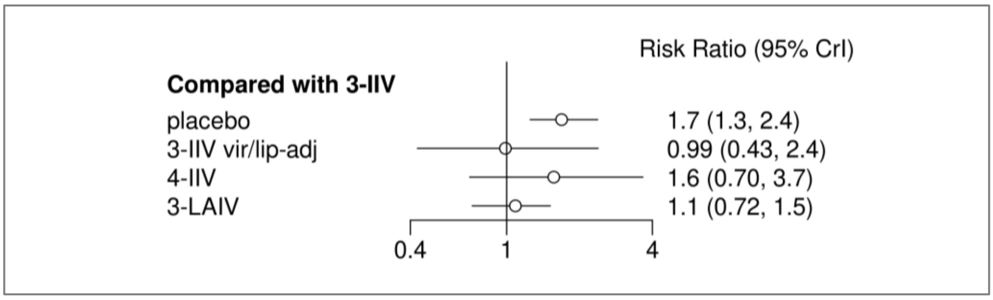

OR <1 favors experimental vaccine compared to placebo or 3-IIV

* **Placebo:** placebo/no vaccine; **3-IIV:** trivalent inactivated intramuscular; **3-IIV HD:** trivalent inactivated high dose intramuscular; **3-IIV ID:** trivalent inactivated intradermal; **3-IIV MF59/AS03-adj:** trivalent inactivated adjuvanted with MF59/ASO3 intramuscular: **3-IIV vir/lip-adj:** trivalent inactivated adjuvanted with Virosome/liposome intramuscular: **3-RIV:** trivalent recombinant intramuscular; **4-IIV:** quadrivalent inactivated intramuscular; **4-IIV HD:** quadrivalent inactivated high dose intramuscular: **4-IIV ID:** quadrivalent inactivated intradermal; **4-IIV MF59/AS03-adj:** quadrivalent inactivated adjuvanted with MF59/ASO3 intramuscular; **4-IIV vir/lip-adj:** quadrivalent inactivated adjuvanted with virosome/liposome intramuscular; **4-RIV:** quadrivalent recombinant intramuscular; **3-LAIV:** trivalent live attenuated intranasal; **4-LAIV:** quadrivalent live attenuated intranasal.

All children: Netleague analysis of ILI*

**placebo**	**0.59 (0.42, 0.8)**	0.59 (0.26, 1.29)	0.93 (0.44, 1.98)	**0.64 (0.44, 0.84)**
**1.69 (1.25, 2.38)**	**3-IIV**	0.99 (0.43, 2.39)	1.57 (0.7, 3.66)	1.09 (0.72, 1.51)
1.71 (0.78, 3.82)	1.01 (0.42, 2.34)	**3-IIV vir/lip-adj**	1.59 (0.53, 4.79)	1.1 (0.44, 2.45)
1.07 (0.5, 2.3)	0.64 (0.27, 1.42)	0.63 (0.21, 1.88)	**4-IIV**	0.69 (0.29, 1.47)
**1.56 (1.19, 2.28)**	0.92 (0.66, 1.39)	0.91 (0.41, 2.28)	1.44 (0.68, 3.5)	**3 LAIV**

The estimate is located at the intersection of the column-defining vaccine and the row-defining vaccine. Data are RRs (95% CrI). Significant results are in bold. In the upper triangle, comparisons of treatments should be read from right to left. An RR below 1 favors the medication on the bottom right vs. the medication on the top left in the diagonal.

* **Placebo:** placebo/no vaccine; **3-IIV:** trivalent inactivated intramuscular; **3-IIV HD:** trivalent inactivated high dose intramuscular; **3-IIV ID:** trivalent inactivated intradermal; **3-IIV MF59/AS03-adj:** trivalent inactivated adjuvanted with MF59/ASO3 intramuscular: **3-IIV vir/lip-adj:** trivalent inactivated adjuvanted with Virosome/liposome intramuscular: **3-RIV:** trivalent recombinant intramuscular; **4-IIV:** quadrivalent inactivated intramuscular; **4-IIV HD:** quadrivalent inactivated high dose intramuscular: **4-IIV ID:** quadrivalent inactivated intradermal; **4-IIV MF59/AS03-adj:** quadrivalent inactivated adjuvanted with MF59/ASO3 intramuscular; **4-IIV vir/lip-adj:** quadrivalent inactivated adjuvanted with virosome/liposome intramuscular; **4-RIV:** quadrivalent recombinant intramuscular; **3-LAIV:** trivalent live attenuated intranasal; **4-LAIV:** quadrivalent live attenuated intranasal.

All children: Network geometry of acute otitis media[*]

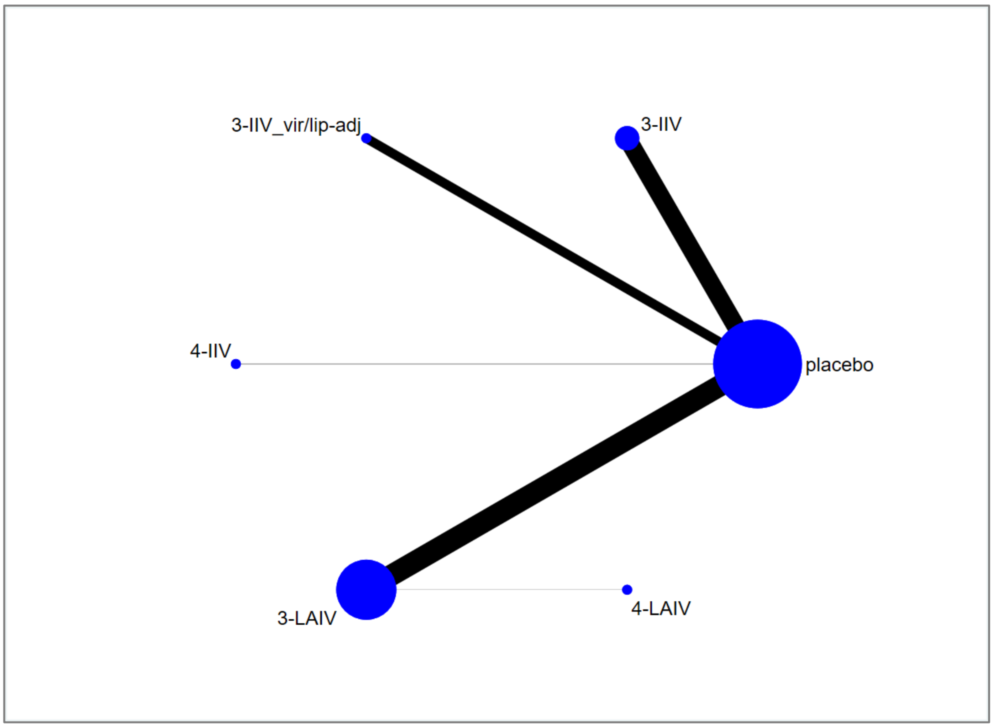

The thickness of the line is proportional to the precision of each direct estimate, and the width of each circle is proportional to the number of studies included in the treatment.

All children: Forest plot of acute otitis media, comparison with placebo/no vaccine and with 3-IIV[*]

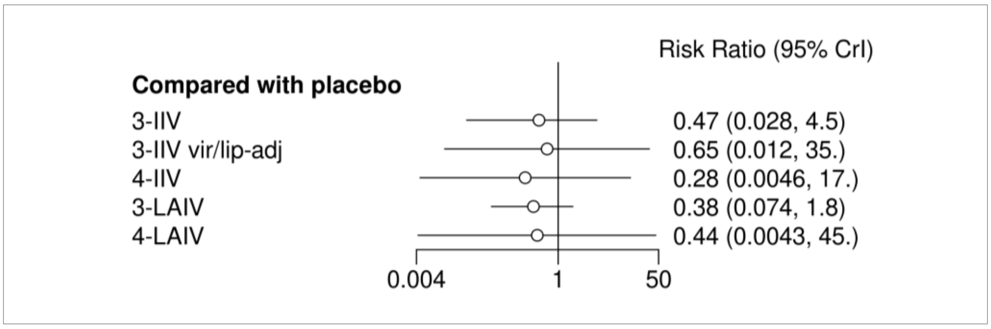

OR <1 favors experimental vaccine compared to placebo or 3-IIV

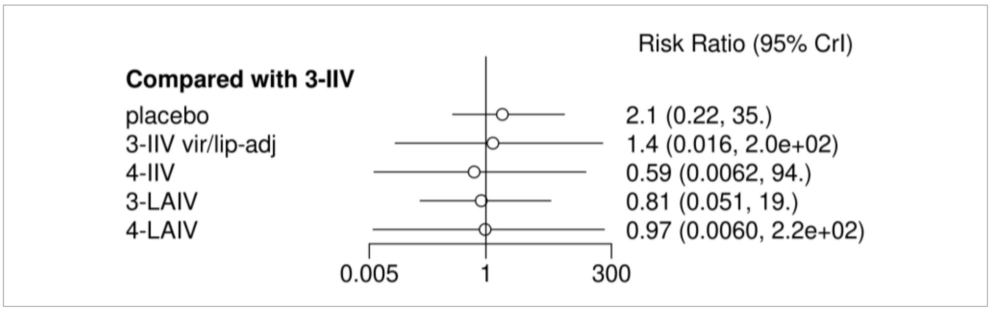

OR <1 favors experimental vaccine compared to placebo or 3-IIV

[*] **Placebo:** placebo/no vaccine; **3-IIV:** trivalent inactivated intramuscular; **3-IIV HD:** trivalent inactivated high dose intramuscular; **3-IIV ID:** trivalent inactivated intradermal; **3-IIV MF59/AS03-adj:** trivalent inactivated adjuvanted with MF59/ASO3 intramuscular: **3-IIV vir/lip-adj:** trivalent inactivated adjuvanted with Virosome/liposome intramuscular: **3-RIV:** trivalent recombinant intramuscular; **4-IIV:** quadrivalent inactivated intramuscular; **4-IIV HD:** quadrivalent inactivated high dose intramuscular: **4-IIV ID:** quadrivalent inactivated intradermal; **4-IIV MF59/AS03-adj:** quadrivalent inactivated adjuvanted with MF59/ASO3 intramuscular; **4-IIV vir/lip-adj:** quadrivalent inactivated adjuvanted with virosome/liposome intramuscular; **4-RIV:** quadrivalent recombinant intramuscular; **3-LAIV:** trivalent live attenuated intranasal; **4-LAIV:** quadrivalent live attenuated intranasal.

All children: Netleague analysis of acute otitis media*

**placebo**	0.47 (0.03, 4.51)	0.65 (0.01, 34.87)	0.28 (0, 16.7)	0.38 (0.07, 1.77)	0.44 (0, 44.83)
2.11 (0.22, 35.3)	**3-IIV**	1.37 (0.02, 201.44)	0.59 (0.01, 93.54)	0.81 (0.05, 19.04)	0.97 (0.01, 216.91)
1.54 (0.03, 83.13)	0.73 (0, 61.45)	**3-IIV vir/lip-adj**	0.43 (0, 130.05)	0.59 (0.01, 40.8)	0.69 (0, 288.72)
3.62 (0.06, 217.29)	1.71 (0.01, 161.07)	2.34 (0.01, 745.27)	**4-IIV**	1.38 (0.02, 105.68)	1.63 (0, 746.01)
2.62 (0.56, 13.44)	1.24 (0.05, 19.75)	1.7 (0.02, 131.34)	0.72 (0.01, 61.6)	**3 LAIV**	1.16 (0.02, 93.17)
2.26 (0.02, 234.81)	1.04 (0, 167.64)	1.46 (0, 698.03)	0.61 (0, 314.4)	0.86 (0.01, 63.6)	**4 LAIV**

The estimate is located at the intersection of the column-defining vaccine and the row-defining vaccine. Data are RRs (95% CrI). Significant results are in bold. In the upper triangle, comparisons of treatments should be read from right to left. An RR below 1 favors the medication on the bottom right vs. the medication on the top left in the diagonal.

* **Placebo:** placebo/no vaccine; **3-IIV:** trivalent inactivated intramuscular; **3-IIV HD:** trivalent inactivated high dose intramuscular; **3-IIV ID:** trivalent inactivated intradermal; **3-IIV MF59/AS03-adj:** trivalent inactivated adjuvanted with MF59/ASO3 intramuscular: **3-IIV vir/lip-adj:** trivalent inactivated adjuvanted with Virosome/liposome intramuscular: **3-RIV:** trivalent recombinant intramuscular; **4-IIV:** quadrivalent inactivated intramuscular; **4-IIV HD:** quadrivalent inactivated high dose intramuscular: **4-IIV ID:** quadrivalent inactivated intradermal; **4-IIV MF59/AS03-adj:** quadrivalent inactivated adjuvanted with MF59/ASO3 intramuscular; **4-IIV vir/lip-adj:** quadrivalent inactivated adjuvanted with virosome/liposome intramuscular; **4-RIV:** quadrivalent recombinant intramuscular; **3-LAIV:** trivalent live attenuated intranasal; **4-LAIV:** quadrivalent live attenuated intranasal.

Pregnancy outcomes: 3-IIV vs placebo/no vaccine, pairwise meta-analyses[*]

Abortion

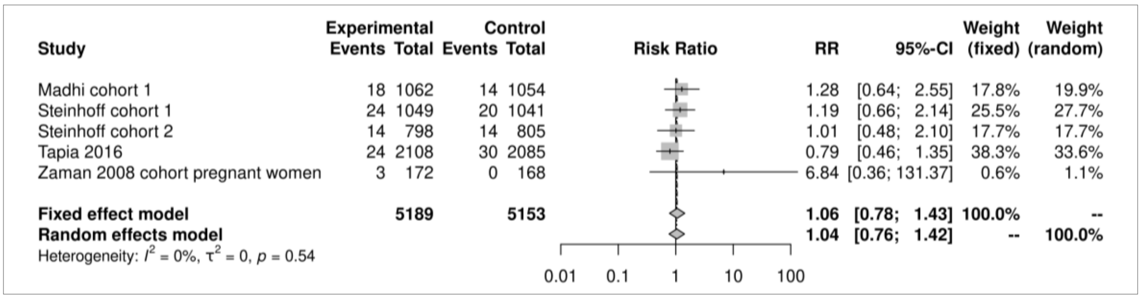

Preterm birth

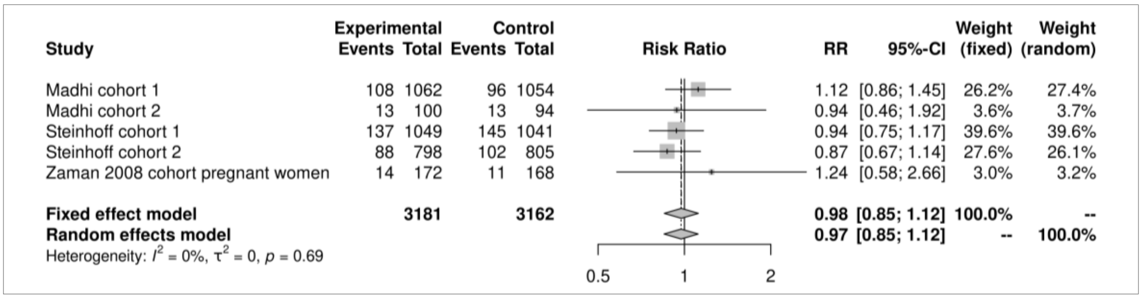

Congenital malformation

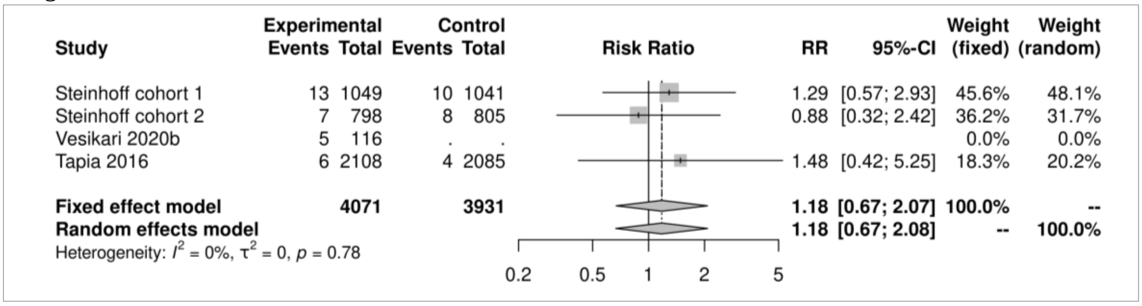

Neonatal death

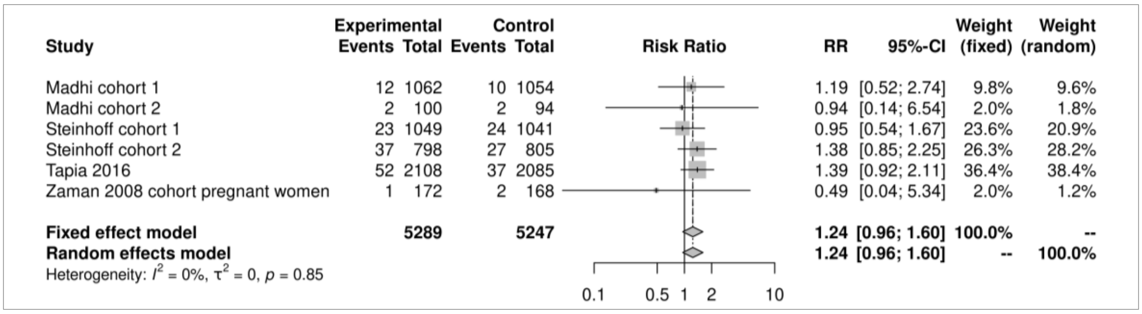

Low birth weight

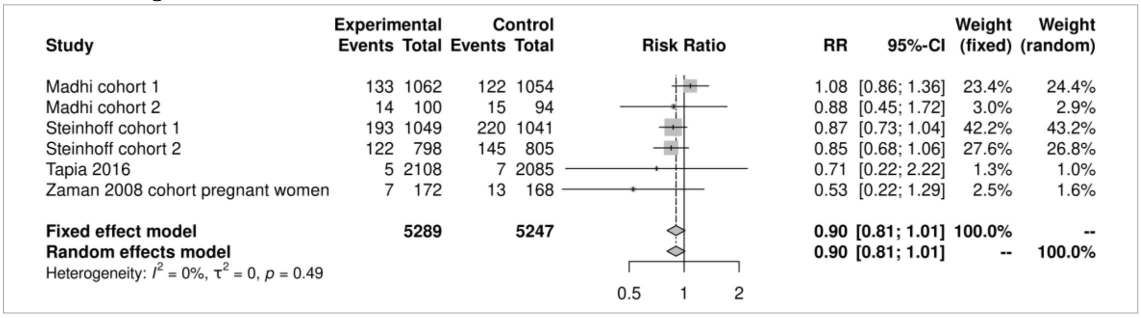

[*] RR <1 favors 3-IIV compared to placebo or no vaccine

Adults and the elderly: Network geometry of any systemic adverse event*

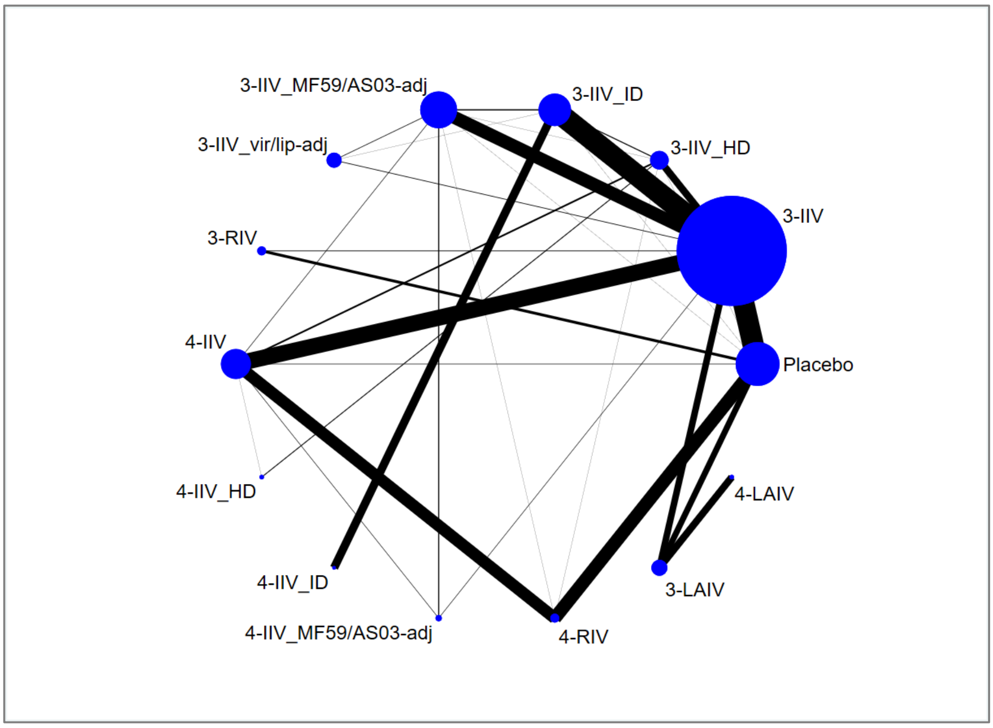

The thickness of the line is proportional to the precision of each direct estimate, and the width of each circle is proportional to the number of studies included in the treatment.

* **Placebo:** placebo/no vaccine; **3-IIV:** trivalent inactivated intramuscular; **3-IIV HD:** trivalent inactivated high dose intramuscular; **3-IIV ID:** trivalent inactivated intradermal; **3-IIV MF59/AS03-adj:** trivalent inactivated adjuvanted with MF59/ASO3 intramuscular: **3-IIV vir/lip-adj:** trivalent inactivated adjuvanted with Virosome/liposome intramuscular: **3-RIV:** trivalent recombinant intramuscular; **4-IIV:** quadrivalent inactivated intramuscular; **4-IIV HD:** quadrivalent inactivated high dose intramuscular: **4-IIV ID:** quadrivalent inactivated intradermal; **4-IIV MF59/AS03-adj:** quadrivalent inactivated adjuvanted with MF59/ASO3 intramuscular; **4-IIV vir/lip-adj:** quadrivalent inactivated adjuvanted with virosome/liposome intramuscular; **4-RIV:** quadrivalent recombinant intramuscular; **3-LAIV:** trivalent live attenuated intranasal; **4-LAIV:** quadrivalent live attenuated intranasal.

Adults and the elderly: Forest plots of any systemic adverse event, comparison with placebo/no vaccine and with 3-IIV*

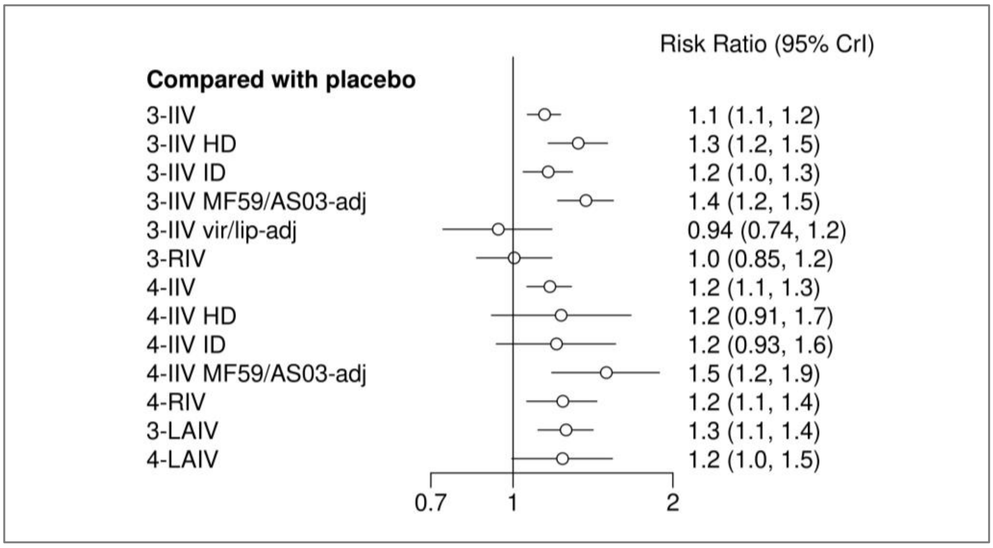

OR <1 favors experimental vaccine compared to placebo or 3-IIV

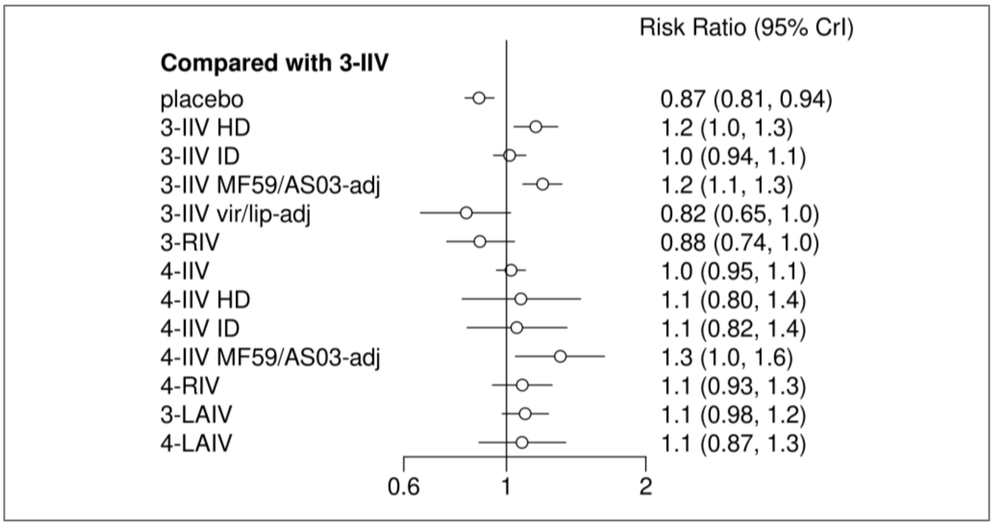

OR <1 favors experimental vaccine compared to placebo or 3-IIV

* **Placebo:** placebo/no vaccine; **3-IIV:** trivalent inactivated intramuscular; **3-IIV HD:** trivalent inactivated high dose intramuscular; **3-IIV ID:** trivalent inactivated intradermal; **3-IIV MF59/AS03-adj:** trivalent inactivated adjuvanted with MF59/ASO3 intramuscular: **3-IIV vir/lip-adj:** trivalent inactivated adjuvanted with Virosome/liposome intramuscular: **3-RIV:** trivalent recombinant intramuscular; **4-IIV:** quadrivalent inactivated intramuscular; **4-IIV HD:** quadrivalent inactivated high dose intramuscular: **4-IIV ID:** quadrivalent inactivated intradermal; **4-IIV MF59/AS03-adj:** quadrivalent inactivated adjuvanted with MF59/ASO3 intramuscular; **4-IIV vir/lip-adj:** quadrivalent inactivated adjuvanted with virosome/liposome intramuscular; **4-RIV:** quadrivalent recombinant intramuscular; **3-LAIV:** trivalent live attenuated intranasal; **4-LAIV:** quadrivalent live attenuated intranasal.

Adults and the elderly: Netleague analysis of any systemic adverse event*

**placebo**	**1.15 (1.06, 1.23)**	**1.33 (1.16, 1.51)**	**1.16 (1.05, 1.29)**	**1.37 (1.21, 1.54)**	0.94 (0.74, 1.18)	1 (0.85, 1.18)	**1.17 (1.06, 1.29)**	1.23 (0.91, 1.67)	1.21 (0.93, 1.56)	**1.5 (1.18, 1.89)**	**1.24 (1.06, 1.44)**	**1.26 (1.11, 1.42)**	1.24 (1, 1.54)
**0.87 (0.81, 0.94)**	**3-IIV**	**1.16 (1.04, 1.29)**	1.02 (0.94, 1.1)	**1.2 (1.09, 1.32)**	0.82 (0.65, 1.02)	0.88 (0.74, 1.04)	1.02 (0.95, 1.1)	1.07 (0.8, 1.45)	1.05 (0.82, 1.35)	**1.31 (1.05, 1.63)**	1.08 (0.93, 1.25)	1.1 (0.98, 1.23)	1.08 (0.87, 1.34)
**0.75 (0.66, 0.86)**	**0.86 (0.78, 0.96)**	**3-IIV HD**	0.88 (0.77, 1)	1.03 (0.89, 1.19)	**0.71 (0.55, 0.9)**	**0.76 (0.62, 0.93)**	0.88 (0.78, 1)	0.93 (0.7, 1.23)	0.91 (0.7, 1.19)	1.13 (0.88, 1.44)	0.93 (0.78, 1.12)	0.95 (0.81, 1.11)	0.93 (0.74, 1.19)
**0.86 (0.77, 0.96)**	0.98 (0.91, 1.07)	1.14 (1, 1.3)	**3-IIV ID**	**1.18 (1.04, 1.32)**	0.81 (0.64, 1.01)	0.86 (0.72, 1.04)	1.01 (0.9, 1.12)	1.06 (0.78, 1.43)	1.04 (0.82, 1.31)	**1.29 (1.02, 1.62)**	1.06 (0.9, 1.26)	1.08 (0.94, 1.24)	1.06 (0.85, 1.34)
**0.73 (0.65, 0.82)**	**0.84 (0.76, 0.92)**	0.97 (0.84, 1.12)	**0.85 (0.75, 0.96)**	**3-IIV MF59/AS03-adj**	**0.68 (0.54, 0.86)**	**0.73 (0.61, 0.89)**	**0.85 (0.76, 0.96)**	0.9 (0.66, 1.22)	0.88 (0.68, 1.15)	1.09 (0.88, 1.35)	0.9 (0.76, 1.08)	0.92 (0.79, 1.07)	0.9 (0.72, 1.15)
1.07 (0.85, 1.36)	1.22 (0.98, 1.53)	**1.41 (1.11, 1.81)**	1.24 (0.99, 1.57)	**1.46 (1.17, 1.84)**	**3-IIV vir/lip-adj**	1.07 (0.81, 1.43)	1.25 (0.99, 1.58)	1.31 (0.91, 1.91)	1.29 (0.93, 1.79)	**1.6 (1.17, 2.17)**	**1.32 (1.01, 1.73)**	**1.34 (1.04, 1.73)**	1.32 (0.97, 1.8)
1 (0.85, 1.17)	1.14 (0.96, 1.35)	**1.32 (1.08, 1.61)**	1.16 (0.96, 1.39)	**1.37 (1.12, 1.65)**	0.93 (0.7, 1.23)	**3-RIV**	1.17 (0.97, 1.39)	1.23 (0.87, 1.72)	1.2 (0.89, 1.62)	**1.49 (1.12, 1.97)**	1.24 (0.99, 1.53)	**1.25 (1.03, 1.52)**	1.23 (0.94, 1.61)
**0.85 (0.78, 0.94)**	0.98 (0.91, 1.05)	1.13 (1, 1.28)	0.99 (0.89, 1.11)	**1.17 (1.04, 1.32)**	0.8 (0.63, 1.01)	0.86 (0.72, 1.03)	**4-IIV**	1.05 (0.78, 1.42)	1.03 (0.8, 1.33)	**1.28 (1.02, 1.6)**	1.06 (0.92, 1.22)	1.07 (0.94, 1.23)	1.06 (0.85, 1.33)
0.81 (0.6, 1.1)	0.93 (0.69, 1.25)	1.08 (0.81, 1.42)	0.95 (0.7, 1.28)	1.11 (0.82, 1.52)	0.76 (0.52, 1.1)	0.82 (0.58, 1.15)	0.95 (0.71, 1.28)	**4-IIV-HD**	0.98 (0.67, 1.44)	1.22 (0.84, 1.75)	1.01 (0.73, 1.39)	1.02 (0.74, 1.4)	1.01 (0.7, 1.45)
0.83 (0.64, 1.08)	0.95 (0.74, 1.22)	1.1 (0.84, 1.43)	0.97 (0.76, 1.22)	1.14 (0.87, 1.48)	0.78 (0.56, 1.08)	0.83 (0.62, 1.13)	0.97 (0.75, 1.25)	1.02 (0.69, 1.5)	**4-IIV-ID**	1.24 (0.89, 1.73)	1.03 (0.77, 1.37)	1.04 (0.79, 1.37)	1.03 (0.74, 1.43)
**0.67 (0.53, 0.85)**	**0.76 (0.61, 0.96)**	0.89 (0.69, 1.13)	**0.78 (0.62, 0.98)**	0.92 (0.74, 1.14)	**0.63 (0.46, 0.85)**	**0.67 (0.51, 0.89)**	**0.78 (0.62, 0.98)**	0.82 (0.57, 1.19)	0.81 (0.58, 1.12)	**4-IIV MF59/AS03-adj**	0.83 (0.64, 1.08)	0.84 (0.66, 1.08)	0.83 (0.61, 1.13)
**0.81 (0.7, 0.94)**	0.92 (0.8, 1.07)	1.07 (0.89, 1.28)	0.94 (0.79, 1.11)	1.11 (0.93, 1.32)	**0.76 (0.58, 0.99)**	0.81 (0.65, 1.01)	0.95 (0.82, 1.09)	0.99 (0.72, 1.38)	0.97 (0.73, 1.3)	1.21 (0.93, 1.57)	**4-RIV**	1.01 (0.85, 1.22)	1 (0.77, 1.3)
**0.79 (0.71, 0.9)**	0.91 (0.81, 1.02)	1.05 (0.9, 1.23)	0.93 (0.8, 1.06)	1.09 (0.94, 1.26)	**0.75 (0.58, 0.96)**	**0.8 (0.66, 0.97)**	0.93 (0.81, 1.06)	0.98 (0.72, 1.34)	0.96 (0.73, 1.26)	1.19 (0.93, 1.53)	0.99 (0.82, 1.18)	**3-LAIV**	0.98 (0.82, 1.18)
0.81 (0.65, 1)	0.93 (0.75, 1.15)	1.07 (0.84, 1.36)	0.94 (0.75, 1.18)	1.11 (0.87, 1.4)	0.76 (0.55, 1.03)	0.81 (0.62, 1.06)	0.95 (0.75, 1.18)	0.99 (0.69, 1.43)	0.97 (0.7, 1.35)	1.21 (0.89, 1.64)	1 (0.77, 1.29)	1.02 (0.85, 1.22)	**4-LAIV**

The estimate is located at the intersection of the column-defining vaccine and the row-defining vaccine. Data are RRs (95% CrI). Significant results are in bold. In the upper triangle, comparisons of treatments should be read from right to left. An RR below 1 favors the medication on the bottom right vs. the medication on the top left in the diagonal.

* **Placebo:** placebo/no vaccine; **3-IIV:** trivalent inactivated intramuscular; **3-IIV HD:** trivalent inactivated high dose intramuscular; **3-IIV ID:** trivalent inactivated intradermal; **3-IIV MF59/AS03-adj:** trivalent inactivated adjuvanted with MF59/ASO3 intramuscular: **3-IIV vir/lip-adj:** trivalent inactivated adjuvanted with Virosome/liposome intramuscular: **3-RIV:** trivalent recombinant intramuscular; **4-IIV:** quadrivalent inactivated intramuscular; **4-IIV HD:** quadrivalent inactivated high dose intramuscular: **4-IIV ID:** quadrivalent inactivated intradermal; **4-IIV MF59/AS03-adj:** quadrivalent inactivated adjuvanted with MF59/ASO3 intramuscular; **4-IIV vir/lip-adj:** quadrivalent inactivated adjuvanted with virosome/liposome intramuscular; **4-RIV:** quadrivalent recombinant intramuscular; **3-LAIV:** trivalent live attenuated intranasal; **4-LAIV:** quadrivalent live attenuated intranasal.

The elderly, ≥61 years: Network geometry of any systemic adverse event *

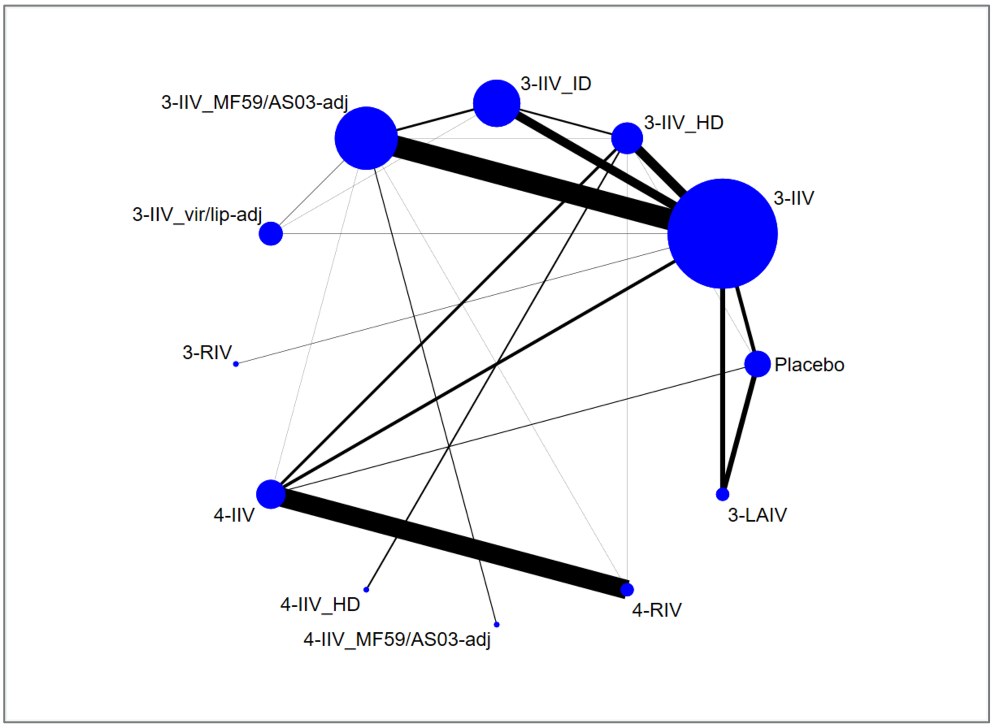

The thickness of the line is proportional to the precision of each direct estimate, and the width of each circle is proportional to the number of studies included in the treatment.

* **Placebo:** placebo/no vaccine; **3-IIV:** trivalent inactivated intramuscular; **3-IIV HD:** trivalent inactivated high dose intramuscular; **3-IIV ID:** trivalent inactivated intradermal; **3-IIV MF59/AS03-adj:** trivalent inactivated adjuvanted with MF59/ASO3 intramuscular: **3-IIV vir/lip-adj:** trivalent inactivated adjuvanted with Virosome/liposome intramuscular: **3-RIV:** trivalent recombinant intramuscular; **4-IIV:** quadrivalent inactivated intramuscular; **4-IIV HD:** quadrivalent inactivated high dose intramuscular: **4-IIV ID:** quadrivalent inactivated intradermal; **4-IIV MF59/AS03-adj:** quadrivalent inactivated adjuvanted with MF59/ASO3 intramuscular; **4-IIV vir/lip-adj:** quadrivalent inactivated adjuvanted with virosome/liposome intramuscular; **4-RIV:** quadrivalent recombinant intramuscular; **3-LAIV:** trivalent live attenuated intranasal; **4-LAIV:** quadrivalent live attenuated intranasal.

The elderly, ≥61 years: Forest plot of any systemic adverse event, comparison with placebo/no vaccine and with 3-IIV[*]

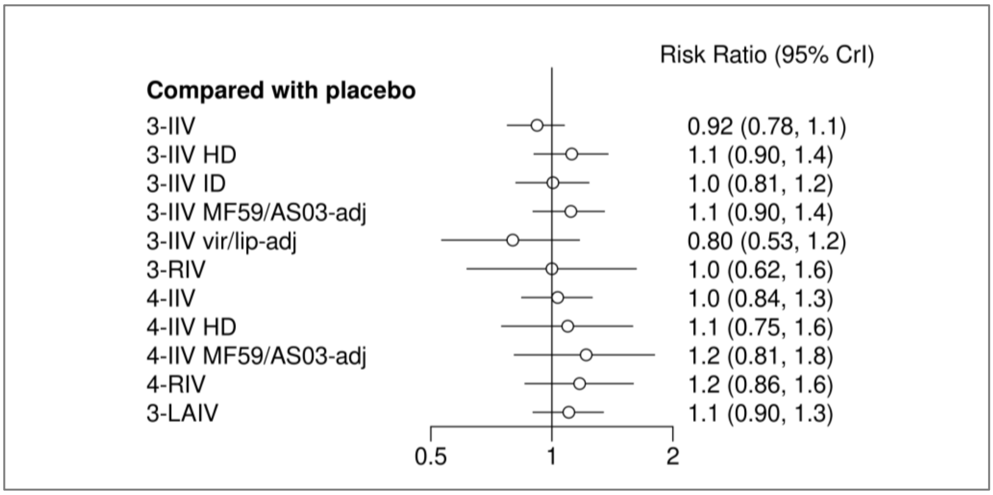

OR <1 favors experimental vaccine compared to placebo or 3-IIV

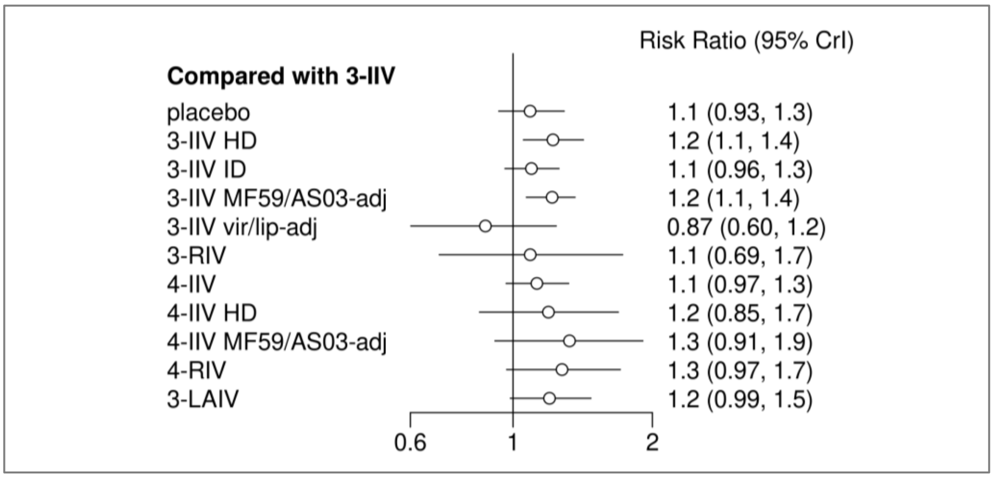

OR <1 favors experimental vaccine compared to placebo or 3-IIV

[*] **Placebo:** placebo/no vaccine; **3-IIV:** trivalent inactivated intramuscular; **3-IIV HD:** trivalent inactivated high dose intramuscular; **3-IIV ID:** trivalent inactivated intradermal; **3-IIV MF59/AS03-adj:** trivalent inactivated adjuvanted with MF59/ASO3 intramuscular: **3-IIV vir/lip-adj:** trivalent inactivated adjuvanted with Virosome/liposome intramuscular: **3-RIV:** trivalent recombinant intramuscular; **4-IIV:** quadrivalent inactivated intramuscular; **4-IIV HD:** quadrivalent inactivated high dose intramuscular: **4-IIV ID:** quadrivalent inactivated intradermal; **4-IIV MF59/AS03-adj:** quadrivalent inactivated adjuvanted with MF59/ASO3 intramuscular; **4-IIV vir/lip-adj:** quadrivalent inactivated adjuvanted with virosome/liposome intramuscular; **4-RIV:** quadrivalent recombinant intramuscular; **3-LAIV:** trivalent live attenuated intranasal; **4-LAIV:** quadrivalent live attenuated intranasal.

The elderly, ≥61 years: Netleague analysis of any systemic adverse event*

**placebo**	0.92 (0.78, 1.07)	1.12 (0.9, 1.38)	1.01 (0.81, 1.24)	1.12 (0.9, 1.35)	0.8 (0.53, 1.17)	1 (0.62, 1.62)	1.03 (0.84, 1.26)	1.1 (0.75, 1.59)	1.22 (0.81, 1.8)	1.17 (0.86, 1.59)	1.1 (0.9, 1.34)
1.09 (0.93, 1.29)	**3-IIV**	**1.22 (1.05, 1.42)**	1.1 (0.96, 1.26)	**1.21 (1.07, 1.36)**	0.87 (0.6, 1.24)	1.09 (0.69, 1.73)	1.13 (0.97, 1.32)	1.19 (0.85, 1.69)	1.32 (0.91, 1.91)	1.28 (0.97, 1.71)	1.2 (0.99, 1.47)
0.89 (0.73, 1.11)	**0.82 (0.7, 0.95)**	**3-IIV HD**	0.9 (0.75, 1.08)	1 (0.82, 1.19)	0.71 (0.48, 1.04)	0.89 (0.55, 1.45)	0.92 (0.77, 1.11)	0.98 (0.72, 1.33)	1.09 (0.73, 1.6)	1.05 (0.78, 1.42)	0.98 (0.77, 1.26)
0.99 (0.81, 1.23)	0.91 (0.8, 1.04)	1.11 (0.92, 1.34)	**3-IIV ID**	1.11 (0.93, 1.29)	0.8 (0.54, 1.14)	0.99 (0.62, 1.6)	1.03 (0.84, 1.25)	1.09 (0.76, 1.56)	1.21 (0.82, 1.76)	1.17 (0.86, 1.59)	1.09 (0.86, 1.4)
0.9 (0.74, 1.11)	**0.82 (0.73, 0.94)**	1 (0.84, 1.23)	0.9 (0.77, 1.07)	**3-IIV MF59/AS03-adj**	0.72 (0.5, 1.03)	0.9 (0.56, 1.45)	0.93 (0.77, 1.14)	0.98 (0.69, 1.43)	1.09 (0.77, 1.55)	1.05 (0.79, 1.45)	0.99 (0.79, 1.27)
1.25 (0.85, 1.88)	1.15 (0.81, 1.66)	1.4 (0.96, 2.09)	1.26 (0.88, 1.84)	1.39 (0.98, 2.01)	**3-IIV vir/lip-adj**	1.26 (0.7, 2.26)	1.29 (0.88, 1.93)	1.37 (0.84, 2.26)	1.53 (0.92, 2.53)	1.47 (0.94, 2.33)	1.38 (0.92, 2.1)
1 (0.62, 1.63)	0.92 (0.58, 1.44)	1.12 (0.69, 1.8)	1.01 (0.62, 1.61)	1.11 (0.69, 1.77)	0.8 (0.44, 1.42)	**3-RIV**	1.03 (0.64, 1.67)	1.09 (0.62, 1.92)	1.22 (0.67, 2.16)	1.17 (0.68, 2.01)	1.1 (0.67, 1.81)
0.97 (0.79, 1.19)	0.89 (0.76, 1.04)	1.08 (0.9, 1.3)	0.97 (0.8, 1.19)	1.08 (0.88, 1.3)	0.77 (0.52, 1.13)	0.97 (0.6, 1.57)	**4-IIV**	1.06 (0.74, 1.52)	1.18 (0.79, 1.74)	1.13 (0.9, 1.45)	1.07 (0.84, 1.36)
0.91 (0.63, 1.33)	0.84 (0.59, 1.18)	1.02 (0.75, 1.39)	0.92 (0.64, 1.32)	1.02 (0.7, 1.45)	0.73 (0.44, 1.19)	0.91 (0.52, 1.62)	0.94 (0.66, 1.35)	**4-IIV-HD**	1.11 (0.67, 1.82)	1.07 (0.7, 1.65)	1.01 (0.68, 1.5)
0.82 (0.56, 1.24)	0.76 (0.52, 1.09)	0.92 (0.62, 1.38)	0.83 (0.57, 1.22)	0.92 (0.65, 1.29)	0.66 (0.4, 1.09)	0.82 (0.46, 1.49)	0.85 (0.57, 1.27)	0.9 (0.55, 1.5)	**4-IIV MF59/AS03-adj**	0.96 (0.61, 1.54)	0.91 (0.6, 1.38)
0.85 (0.63, 1.17)	0.78 (0.59, 1.03)	0.96 (0.71, 1.28)	0.86 (0.63, 1.17)	0.95 (0.69, 1.27)	0.68 (0.43, 1.07)	0.85 (0.5, 1.46)	0.88 (0.69, 1.12)	0.93 (0.61, 1.43)	1.04 (0.65, 1.63)	**4-RIV**	0.94 (0.67, 1.32)
0.91 (0.74, 1.11)	0.83 (0.68, 1.01)	1.02 (0.79, 1.3)	0.91 (0.72, 1.16)	1.01 (0.79, 1.27)	0.73 (0.48, 1.09)	0.91 (0.55, 1.5)	0.94 (0.73, 1.19)	0.99 (0.67, 1.47)	1.1 (0.72, 1.66)	1.06 (0.76, 1.5)	**3-LAIV**

The estimate is located at the intersection of the column-defining vaccine and the row-defining vaccine. Data are RRs (95% CrI). Significant results are in bold. In the upper triangle, comparisons of treatments should be read from right to left. An RR below 1 favors the medication on the bottom right vs. the medication on the top left in the diagonal.

* **Placebo:** placebo/no vaccine; **3-IIV:** trivalent inactivated intramuscular; **3-IIV HD:** trivalent inactivated high dose intramuscular; **3-IIV ID:** trivalent inactivated intradermal; **3-IIV MF59/AS03-adj:** trivalent inactivated adjuvanted with MF59/ASO3 intramuscular: **3-IIV vir/lip-adj:** trivalent inactivated adjuvanted with Virosome/liposome intramuscular: **3-RIV:** trivalent recombinant intramuscular; **4-IIV:** quadrivalent inactivated intramuscular; **4-IIV HD:** quadrivalent inactivated high dose intramuscular: **4-IIV ID:** quadrivalent inactivated intradermal; **4-IIV MF59/AS03-adj:** quadrivalent inactivated adjuvanted with MF59/ASO3 intramuscular; **4-IIV vir/lip-adj:** quadrivalent inactivated adjuvanted with virosome/liposome intramuscular; **4-RIV:** quadrivalent recombinant intramuscular; **3-LAIV:** trivalent live attenuated intranasal; **4-LAIV:** quadrivalent live attenuated intranasal.

Adult and the elderly, immunocompromised-cancer participants: Network geometry of any systemic adverse event*

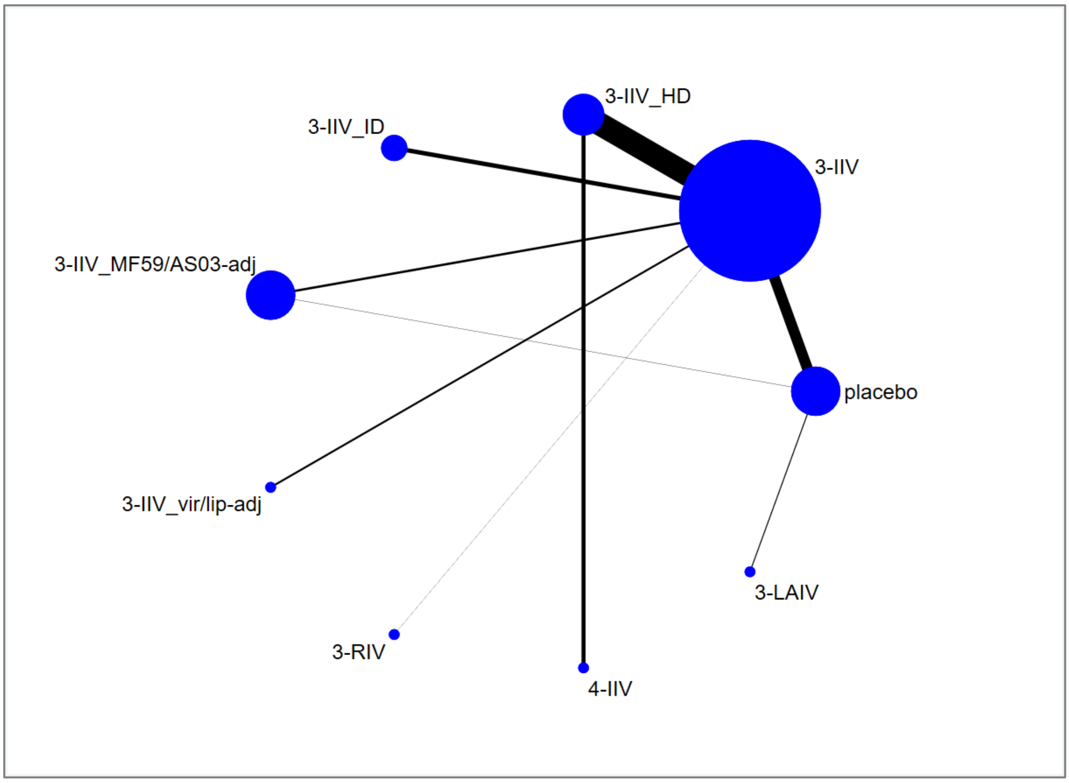

The thickness of the line is proportional to the precision of each direct estimate, and the width of each circle is proportional to the number of studies included in the treatment.

* **Placebo:** placebo/no vaccine; **3-IIV:** trivalent inactivated intramuscular; **3-IIV HD:** trivalent inactivated high dose intramuscular; **3-IIV ID:** trivalent inactivated intradermal; **3-IIV MF59/AS03-adj:** trivalent inactivated adjuvanted with MF59/ASO3 intramuscular: **3-IIV vir/lip-adj:** trivalent inactivated adjuvanted with Virosome/liposome intramuscular: **3-RIV:** trivalent recombinant intramuscular; **4-IIV:** quadrivalent inactivated intramuscular; **4-IIV HD:** quadrivalent inactivated high dose intramuscular: **4-IIV ID:** quadrivalent inactivated intradermal; **4-IIV MF59/AS03-adj:** quadrivalent inactivated adjuvanted with MF59/ASO3 intramuscular; **4-IIV vir/lip-adj:** quadrivalent inactivated adjuvanted with virosome/liposome intramuscular; **4-RIV:** quadrivalent recombinant intramuscular; **3-LAIV:** trivalent live attenuated intranasal; **4-LAIV:** quadrivalent live attenuated intranasal.

Adult and the elderly, immunocompromised-cancer participants: Forest plots of any systemic adverse event, comparison with placebo/no vaccine and with 3-IIV[*]

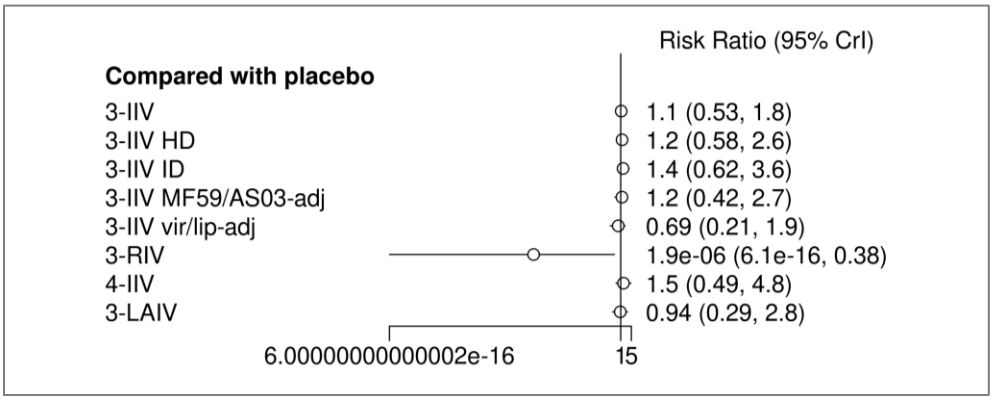

OR <1 favors experimental vaccine compared to placebo or 3-IIV

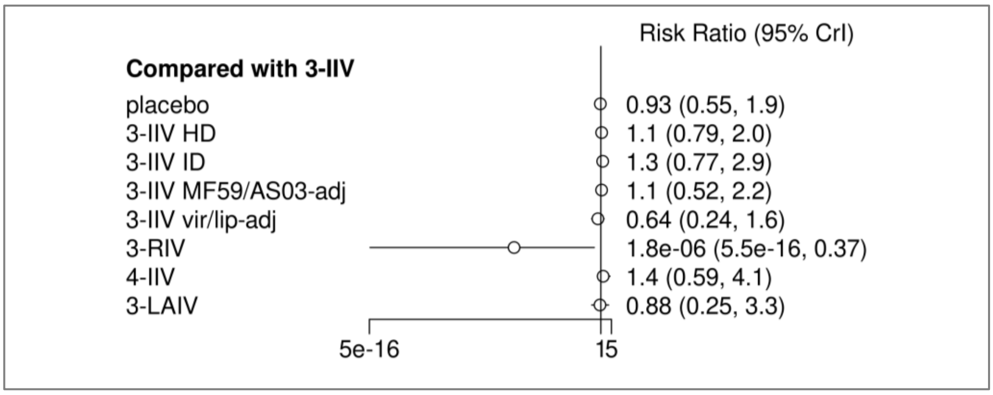

OR <1 favors experimental vaccine compared to placebo or 3-IIV

[*] **Placebo:** placebo/no vaccine; **3-IIV:** trivalent inactivated intramuscular; **3-IIV HD:** trivalent inactivated high dose intramuscular; **3-IIV ID:** trivalent inactivated intradermal; **3-IIV MF59/AS03-adj:** trivalent inactivated adjuvanted with MF59/ASO3 intramuscular: **3-IIV vir/lip-adj:** trivalent inactivated adjuvanted with Virosome/liposome intramuscular: **3-RIV:** trivalent recombinant intramuscular; **4-IIV:** quadrivalent inactivated intramuscular; **4-IIV HD:** quadrivalent inactivated high dose intramuscular: **4-IIV ID:** quadrivalent inactivated intradermal; **4-IIV MF59/AS03-adj:** quadrivalent inactivated adjuvanted with MF59/ASO3 intramuscular; **4-IIV vir/lip-adj:** quadrivalent inactivated adjuvanted with virosome/liposome intramuscular; **4-RIV:** quadrivalent recombinant intramuscular; **3-LAIV:** trivalent live attenuated intranasal; **4-LAIV:** quadrivalent live attenuated intranasal.

Adult and rhe elderly, immunocompromised-cancer participants: Netleague analysis of any systemic adverse event*

**placebo**	1.08 (0.53, 1.83)	1.22 (0.58, 2.63)	1.42 (0.62, 3.62)	1.2 (0.42, 2.72)	0.69 (0.21, 1.95)	**0 (0, 0.38)**	1.52 (0.49, 4.76)	0.94 (0.29, 2.83)
0.93 (0.55, 1.89)	**3-IIV**	1.13 (0.79, 1.98)	1.33 (0.77, 2.91)	1.12 (0.52, 2.24)	0.64 (0.24, 1.65)	**0 (0, 0.37)**	1.42 (0.59, 4.07)	0.88 (0.25, 3.29)
0.82 (0.38, 1.74)	0.88 (0.51, 1.26)	**3-IIV-HD**	1.16 (0.56, 2.68)	0.98 (0.36, 2.12)	0.56 (0.18, 1.47)	**0 (0, 0.31)**	1.24 (0.53, 2.9)	0.77 (0.19, 2.88)
0.7 (0.28, 1.61)	0.75 (0.34, 1.3)	0.86 (0.37, 1.8)	**3-IIV-ID**	0.84 (0.27, 2.03)	0.48 (0.13, 1.36)	**0 (0, 0.27)**	1.07 (0.33, 3.21)	0.66 (0.15, 2.58)
0.83 (0.37, 2.4)	0.89 (0.45, 1.92)	1.02 (0.47, 2.75)	1.19 (0.49, 3.77)	**3-IIV MF59/AS03-adj**	0.57 (0.18, 1.97)	**0 (0, 0.35)**	1.26 (0.43, 4.9)	0.78 (0.2, 3.65)
1.46 (0.51, 4.86)	1.56 (0.61, 4.09)	1.79 (0.68, 5.52)	2.09 (0.74, 7.5)	1.77 (0.51, 5.59)	**3-IIV vir/lip-adj**	**0 (0, 0.62)**	2.22 (0.63, 9.28)	1.37 (0.29, 7.08)
**540422.99 (2.6, 1641730164398524)**	**543516.4 (2.7, 1808085701935791)**	**640275.63 (3.2, 2125998396124094)**	**734852.91 (3.7, 2394122047876191)**	**657357.39 (2.88, 2209682118404833)**	**335286.59 (1.62, 1150286171477600)**	**3-RIV**	**808442.48 (3.79, 2574872698940468)**	**483429.27 (2.2, 1466279156913650)**
0.66 (0.21, 2.03)	0.71 (0.25, 1.7)	0.8 (0.35, 1.88)	0.93 (0.31, 3.07)	0.79 (0.2, 2.33)	0.45 (0.11, 1.58)	**0 (0, 0.26)**	**4-IIV**	0.62 (0.12, 2.92)
1.07 (0.35, 3.45)	1.14 (0.3, 4.02)	1.3 (0.35, 5.18)	1.52 (0.39, 6.73)	1.28 (0.27, 5.04)	0.73 (0.14, 3.44)	**0 (0, 0.46)**	1.62 (0.34, 8.21)	**3-LAIV**

The estimate is located at the intersection of the column-defining vaccine and the row-defining vaccine. Data are RRs (95% CrI). Significant results are in bold. In the upper triangle, comparisons of treatments should be read from right to left. An RR below 1 favors the medication on the bottom right vs. the medication on the top left in the diagonal.

* **Placebo:** placebo/no vaccine; **3-IIV:** trivalent inactivated intramuscular; **3-IIV HD:** trivalent inactivated high dose intramuscular; **3-IIV ID:** trivalent inactivated intradermal; **3-IIV MF59/AS03-adj:** trivalent inactivated adjuvanted with MF59/ASO3 intramuscular: **3-IIV vir/lip-adj:** trivalent inactivated adjuvanted with Virosome/liposome intramuscular: **3-RIV:** trivalent recombinant intramuscular; **4-IIV:** quadrivalent inactivated intramuscular; **4-IIV HD:** quadrivalent inactivated high dose intramuscular: **4-IIV ID:** quadrivalent inactivated intradermal; **4-IIV MF59/AS03-adj:** quadrivalent inactivated adjuvanted with MF59/ASO3 intramuscular; **4-IIV vir/lip-adj:** quadrivalent inactivated adjuvanted with virosome/liposome intramuscular; **4-RIV:** quadrivalent recombinant intramuscular; **3-LAIV:** trivalent live attenuated intranasal; **4-LAIV:** quadrivalent live attenuated intranasal.

All children: Network geometry of any systemic adverse event*

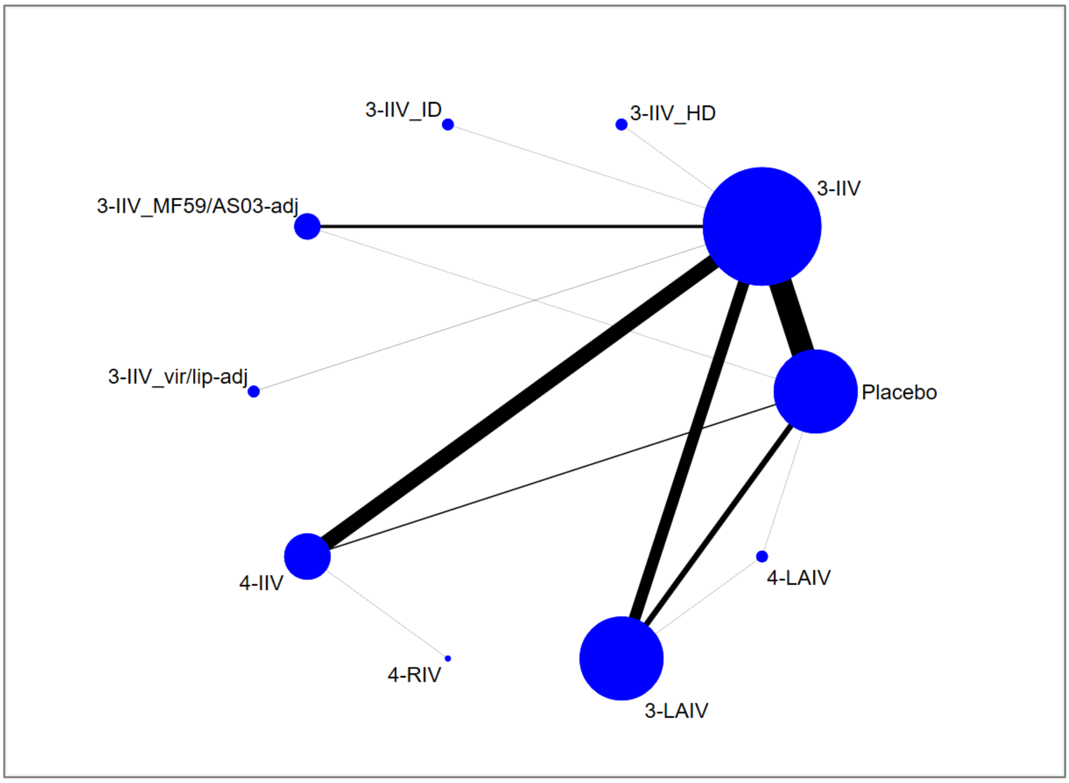

The thickness of the line is proportional to the precision of each direct estimate, and the width of each circle is proportional to the number of studies included in the treatment.

* **Placebo:** placebo/no vaccine; **3-IIV:** trivalent inactivated intramuscular; **3-IIV HD:** trivalent inactivated high dose intramuscular; **3-IIV ID:** trivalent inactivated intradermal; **3-IIV MF59/AS03-adj:** trivalent inactivated adjuvanted with MF59/ASO3 intramuscular: **3-IIV vir/lip-adj:** trivalent inactivated adjuvanted with Virosome/liposome intramuscular: **3-RIV:** trivalent recombinant intramuscular; **4-IIV:** quadrivalent inactivated intramuscular; **4-IIV HD:** quadrivalent inactivated high dose intramuscular: **4-IIV ID:** quadrivalent inactivated intradermal; **4-IIV MF59/AS03-adj:** quadrivalent inactivated adjuvanted with MF59/ASO3 intramuscular; **4-IIV vir/lip-adj:** quadrivalent inactivated adjuvanted with virosome/liposome intramuscular; **4-RIV:** quadrivalent recombinant intramuscular; **3-LAIV:** trivalent live attenuated intranasal; **4-LAIV:** quadrivalent live attenuated intranasal.

All children: Forest plots of any systemic adverse event, comparison with placebo/no vaccine and with 3-IIV*

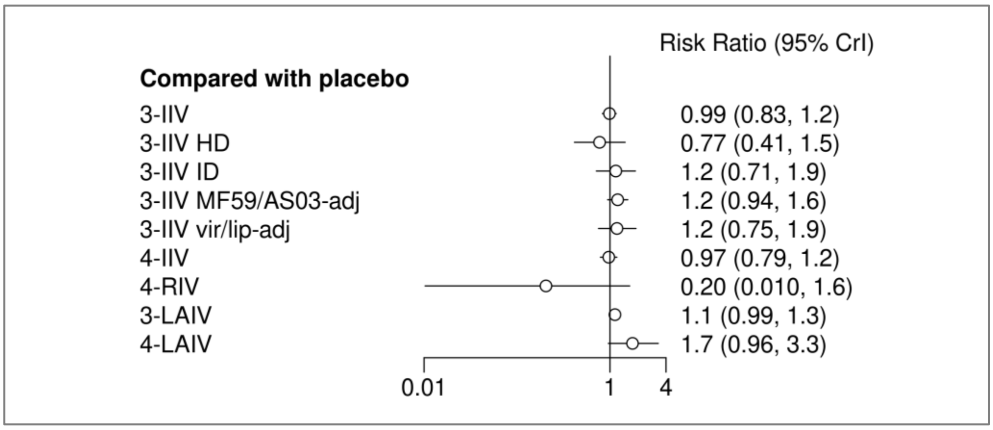

OR <1 favors experimental vaccine compared to placebo or 3-IIV

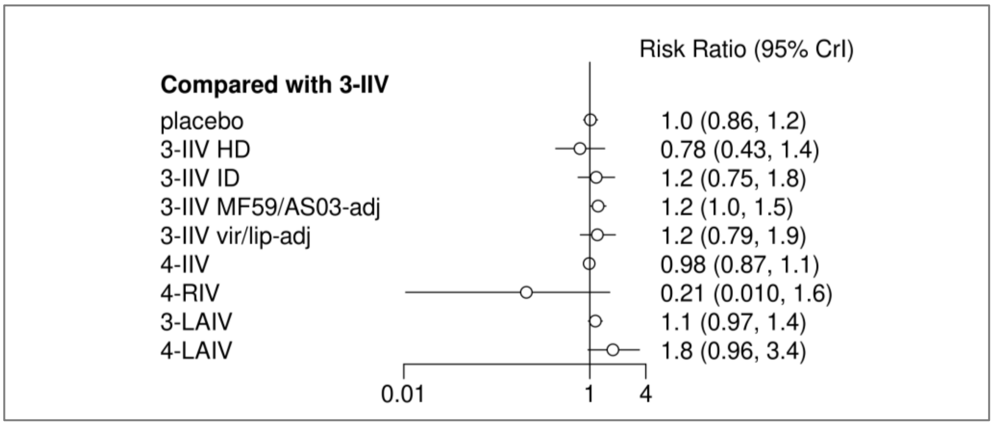

OR <1 favors experimental vaccine compared to placebo or 3-IIV

* **Placebo:** placebo/no vaccine; **3-IIV:** trivalent inactivated intramuscular; **3-IIV HD:** trivalent inactivated high dose intramuscular; **3-IIV ID:** trivalent inactivated intradermal; **3-IIV MF59/AS03-adj:** trivalent inactivated adjuvanted with MF59/ASO3 intramuscular: **3-IIV vir/lip-adj:** trivalent inactivated adjuvanted with Virosome/liposome intramuscular: **3-RIV:** trivalent recombinant intramuscular; **4-IIV:** quadrivalent inactivated intramuscular; **4-IIV HD:** quadrivalent inactivated high dose intramuscular: **4-IIV ID:** quadrivalent inactivated intradermal; **4-IIV MF59/AS03-adj:** quadrivalent inactivated adjuvanted with MF59/ASO3 intramuscular; **4-IIV vir/lip-adj:** quadrivalent inactivated adjuvanted with virosome/liposome intramuscular; **4-RIV:** quadrivalent recombinant intramuscular; **3-LAIV:** trivalent live attenuated intranasal; **4-LAIV:** quadrivalent live attenuated intranasal.

All children: Netleague analysis of any systemic adverse event[*]

**placebo**	0.99 (0.83, 1.16)	0.77 (0.41, 1.45)	1.15 (0.71, 1.87)	1.21 (0.94, 1.55)	1.19 (0.75, 1.9)	0.97 (0.79, 1.18)	0.2 (0.01, 1.63)	1.13 (0.99, 1.28)	1.75 (0.96, 3.3)
1.01 (0.86, 1.21)	**3-IIV**	0.78 (0.43, 1.44)	1.17 (0.75, 1.85)	**1.23 (1.02, 1.49)**	1.21 (0.79, 1.88)	0.98 (0.87, 1.12)	0.21 (0.01, 1.64)	1.15 (0.97, 1.36)	1.77 (0.96, 3.41)
1.3 (0.69, 2.41)	1.28 (0.69, 2.32)	**3-IIV HD**	1.5 (0.7, 3.19)	1.57 (0.83, 2.94)	1.54 (0.73, 3.24)	1.26 (0.67, 2.31)	0.26 (0.01, 2.28)	1.46 (0.77, 2.72)	2.27 (0.95, 5.45)
0.87 (0.53, 1.4)	0.85 (0.54, 1.33)	0.67 (0.31, 1.42)	**3-IIV ID**	1.05 (0.64, 1.71)	1.03 (0.55, 1.94)	0.84 (0.52, 1.34)	0.18 (0.01, 1.48)	0.98 (0.6, 1.58)	1.51 (0.7, 3.35)
0.83 (0.64, 1.06)	**0.82 (0.67, 0.98)**	0.64 (0.34, 1.2)	0.95 (0.59, 1.56)	**3-IIV MF59/AS03-adj**	0.98 (0.62, 1.58)	0.8 (0.64, 1)	0.17 (0.01, 1.34)	0.93 (0.72, 1.2)	1.44 (0.76, 2.84)
0.84 (0.53, 1.33)	0.83 (0.53, 1.26)	0.65 (0.31, 1.37)	0.97 (0.52, 1.81)	1.02 (0.63, 1.62)	**3-IIV vir/lip-adj**	0.82 (0.52, 1.26)	0.17 (0.01, 1.41)	0.95 (0.59, 1.5)	1.47 (0.69, 3.2)
1.03 (0.84, 1.27)	1.02 (0.9, 1.15)	0.8 (0.43, 1.49)	1.19 (0.75, 1.91)	1.25 (1, 1.57)	1.22 (0.79, 1.94)	**4-IIV**	0.21 (0.01, 1.66)	1.17 (0.95, 1.43)	1.8 (0.96, 3.5)
4.89 (0.61, 97.48)	4.81 (0.61, 95.31)	3.8 (0.44, 76.63)	5.66 (0.68, 114.54)	5.91 (0.75, 116.85)	5.82 (0.71, 114.47)	4.73 (0.6, 93.43)	**4-RIV**	5.52 (0.7, 109.88)	**8.6 (1.01, 184.89)**
0.88 (0.78, 1.01)	0.87 (0.74, 1.03)	0.68 (0.37, 1.29)	1.02 (0.63, 1.67)	1.07 (0.84, 1.38)	1.05 (0.67, 1.69)	0.86 (0.7, 1.05)	0.18 (0.01, 1.44)	**3-LAIV**	1.55 (0.85, 2.93)
0.57 (0.3, 1.04)	0.56 (0.29, 1.04)	0.44 (0.18, 1.05)	0.66 (0.3, 1.42)	0.69 (0.35, 1.32)	0.68 (0.31, 1.45)	0.56 (0.29, 1.04)	**0.12 (0.01, 0.99)**	0.65 (0.34, 1.18)	**4-LAIV**

The estimate is located at the intersection of the column-defining vaccine and the row-defining vaccine. Data are RRs (95% CrI). Significant results are in bold. In the upper triangle, comparisons of treatments should be read from right to left. An RR below 1 favors the medication on the bottom right vs. the medication on the top left in the diagonal.

[*] **Placebo:** placebo/no vaccine; **3-IIV:** trivalent inactivated intramuscular; **3-IIV HD:** trivalent inactivated high dose intramuscular; **3-IIV ID:** trivalent inactivated intradermal; **3-IIV MF59/AS03-adj:** trivalent inactivated adjuvanted with MF59/ASO3 intramuscular: **3-IIV vir/lip-adj:** trivalent inactivated adjuvanted with Virosome/liposome intramuscular: **3-RIV:** trivalent recombinant intramuscular; **4-IIV:** quadrivalent inactivated intramuscular; **4-IIV HD:** quadrivalent inactivated high dose intramuscular: **4-IIV ID:** quadrivalent inactivated intradermal; **4-IIV MF59/AS03-adj:** quadrivalent inactivated adjuvanted with MF59/ASO3 intramuscular; **4-IIV vir/lip-adj:** quadrivalent inactivated adjuvanted with virosome/liposome intramuscular; **4-RIV:** quadrivalent recombinant intramuscular; **3-LAIV:** trivalent live attenuated intranasal; **4-LAIV:** quadrivalent live attenuated intranasal.

Children, aged ≤5 years: Network geometry of any systemic adverse event*

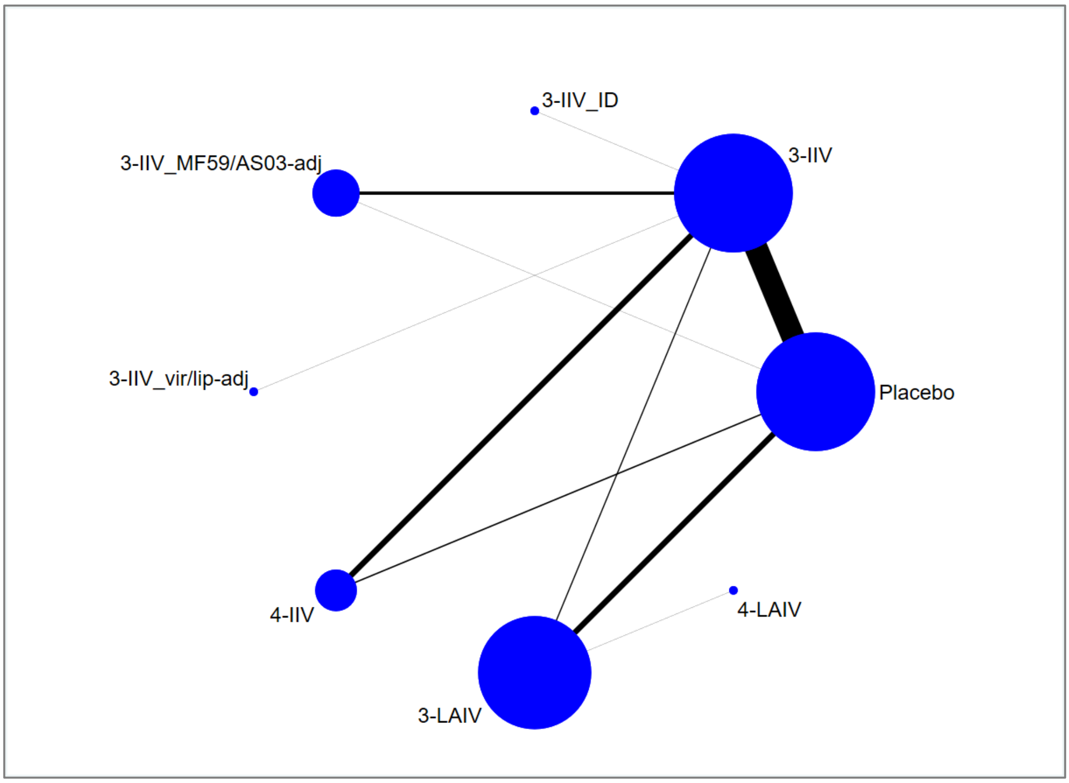

The thickness of the line is proportional to the precision of each direct estimate, and the width of each circle is proportional to the number of studies included in the treatment.

* **Placebo:** placebo/no vaccine; **3-IIV:** trivalent inactivated intramuscular; **3-IIV HD:** trivalent inactivated high dose intramuscular; **3-IIV ID:** trivalent inactivated intradermal; **3-IIV MF59/AS03-adj:** trivalent inactivated adjuvanted with MF59/ASO3 intramuscular: **3-IIV vir/lip-adj:** trivalent inactivated adjuvanted with Virosome/liposome intramuscular: **3-RIV:** trivalent recombinant intramuscular; **4-IIV:** quadrivalent inactivated intramuscular; **4-IIV HD:** quadrivalent inactivated high dose intramuscular: **4-IIV ID:** quadrivalent inactivated intradermal; **4-IIV MF59/AS03-adj:** quadrivalent inactivated adjuvanted with MF59/ASO3 intramuscular; **4-IIV vir/lip-adj:** quadrivalent inactivated adjuvanted with virosome/liposome intramuscular; **4-RIV:** quadrivalent recombinant intramuscular; **3-LAIV:** trivalent live attenuated intranasal; **4-LAIV:** quadrivalent live attenuated intranasal.

Children, aged ≤5 years: Forest plots of any systemic adverse event, comparison with placebo/no vaccine and with 3-IIV*

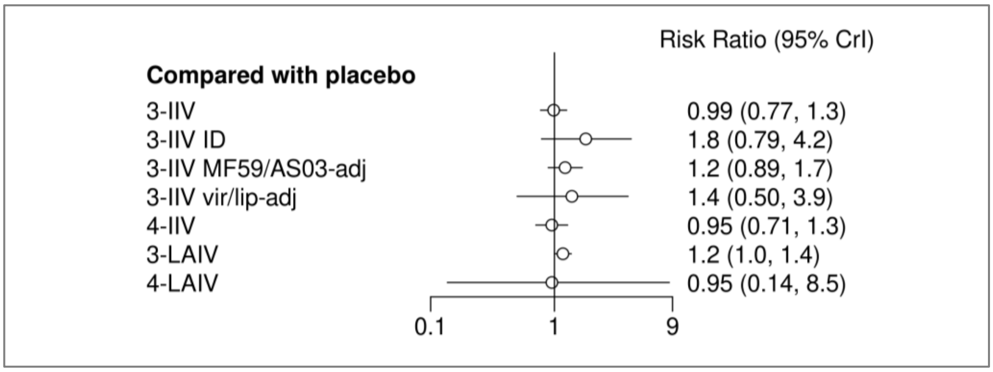

OR <1 favors experimental vaccine compared to placebo or 3-IIV

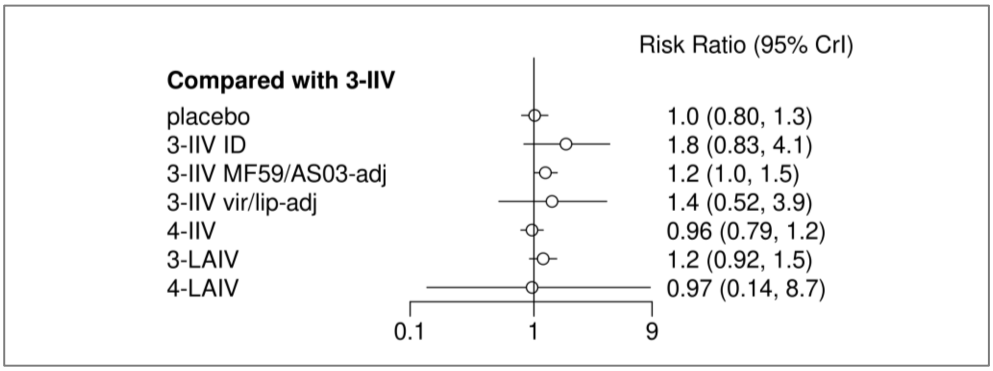

OR <1 favors experimental vaccine compared to placebo or 3-IIV

* **Placebo:** placebo/no vaccine; **3-IIV:** trivalent inactivated intramuscular; **3-IIV HD:** trivalent inactivated high dose intramuscular; **3-IIV ID:** trivalent inactivated intradermal; **3-IIV MF59/AS03-adj:** trivalent inactivated adjuvanted with MF59/ASO3 intramuscular: **3-IIV vir/lip-adj:** trivalent inactivated adjuvanted with Virosome/liposome intramuscular: **3-RIV:** trivalent recombinant intramuscular; **4-IIV:** quadrivalent inactivated intramuscular; **4-IIV HD:** quadrivalent inactivated high dose intramuscular: **4-IIV ID:** quadrivalent inactivated intradermal; **4-IIV MF59/AS03-adj:** quadrivalent inactivated adjuvanted with MF59/ASO3 intramuscular; **4-IIV vir/lip-adj:** quadrivalent inactivated adjuvanted with virosome/liposome intramuscular; **4-RIV:** quadrivalent recombinant intramuscular; **3-LAIV:** trivalent live attenuated intranasal; **4-LAIV:** quadrivalent live attenuated intranasal.

Children, aged ≤5 years: Netleague analysis of any systemic adverse event*

**placebo**	0.99 (0.77, 1.26)	1.79 (0.79, 4.18)	1.22 (0.89, 1.68)	1.38 (0.5, 3.94)	0.95 (0.71, 1.28)	1.17 (1, 1.37)	0.95 (0.14, 8.48)
1.01 (0.8, 1.29)	**3-IIV**	1.82 (0.83, 4.09)	1.24 (1, 1.54)	1.4 (0.52, 3.87)	0.96 (0.79, 1.19)	1.19 (0.92, 1.53)	0.97 (0.14, 8.7)
0.56 (0.24, 1.27)	0.55 (0.24, 1.2)	**3-IIV ID**	0.68 (0.29, 1.54)	0.77 (0.22, 2.76)	0.53 (0.23, 1.19)	0.65 (0.28, 1.49)	0.53 (0.07, 5.47)
0.82 (0.6, 1.12)	0.81 (0.65, 1)	1.47 (0.65, 3.4)	**3-IIV MF59/AS03-adj**	1.13 (0.41, 3.21)	0.78 (0.58, 1.05)	0.96 (0.69, 1.32)	0.78 (0.11, 7.05)
0.72 (0.25, 2)	0.71 (0.26, 1.92)	1.29 (0.36, 4.63)	0.88 (0.31, 2.43)	**3-IIV vir/lip-adj**	0.69 (0.24, 1.89)	0.85 (0.3, 2.35)	0.69 (0.08, 7.53)
1.05 (0.78, 1.41)	1.04 (0.84, 1.27)	1.88 (0.84, 4.35)	1.28 (0.96, 1.73)	1.45 (0.53, 4.1)	**4-IIV**	1.23 (0.9, 1.67)	1 (0.14, 9.13)
0.85 (0.73, 1)	0.84 (0.66, 1.08)	1.53 (0.67, 3.58)	1.04 (0.76, 1.45)	1.18 (0.43, 3.36)	0.81 (0.6, 1.11)	**3 LAIV**	0.81 (0.12, 7.16)
1.05 (0.12, 7.33)	1.03 (0.11, 7.32)	1.88 (0.18, 15.32)	1.28 (0.14, 9.15)	1.44 (0.13, 12.84)	1 (0.11, 7.11)	1.23 (0.14, 8.55)	**4 LAIV**

The estimate is located at the intersection of the column-defining vaccine and the row-defining vaccine. Data are RRs (95% CrI). Significant results are in bold. In the upper triangle, comparisons of treatments should be read from right to left. An RR below 1 favors the medication on the bottom right vs. the medication on the top left in the diagonal.

* **Placebo:** placebo/no vaccine; **3-IIV:** trivalent inactivated intramuscular; **3-IIV HD:** trivalent inactivated high dose intramuscular; **3-IIV ID:** trivalent inactivated intradermal; **3-IIV MF59/AS03-adj:** trivalent inactivated adjuvanted with MF59/ASO3 intramuscular: **3-IIV vir/lip-adj:** trivalent inactivated adjuvanted with Virosome/liposome intramuscular: **3-RIV:** trivalent recombinant intramuscular; **4-IIV:** quadrivalent inactivated intramuscular; **4-IIV HD:** quadrivalent inactivated high dose intramuscular: **4-IIV ID:** quadrivalent inactivated intradermal; **4-IIV MF59/AS03-adj:** quadrivalent inactivated adjuvanted with MF59/ASO3 intramuscular; **4-IIV vir/lip-adj:** quadrivalent inactivated adjuvanted with virosome/liposome intramuscular; **4-RIV:** quadrivalent recombinant intramuscular; **3-LAIV:** trivalent live attenuated intranasal; **4-LAIV:** quadrivalent live attenuated intranasal.

Adults and the elderly: Network geometry of any local adverse event*

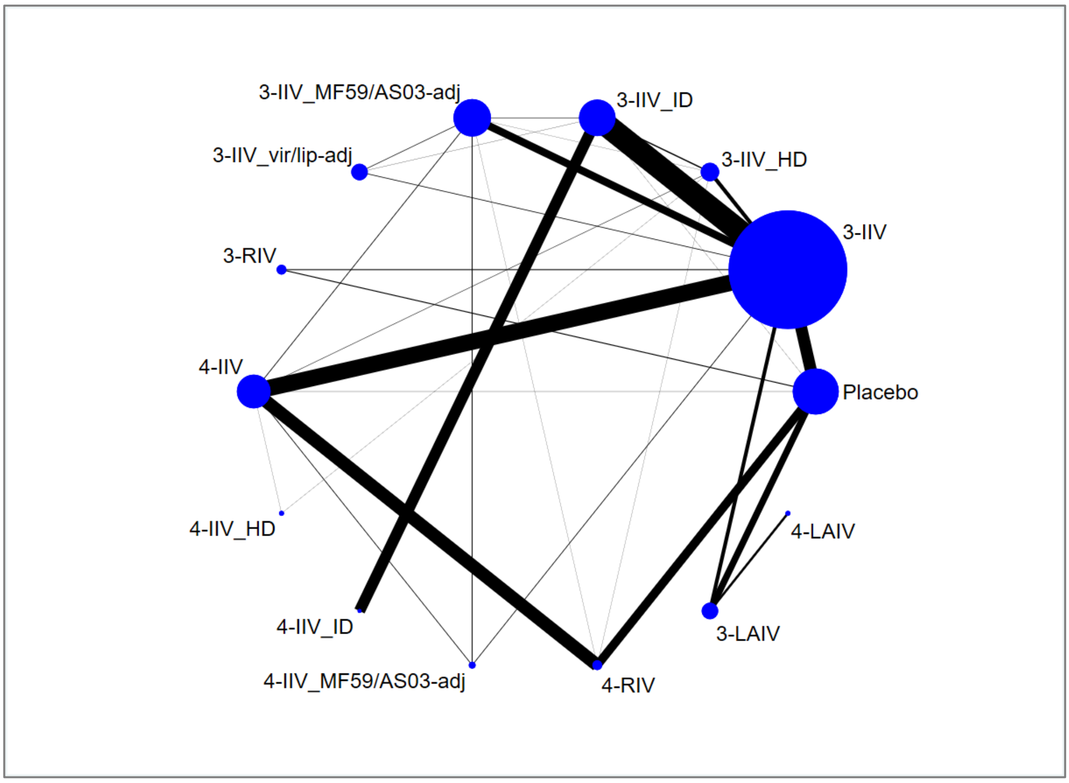

The thickness of the line is proportional to the precision of each direct estimate, and the width of each circle is proportional to the number of studies included in the treatment.

* **Placebo:** placebo/no vaccine; **3-IIV:** trivalent inactivated intramuscular; **3-IIV HD:** trivalent inactivated high dose intramuscular; **3-IIV ID:** trivalent inactivated intradermal; **3-IIV MF59/AS03-adj:** trivalent inactivated adjuvanted with MF59/ASO3 intramuscular: **3-IIV vir/lip-adj:** trivalent inactivated adjuvanted with Virosome/liposome intramuscular: **3-RIV:** trivalent recombinant intramuscular; **4-IIV:** quadrivalent inactivated intramuscular; **4-IIV HD:** quadrivalent inactivated high dose intramuscular: **4-IIV ID:** quadrivalent inactivated intradermal; **4-IIV MF59/AS03-adj:** quadrivalent inactivated adjuvanted with MF59/ASO3 intramuscular; **4-IIV vir/lip-adj:** quadrivalent inactivated adjuvanted with virosome/liposome intramuscular; **4-RIV:** quadrivalent recombinant intramuscular; **3-LAIV:** trivalent live attenuated intranasal; **4-LAIV:** quadrivalent live attenuated intranasal.

Adults and the elderly: Forest plots of any local adverse event, comparison with placebo/no vaccine and with 3-IIV[*]

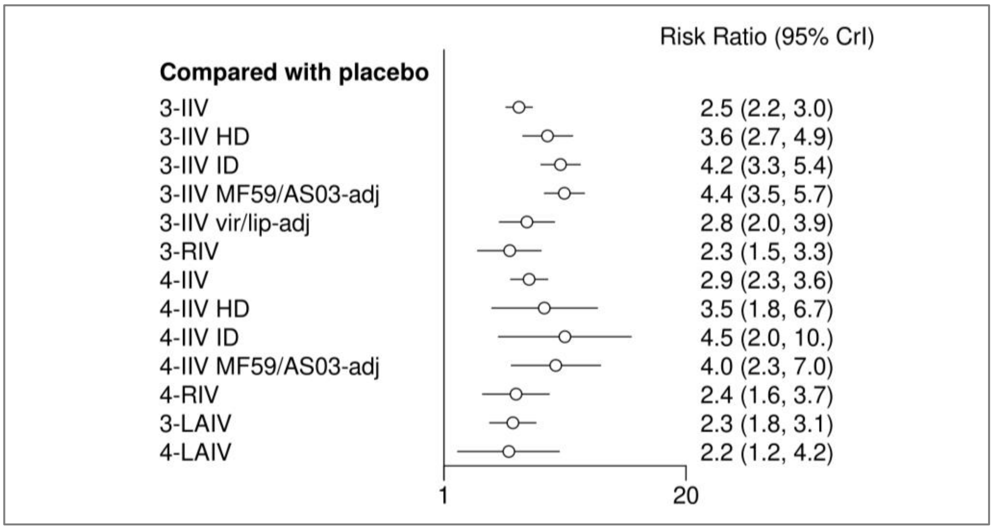

OR <1 favors experimental vaccine compared to placebo or 3-IIV

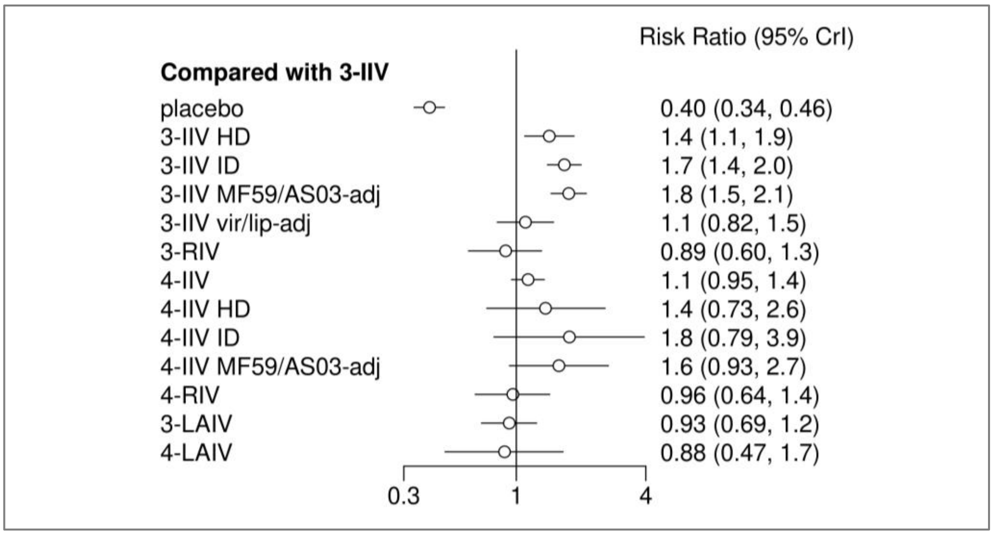

OR <1 favors experimental vaccine compared to placebo or 3-IIV

[*] **Placebo:** placebo/no vaccine; **3-IIV:** trivalent inactivated intramuscular; **3-IIV HD:** trivalent inactivated high dose intramuscular; **3-IIV ID:** trivalent inactivated intradermal; **3-IIV MF59/AS03-adj:** trivalent inactivated adjuvanted with MF59/ASO3 intramuscular: **3-IIV vir/lip-adj:** trivalent inactivated adjuvanted with Virosome/liposome intramuscular: **3-RIV:** trivalent recombinant intramuscular; **4-IIV:** quadrivalent inactivated intramuscular; **4-IIV HD:** quadrivalent inactivated high dose intramuscular: **4-IIV ID:** quadrivalent inactivated intradermal; **4-IIV MF59/AS03-adj:** quadrivalent inactivated adjuvanted with MF59/ASO3 intramuscular; **4-IIV vir/lip-adj:** quadrivalent inactivated adjuvanted with virosome/liposome intramuscular; **4-RIV:** quadrivalent recombinant intramuscular; **3-LAIV:** trivalent live attenuated intranasal; **4-LAIV:** quadrivalent live attenuated intranasal.

Adults and the elderly: Netleague analysis of any local adverse event*

**placebo**	**2.53 (2.16, 2.98)**	**3.6 (2.66, 4.91)**	**4.23 (3.32, 5.4)**	**4.44 (3.47, 5.69)**	**2.79 (1.99, 3.93)**	**2.26 (1.52, 3.33)**	**2.87 (2.29, 3.61)**	**3.46 (1.81, 6.68)**	**4.47 (1.96, 10.15)**	**3.99 (2.3, 6.95)**	**2.44 (1.61, 3.68)**	**2.35 (1.76, 3.12)**	**2.23 (1.18, 4.17)**
**0.4 (0.34, 0.46)**	**3-IIV**	**1.42 (1.09, 1.86)**	**1.67 (1.4, 2)**	**1.75 (1.45, 2.12)**	1.1 (0.82, 1.49)	0.89 (0.6, 1.31)	1.13 (0.95, 1.35)	1.37 (0.73, 2.59)	1.77 (0.79, 3.93)	1.58 (0.93, 2.68)	0.96 (0.64, 1.43)	0.93 (0.69, 1.24)	0.88 (0.47, 1.65)
**0.28 (0.2, 0.38)**	**0.7 (0.54, 0.91)**	**3-IIV HD**	1.18 (0.86, 1.6)	1.23 (0.89, 1.69)	0.77 (0.52, 1.15)	0.63 (0.39, 1)	0.8 (0.6, 1.07)	0.96 (0.52, 1.8)	1.24 (0.53, 2.87)	1.11 (0.61, 1.99)	0.68 (0.43, 1.07)	**0.65 (0.44, 0.96)**	0.62 (0.31, 1.22)
**0.24 (0.19, 0.3)**	**0.6 (0.5, 0.72)**	0.85 (0.62, 1.17)	**3-IIV ID**	1.05 (0.82, 1.34)	**0.66 (0.47, 0.92)**	**0.53 (0.35, 0.82)**	**0.68 (0.53, 0.87)**	0.82 (0.43, 1.59)	1.06 (0.48, 2.31)	0.94 (0.54, 1.64)	**0.58 (0.37, 0.89)**	**0.55 (0.39, 0.78)**	0.53 (0.27, 1.01)
**0.23 (0.18, 0.29)**	**0.57 (0.47, 0.69)**	0.81 (0.59, 1.12)	0.95 (0.75, 1.22)	**3-IIV MF59/AS03-adj**	**0.63 (0.46, 0.86)**	**0.51 (0.33, 0.78)**	**0.65 (0.5, 0.83)**	0.78 (0.41, 1.52)	1.01 (0.44, 2.29)	0.9 (0.53, 1.52)	**0.55 (0.35, 0.85)**	**0.53 (0.37, 0.75)**	**0.5 (0.26, 0.97)**
**0.36 (0.25, 0.5)**	0.91 (0.67, 1.23)	1.29 (0.87, 1.92)	**1.52 (1.09, 2.11)**	**1.59 (1.16, 2.18)**	**3-IIV vir/lip-adj**	0.81 (0.49, 1.32)	1.03 (0.73, 1.45)	1.24 (0.62, 2.51)	1.6 (0.68, 3.75)	1.43 (0.79, 2.6)	0.87 (0.53, 1.43)	0.84 (0.55, 1.28)	0.8 (0.4, 1.6)
**0.44 (0.3, 0.66)**	1.12 (0.76, 1.67)	1.6 (1, 2.57)	**1.88 (1.23, 2.89)**	**1.97 (1.28, 3.05)**	1.24 (0.76, 2.03)	**3-RIV**	1.27 (0.83, 1.95)	1.53 (0.73, 3.25)	1.98 (0.81, 4.86)	1.77 (0.92, 3.44)	1.08 (0.63, 1.87)	1.04 (0.65, 1.67)	0.99 (0.48, 2.07)
**0.35 (0.28, 0.44)**	0.88 (0.74, 1.05)	1.26 (0.94, 1.68)	**1.48 (1.15, 1.89)**	**1.55 (1.2, 1.99)**	0.97 (0.69, 1.37)	0.79 (0.51, 1.2)	**4-IIV**	1.21 (0.65, 2.28)	1.56 (0.68, 3.53)	1.39 (0.81, 2.4)	0.85 (0.58, 1.24)	0.82 (0.58, 1.15)	0.78 (0.4, 1.49)
**0.29 (0.15, 0.55)**	0.73 (0.39, 1.37)	1.04 (0.55, 1.94)	1.22 (0.63, 2.35)	1.28 (0.66, 2.46)	0.81 (0.4, 1.62)	0.65 (0.31, 1.37)	0.83 (0.44, 1.54)	**4-IIV HD**	1.29 (0.46, 3.58)	1.15 (0.51, 2.59)	0.7 (0.34, 1.45)	0.68 (0.33, 1.35)	0.64 (0.26, 1.57)
**0.22 (0.1, 0.51)**	0.57 (0.25, 1.27)	0.81 (0.35, 1.88)	0.95 (0.43, 2.08)	0.99 (0.44, 2.27)	0.62 (0.27, 1.46)	0.51 (0.21, 1.24)	0.64 (0.28, 1.47)	0.78 (0.28, 2.16)	**4-IIV ID**	0.89 (0.34, 2.34)	0.55 (0.22, 1.34)	0.53 (0.22, 1.24)	0.5 (0.18, 1.39)
**0.25 (0.14, 0.43)**	0.63 (0.37, 1.08)	0.9 (0.5, 1.63)	1.06 (0.61, 1.85)	1.11 (0.66, 1.87)	0.7 (0.39, 1.27)	0.57 (0.29, 1.09)	0.72 (0.42, 1.24)	0.87 (0.39, 1.97)	1.12 (0.43, 2.93)	**4-IIV MF59/AS03-adj**	0.61 (0.32, 1.17)	0.59 (0.32, 1.07)	0.56 (0.24, 1.27)
**0.41 (0.27, 0.62)**	1.04 (0.7, 1.55)	1.48 (0.93, 2.35)	**1.73 (1.12, 2.7)**	**1.82 (1.18, 2.82)**	1.14 (0.7, 1.89)	0.93 (0.53, 1.6)	1.18 (0.81, 1.73)	1.42 (0.69, 2.96)	1.83 (0.75, 4.5)	1.64 (0.85, 3.17)	**4-RIV**	0.96 (0.59, 1.57)	0.91 (0.44, 1.91)
**0.43 (0.32, 0.57)**	1.08 (0.8, 1.45)	**1.53 (1.04, 2.29)**	**1.8 (1.28, 2.55)**	**1.89 (1.34, 2.69)**	1.19 (0.78, 1.81)	0.96 (0.6, 1.54)	1.22 (0.87, 1.72)	1.48 (0.74, 2.99)	1.9 (0.81, 4.49)	1.7 (0.93, 3.14)	1.04 (0.64, 1.69)	**3-LAIV**	0.95 (0.54, 1.66)
**0.45 (0.24, 0.84)**	1.13 (0.61, 2.14)	1.61 (0.82, 3.23)	1.9 (0.99, 3.68)	**1.99 (1.03, 3.85)**	1.25 (0.62, 2.52)	1.01 (0.48, 2.1)	1.29 (0.67, 2.48)	1.55 (0.64, 3.82)	2 (0.72, 5.58)	1.79 (0.79, 4.1)	1.09 (0.52, 2.29)	1.05 (0.6, 1.84)	**4-LAIV**

The estimate is located at the intersection of the column-defining vaccine and the row-defining vaccine. Data are RRs (95% CrI). Significant results are in bold. In the upper triangle, comparisons of treatments should be read from right to left. An RR below 1 favors the medication on the bottom right vs. the medication on the top left in the diagonal.

* **Placebo:** placebo/no vaccine; **3-IIV:** trivalent inactivated intramuscular; **3-IIV HD:** trivalent inactivated high dose intramuscular; **3-IIV ID:** trivalent inactivated intradermal; **3-IIV MF59/AS03-adj:** trivalent inactivated adjuvanted with MF59/ASO3 intramuscular: **3-IIV vir/lip-adj:** trivalent inactivated adjuvanted with Virosome/liposome intramuscular: **3-RIV:** trivalent recombinant intramuscular; **4-IIV:** quadrivalent inactivated intramuscular; **4-IIV HD:** quadrivalent inactivated high dose intramuscular: **4-IIV ID:** quadrivalent inactivated intradermal; **4-IIV MF59/AS03-adj:** quadrivalent inactivated adjuvanted with MF59/ASO3 intramuscular; **4-IIV vir/lip-adj:** quadrivalent inactivated adjuvanted with virosome/liposome intramuscular; **4-RIV:** quadrivalent recombinant intramuscular; **3-LAIV:** trivalent live attenuated intranasal; **4-LAIV:** quadrivalent live attenuated intranasal.

All children: Network geometry of any local adverse event*

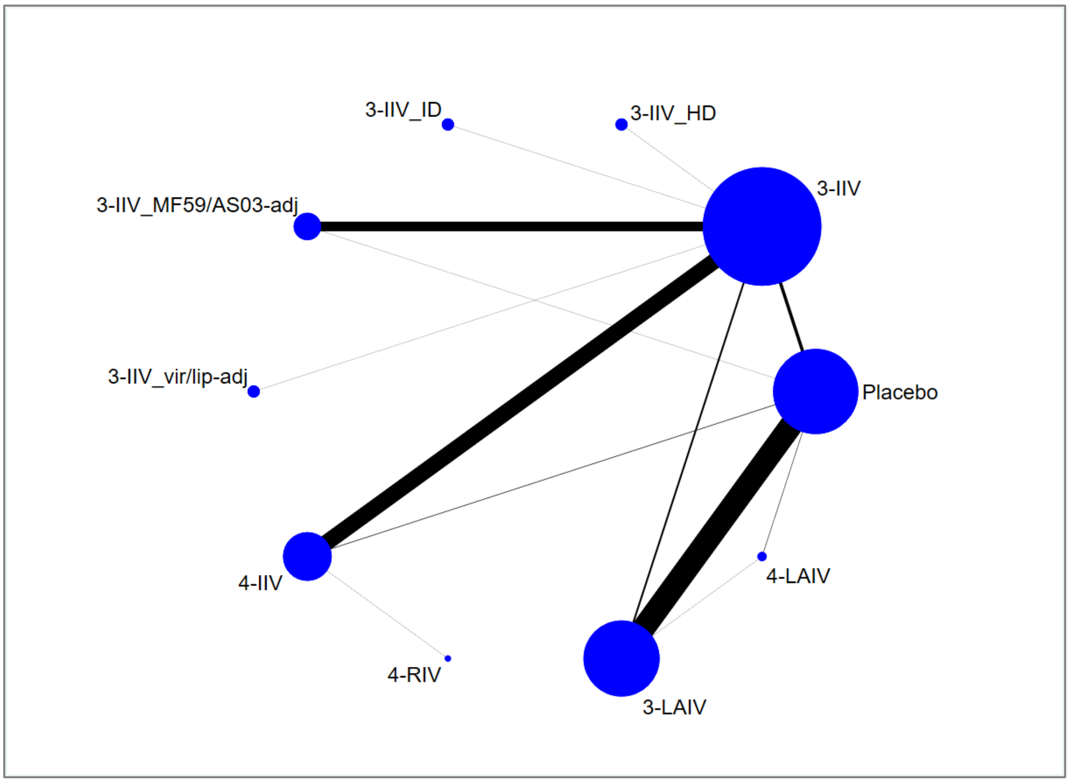

The thickness of the line is proportional to the precision of each direct estimate, and the width of each circle is proportional to the number of studies included in the treatment.

* **Placebo:** placebo/no vaccine; **3-IIV:** trivalent inactivated intramuscular; **3-IIV HD:** trivalent inactivated high dose intramuscular; **3-IIV ID:** trivalent inactivated intradermal; **3-IIV MF59/AS03-adj:** trivalent inactivated adjuvanted with MF59/ASO3 intramuscular: **3-IIV vir/lip-adj:** trivalent inactivated adjuvanted with Virosome/liposome intramuscular: **3-RIV:** trivalent recombinant intramuscular; **4-IIV:** quadrivalent inactivated intramuscular; **4-IIV HD:** quadrivalent inactivated high dose intramuscular: **4-IIV ID:** quadrivalent inactivated intradermal; **4-IIV MF59/AS03-adj:** quadrivalent inactivated adjuvanted with MF59/ASO3 intramuscular; **4-IIV vir/lip-adj:** quadrivalent inactivated adjuvanted with virosome/liposome intramuscular; **4-RIV:** quadrivalent recombinant intramuscular; **3-LAIV:** trivalent live attenuated intranasal; **4-LAIV:** quadrivalent live attenuated intranasal.

All children: Forest plots of any local adverse event, comparison with placebo/no vaccine and with 3-IIV*

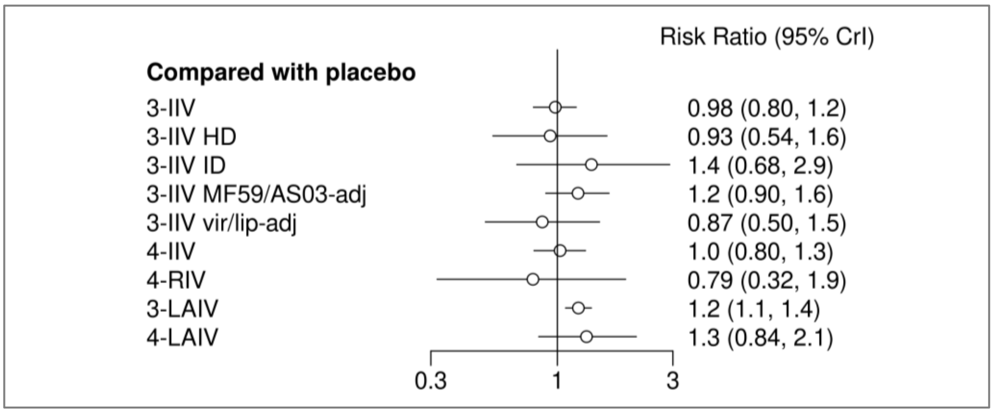

OR <1 favors experimental vaccine compared to placebo or 3-IIV

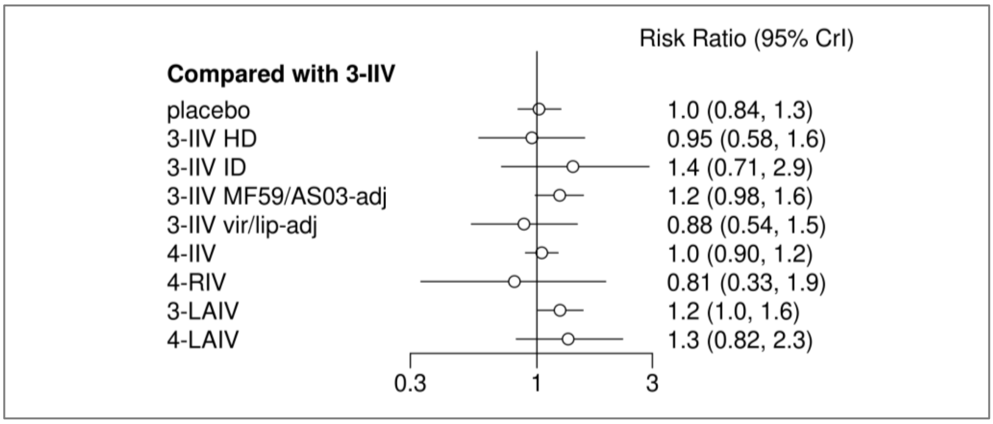

OR <1 favors experimental vaccine compared to placebo or 3-IIV

* **Placebo:** placebo/no vaccine; **3-IIV:** trivalent inactivated intramuscular; **3-IIV HD:** trivalent inactivated high dose intramuscular; **3-IIV ID:** trivalent inactivated intradermal; **3-IIV MF59/AS03-adj:** trivalent inactivated adjuvanted with MF59/ASO3 intramuscular: **3-IIV vir/lip-adj:** trivalent inactivated adjuvanted with Virosome/liposome intramuscular: **3-RIV:** trivalent recombinant intramuscular; **4-IIV:** quadrivalent inactivated intramuscular; **4-IIV HD:** quadrivalent inactivated high dose intramuscular: **4-IIV ID:** quadrivalent inactivated intradermal; **4-IIV MF59/AS03-adj:** quadrivalent inactivated adjuvanted with MF59/ASO3 intramuscular; **4-IIV vir/lip-adj:** quadrivalent inactivated adjuvanted with virosome/liposome intramuscular; **4-RIV:** quadrivalent recombinant intramuscular; **3-LAIV:** trivalent live attenuated intranasal; **4-LAIV:** quadrivalent live attenuated intranasal.

All children: Netleague analysis of any local adverse event*

**placebo**	0.98 (0.8, 1.2)	0.93 (0.54, 1.6)	1.38 (0.68, 2.91)	1.22 (0.9, 1.63)	0.87 (0.5, 1.49)	1.03 (0.8, 1.31)	0.79 (0.32, 1.91)	**1.22 (1.08, 1.38)**	1.32 (0.84, 2.12)
1.02 (0.84, 1.26)	**3-IIV**	0.95 (0.58, 1.57)	1.41 (0.71, 2.91)	1.24 (0.98, 1.56)	0.88 (0.54, 1.47)	1.05 (0.9, 1.22)	0.81 (0.33, 1.92)	**1.25 (1.01, 1.55)**	1.35 (0.82, 2.26)
1.07 (0.62, 1.85)	1.05 (0.64, 1.74)	**3-IIV HD**	1.48 (0.64, 3.6)	1.3 (0.75, 2.26)	0.93 (0.46, 1.91)	1.1 (0.65, 1.86)	0.85 (0.31, 2.3)	1.31 (0.76, 2.27)	1.41 (0.7, 2.92)
0.72 (0.34, 1.47)	0.71 (0.34, 1.4)	0.68 (0.28, 1.57)	**3-IIV ID**	0.88 (0.41, 1.8)	0.63 (0.26, 1.47)	0.74 (0.36, 1.49)	0.57 (0.18, 1.72)	0.88 (0.42, 1.8)	0.95 (0.4, 2.27)
0.82 (0.61, 1.12)	0.81 (0.64, 1.02)	0.77 (0.44, 1.34)	1.14 (0.56, 2.44)	**3-IIV MF59/AS03-adj**	0.71 (0.41, 1.25)	0.84 (0.64, 1.12)	0.65 (0.26, 1.6)	1 (0.74, 1.38)	1.09 (0.63, 1.92)
1.15 (0.67, 1.98)	1.13 (0.68, 1.86)	1.08 (0.52, 2.18)	1.6 (0.68, 3.85)	1.4 (0.8, 2.43)	**3-IIV vir/lip-adj**	1.18 (0.7, 2)	0.91 (0.33, 2.5)	1.41 (0.81, 2.43)	1.52 (0.75, 3.14)
0.97 (0.77, 1.25)	0.95 (0.82, 1.11)	0.91 (0.54, 1.54)	1.35 (0.67, 2.82)	1.19 (0.9, 1.56)	0.84 (0.5, 1.44)	**4-IIV**	0.77 (0.32, 1.81)	1.19 (0.93, 1.54)	1.29 (0.77, 2.2)
1.26 (0.52, 3.14)	1.24 (0.52, 3.01)	1.18 (0.43, 3.26)	1.76 (0.58, 5.44)	1.54 (0.62, 3.84)	1.09 (0.4, 3.06)	1.3 (0.55, 3.12)	**4-RIV**	1.54 (0.64, 3.84)	1.67 (0.62, 4.68)
**0.82 (0.72, 0.92)**	**0.8 (0.64, 0.99)**	0.76 (0.44, 1.32)	1.13 (0.55, 2.39)	1 (0.73, 1.35)	0.71 (0.41, 1.23)	0.84 (0.65, 1.08)	0.65 (0.26, 1.57)	**3-LAIV**	1.08 (0.68, 1.75)
0.76 (0.47, 1.19)	0.74 (0.44, 1.22)	0.71 (0.34, 1.43)	1.05 (0.44, 2.51)	0.92 (0.52, 1.58)	0.66 (0.32, 1.33)	0.78 (0.45, 1.3)	0.6 (0.21, 1.62)	0.93 (0.57, 1.47)	**4-LAIV**

The estimate is located at the intersection of the column-defining vaccine and the row-defining vaccine. Data are RRs (95% CrI). Significant results are in bold. In the upper triangle, comparisons of treatments should be read from right to left. An RR below 1 favors the medication on the bottom right vs. the medication on the top left in the diagonal.

* **Placebo:** placebo/no vaccine; **3-IIV:** trivalent inactivated intramuscular; **3-IIV HD:** trivalent inactivated high dose intramuscular; **3-IIV ID:** trivalent inactivated intradermal; **3-IIV MF59/AS03-adj:** trivalent inactivated adjuvanted with MF59/ASO3 intramuscular: **3-IIV vir/lip-adj:** trivalent inactivated adjuvanted with Virosome/liposome intramuscular: **3-RIV:** trivalent recombinant intramuscular; **4-IIV:** quadrivalent inactivated intramuscular; **4-IIV HD:** quadrivalent inactivated high dose intramuscular: **4-IIV ID:** quadrivalent inactivated intradermal; **4-IIV MF59/AS03-adj:** quadrivalent inactivated adjuvanted with MF59/ASO3 intramuscular; **4-IIV vir/lip-adj:** quadrivalent inactivated adjuvanted with virosome/liposome intramuscular; **4-RIV:** quadrivalent recombinant intramuscular; **3-LAIV:** trivalent live attenuated intranasal; **4-LAIV:** quadrivalent live attenuated intranasal.

Children with pre-existing respiratory diseases: Network geometry of exacerbation chronic respiratory disease*

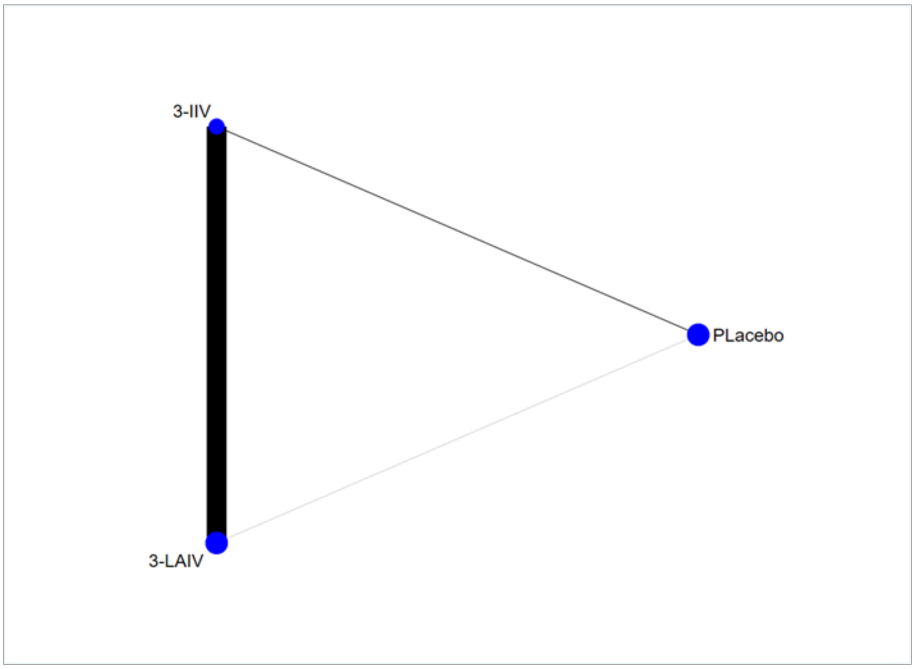

The thickness of the line is proportional to the precision of each direct estimate, and the width of each circle is proportional to the number of studies included in the treatment.

Children with pre-existing respiratory diseases: Forest plots of exacerbation chronic respiratory disease, comparison with placebo-no vaccine and with 3-IIV*

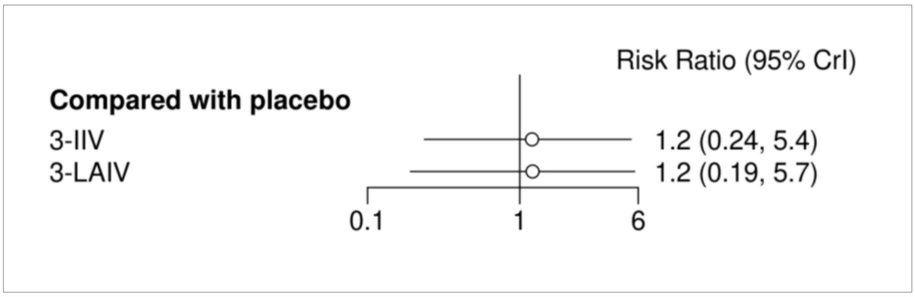

OR <1 favors experimental vaccine compared to placebo or 3-IIV

* **Placebo:** placebo/no vaccine; **3-IIV:** trivalent inactivated intramuscular; **3-IIV HD:** trivalent inactivated high dose intramuscular; **3-IIV ID:** trivalent inactivated intradermal; **3-IIV MF59/AS03-adj:** trivalent inactivated adjuvanted with MF59/ASO3 intramuscular: **3-IIV vir/lip-adj:** trivalent inactivated adjuvanted with Virosome/liposome intramuscular: **3-RIV:** trivalent recombinant intramuscular; **4-IIV:** quadrivalent inactivated intramuscular; **4-IIV HD:** quadrivalent inactivated high dose intramuscular: **4-IIV ID:** quadrivalent inactivated intradermal; **4-IIV MF59/AS03-adj:** quadrivalent inactivated adjuvanted with MF59/ASO3 intramuscular; **4-IIV vir/lip-adj:** quadrivalent inactivated adjuvanted with virosome/liposome intramuscular; **4-RIV:** quadrivalent recombinant intramuscular; **3-LAIV:** trivalent live attenuated intranasal; **4-LAIV:** quadrivalent live attenuated intranasal.

Children with pre-existing respiratory diseases: Netleague analysis of exacerbation chronic respiratory disease*

**placebo**	1.2 (0.24, 5.39)	1.21 (0.19, 5.68)
0.83 (0.19, 4.22)	**3-IIV**	1.02 (0.2, 4.26)
0.82 (0.18, 5.22)	0.98 (0.23, 5.05)	**3-LAIV**

The estimate is located at the intersection of the column-defining vaccine and the row-defining vaccine. Data are RRs (95% CrI). Significant results are in bold. In the upper triangle, comparisons of treatments should be read from right to left. An RR below 1 favors the medication on the bottom right vs. the medication on the top left in the diagonal.

* **Placebo:** placebo/no vaccine; **3-IIV:** trivalent inactivated intramuscular; **3-IIV HD:** trivalent inactivated high dose intramuscular; **3-IIV ID:** trivalent inactivated intradermal; **3-IIV MF59/AS03-adj:** trivalent inactivated adjuvanted with MF59/ASO3 intramuscular: **3-IIV vir/lip-adj:** trivalent inactivated adjuvanted with Virosome/liposome intramuscular: **3-RIV:** trivalent recombinant intramuscular; **4-IIV:** quadrivalent inactivated intramuscular; **4-IIV HD:** quadrivalent inactivated high dose intramuscular: **4-IIV ID:** quadrivalent inactivated intradermal; **4-IIV MF59/AS03-adj:** quadrivalent inactivated adjuvanted with MF59/ASO3 intramuscular; **4-IIV vir/lip-adj:** quadrivalent inactivated adjuvanted with virosome/liposome intramuscular; **4-RIV:** quadrivalent recombinant intramuscular; **3-LAIV:** trivalent live attenuated intranasal; **4-LAIV:** quadrivalent live attenuated intranasal.

SUCRA Adults and the elderly*

vaccine	laboratory confirmed influenza	any systemic adverse events
3-IIV HD	0.80	0.22
3-IIV MF59/AS03-adj	0.69	0.15
3-RIV	0.67	0.87
3-IIV	0.66	0.64
3-IIV ID	0.56	0.58
4-RIV	0.49	0.40
4-IIV	0.30	0.56
3-LAIV	0.29	0.35
placebo	0.02	0.89
3-IIV vir/lip-adj		0.93
4-IIV HD		0.44
4-IIV ID		0.48
4-IIV MF59/AS03-adj		0.07
4-LAIV		0.41

SUCRA probability any Children*

vaccine	laboratory confirmed influenza	any systemic adverse events
3-IIV MF59/AS03-adj	0.95	
3-LAIV	0.79	0.34
4-IIV	0.47	0.66
3-IIV	0.42	0.63
4-LAIV	0.29	0.07
placebo	0.07	0.60
3-IIV HD		0.77
3-IIV ID		0.37
3-IIV vir/lip-adj		0.34
4-IIV vir/lip-adj		0.25
4-RIV		0.94

* **Placebo:** placebo/no vaccine; **3-IIV:** trivalent inactivated intramuscular; **3-IIV HD:** trivalent inactivated high dose intramuscular; **3-IIV ID:** trivalent inactivated intradermal; **3-IIV MF59/AS03-adj:** trivalent inactivated adjuvanted with MF59/ASO3 intramuscular: **3-IIV vir/lip-adj:** trivalent inactivated adjuvanted with Virosome/liposome intramuscular: **3-RIV:** trivalent recombinant intramuscular; **4-IIV:** quadrivalent inactivated intramuscular; **4-IIV HD:** quadrivalent inactivated high dose intramuscular: **4-IIV ID:** quadrivalent inactivated intradermal; **4-IIV MF59/AS03-adj:** quadrivalent inactivated adjuvanted with MF59/ASO3 intramuscular; **4-IIV vir/lip-adj:** quadrivalent inactivated adjuvanted with virosome/liposome intramuscular; **4-RIV:** quadrivalent recombinant intramuscular; **3-LAIV:** trivalent live attenuated intranasal; **4-LAIV:** quadrivalent live attenuated intranasal.

Analyses of outcomes and subgroups not performed because of paucity of data

The elderly >61 years: hospitalization, mortality, ILI, any local AE

Children < 5 years: hospitalization, mortality, ILI, any local AE, acute otitis media

Adult immunocompromised/cancer: hospitalization, mortality, ILI, any local AE

Adult with pre-existing chronic respiratory disease: laboratory confirmed influenza, hospitalization, mortality, ILI, any systemic AE, any local AE, exacerbation pre-existing chronic respiratory disease

Children immunocompromised/cancer: laboratory confirmed influenza, hospitalization, mortality, ILI, any systemic AE, any local AE

Children with pre-existing chronic respiratory disease: hospitalization, mortality, ILI, any systemic AE, any local AE
